# Laboratory mouse housing conditions can be improved using common environmental enrichment without compromising data

**DOI:** 10.1371/journal.pbio.2005019

**Published:** 2018-04-16

**Authors:** Viola André, Christine Gau, Angelika Scheideler, Juan A. Aguilar-Pimentel, Oana V. Amarie, Lore Becker, Lillian Garrett, Wolfgang Hans, Sabine M. Hölter, Dirk Janik, Kristin Moreth, Frauke Neff, Manuela Östereicher, Ildiko Racz, Birgit Rathkolb, Jan Rozman, Raffi Bekeredjian, Jochen Graw, Martin Klingenspor, Thomas Klopstock, Markus Ollert, Carsten Schmidt-Weber, Eckhard Wolf, Wolfgang Wurst, Valérie Gailus-Durner, Markus Brielmeier, Helmut Fuchs, Martin Hrabé de Angelis

**Affiliations:** 1 Research Unit Comparative Medicine, Helmholtz Zentrum München, German Research Center for Environmental Health, Neuherberg, Germany; 2 German Mouse Clinic, Institute of Experimental Genetics, Helmholtz Zentrum München, German Research Center for Environmental Health, Neuherberg, Germany; 3 Institute of Developmental Genetics, Helmholtz Zentrum München, German Research Center for Environmental Health, Neuherberg, Germany; 4 Institute of Pathology, Helmholtz Zentrum München, German Research Center for Environmental Health, Neuherberg, Germany; 5 Institute of Molecular Psychiatry, University of Bonn, Bonn, Germany; 6 Institute of Molecular Animal Breeding and Biotechnology, Gene Center, Ludwig-Maximilians-Universität München, Munich, Germany; 7 German Center for Diabetes Research (DZD), Neuherberg, Germany; 8 Department of Cardiology, University of Heidelberg, Heidelberg, Germany; 9 Molecular Nutritional Medicine, Else Kröner-Fresenius Center, Technische Universität München, Freising-Weihenstephan, Germany; 10 ZIEL—Center for Nutrition and Food Sciences, Technische Universität München, Freising, Germany; 11 Department of Neurology, Friedrich-Baur-Institute, Klinikum der Ludwig-Maximilians-Universität München, Munich, Germany; 12 Deutsches Institut für Neurodegenerative Erkrankungen (DZNE), Site Munich, Munich, Germany; 13 Munich Cluster for Systems Neurology (SyNergy), Adolf-Butenandt-Institut, Ludwig-Maximilians-Universität München, Munich, Germany; 14 Department of Dermatology and Allergy Center, Odense Research Center for Anaphylaxis (ORCA), Odense University hospital, University of Southern Denmark, Odense C, Denmark; 15 Department of Infection and Immunity, Luxembourg Institute of Health (LIH), Esch-sur-Alzette, Luxembourg; 16 Center of Allergy & Environment (ZAUM), Technische Universität München and Helmholtz Zentrum München, Munich, Germany and Member of the German Center for Lung Research (DZL), Gießen, Germany; 17 Technische Universität München, Freising-Weihenstephan, Chair of Developmental Genetics, c/o Helmholtz Zentrum München, Neuherberg, Germany; 18 Max Planck Institute of Psychiatry, Munich, Germany; 19 Chair of Experimental Genetics, School of Life Science Weihenstephan, Technische Universität München, Freising, Germany; University College London, United Kingdom of Great Britain and Northern Ireland

## Abstract

Animal welfare requires the adequate housing of animals to ensure health and well-being. The application of environmental enrichment is a way to improve the well-being of laboratory animals. However, it is important to know whether these enrichment items can be incorporated in experimental mouse husbandry without creating a divide between past and future experimental results. Previous small-scale studies have been inconsistent throughout the literature, and it is not yet completely understood whether and how enrichment might endanger comparability of results of scientific experiments. Here, we measured the effect on means and variability of 164 physiological parameters in 3 conditions: with nesting material with or without a shelter, comparing these 2 conditions to a “barren” regime without any enrichments. We studied a total of 360 mice from each of 2 mouse strains (C57BL/6NTac and DBA/2NCrl) and both sexes for each of the 3 conditions. Our study indicates that enrichment affects the mean values of some of the 164 parameters with no consistent effects on variability. However, the influence of enrichment appears negligible compared to the effects of other influencing factors. Therefore, nesting material and shelters may be used to improve animal welfare without impairment of experimental outcome or loss of comparability to previous data collected under barren housing conditions.

## Author summary

Adequate housing of laboratory animals is essential to guarantee their well-being. From a scientific perspective, physically and mentally healthy animals also contribute to increased validity and reproducibility of experimental results. The choice of nesting material or shelter type, referred to as environmental enrichment, may influence how laboratory animals perform species-specific behaviors. Consequently, changes in these nesting and shelter materials could influence scientific results by, for example, increasing variability in measured characteristics. Whether studies using different environmental enrichment materials can be compared is currently questioned. Our study shows that simple, species-specific environmental enrichment in the form of nesting material alone or in combination with a shelter did not consistently increase variability of physiological parameters in mice. Differences in parameter average values appeared to be of minor biological relevance when compared to the effects of other environmental factors. These simple environmental enrichment devices may therefore be applied to improve the housing environment of laboratory mice without compromising data validity or comparability.

## Introduction

The provision of species-appropriate environmental enrichment—which can be defined as additions to the cage environment that allow natural motivated behaviors enabling the animals to control their environment [1]—is generally promoted as a way to improve animal welfare [1,2] and is also legally requested within the European Union by Directive 2010/63/EU [3]. However, there are various kinds of enrichment items available, and their imprudent application might interfere with the comparability of scientific results as even “seemingly minor alterations in the environment can have significant effects on experimental outcomes” [4]. A study by Macri et al. suggests that the adoption of environmental enrichment according to Directive 2010/63/EU might strongly influence the conclusions drawn from pharmacological and behavioral studies. In their study, they tested a synthetic cannabinoid compound and concluded that “whether the compound shall be considered a cannabinoid agonist may strongly depend on the specific conditions in which mice are reared” [5]. This underlines the potential risk following the implementation of inconsiderate enrichment strategies without preceding evaluation.

The term environmental enrichment is widely applied and also includes experimental paradigms, where intensive environmental enrichment strategies are used to explore effects of a more complex environment [6]. This so-called “super-enrichment”—as, for example, described in a protocol by Slater et al. [7]—induces behavioral [8], emotional [9,10], physiological [11], and neurobiological [10,12,13] changes in mice compared to barren housing. Moreover, such environmental enrichment can improve pathological conditions. For example, enrichment can suppress tumor growth and reduce adiposity [7] and alleviate the intensities of various phenotypes in animal models (see [14] for an overview).

Besides such use of (super)enrichment as an experimental tool, simple enrichment is regularly used in laboratory animal facilities to ensure health and well-being of the animals and to meet their physiological and ethological needs as much as possible. It is thus applied as a refinement strategy according to the Three Rs (3Rs) of Russell and Burch [15]. The results of studies that determine the effects of those more commonly used forms of enrichment are varied.

Some authors found that nesting material influenced behavioral parameters of mice [16] or affected the scientific outcome in a well-described mouse model for allergic asthma [17]. Shelters as enrichment also altered motor coordination and some behavioral parameters in mice [18]. Furthermore, several studies revealed that even simple forms of enrichment, like nesting material [19], shelter [20], shelter combined with a scaffolding [21], or labyrinths [22], affected aggression and stress-related parameters in male mice of certain strains. Others found no effects of nesting material [23] or a shelter [24] on behavior and some physiological parameters.

Moreover, there has been much discussion about possible consequences of environmental enrichment on variability in data. Concern has been expressed by some authors that the response to and experience with environmental enrichment might differ between individuals so that environmental enrichment might lead to a higher variability in physiological parameters [25–28]. This might add to the individual variability within groups [29] and thereby increase the number of animals needed to reach statistical power. On the other hand, a multilaboratory study by Wolfer et al. found that environmental enrichment did not increase within-group variability in 19 out of 20 parameters of 4 behavioral tests in female mice [30]. Richter et al. describe a different approach and suggested that a conscious standardized heterogenization of environmental conditions might indeed increase within-experiment variation but then might also lead to lower between-experiment variation and therefore might improve external validity of experiments. An efficient heterogenization strategy is yet to be determined, though [31–33]. But while an environmental heterogenization strategy might give information about generalizability, according to van der Staay et al., there is the risk that subtle effects might be missed [34]. The choice of study design (strict standardization versus heterogenization) is therefore heavily dependent on the scientific questions a study aims to answer [34].

Taken together, despite a diverse body of literature, the assessment of the actual influence of enrichment remains difficult because the existing literature is, in large part, based on (single-cohort) studies that examine environmental enrichment effects under very specific conditions in respect to examined strain, sex, enrichment device, and parameters. But direction and size of effects seem to vary depending on the type and combination of enrichment [35], the mouse strain [36,37], sex [38], time and duration of enrichment [39], and which parameters were studied [26]; the applicability across rodent species, strains, sexes, and ages is uncertain [40]. To prevent unclear or spurious results and the need for higher numbers of animals due to increased variability within groups, a systematic evaluation of enrichment strategies is crucial [41,42], and environmental enrichment interventions should be “carefully selected, thoroughly defined, and purposefully used” [4]. This is of special importance in light of the ongoing debate about reproducibility because certain environmental factors might account for irreproducible rodent experiments [43,44] and variation or lack of standardization in environmental enrichment strategies might contribute to problems with reproducibility in preclinical research [4]. Kent et al. conclude in the ACLAM position statement on reproducibility that “it is incumbent on laboratory animal veterinarians and the scientific community to define elements of study design that affect experimental reproducibility” [45].

**Fig 1:**
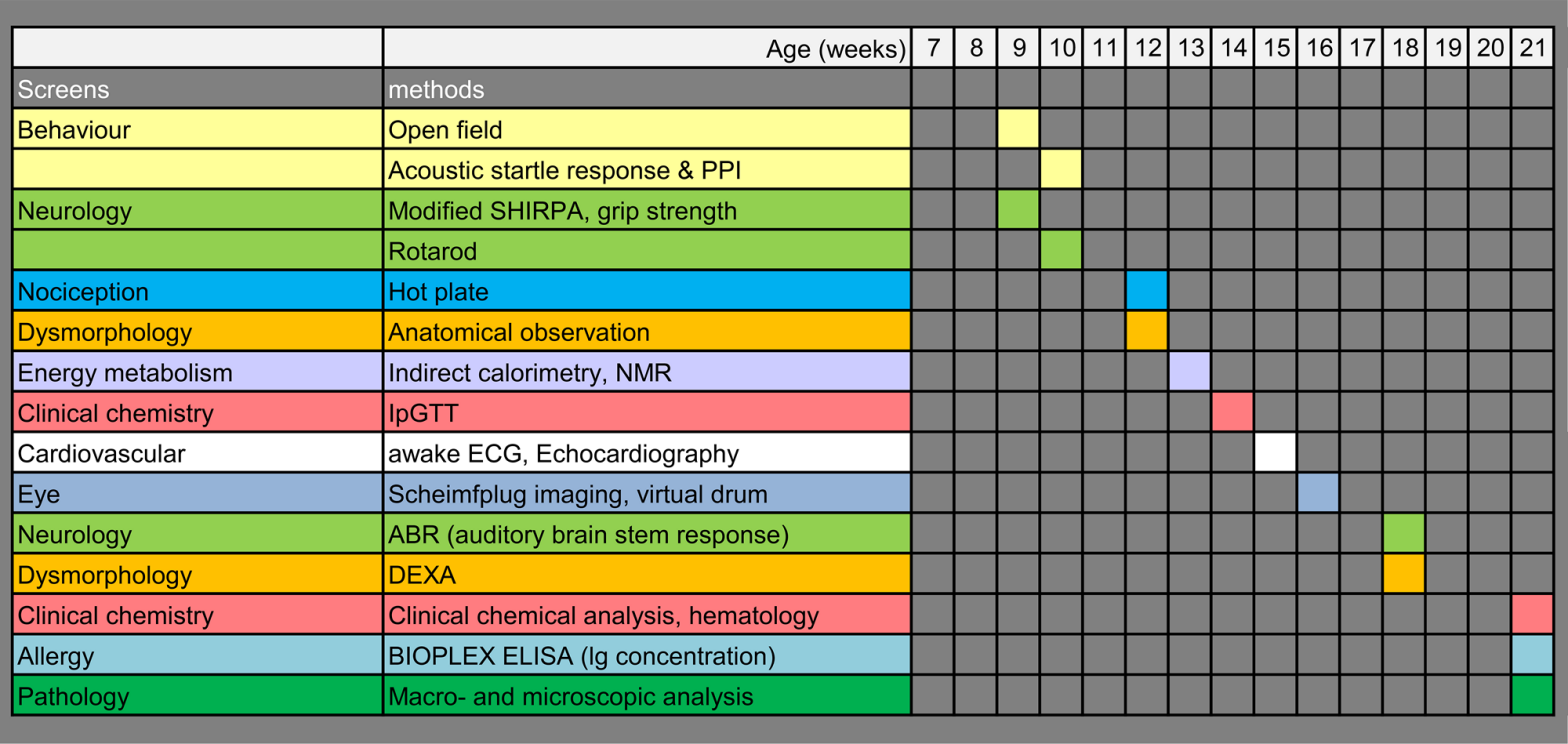
Workflow for the screens of this study. DEXA, dual-energy x-ray absorptiometry; IpGTT, intraperitoneal glucose tolerance test; NMR, nuclear magnetic resonance; PPI, pre-pulse inhibition; SHIRPA, Smithkline Beecham, MRC Harwell, Imperial College, the Royal London Hospital Phenotype Assessment.

Our study approach was therefore to investigate the effect of 2 simple, commonly used, and easily applicable enrichment items—namely, a shelter and nesting material (nestlet) versus nestlet alone or none of the above—on means and variability (expressed by coefficients of variation [CVs]) of a wide range of physiological parameters in a systematic, highly standardized study design (see Fig 1). To this end, we wanted to find out whether those environmental enrichments would change results of future and follow-up studies compared to the former standard of barren housing. For our purposes, we also specifically wished to ascertain whether environmental enrichment alters the measuring system of the German Mouse Clinic [46–48]. We examined male and female wild-type mice of 2 commonly used inbred mouse strains, C57BL/6NTac (B6) and DBA/2NCrl (D2). The study was conducted in 3 replicates using 3 independent cohorts of mice to give information about variability.

## Results

**Fig 2:**
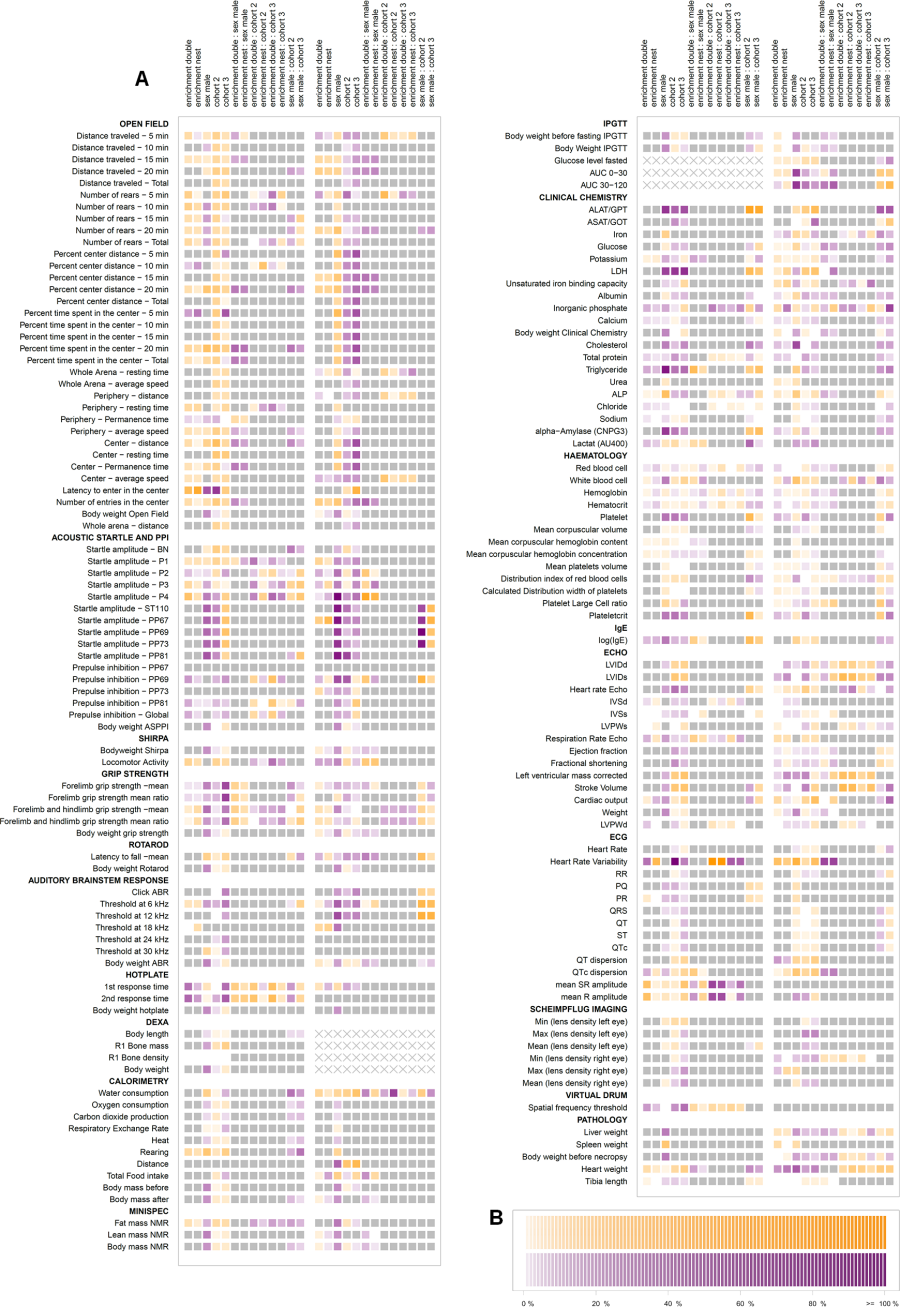
Influence of main factors (enrichment, sex, cohort) on mean of parameters in 2 mouse strains. (A) The heatmap shows the results for all metric parameters (rows) and influencing factors and their double interactions (columns). The B6 and D2 data are on the left and right of each column, respectively. The influence of main factors was evaluated using linear models. A variable selection using the BIC was performed. Reference categories are enrichment none, sex female, and the first cohort. The differences between means of parameters are expressed as color-coded estimators β of the respective model in percent of the intercept of the model. The estimator β describes the change induced by the respective factors. The intercept is the mean of the parameter of the reference group and is set as 100%. Grey shade: variable selection using the BIC yielded nonrelevant influencing factor, thus no effect can be assumed; violet shade: factor is relevant for the model, i.e., mean is increased compared to reference group; yellow shade: factor is relevant for the model, i.e., mean is decreased compared to reference group; strength of color reflects size of difference. “X” marks parameters that were not analyzed. (B) Color grading scheme: the color grading illustrates the change in percent of the intercept; the strength of color reflects the size of difference. ABR, auditory brain stem response; ALAT/GPT, Alanine aminotransferase/Glutamat pyruvat transaminase; ALP, Alkaline phosphatase; ASAT/GOT, Aspartate aminotransferase/Glutamat oxalacetat transaminase; ASR, acoustic startle response; AU400, name of clinical chemistry analyzer; AUC, area under the curve; BIC, Bayesian Information Criterion; BN, background noise; CNPG3, substrate of test reaction (2-Chloro-4-Nitrophenyl-α-D-Maltotriosid); DEXA, dual-energy x-ray absorptiometry; HR, heart rate; HRV, heart rate variability; IgE, immunoglobulin E; IpGTT, intraperitoneal glucose tolerance test; LDH, Lactat-dehydrogenase; LVIDd, left ventricular end-diastolic internal diameter; LVIDs, left ventricular end-systolic internal diameter; LVPW, left ventricular posterior wall; P1-P4, startle amplitude following pulse of 67 (P1), 69 (P2), 73 (P3), 81 (P4) dB; PP (67, 69, 73, 81), startle amplitude and PPI following startle pulse (110 dB) with preceding pre-pulse of 67, 69, 73, 81 dB; PPI, pre-pulse inhibition; qNMR, quantitative nuclear magnetic resonance; QTc, corrected QT; R1, region analyzed (whole body excluding the skull); SHIRPA, Smithkline Beecham, MRC Harwell, Imperial College, the Royal London Hospital Phenotype Assessment; ST 110, acoustic startle response at 110 dB.

The aim of this study was to evaluate the influence of simple environmental enrichment on the mean and variability of a broad range of physiological parameters in 2 strains of mice. The parameters we chose covered the areas of behavior, dysmorphology, neurology, clinical chemistry and hematology, eye, allergy, energy metabolism, pain perception, cardiovascular health, and pathology. Details of the parameters can be depicted from Fig 2 (quantitative parameters) and S1 Table, S2 Table, and S1 Text (qualitative parameters). To gain information about variability, 3 independent cohorts of mice were measured.

### Environmental enrichment effects on mean values of quantitative physiological traits

We analyzed the influence of environmental enrichment on the mean of our parameters using linear models with “main factors”: enrichment, sex, and cohort. We used a variable selection using the Bayesian Information Criterion (BIC) to determine differences between the experimental groups. The reference group was the unenriched females from cohort 1.

The influence of main factors (enrichment, sex, cohort) on the mean of all metric parameters and their double interactions is shown as a heatmap (Fig 2) with strength of color reflecting the size of difference. If the variable selection yielded a variable as nonrelevant, it was not included in the model, and the parameter was marked as grey (i.e., no difference) in the heatmap (Fig 2).

**Fig 3:**
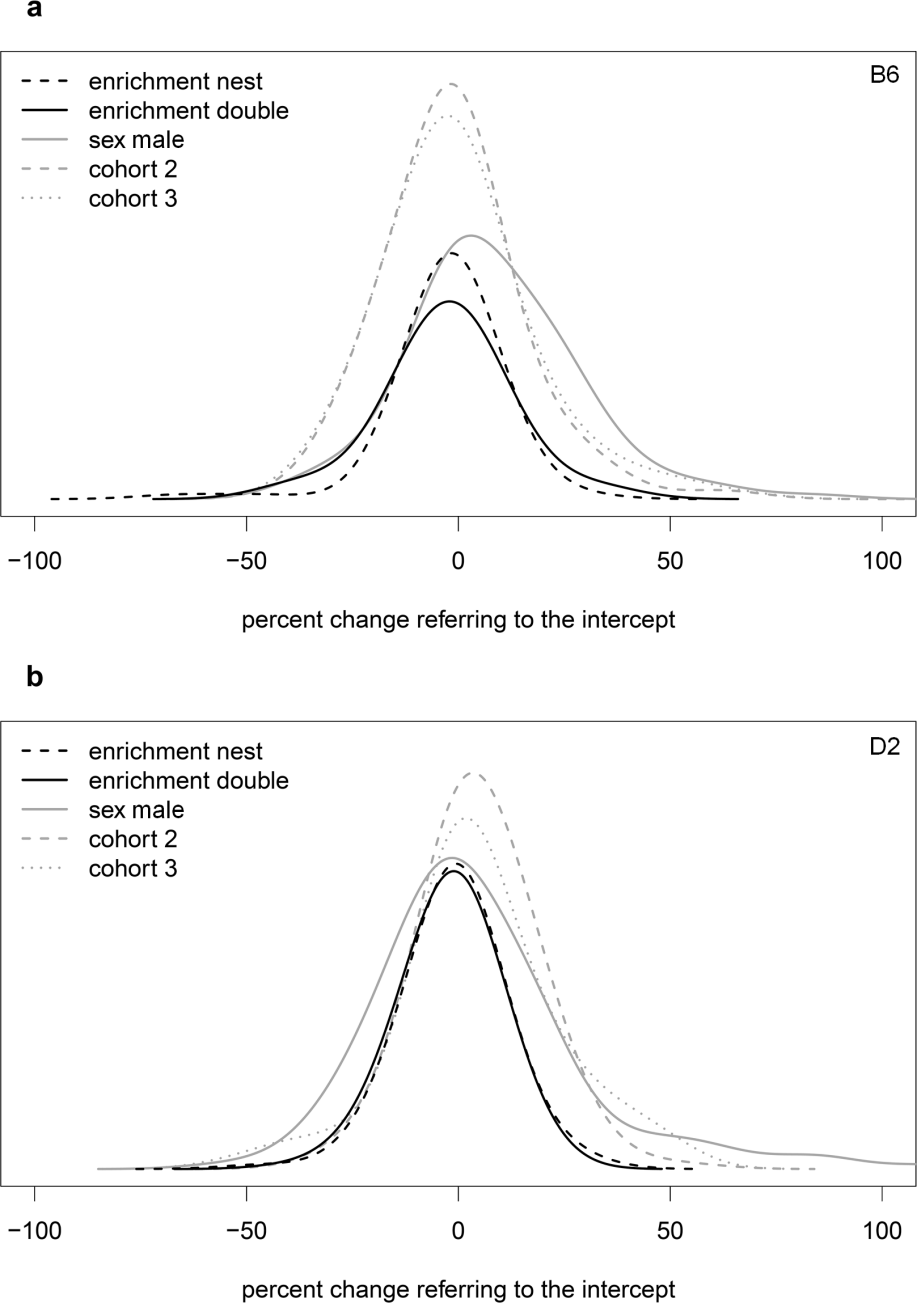
Smoothed histogram showing the influence of main factors (enrichment, sex, cohort) on means in 2 strains. The influence is expressed as differences in percent of intercept for B6 (a) and D2 (b) mice. The intercept is the mean of the parameter of the reference group. The area under the curve is proportional to number of parameters, for which the variable selection using the BIC yielded relevance (yellow and violet shading in Fig 2); nonselected parameters (grey in the heatmap of Fig 2) are not included (number of selected factors for B6 [a]: enrichment nest: 69; enrichment double: 69; sex male: 118; cohort 2: 152; cohort 3: 152; number of selected factors for D2 [b]: enrichment nest: 88; enrichment double: 88; sex male: 144; cohort 2: 135; cohort 3: 135). The height of the histogram is calculated as density multiplied by total number of parameters and is proportional to number of parameters that fall in the respective bin of the x-axis.

To better illustrate the effects of enrichment compared to the effects of factors sex and cohort, we computed smoothed histograms (Fig 3) of parameters that were influenced by the respective main factors.

**Fig 4:**
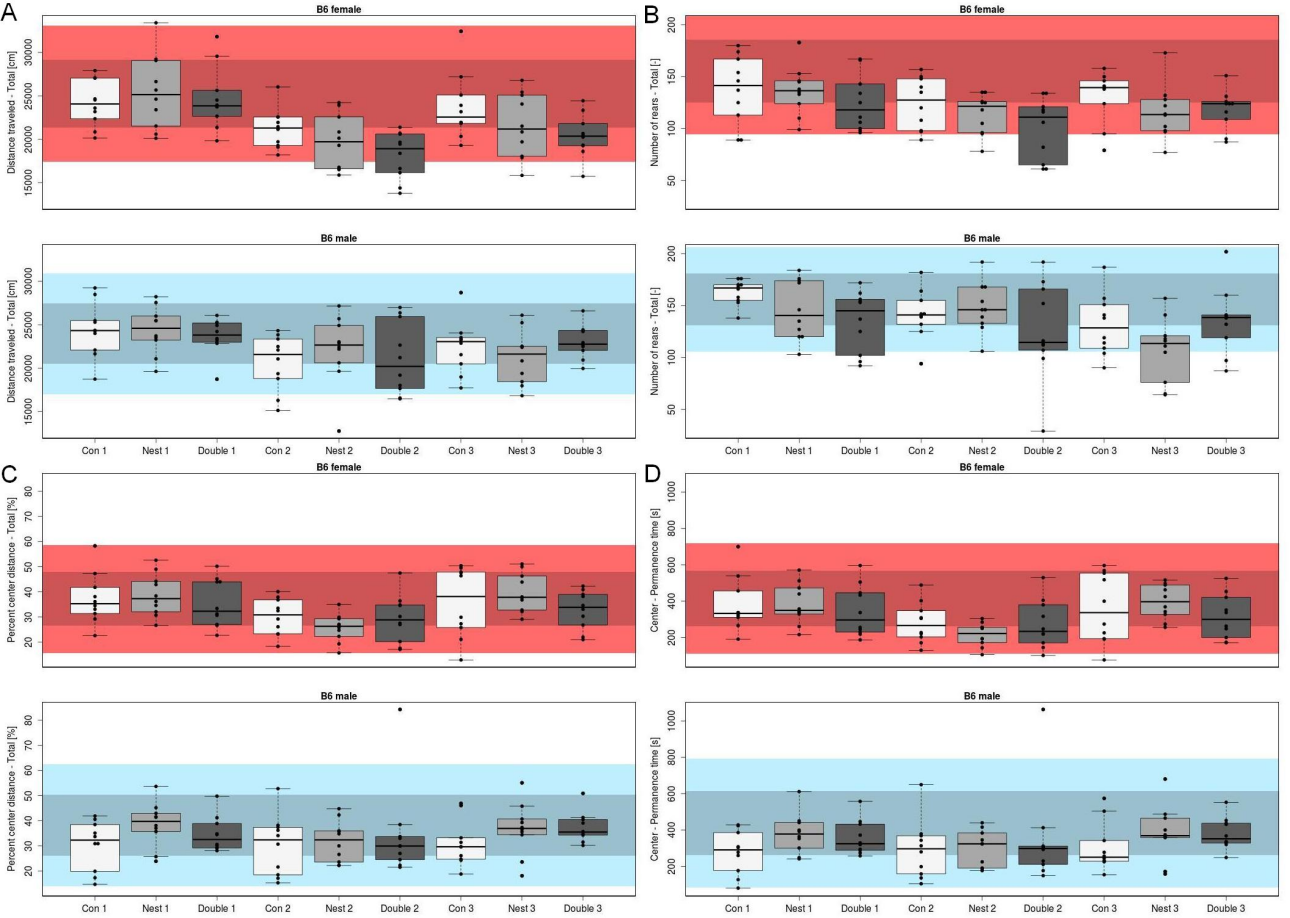
Raw data of selected parameters on the background of the biological range for B6. Raw data of the 4 main parameters of the open field test: (A) “distance traveled total,” (B) “number of rears total,” (C) “percent center distance total,” and (D) “center permanence time” as box- and whisker-plots. The box represents 25th percentile, median, and 75th percentile; the length of whiskers is maximally the 1.5-fold interquartile range but is determined by the last value within this range. All individual values are shown for each experimental group (“con,” nest, double) and every cohort (1, 2, 3) for female and male mice in the upper and lower plot, respectively, for each selected parameter (A–D). The range in the background gives 1 SD (dark shading) and 2 SD (bright shading) of >200 reference B6 female (red) and male (blue) mice. The reference mice were same-aged wild-type control mice from other phenotyping projects of the GMC and were measured within the same timespan as the mice used in this project. “con”, control; GMC, German Mouse Clinic.

For an exemplary evaluation of the biological relevance of our findings in the open field test, we prepared boxplots of raw data for the 4 main parameters on which we normally focus. They are “distance traveled total” (index of locomotor activity), “number of rears total” (index of exploratory activity), “percent center distance total,” and “center permanence time” (indices of anxiety-related behavior). Furthermore, in each plot we included data of more than 200 B6 wild-type animals, which were measured as control mice in phenotyping projects of the German Mouse Clinic (GMC). Because these B6 were the same age and used in the same timespan as the mice of this study, the data can serve as a biological range for B6 mice (Fig 4).

Raw data in form of boxplots (S1 Fig) and individual values (S1 Data) for every parameter are provided in Supporting information.

Some aspects of our analysis are illustrated below by considering the 2 different mouse strains used (B6 and D2) individually.

### Specific analysis of the means of the physiological parameters for the B6 strain

In B6 mice, the means of 69 out of 161 parameters were changed by enrichment; a higher number of parameters was affected by sex (118/161) and cohort (152/161).

Overall, percentage differences of the mean of the respective groups and their control were distinctly higher for factor sex and cohort than for factor enrichment (Fig 3A). Considerably fewer parameters were influenced by enrichment than by the other main factors as can be seen by the smaller area under the curve for factor enrichment compared to factors sex and cohort. Both enrichment curves were narrower than the curves of the other main factors. This indicates that the influence of enrichment was considerably smaller than that of the other main factors.

A closer look at single research areas in the heatmap in Fig 2 reveals that some were rather robust against housing effects. The measured parameters of the following tests were mainly unchanged by enrichment compared to controls in B6: rotarod (neurology), Scheimpflug imaging (eye), auditory brain stem response (ABR; neurology), indirect calorimetry, and quantitative nuclear magnetic resonance (qNMR; energy metabolism). Other tests were more susceptible to effects of enrichment; in the open field test (behavior), 20 out of 34 parameters were influenced by nest and double enrichment. The percentage difference between mean of nest- and double-enriched groups and mean of controls ranged between 1% and 15% in 17 out of 20 (double enrichment) and 18 out of 20 (nest enrichment) parameters. Two of the 4 main parameters of the open field test (“number of rears total” and “center permanence time”) were affected by enrichment. For those parameters, most values lay within the biological range of B6 (Fig 4). As expected, there were sex differences for many parameters. Although our tests were performed under strictly standardized conditions, there were differences between the cohort replicates for a wide range of 152 out of 161 parameters.

### Specific analysis of the means of the physiological parameters for the D2 strain

For D2 mice, a difference between enriched and nonenriched mice could be found in 88 out of 160 parameters. For the other main factors, 144 out of 160 (sex) and 135 out of 160 (cohort) parameters were affected by the respective factors.

Overall, as in B6, fewer parameters were influenced by factor enrichment than by other main factors, which is expressed by the smaller area under the curve for factor enrichment in comparison to the other main factors (Fig 3B). Curves of double and nest enrichment were narrower than those of other main factors, which indicate that the influence of enrichment, on average, was smaller than the influence of factors cohort and sex.

Again, Fig 2 reveals that some tests appeared to be less sensitive towards effects of environmental enrichment than others, for example, Scheimpflug imaging and virtual drum (eye), indirect calorimetry (energy metabolism), immunoglobulin E (IgE; allergy), and ECG (cardiovascular). As in B6, factor sex also influenced a large part of parameters with partially prominent effects in D2 mice. Factor cohort also influenced many parameters regarding mean values; 135 out of 160 parameters were changed by cohort in D2 mice.

### Environmental enrichment effects on variability of measured parameters (expressed as CVs)

To evaluate the impact of environmental enrichment on variability, we used bootstrapped samples of the original data. Therefore, we drew a sample from each subgroup 1,000 times. As a comparable measure for variability, the CV was used.

Bootstrapped CV values were then analyzed with linear models, using enrichment, sex, and cohort as main factors.

**Fig 5:**
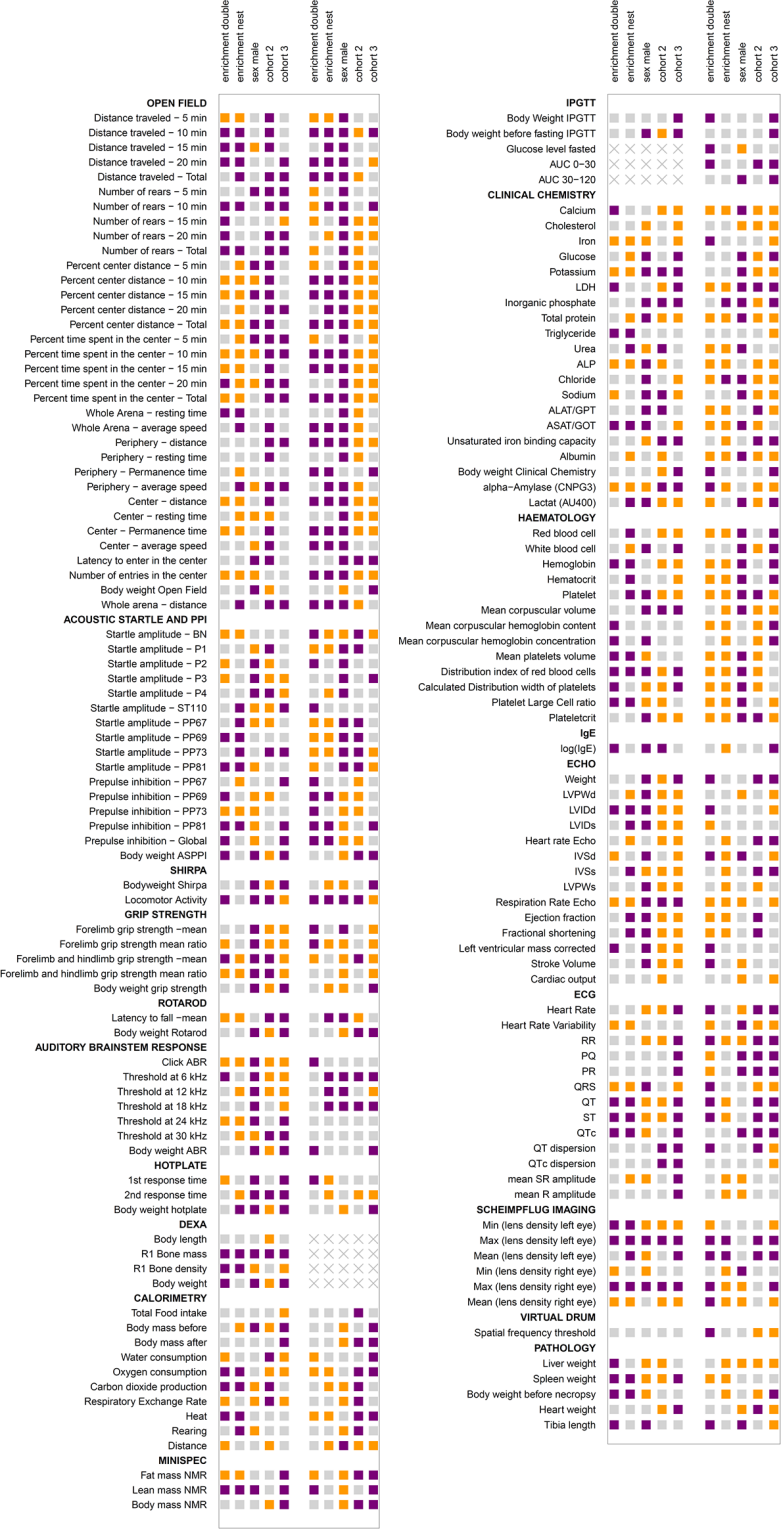
Influence of main factors (enrichment, sex, cohort) on bootstrapped CVs in 2 strains. The heatmap shows the results for all metric parameters (rows) and influencing factors (columns) for B6 (left column) and D2 (right column). Reference categories are enrichment none, sex female, and the first cohort. Results of the bootstrap method are summarized by computed confidence intervals for estimators (β), and bootstrapped CVs are then classified into the following 3 categories: confidence interval for β includes 0, thus no effect is assumed (grey); confidence interval for β is greater than 0, i.e., bootstrapped CVs are increased compared to reference group (violet); confidence interval for β is below 0, i.e., bootstrapped CVs are decreased compared to reference group (yellow). “X” mark parameters that were not analyzed. ABR, auditory brain stem response; ALAT/GPT, Alanine aminotransferase/Glutamat pyruvat transaminase; ALP, Alkaline phosphatase; ASAT/GOT, Aspartate aminotransferase/Glutamat oxalacetat transaminase; ASR, acoustic startle response; AU400, name of clinical chemistry analyzer; AUC, area under the curve; BIC, Bayesian Information Criterion; BN, background noise; CNPG3, substrate of test reaction (2-Chloro-4-Nitrophenyl-α-D-Maltotriosid); CV, coefficient of variation; DEXA, dual-energy x-ray absorptiometry; HR, heart rate; HRV, heart rate variability; IgE, immunoglobulin E; IpGTT, intraperitoneal glucose tolerance test; LDH, Lactat-dehydrogenase; LVIDd, left ventricular end-diastolic internal diameter; LVIDs, left ventricular end-systolic internal diameter; LVPW, left ventricular posterior wall; P1-P4, startle amplitude following pulse of 67 (P1), 69 (P2), 73 (P3), 81 (P4) dB; PP (67, 69, 73, 81), startle amplitude and PPI following startle pulse (110 dB) with preceding pre-pulse of 67, 69, 73, 81 dB; PPI, pre-pulse inhibition; qNMR, quantitative nuclear magnetic resonance; QTc, corrected QT; R1, region analyzed (whole body excluding the skull); SHIRPA, Smithkline Beecham, MRC Harwell, Imperial College, the Royal London Hospital Phenotype Assessment; ST 110, acoustic startle response at 110 dB.

The influence of the main factors on bootstrapped CVs is shown as a heatmap (Fig 5). Results of the bootstrap method are summarized by computed confidence intervals for estimators (β), and bootstrapped CVs were then classified into the following 3 categories: β includes 0, thus no effect is assumed (grey); confidence interval for β is greater than 0, i.e., bootstrapped CVs are increased compared to reference group (violet); confidence interval for β is below 0, i.e., bootstrapped CVs are decreased compared to reference group (yellow).

### Specific analysis of the variability of the physiological parameters

In B6 mice, CVs of 84 out of 161 and 93 out of 161 parameters were affected (either increased or decreased) by double enrichment and nest enrichment, respectively, compared to controls. For D2 mice, double enrichment influenced CVs of 101 out of 160 parameters, and nest enrichment affected 91 out of 160 parameters in comparison to controls. Overall, no distinct patterns could be observed that hinted towards a general increase or decrease of CVs of parameters in a certain test due to factor enrichment. Rather, within tests, CVs of individual parameters were increased, decreased, or not changed concomitantly (Fig 5).

For the other main factors, CVs of 107 out of 161 (sex), 114 out of 161 (cohort 2), and 112 out of 161 (cohort 3) parameters were influenced in B6; in D2, CVs of 114 out of 160 (sex), 101 out of 160 (cohort 2), and 114 out of 160 (cohort 3) parameters were changed.

Overall, no clear indication could be found that factor enrichment induced higher CVs neither in B6 nor in D2. However, enrichment (double and nest) influenced variability in fewer parameters than sex and cohort in both strains of mice.

### Categorical data

No effects of different housing conditions were found on categorical and qualitative data relevant for detection of abnormalities (in Smithkline Beecham, MRC Harwell, Imperial College, the Royal London Hospital Phenotype Assessment [SHIRPA] of the neurology screen, see S1 Table; in morphological examination of the dysmorphology screen, see S1 Text; in histopathological examination of the pathology screen, see S2 Table). Data of these tests were not included in the linear model analysis.

## Discussion

The aim of this study was to investigate the effects of commonly used environmental enrichment on a comprehensive range of physiological parameters that cover key experimental procedures of medical research. Three independent cohorts of mice of 2 strains and both sexes with 3 different housing conditions were measured in a highly standardized study design.

Mean values of about half of our quantitative parameters were affected by enriched housing in both strains (B6: 43%; D2: 55%). However, the differences found were mostly small, and the biological relevance still has to be interpreted separately for each parameter. For example, in our study, two-thirds of the parameters of the open field test were changed by enrichment in B6 mice. But many of those parameters are correlated with each other, so that if one is changed, the others change concurrently, which might seem to inflate the number of affected parameters. Moreover, the differences of most parameters were rather small (1%–15%). To further evaluate the biological relevance of our findings in the open field test, we compared results of the 4 main parameters (“distance traveled total,” “number of rears total,” “percent center distance total,” and “center permanence time”), on which we normally focus with the biological range of B6 mice. Two of the 4 mentioned parameters of the open field test (“number of rears total” and “center permanence time”) were affected by enrichment. However, these effects could not be observed in all 3 cohorts, and even for these metrics, most of the measured values also lay within the biological range of B6 mice. This suggests that the effects of factor enrichment were within the regular variation that can be seen between different cohorts.

It has already been shown that large effects—e.g., strain differences in behavioral testing—could be reproduced in the environment of different laboratories despite differences in absolute values [49,50]. The fact that we did not find biologically relevant effects of simple enrichment on, e.g., behavior, while others did [16] might indicate that the effects were too small to be found consistently.

On the other hand, we observed that some tests and parameters were robust towards the influence of environmental enrichment, as they were not changed. For some parameters, our results provide the same indication as studies in which no effects of enrichment were found in rotarod [16], body weight [27,51], food intake [51,52], or liver and spleen weight [27] in B6 mice. Other studies observed changes of body weight [23,52] and food intake [23] due to certain kinds of environmental enrichment, but—unlike them—our results did not hint towards changes in body weight. The differential findings between the studies can in part be explained by different study designs because factors like type and combination of enrichment [35], examined strain [36,37], sex [38], time and duration of enrichment [39], and studied parameters [26] influence the effects of environmental enrichment.

In our study, however, all those factors were standardized, and other environmental factors seemed to influence parameters equally or even more so than factor enrichment. These are represented by factor cohort, which changed 94% (B6) and 84% (D2) of examined parameters. Compared to factor enrichment, influence of cohort was apparent for more parameters, and on average, the observed effect was also stronger. Cohort effects have already been described as temporal variation [53] in phenotyping studies and can be attributed to differing body weights [54], seasonal variation [55], uncontrolled noise [56], sex of the experimenter [57], differing experimenters [58], and probably other factors that are yet unknown [4]. Tests in our study were conducted by differing experimenters of both sexes, which might have contributed to variation between cohorts [57,58]. Tail handling of mice has been shown to induce anxiety, which might have influenced behavioral results in our study [59,60], and battery testing itself can also induce additional noise [61] and add to the differences between cohorts. Influence of the above-discussed environmental factors attributing to cohort effects could also be accountable for differential study results that examine effects of simple enrichment in a single-cohort design. Furthermore, other (yet unknown) environmental factors might interfere with the measurements of the effects of enrichment. In a recent study, scientists failed to reproduce the finding that environmental enrichment decreased tumor growth [62] and also concluded that “other environmental factors are likely acting either in concert with or against environmental enrichment conditions to provide the variable results found” [63].

Altogether, even though we found that enrichment affects the mean of some parameters, its overall influence appeared to be of minor biological relevance in the background of the stronger environmental effects represented by the cohort. However, our study was designed to give a broad overview of possible effects of simple enrichment on a large number of physiological parameters of different research fields. Because we did not test an a priori hypothesis, our statistical analysis did not include a measure of statistical significance. Providing such a broad summary is helpful and can be used by other researchers to focus on single parameters of interest in confirmative studies. Furthermore, for logistical reasons, a blind outcome assessment was not possible in our study. However, most of the conducted tests are considered to be robust to subjective bias because animals are examined with the help of technical devices and parameters are digitally recorded and analyzed. We did not address the question, whether the used enrichment benefits animal welfare or provides a more realistic scenario in terms of resembling a natural environment. It is well known that barren housing conditions can cause impaired brain development and abnormal repetitive behaviors [13,64], which can compromise the validity of animal experiments and add variation [65]. However, the results we present here are, to our knowledge, the first systematic comparison of simple forms of enrichment with the former state-of-the-art—i.e., barren housing—on a large number of physiological parameters.

Apart from analyzing effects of common enrichment items on mean values, our second point of interest was in examining the effect on variability. We found that environmental enrichment influenced the CVs of 52% to 63% of parameters with no clear tendency towards an increase or a decrease. This is in concordance with other studies examining the effect of environmental enrichment on variability of data. Some studies found that variability of some parameters can be increased under enriched housing conditions [25,28] or rather decreased [58]. Others found no effects of housing on variability [11,66,67] or inconsistent results with effects on variability dependent on sex and studied parameters [26]. But the mentioned studies were single-cohort studies, which might account for inconsistency of results between studies. The only other multicohort study studied female mice only and found that within-group variability of several behavioral parameters was not affected by enriched housing [30].

Knowledge of possible influence of factors on variability is crucial to estimate whether reproducibility might be jeopardized. Concerns have been expressed that individual mice might interact differently with enrichment, which might lead to an increased variability of physiological parameters and therefore to higher animal numbers needed to obtain the appropriate statistical power in statistical evaluation [25,26]. If indeed enrichment led to higher variability, experimental results achieved with the same number of animals would not be reproducible after changing the housing conditions. This would yield an ethical conflict between reduction and refinement because enrichment is applied to enhance animal welfare (refinement), while higher animal numbers would represent a contrast to the principle of reduction. However, our study gave no clear indication that simple forms of environmental enrichment increase the variability of a broad range of physiological parameters. Moreover, it must be stressed that absolute replicability of results cannot be achieved because the environmental conditions cannot be fully reproduced despite standardization of environmental factors. However, relevant differences for hypothesis-driven comparisons should be reproducible over the small noise induced by differing environmental factors to be of external validity. As our study shows, simple environmental enrichment according to Directive 2010/63/EU adds only little to the noise between cohorts.

Broadly and comprehensively speaking, our data argue that simple environmental enrichment does not greatly vary relevant specific parameters of biological and medical enquiry. We conclude that nesting material and shelters can thus be liberally applied to improve laboratory animal welfare without skewing results, and new data from these conditions can be compared to past data that were collected in barren housing.

## Material and methods

### Ethics statement

All animal experiments were performed in compliance with German animal welfare law and were approved by the institutional animal care and use committee (“Committee for animal experiments and animal facility” of the Helmholtz Zentrum München) and by the District Government of Upper Bavaria (approval number: 55.2-1-54-2532-199-13).

### Subjects and housing

A total number of 360 C57BL/6NTac (B6; Taconic, Denmark) and DBA/2NCrl (D2; Charles River, Germany) mice of both sexes were used in our study. Sixty mice (30 male, 30 female) of each strain were used in 1 set of examinations (see Table 1 for a full visual representation of the breakdown of the study design). This set of examinations was repeated twice so that 3 independent cohorts of 60 mice per strain were examined on the whole within a total timespan of 13 mo. Upon arrival, at 3 wk of age, animals were weighed, ear tagged, and split into 3 different groups (*n* = 10) following a stratified randomization scheme so that all groups had a similar body weight distribution at the beginning. All mice were housed in same-sex groups of 5, in type II polycarbonate cages in individually ventilated caging (IVC) systems (Tecniplast Greenline GM 500) with bedding (wood shavings, Altromin) and water and food ad libitum (standardized mouse diet, 1314, Altromin). The enrichment groups additionally had either a nestlet (PLEXX, Article ref. 14010)—which are cotton pads that mice can shred and use as nesting material (group “nest”)—or a nestlet plus an orange plastic mouse igloo (PLEXX, Article ref. 13100) as a shelter (group “double”), whereas the “control” group had no enrichment items at all (pictures of the 3 housing conditions are shown in S1 Fig). Nestlets are commonly used in laboratory animal facilities and can additionally be applied to evaluate mouse welfare by nest complexity scoring as needed [68,69]. Cages were cleaned, and enrichment items were renewed weekly. On those occasions, mice were also weighed and examined to evaluate their health. The animal room had a controlled 12/12–h light/dark cycle (lights on at 6:00 AM), temperature (22 ± 2°C), and relative humidity (45%–65%).

**Table 1:** Animal numbers used for this study.

Strain	B6						D2					
Experimental group	Control		Nest		Double		Control		Nest		Double	
Sex	M	F	M	F	M	F	M	F	M	F	M	F
Cohort 1	10	10	10	10	10	10	10	10	10	10	10	10
Cohort 2	10	10	10	10	10	10	10	10	10	10	10	10
Cohort 3	10	10	10	10	10	10	10	10	10	10	10	10

Altogether, 360 mice of 2 strains (C57BL/6NTac [B6], Taconic, Denmark; DBA/2NCrl [D2], Charles River, Germany) and both sexes were assigned to 3 experimental groups of different housing conditions (control, nest, double) according to the Table. One set of examinations is represented by each of the 3 cohorts (cohort 1, cohort 2, and cohort 3). Each cohort consisted of 12 groups with *n* = 10 animals per group. Due to technical reasons or death of animals during the course of the study, the numbers were reduced in some single groups or screens (see S2 Text). F, female; M, male.

### General study design

At 3 wk of age, mice were imported into the animal facility and randomly assigned to the experimental groups (“control,” “nest,” “double”). From wk 9 to 21, mice were examined following the workflow of the primary screen for phenotypic analysis with minor adaptions (Fig 1).

### Phenotype screens

The phenotyping screens were performed at the German Mouse Clinic, which offers a large-scale standardized and comprehensive phenotypic analysis of mice. In this study, mice were examined in the fields of behavior, dysmorphology, neurology, clinical chemistry and hematology, eye, allergy, energy metabolism, pain perception, cardiovascular health, and pathology (Fig 1). The phenotyping screens followed standardized examination protocols, as previously described (www.mouseclinic.de) [46–48]. In general, *n* = 10 mice per group (see Table 1) were examined, except for the eye screen (*n* = 7), cardiovascular screen (*n* = 7 in the first cohort of B6 mice, only for ECHO), and pathology screen (*n* = 4–5 for macroscopical analysis; *n* = 1–2 for histological analysis). Due to a few cases of unexpected death, animal numbers of groups were in some instances reduced to *n* = 9 and once to *n* = 8 (for details, see S2 Text).

### Behavior

#### Open field

The test apparatus (ActiMot, TSE) was a transparent and infrared light-permeable acrylic test arena (45.5 × 45.5 × 39.5 cm internal measurements) surrounded by a square-shaped frame with 2 pairs of light-beam strips. Lux levels were set at approximately 150 in the corners and 200 in the center of the arena. After at least 30 min of acclimatization to the test room, mice were carefully picked up by the base of the tail and were gently placed into the test arena to freely explore it for 20 min. After each trial, the arena was cleaned with a disinfectant (Pursept-A Xpress, Merz), which was used for the procedure in all cohorts. The following parameters were digitally measured: distance traveled; resting and permanence time; and speed of movement for the whole arena, the periphery, and the center as well as number of rearings. Rearing frequency, percentage distance traveled, and percentage time spent in the center, as well as the latency to first entry in the center and center entry frequency, were calculated.

#### Acoustic startle response and pre-pulse inhibition

Acoustic startle response (ASR) and pre-pulse inhibition (PPI) were assessed using a startle apparatus (Med Associates, St Albans City, VT). The protocol for PPI was based on the IMPReSS protocol from the International Mouse Phenotyping consortium (see https://www.mousephenotype.org/impress/protocol/176/7), adapted to the specifications of our startle equipment. Background noise was 65 dB, and startle pulses were bursts of white noise (40 msec). A session was initiated with a 5-min acclimation period followed by 5 presentations of leader startle pulses (110 dB) that were excluded from statistical analysis. Trial types for the PPI included 4 different pre-pulse intensities (67, 69, 73, 81 dB); each pre-pulse preceded the startle pulse (110 dB) by a 50-msec interstimulus interval. Each trial type was presented 10 times in random order, organized in 10 blocks, each trial type occurring once per block. Intertrial intervals varied from 20 to 30 s.

### Neurology

#### Modified SHIRPA

Twenty-three parameters were examined to test for basic neurological function and general health by using a modification of the SHIRPA protocol [70]. The details of the procedure can be found elsewhere [71].

#### Grip strength

For measurement of muscle strength, a grip strength meter system was used (Bioseb, Chaville, France). Mice were lowered onto a metal grid that they were allowed to grasp with either 2 or 4 paws. They were then pulled away slowly, and the maximal force until they release the grid was measured by a force meter. Each mouse was measured 3 times within 1 min, and mean values were used as representative values for one individual mouse.

#### Rotarod

A rotarod (Bioseb, Chaville, France) was used to assess motor coordination, balance, and motor learning ability [72]. Mice were placed on the rotarod at an accelerating speed from 4 to 40 rpm for 5 min for 3 measurements, with 15 min between each trial, and latencies were recorded. Measurements were terminated when the mouse either fell off the rod, showed passive cycling, or after a maximum time of 5 min.

#### ABR

Measurement of the ABR was used to objectively evaluate hearing sensitivity of mice in anesthetized animals (i.p., 137 mg ketamine/6,6 mg xylazine/ kg body weight). Two electrodes were attached subcutaneously between mastoid and vertex to detect the physiological hearing curves produced by signal propagation in the brain stem following acoustic stimulation of different frequencies (6, 12, 18, 24, 30 Hz) in an ABR workstation (Tucker-Davis Technologies, Alachua, FL). Auditory threshold was determined as the minimum sound pressure level at the respective frequency that still triggered a typical ABR curve.

### Nociception

#### Hot plate

The hot plate test was conducted as described recently [48]. In short, mice were placed on a metal plate that was maintained at 52 ± 2°C, and type of the first 2 reactions and respective latencies were recorded for assessment of thermal sensitivity indicative for nociceptive pathway integrity. Mice were taken off the plate as soon as they showed 2 signs or after maximum time of 30 s to avoid tissue lesion.

### Dysmorphology

#### Anatomical observation

The anatomical observation is a basic examination to screen for any morphological abnormalities and was performed as described previously [73].

#### DEXA

DEXA analysis was executed during the same anesthesia as ABR following a standardized protocol [48]. For bone density measurement, a pDEXA Sabre X-ray Bone Densitometer (Norland Medical Systems, Basingstoke, Hampshire, UK) was used with the following settings: scan speed 20 mm/s, resolution 0.5 mm × 1.0 mm, histogram average width setting 0.020 g/cm^2^.

For technical reasons, 1 cohort of D2 mice could not be measured with DEXA analysis and is therefore not included in linear model analysis. Data will be made accessible if requested.

### Energy metabolism

#### Indirect calorimetry

For assessment of energy metabolism, gas exchange (O_2_ consumption, CO_2_ production) was monitored for individual mice in an open-circuit respirometry system for 21 h (13:00 CET until 10:00 CET the next morning) with free access to food and water. Mice had no enrichment materials during this time. Respiratory exchange ratio could then be calculated. Additionally, locomotor activity and cumulative food intake were also recorded.

#### qNMR

Body composition was measured with qNMR (MiniSpec LF50, Bruker Optics, Ettlingen, Germany). Body mass, lean tissue, and body fat were thereby assessed in live and awake mice. Both indirect calorimetry as well as qNMR were conducted as described earlier [48].

### Clinical chemistry, hematology and allergy assessment

#### Intraperitoneal glucose tolerance test

Intraperitoneal glucose tolerance test (IpGTT) was performed after 16 to 18 h of fasting. After initial assessment of body weight, basal glucose levels (after fasting) were determined by cutting off a maximum of 1 mm of the tail tip to receive a drop of blood. Glucose levels were analyzed with an Accu-Chek Aviva glucose analyzer (Roche/Mannheim). Then, mice were injected intraperitoneally with 2 g of glucose per kg body weight using a 20% glucose solution, a 25-gauge needle, and a 1-ml syringe. At 15, 30, 60, and 120 min after glucose injection, additional blood samples (1 drop each) were collected and used to determine blood glucose levels as described above. Repeated bleeding was induced by removing the clot from the first incision and massaging the tail of the mouse. For the analysis, the areas under the curve above basal glucose level for the first 30 min (AUC 0–30) and for the remaining 90 min (AUC 30–120) were calculated as described previously [74]. For technical reasons, parameters glucose level fasted, AUC 0–30, and AUC 30–120 had to be excluded for B6 mice and so were not included in the linear model analysis.

#### Blood sampling

Blood samples were taken from isoflurane-anesthetized mice by puncturing the retrobulbar sinus with nonheparinized glass capillaries (1.0 mm in diameter; Neolab; Munich, Germany). After collecting the samples, mice were euthanized during narcosis by cervical dislocation or with CO_2_. Blood samples were divided into 2 portions. The major portion was collected in a heparinized tube (Li-heparin, KABE; Numbrecht, Germany; Art.No. 078028). Cells and plasma were separated by a centrifugation step (10 min, 5,000 × g; 8°C, Biofuge Fresco, Heraeus; Hanau, Germany). Plasma was used for measurement of immunoglobulin concentration and clinical chemistry assessment. The smaller portion was collected in an EDTA-coated tube (KABE, Art.No 078035) for hematological investigations.

#### Clinical chemistry, hematology, allergy assessment

Detailed information for measurements of clinical chemistry, hematology, and allergy assessment were described recently [48,75].

### Cardiovascular health

#### Echocardiography

Left ventricular function was evaluated on nonanesthetized conscious mice by transthoracic echocardiography using a Vevo 2100 Imaging System (Visual Sonics) with a 30 MHz probe. Left ventricular parasternal short- and long-axis views were obtained in B-mode imaging, and left ventricular parasternal short-axis views were obtained in M-mode imaging at the papillary-muscle level. The short-axis M-mode images were used to measure left ventricular end-diastolic internal diameter (LVIDd), left ventricular end-systolic internal diameter (LVIDs), diastolic and systolic septal wall thickness (IVS), and left ventricular diastolic and systolic posterior wall (LVPW) thickness in 3 consecutive beats according to the American Society of Echocardiography leading edge method [76]. Some additional parameters such as fractional shortening, corrected left ventricular mass, stroke volume, and heart and respiratory rate were calculated from the above measured parameters [77].

For the first cohort of B6 mice, only n = 7 animals were examined.

#### Electrocardiography

ECGs were recorded in conscious mice with the ECGenie (Mouse Specifics, Boston, MA) and analyzed using e-Mouse software (Mouse Specifics, Boston, MA) The cardiac electrical activity was detected noninvasively through the animals’ paws. The size and arrangement of the electrodes were configured to advance contact with 3 of the animals’ paws to provide an ECG signal that is equivalent to Einthoven lead II. For each animal, intervals and amplitudes were evaluated from continuous recordings of at least 15 ECG signals. e-MOUSE software uses peak detection to calculate the heart rate (HR). HR variability (HRV) was calculated as the mean of the differences between sequential HRs. The software plots its interpretation of P, Q, R, S, and T for each beat so that unfiltered noise or motion artifacts are rejected. This is followed by calculations of the mean of the ECG time intervals for each set of waveforms. The corrected QT interval (QTc) was calculated by dividing the QT interval by the square root of the preceding RR interval. QT dispersion was measured as interlead variability of QT intervals. The QTc dispersion was calculated as the rate corrected–QT dispersion.

In the group female, double, B6 of the first cohort, 3 animals had to be excluded due to technical reasons.

### Eye

#### Scheimpflug imaging

Images of corneas and lenses were taken with a Pentacam digital camera system (Oculus GmbH, Wetzlar, Germany). Pupils were widened by a drop of 0.5% atropine. Then, mice were held on a platform such that the vertical light slit (light source: LEDs, 475 nm) was orientated in the middle of the eyeball. Distance between eye and camera was fine-adjusted with the help of the provided software in order to guarantee optimal focus. Subsequently, the measurements were started manually. Mean density across the lens was quantified with the provided densitometry tool (Oculus GmbH, Wetzlar, Germany). Additionally, we conducted a qualitative examination of lens and cornea.

#### Virtual drum test

Vision tests were performed with the OptoMotry virtual optokinetic drum system (Cerebral Mechanics, Lethbridge, Canada) as described previously [78]. Briefly, a cylinder composed of a sine wave grating—drawn in 3D coordinate space on 4 computer monitors facing to form a square—rotates around the animal, which is placed on a platform in the middle of the drum. Visually unimpaired mice track the grating with reflexive head and neck movements (head tracking). Vision threshold of the tested mice was quantified by a randomized simple staircase test. Rotation speed and contrast were set to 12.0 d/s and 100%, respectively.

For the eye screen, only n = 7 animals were examined.

### Pathology

#### Pathological examination

Following blood sampling and euthanasia, mice underwent macroscopical pathological examination (http://eulep.pdn.cam.ac.uk/Necropsy_of_the_Mouse/index.php). After complete dissection, all organs were fixed in 4% buffered formalin and embedded in paraffin for histological examination. Four-μm–thick sections from skin, heart, muscle, lung, cerebrum, cerebellum, thymus, spleen, cervical lymph nodes, trachea, thyroid and parathyroid glands, adrenal glands, esophagus, stomach, intestine, liver, gall bladder, pancreas, kidneys, reproductive organs, and urinary bladder were cut and stained with hematoxylin–eosin (HE).

For macroscopical pathological examination, only n = 4–5 animals were examined; for histological examination, n = 1–2.

### Statistical analysis

Our study aimed at answering the following questions: firstly, whether the mean of each parameter is influenced by enrichment and secondly, whether enrichment has an influence on the variability of the measurements. We considered sex and cohort as additional predictors for the possible alteration of mean and variability of the measurements.

The influence of enrichment on the mean values of the measured parameters was evaluated with linear models defined by

$$y=\beta_{\mathrm{Intercept}}+\beta_{\mathrm{enrichment}\;\mathrm{nest}}+\beta_{\mathrm{enrichment}\;\mathrm{double}}+\beta_{\mathrm{sex}\;\mathrm{male}}+\beta_{\mathrm{cohort}\;2}+\beta_{\mathrm{cohort}\;3}+all\;double\;interactions+\varepsilon$$

A variable selection using the BIC was performed. The heatmap (Fig 2) shows the color-coded estimators (β) of the respective model in percent of the intercept of the model to provide a value which is comparable between the parameters. Nonselected influencing factors are marked in grey.

To evaluate the impact of enrichment on the variability, bootstrapped [79] samples of the original data were used. Therefore, 1,000 samples were drawn of size n_j_ from each group j (enrichment × sex × cohort combination). As a comparable measure for variability, the CV was used.

Linear models using the bootstrapped CV as outcome, and enrichment, sex, and cohort as influencing factors, were fitted. Because the interactions effects did not appear often as relevant in the linear models for the analysis of the mean values, only main effects have been included in the models for CV.

To summarize the results of the bootstrap method, empirical 95% confidence intervals for the estimators of all influencing variables of the linear models were computed. The results are summarized in 3 categories and are displayed in Fig 5, as follows: confidence interval includes 0 (grey; thus no effect is assumed), confidence interval is greater than 0 (violet), or confidence interval is below 0 (yellow).

All analyses were performed separately for each measured parameter and mouse strain. The fact that some of the parameters are highly correlated needs to be taken into consideration when interpreting the results.

Measures for significance were omitted due to the fact that the main purpose of the study was to get an overview about direction and magnitude of possible enrichment effects on parameters of different research fields. Providing such a broad summary is helpful and can be used by other researchers to focus on single parameters of interest in confirmative studies.

The statistic software R (version 3.0.2, R Foundation for Statistical Computing, Vienna, Austria) was used for all analyses and graphs. Dummy coding was used for all categorical factors in the linear models. Reference categories were as follows: enrichment: none; sex: female; and the cohort: 1.

## Supporting information

S1 Data Raw data of measured parameters (all screens). (ZIP)

S1 Fig Raw data of measured parameters (all screens). Raw data are shown as whisker-plots with the box representing 25th percentile, median, and 75th percentile. The length of whiskers is maximally the 1.5-fold interquartile range but is determined by the last value within this range. All individual values and each subgroup are shown. All individual values are shown for each experimental group (con, nest, double) and every cohort (1, 2, 3) for female and male mice in the upper and lower plot, respectively, for each of the 164 quantitative parameters.
(PDF)

S2 Fig Housing conditions of the 3 experimental groups. (A) “Control” group without any items; (B) group “nest” with 1 cotton nestlet (PLEXX, Article ref. 14010); (C) group “double” with 1 cotton nestlet plus 1 plastic mouse igloo (PLEXX, Article ref. 13100).
(PNG)

S1 Table Results of SHIRPA (neurology screen). Results are presented as number of animals receiving the parameter-specific score in combined cohorts and are described as follows: (x/y/z) with x = number of animals with score “0”; y = number of animals with score “1”; z = number of animals with score “2.” Specifications for scores: body position: 0 = inactive; 1 = active; 2 = excessively active; tremor: 0 = absent; 1 = present; defecation: 0 = present; 1 = absent; transfer arousal: 0 = prolonged freeze; 1 = brief freeze; 2 = immediate movement; gait: 0 = fluid; 1 = abnormal; tail elevation: 0 = dragging; 1 = horizontal; 2 = elevated; startle response: 0 = no reaction; 1 = Preyer reflex; 2 = jumping; touch escape: 0 = no response; 1 = response to touch; 2 = flees prior to touch; trunk curl: 0 = absent; 1 = present; limb grasping: 0 = absent; 1 = present; pinna reflex: 0 = present; 1 = absent; urination: 0 = present; 1 = absent; contact righting reflex: 0 = present; 1 = absent; evidence of biting: 0 = no aggression; 1 = aggressive; vocalization: 0 = no; 1 = yes. SHIRPA, Smithkline Beecham, MRC Harwell, Imperial College, the Royal London hospital Phenotype Assessment.
(PDF)

S2 Table Summary of background lesions in histopathological examination (pathology screen). Affected animals are shown as portion of total examined animals of combined cohorts.
(PDF)

S1 Text Results of morphological examination (dysmorphology screen). (PDF)

S2 Text Reduction of animal numbers in single groups or screens. (PDF)

## Abbreviations

ABR: auditory brain stem response
ASR: acoustic startle response
BIC: Bayesian Information Criterion
CV: coefficient of variation
DEXA: dual-energy x-ray absorptiometry
GMC: German Mouse Clinic
HE: hematoxylin–eosin
HR: heart rate
HRV: heart rate variability
IgE: immunoglobulin E
IpGTT: intraperitoneal glucose tolerance test
IVC: individually ventilated caging
LVIDd: left ventricular end-diastolic internal diameter
LVIDs: left ventricular end-systolic internal diameter
LVPW: left ventricular posterior wall
PPI: pre-pulse inhibition
qNMR: quantitative nuclear magnetic resonance
QTc: corrected QT
SHIRPA: Smithkline Beecham, MRC Harwell, Imperial College, the Royal London Hospital Phenotype Assessment
