# Supplementary material for: Laboratory mouse housing conditions can be improved using common environmental enrichment without compromising data
Source: PLoS Biol. 2018 Apr 16;16(4):e2005019. doi: 10.1371/journal.pbio.2005019 (PMC5922977; doi:10.1371/journal.pbio.2005019)
Supplement: S1 Fig — Raw data are shown as whisker-plots with the box representing 25th percentile, median, and 75th percentile. The length of whiskers is maximally the 1.5-fold interquartile range but is determined by the last value within this range. All individual values and each subgroup are shown. All individual values are shown for each experimental group (con, nest, double) and every cohort (1, 2, 3) for female and male mice in the upper and lower plot, respectively, for each of the 164 quantitative parameters. (PDF) [file pbio.2005019.s002.pdf]

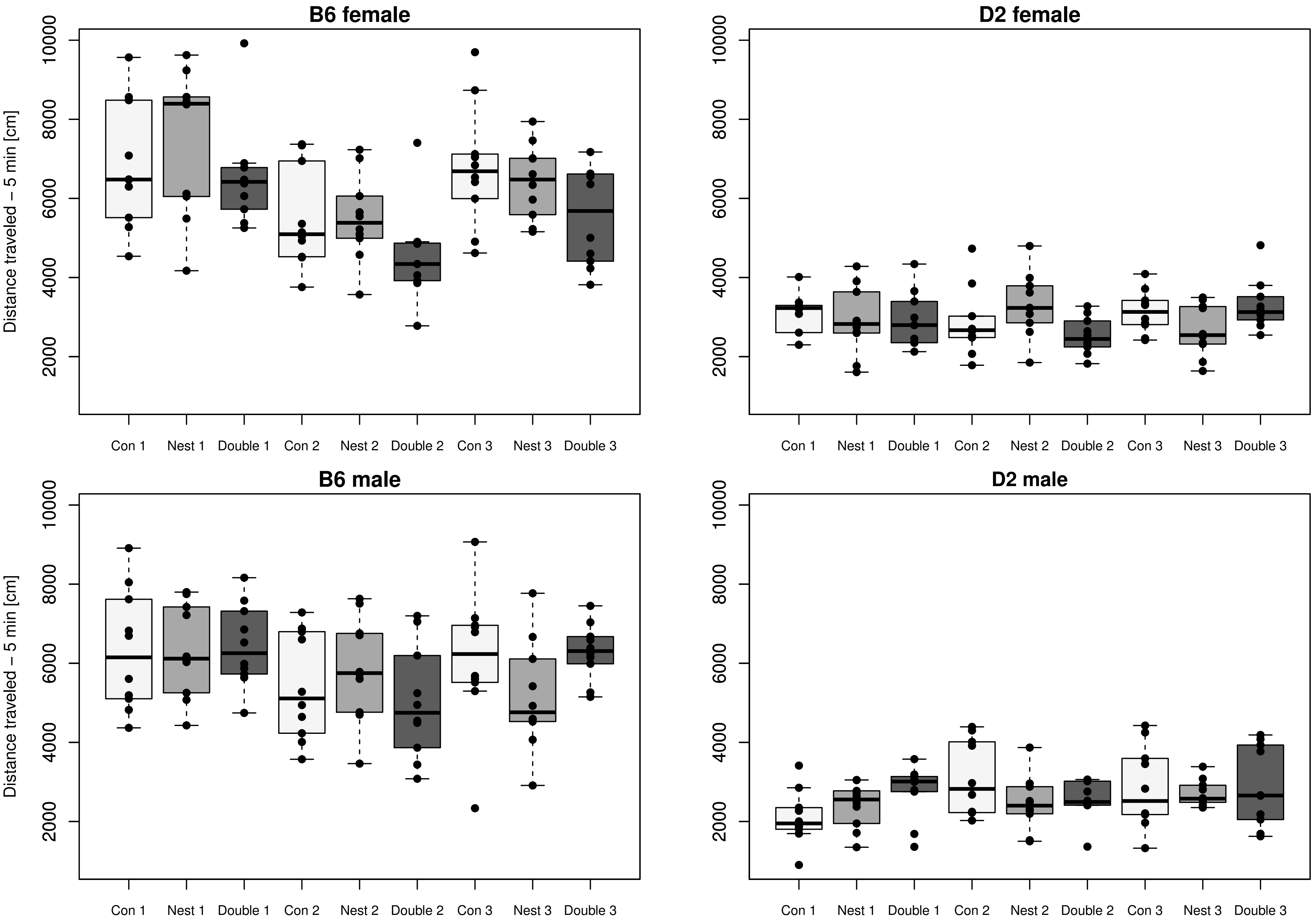

B6 female
D2 female
B6 male
D2 male
Distance traveled – 5 min [cm]
2000
4000
6000
8000
10000
Con 1
Nest 1
Double 1
Con 2
Nest 2
Double 2
Con 3
Nest 3
Double 3

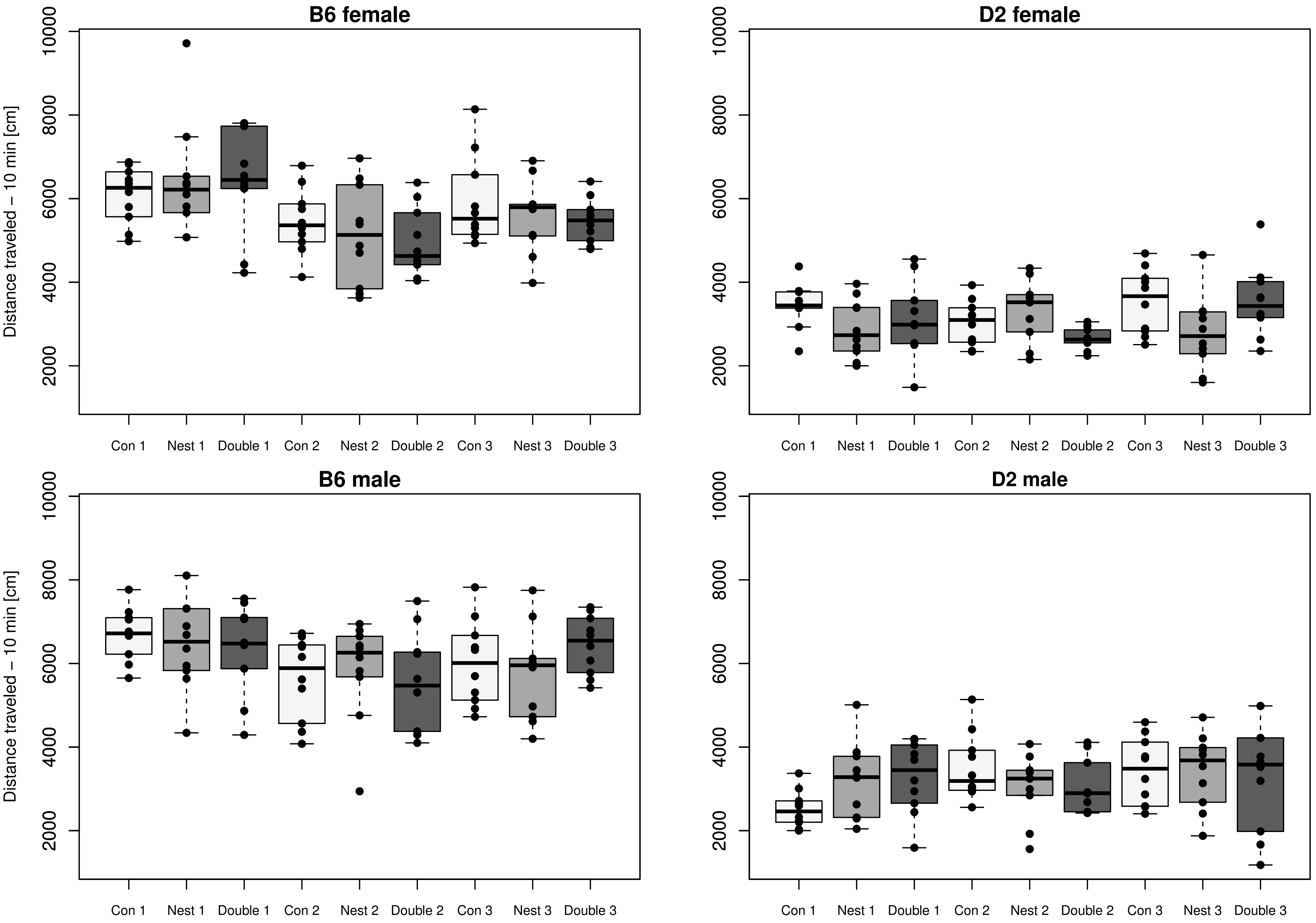
B6 female
D2 female
B6 male
D2 male
Distance traveled – 10 min [cm]
2000
4000
6000
8000
10000
Con 1
Nest 1
Double 1
Con 2
Nest 2
Double 2
Con 3
Nest 3
Double 3

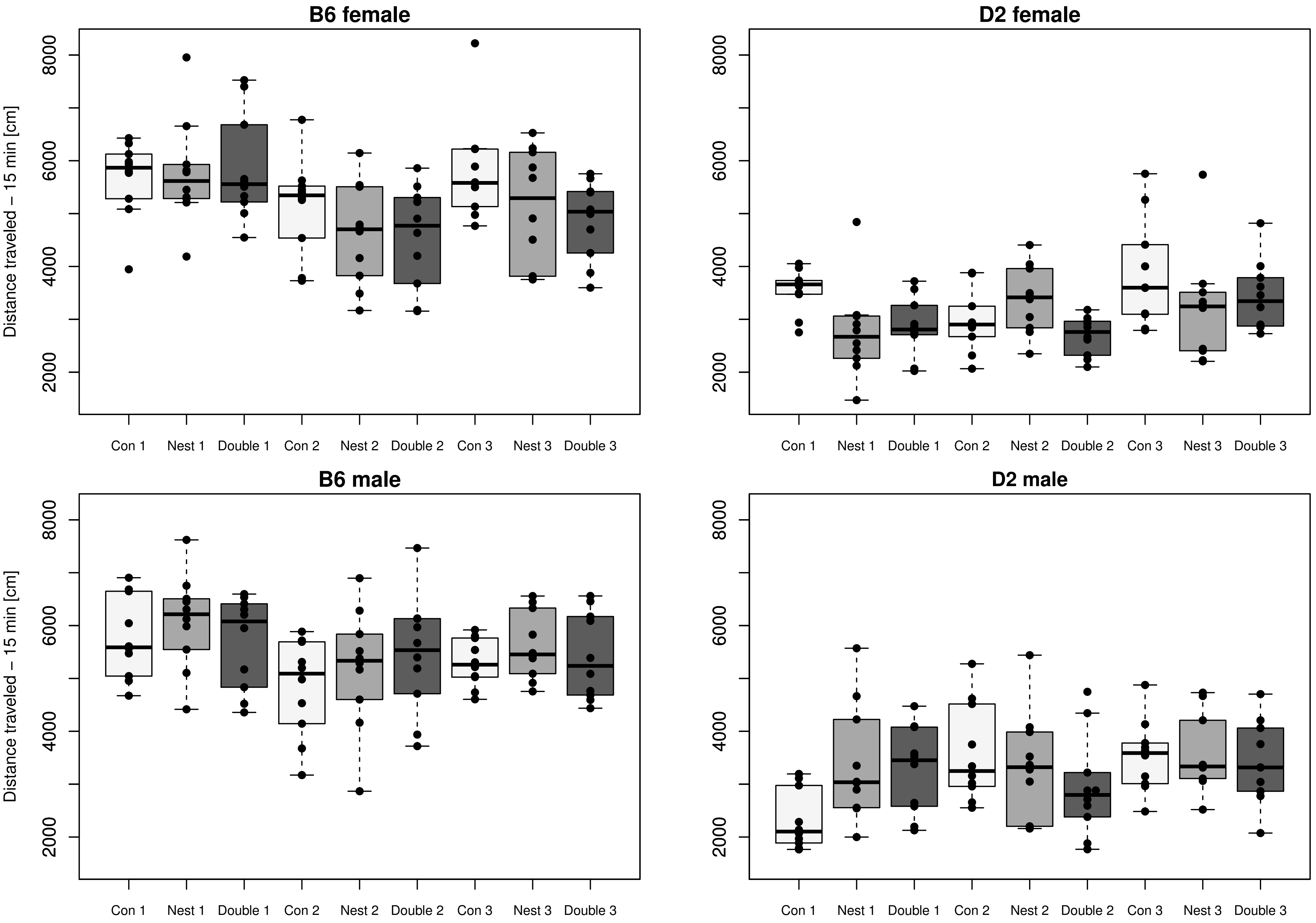

B6 female
D2 female
B6 male
D2 male
Distance traveled – 15 min [cm]
2000
4000
6000
8000
Con 1
Nest 1
Double 1
Con 2
Nest 2
Double 2
Con 3
Nest 3
Double 3

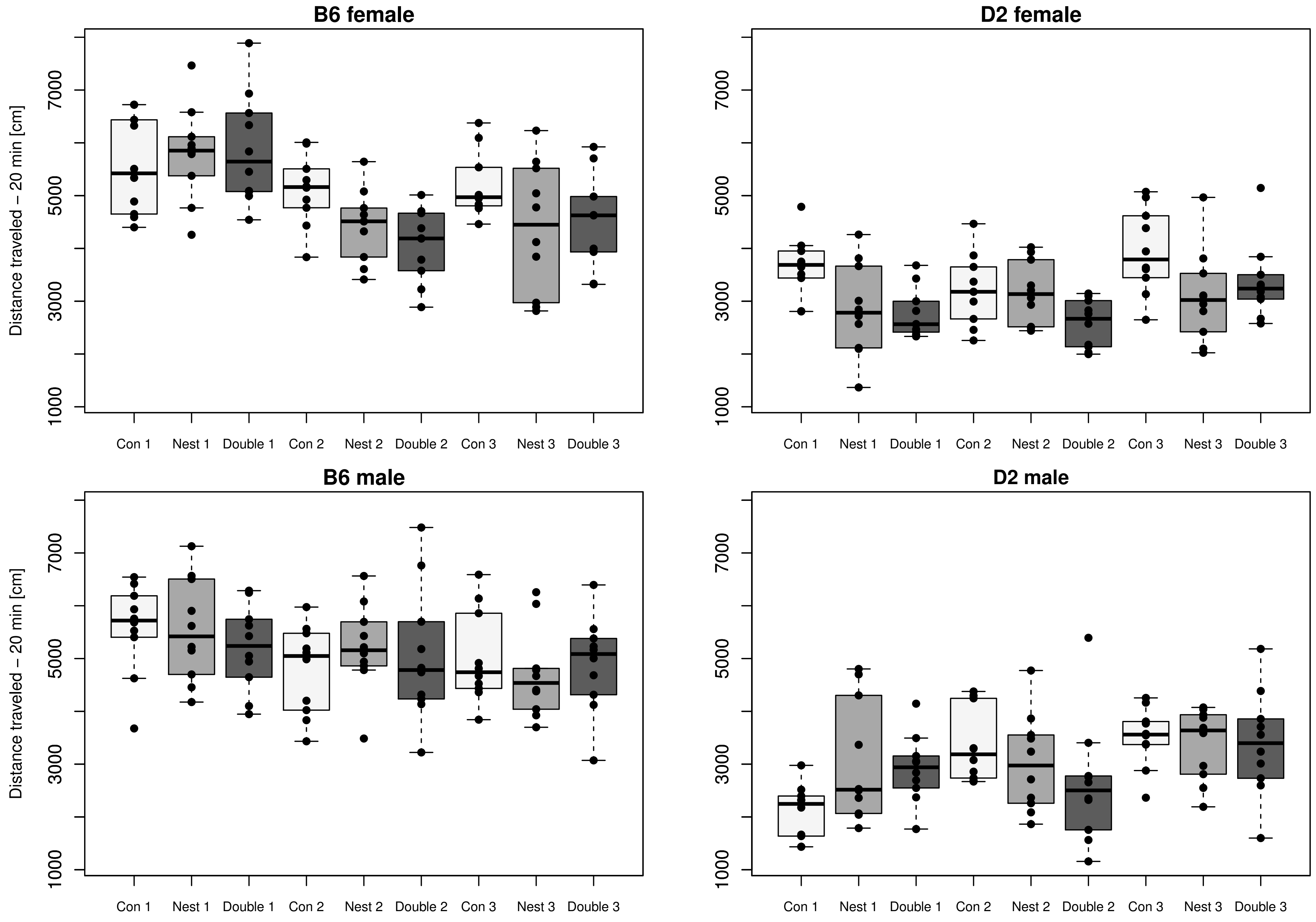
B6 female
D2 female
B6 male
D2 male
Distance traveled – 20 min [cm]
1000
3000
5000
7000
Con 1
Nest 1
Double 1
Con 2
Nest 2
Double 2
Con 3
Nest 3
Double 3

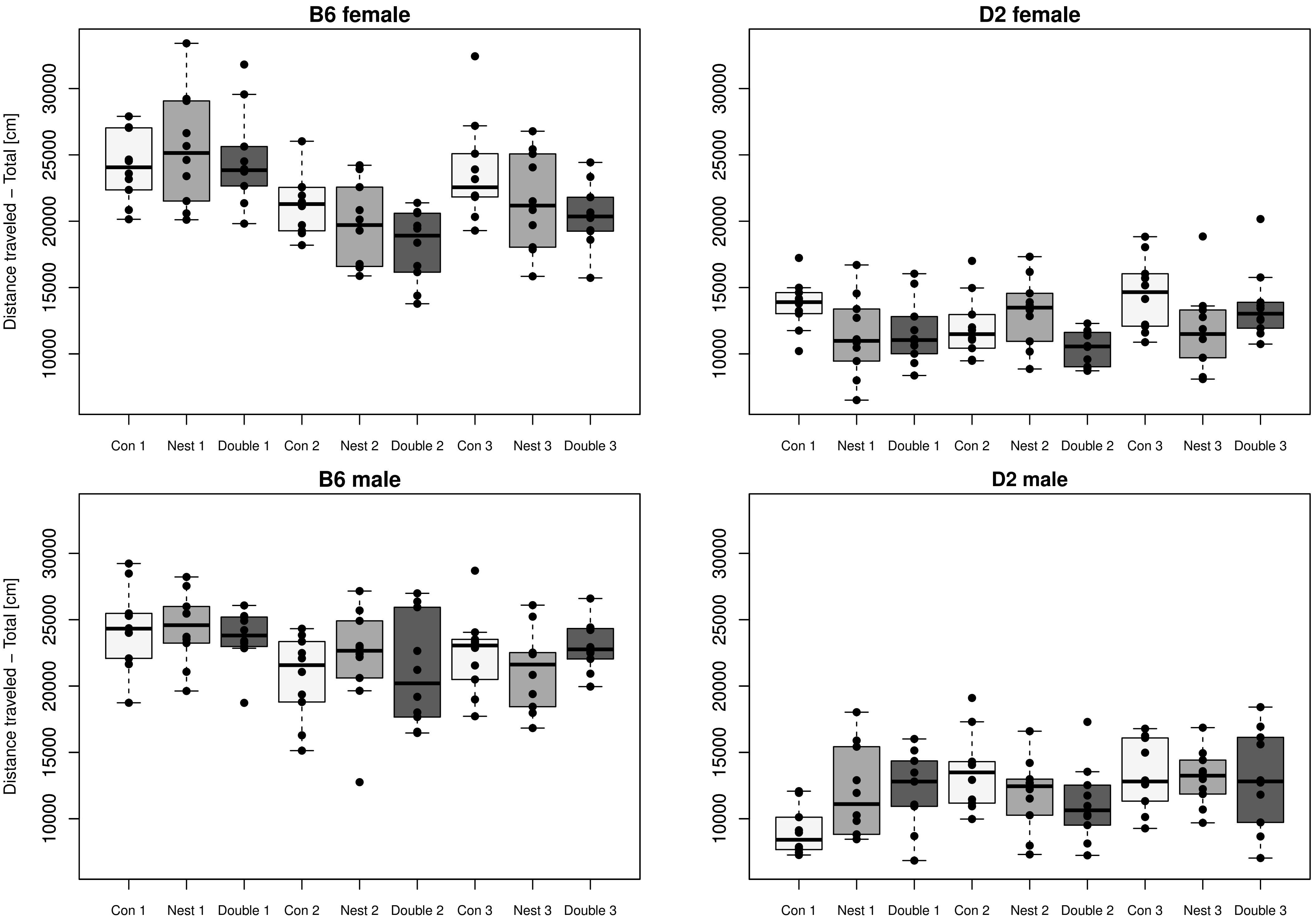
B6 female
D2 female
B6 male
D2 male
Distance traveled – Total [cm]
10000
15000
20000
25000
30000
Con 1
Nest 1
Double 1
Con 2
Nest 2
Double 2
Con 3
Nest 3
Double 3

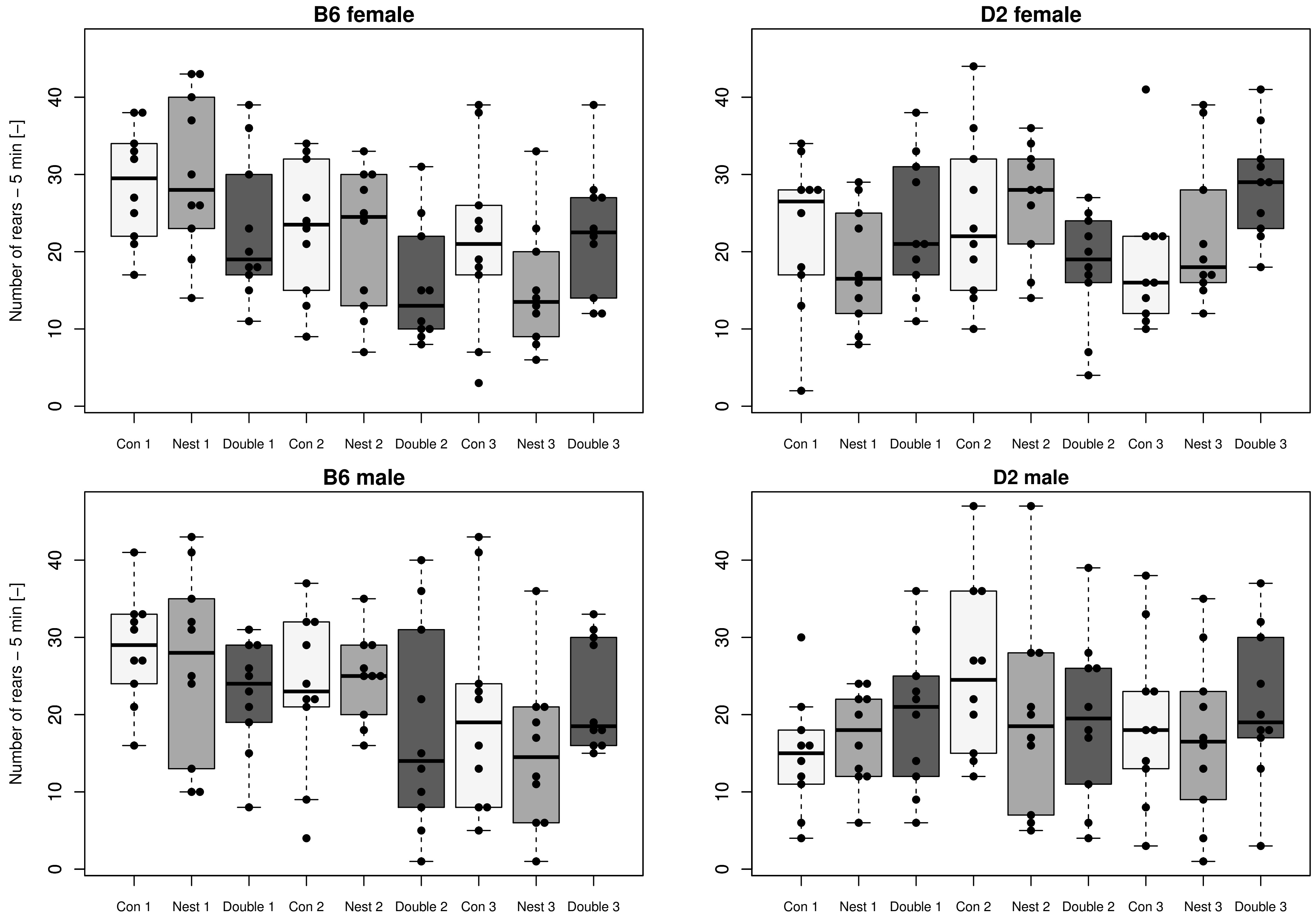

B6 female
D2 female
B6 male
D2 male
Number of rears – 5 min [–]
0
10
20
30
40
Con 1
Nest 1
Double 1
Con 2
Nest 2
Double 2
Con 3
Nest 3
Double 3

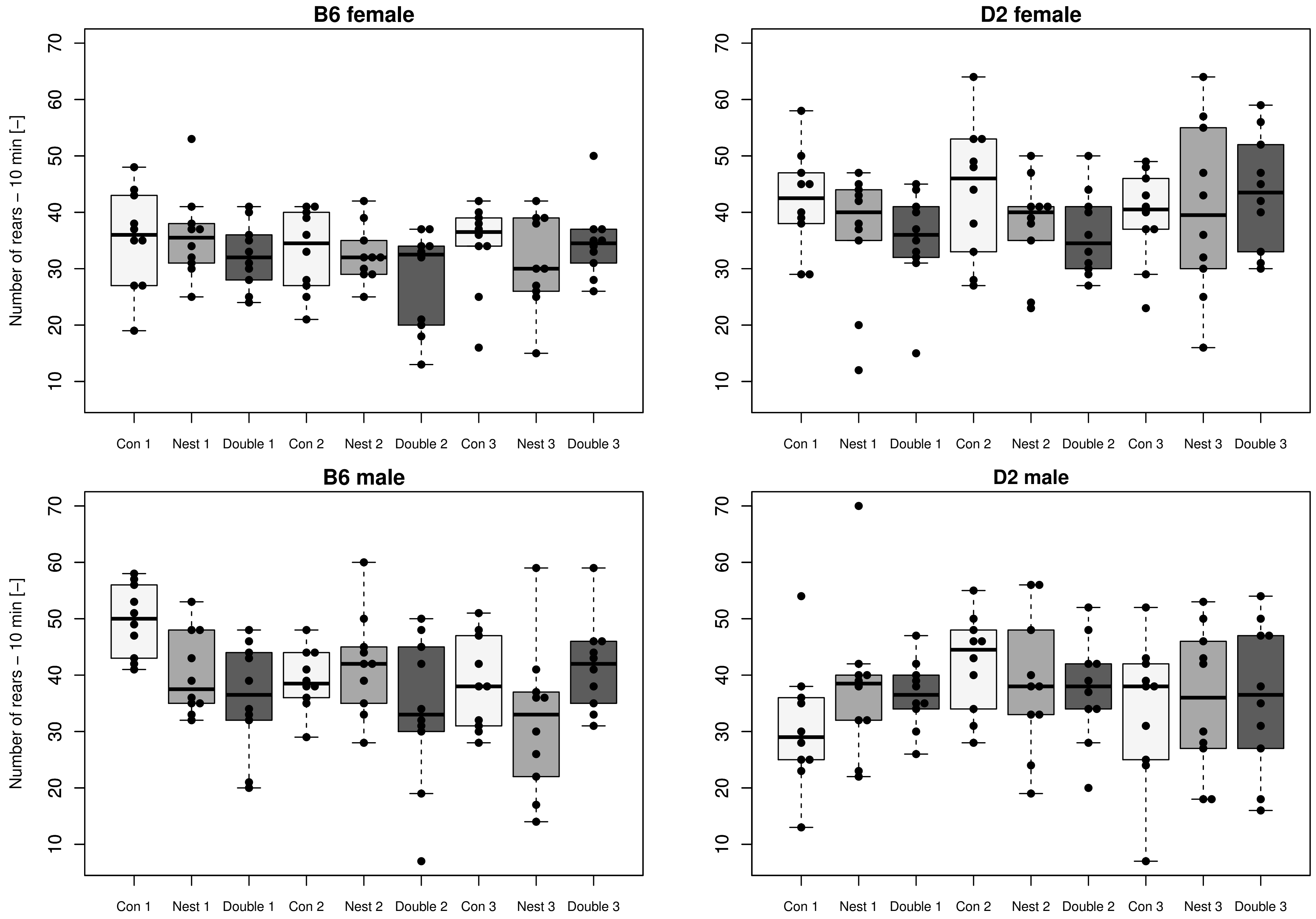
B6 female
D2 female
B6 male
D2 male
Number of rears – 10 min [–]
10
20
30
40
50
60
70
Con 1
Nest 1
Double 1
Con 2
Nest 2
Double 2
Con 3
Nest 3
Double 3

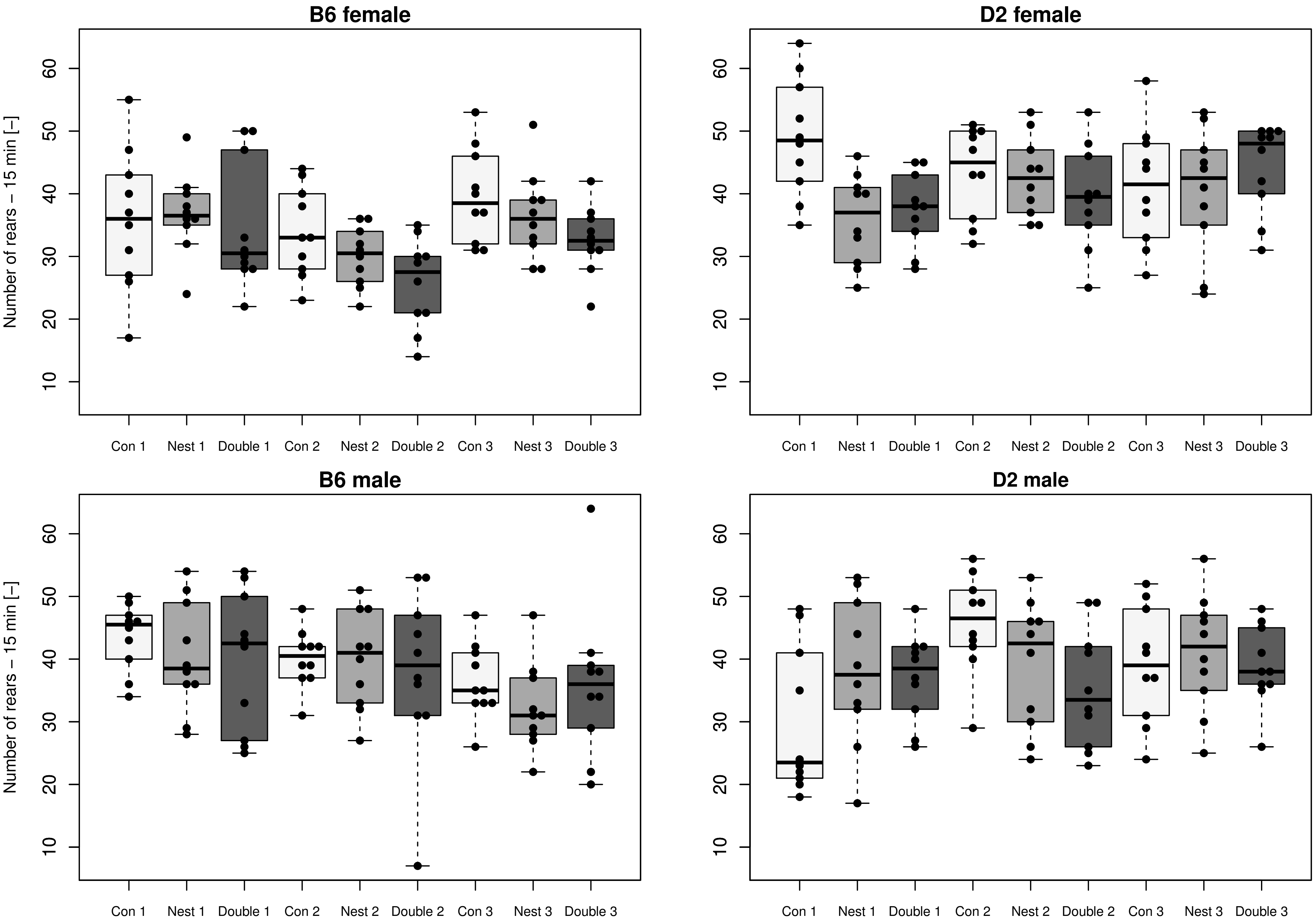
B6 female
D2 female
B6 male
D2 male
Number of rears – 15 min [–]
10
20
30
40
50
60
Con 1
Nest 1
Double 1
Con 2
Nest 2
Double 2
Con 3
Nest 3
Double 3

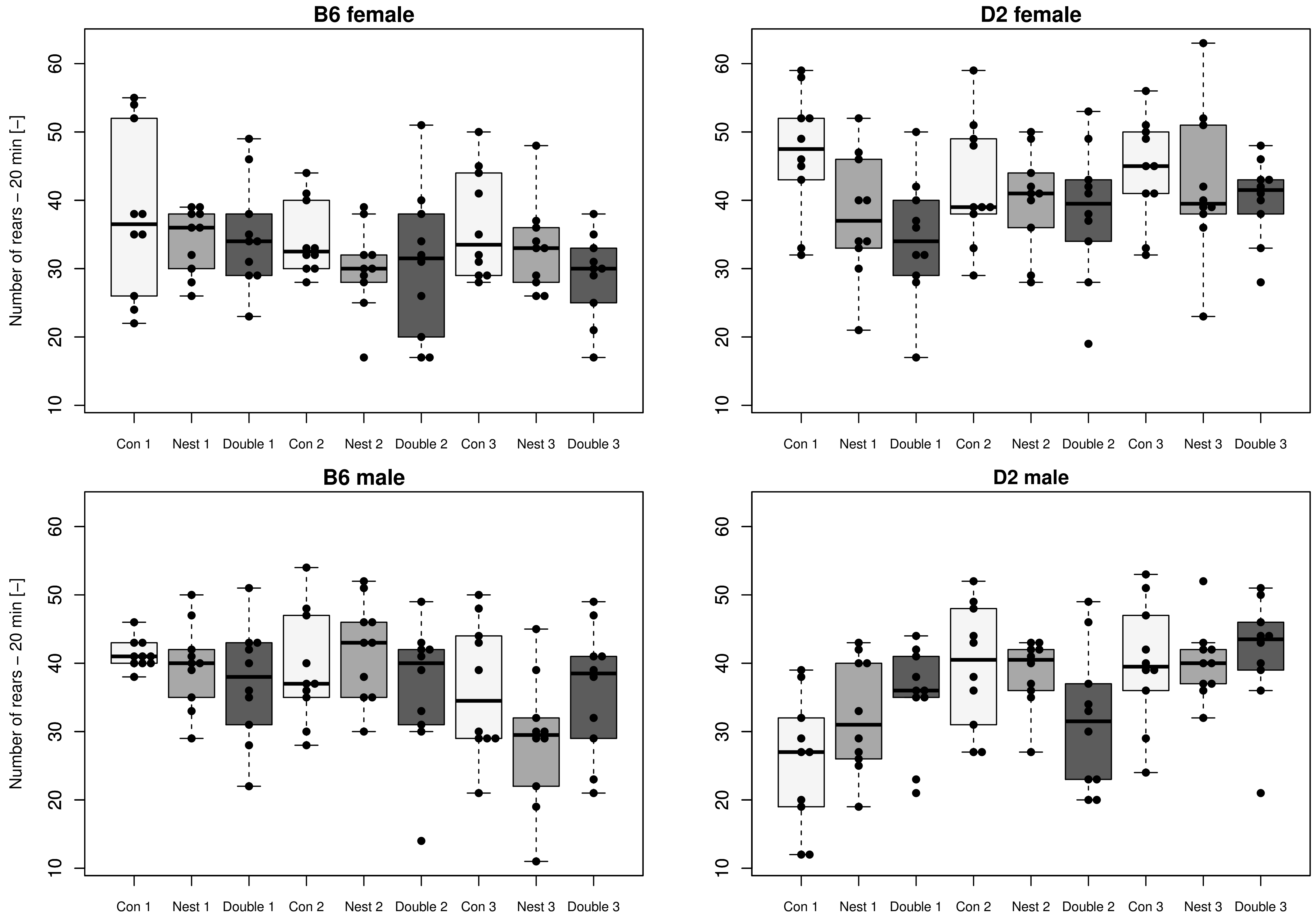
B6 female
D2 female
B6 male
D2 male
Number of rears – 20 min [–]
10
20
30
40
50
60
Con 1
Nest 1
Double 1
Con 2
Nest 2
Double 2
Con 3
Nest 3
Double 3

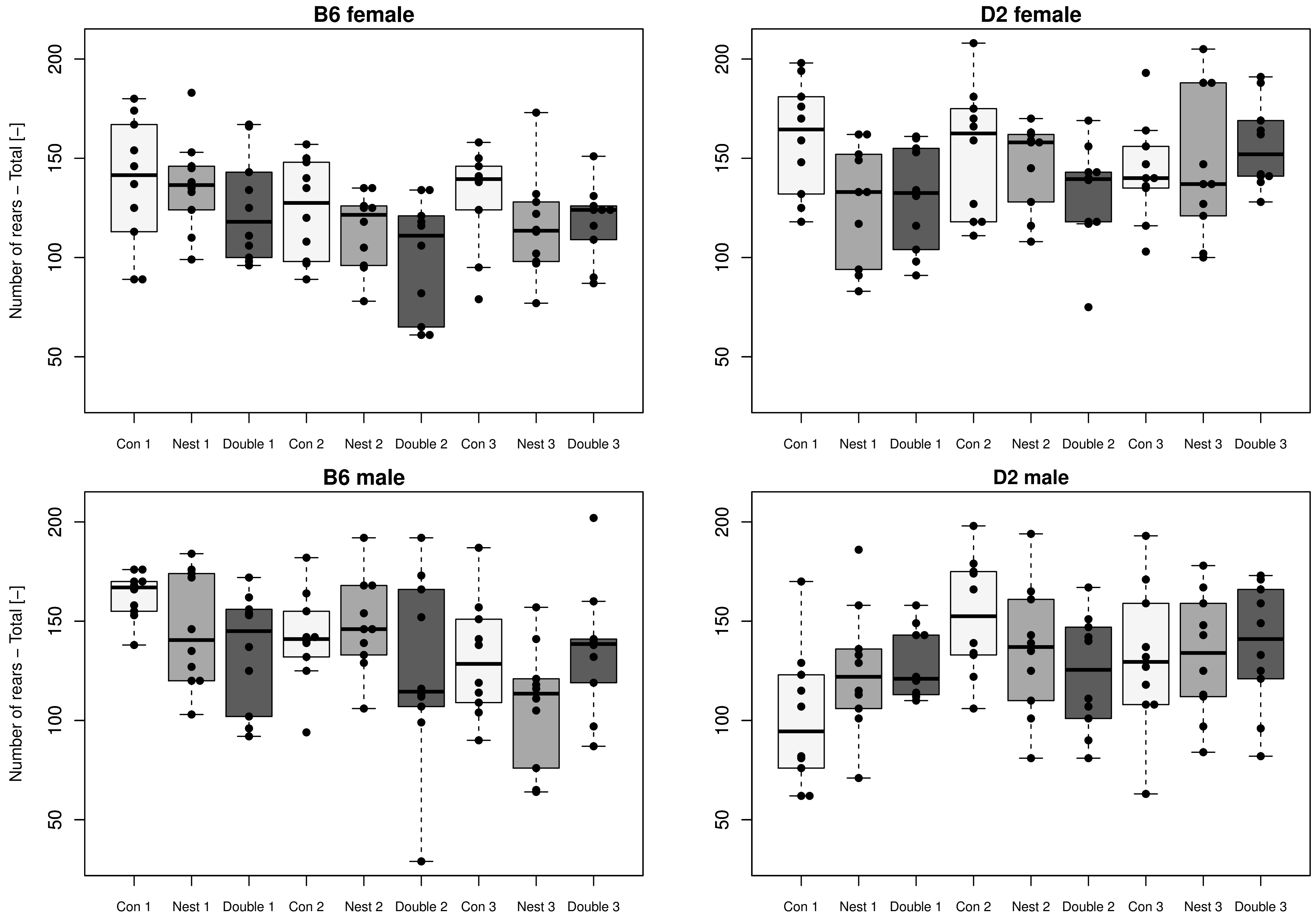
B6 female
D2 female
B6 male
D2 male
Number of rears – Total [–]
200
150
100
50
Con 1
Nest 1
Double 1
Con 2
Nest 2
Double 2
Con 3
Nest 3
Double 3

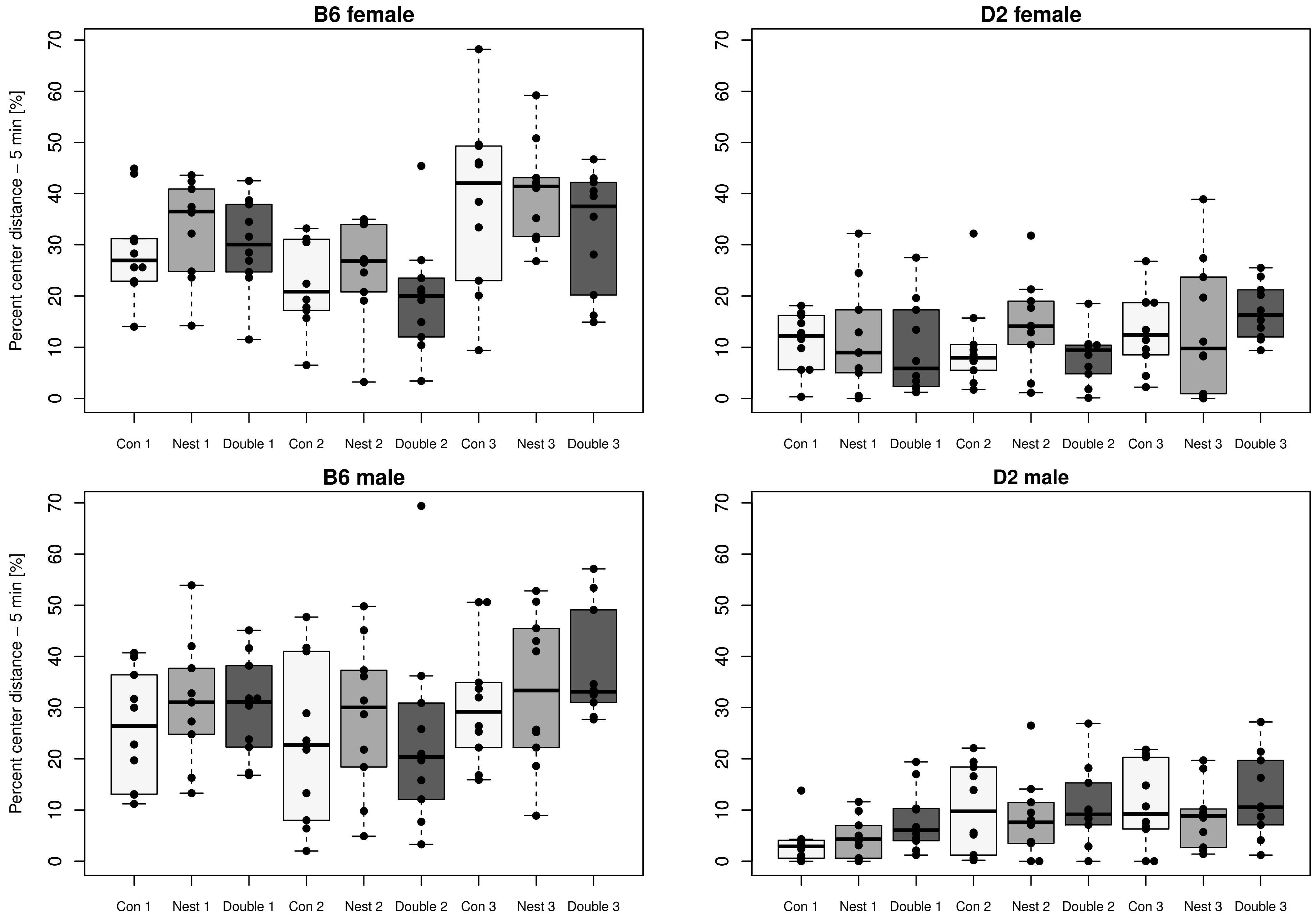
B6 female
D2 female
B6 male
D2 male
Percent center distance – 5 min [%]
0
10
20
30
40
50
60
70
Con 1
Nest 1
Double 1
Con 2
Nest 2
Double 2
Con 3
Nest 3
Double 3

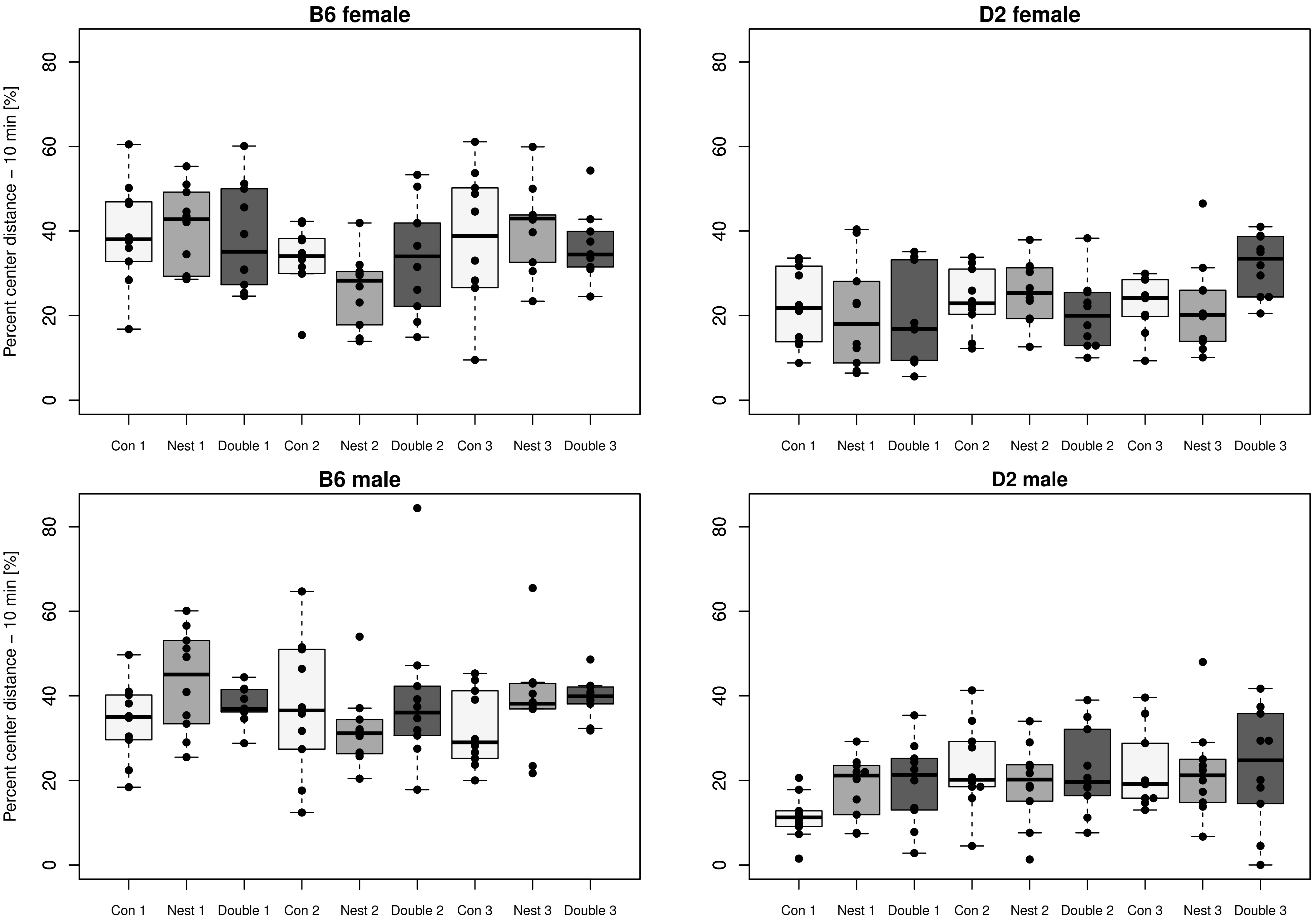
B6 female
D2 female
B6 male
D2 male
Percent center distance – 10 min [%]
0
20
40
60
80
Con 1
Nest 1
Double 1
Con 2
Nest 2
Double 2
Con 3
Nest 3
Double 3

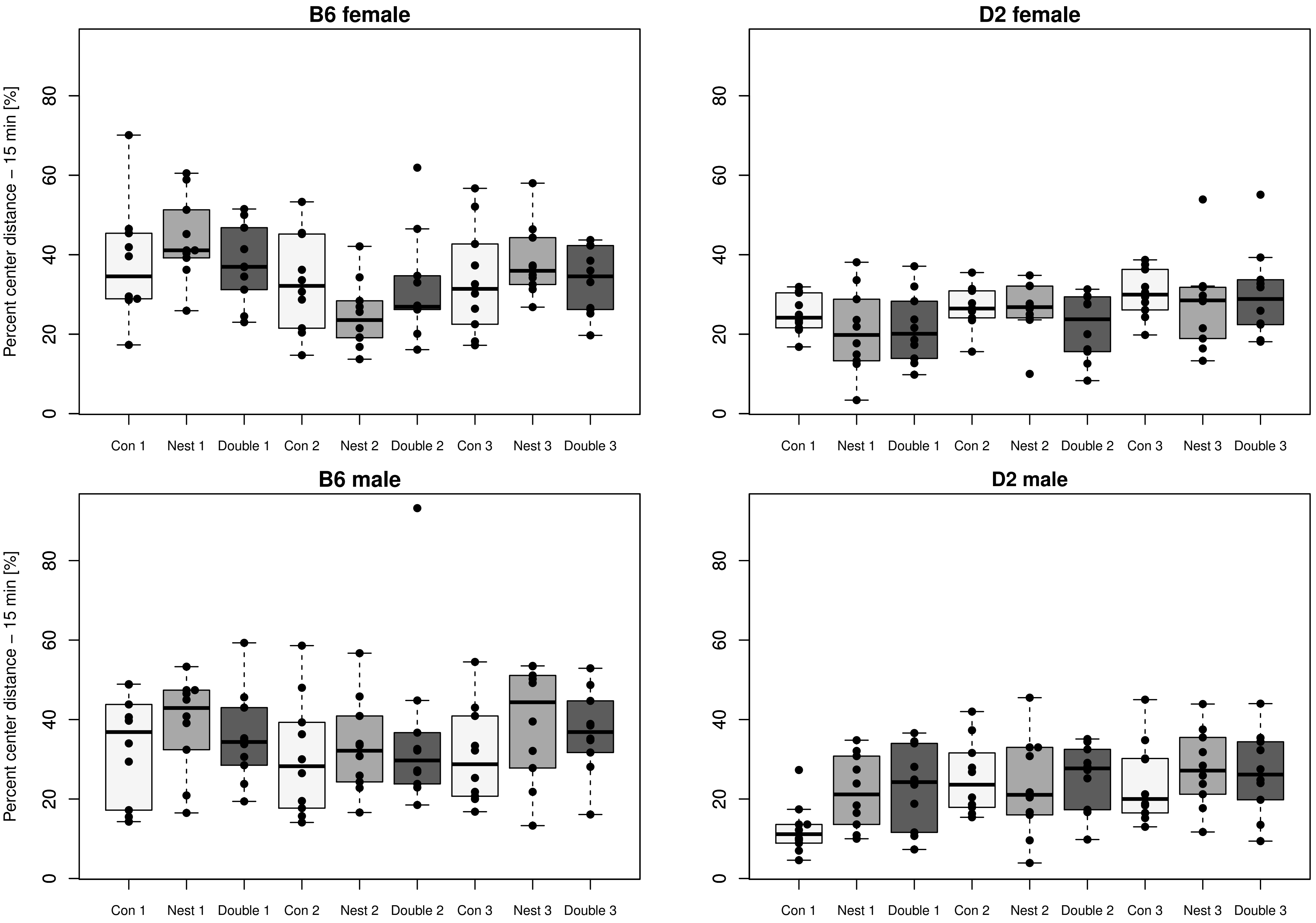

B6 female
D2 female
B6 male
D2 male
Percent center distance – 15 min [%]
0
20
40
60
80
Con 1
Nest 1
Double 1
Con 2
Nest 2
Double 2
Con 3
Nest 3
Double 3

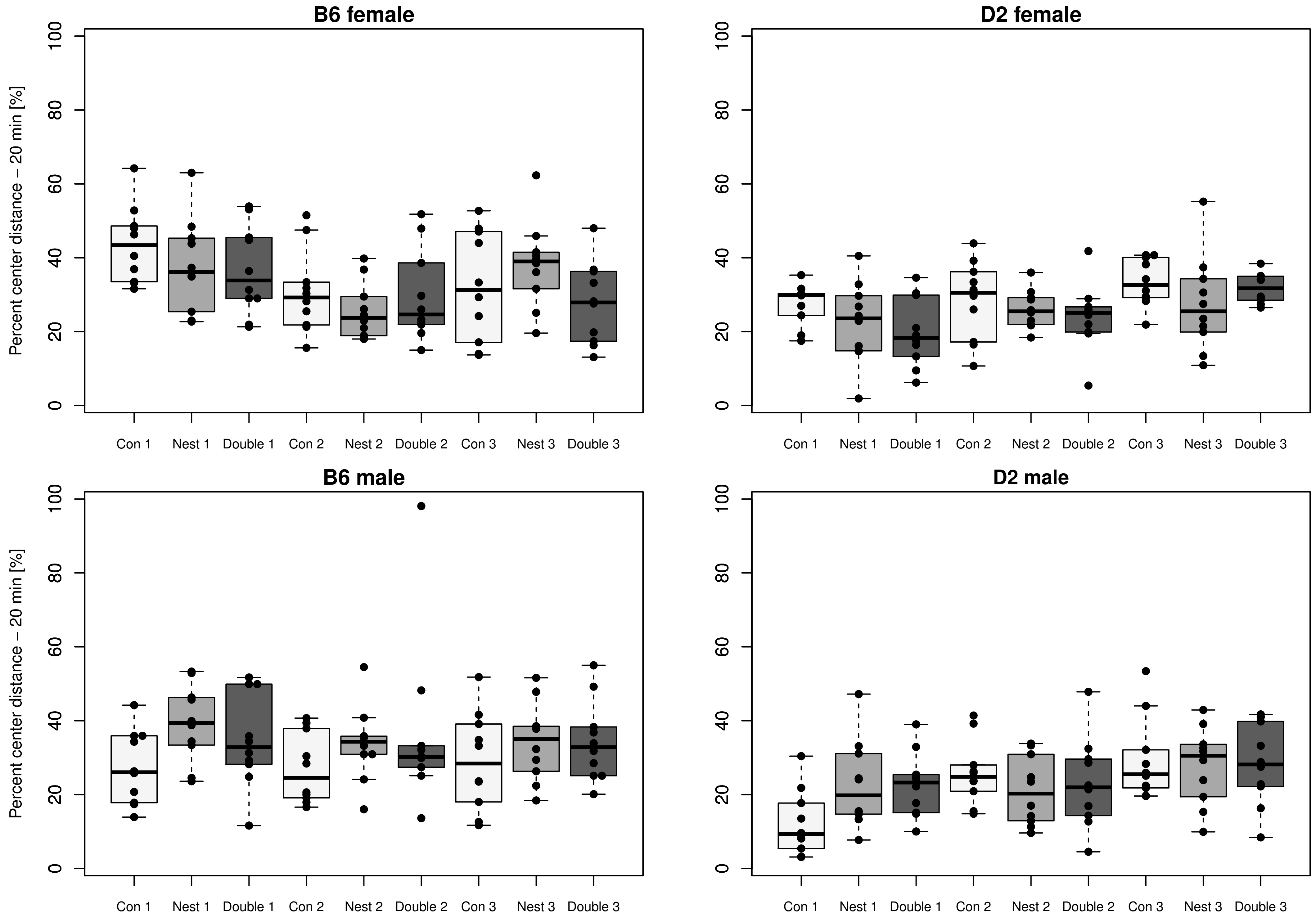

B6 female
D2 female
B6 male
D2 male
Percent center distance – 20 min [%]
0
20
40
60
80
100
Con 1
Nest 1
Double 1
Con 2
Nest 2
Double 2
Con 3
Nest 3
Double 3

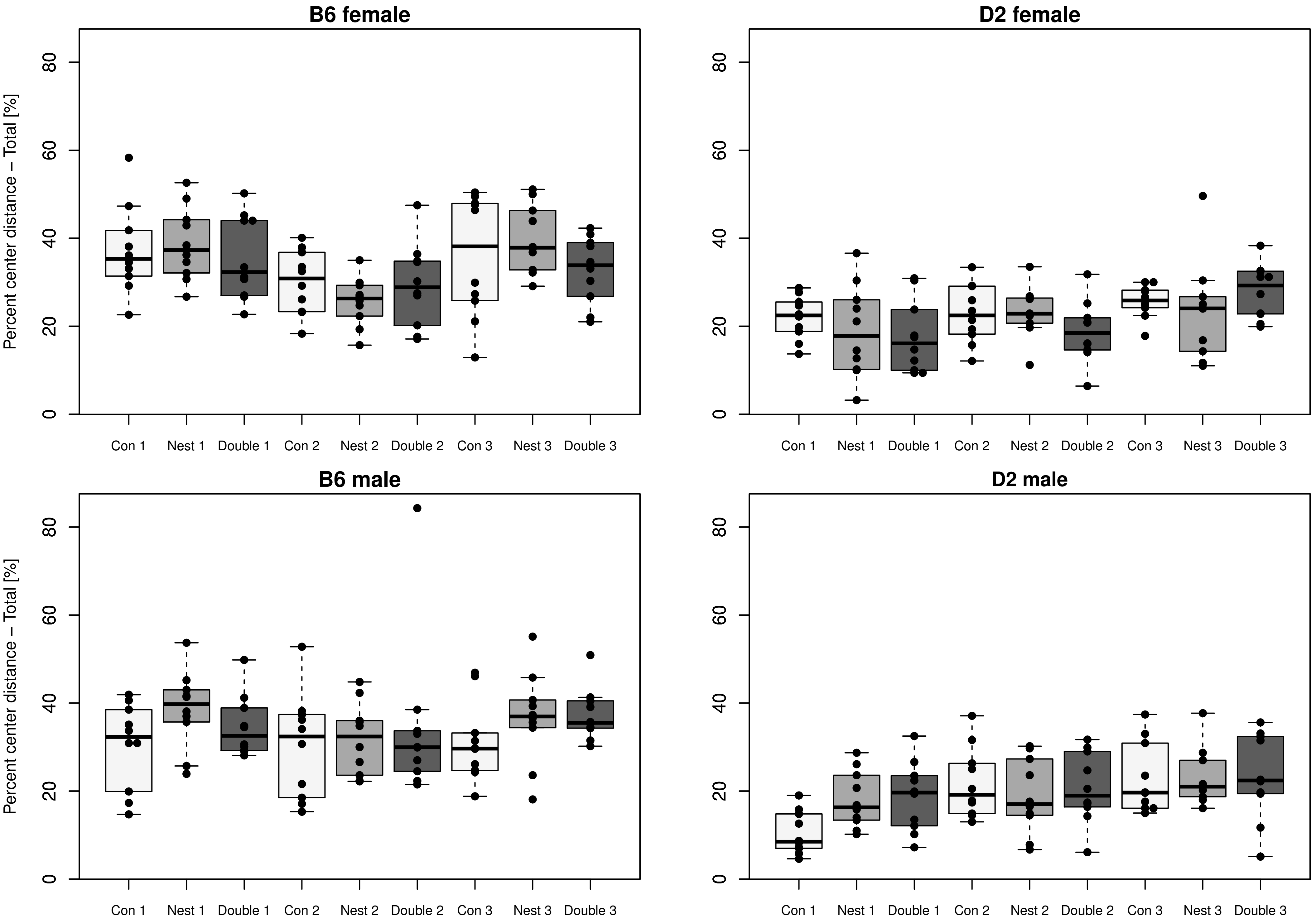

B6 female
D2 female
B6 male
D2 male
Percent center distance – Total [%]
0
20
40
60
80
Con 1
Nest 1
Double 1
Con 2
Nest 2
Double 2
Con 3
Nest 3
Double 3

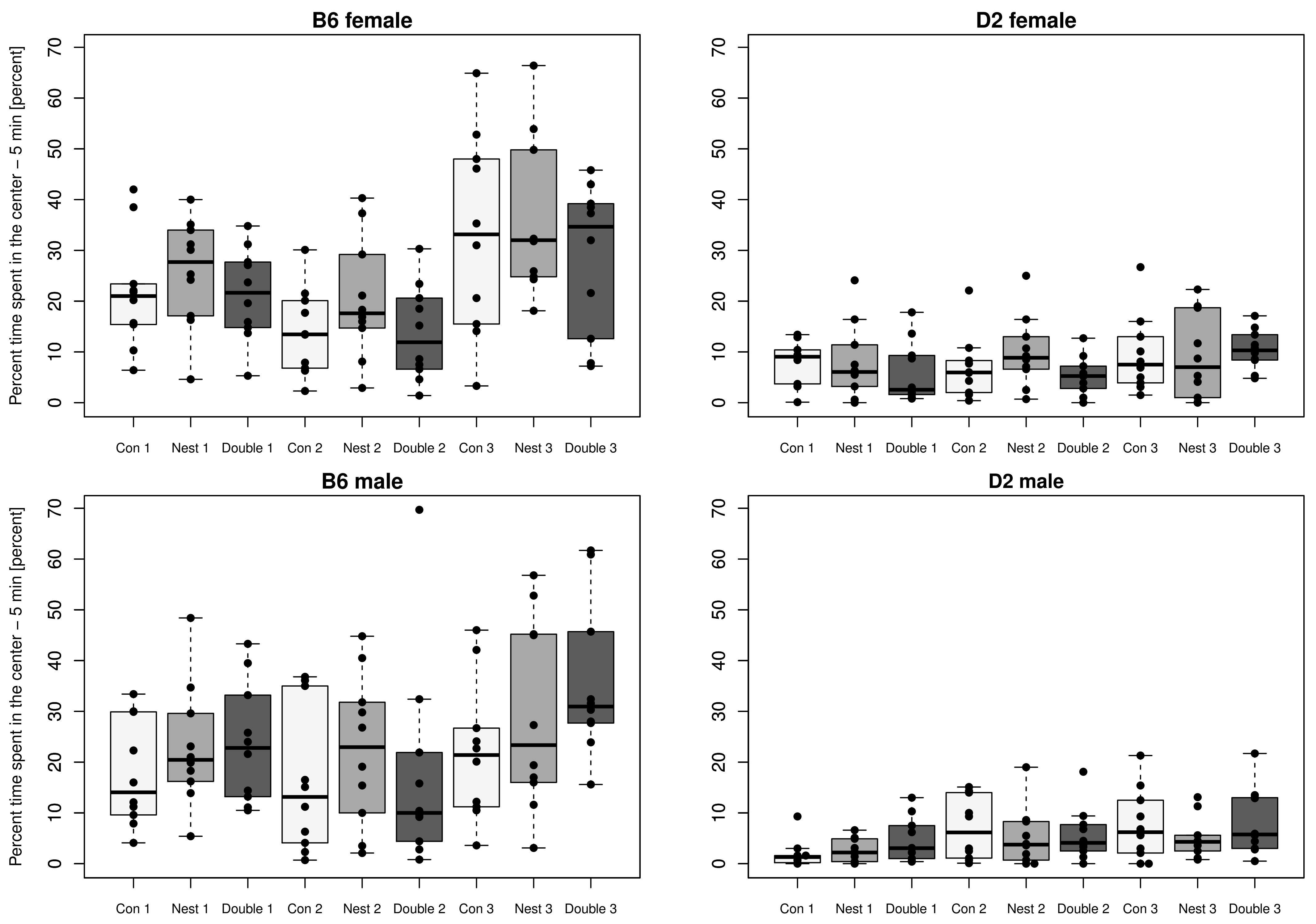

B6 female
D2 female
B6 male
D2 male
Percent time spent in the center – 5 min [percent]
0
10
20
30
40
50
60
70
Con 1
Nest 1
Double 1
Con 2
Nest 2
Double 2
Con 3
Nest 3
Double 3

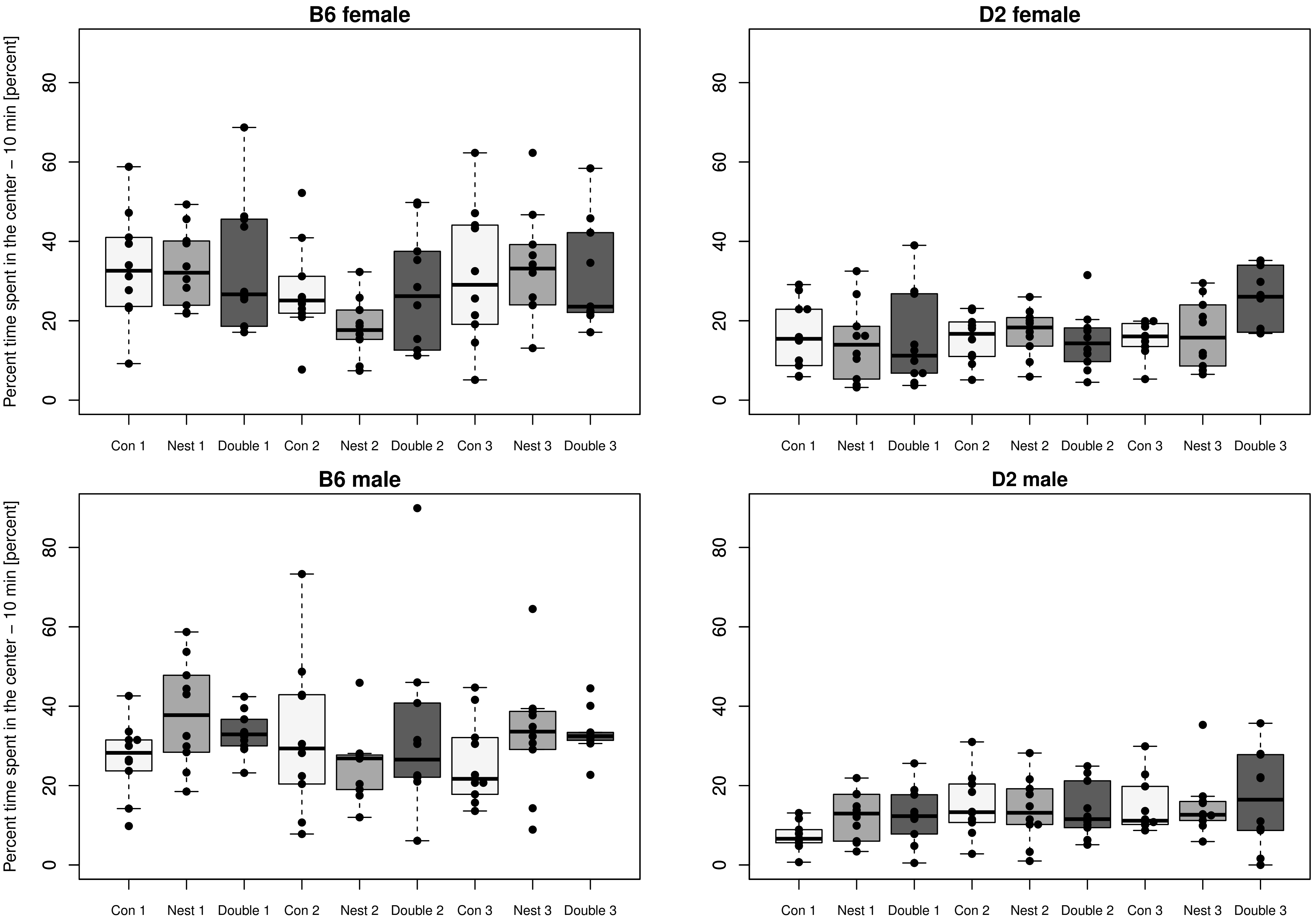

B6 female
D2 female
B6 male
D2 male
Percent time spent in the center – 10 min [percent]
0
20
40
60
80
Con 1
Nest 1
Double 1
Con 2
Nest 2
Double 2
Con 3
Nest 3
Double 3

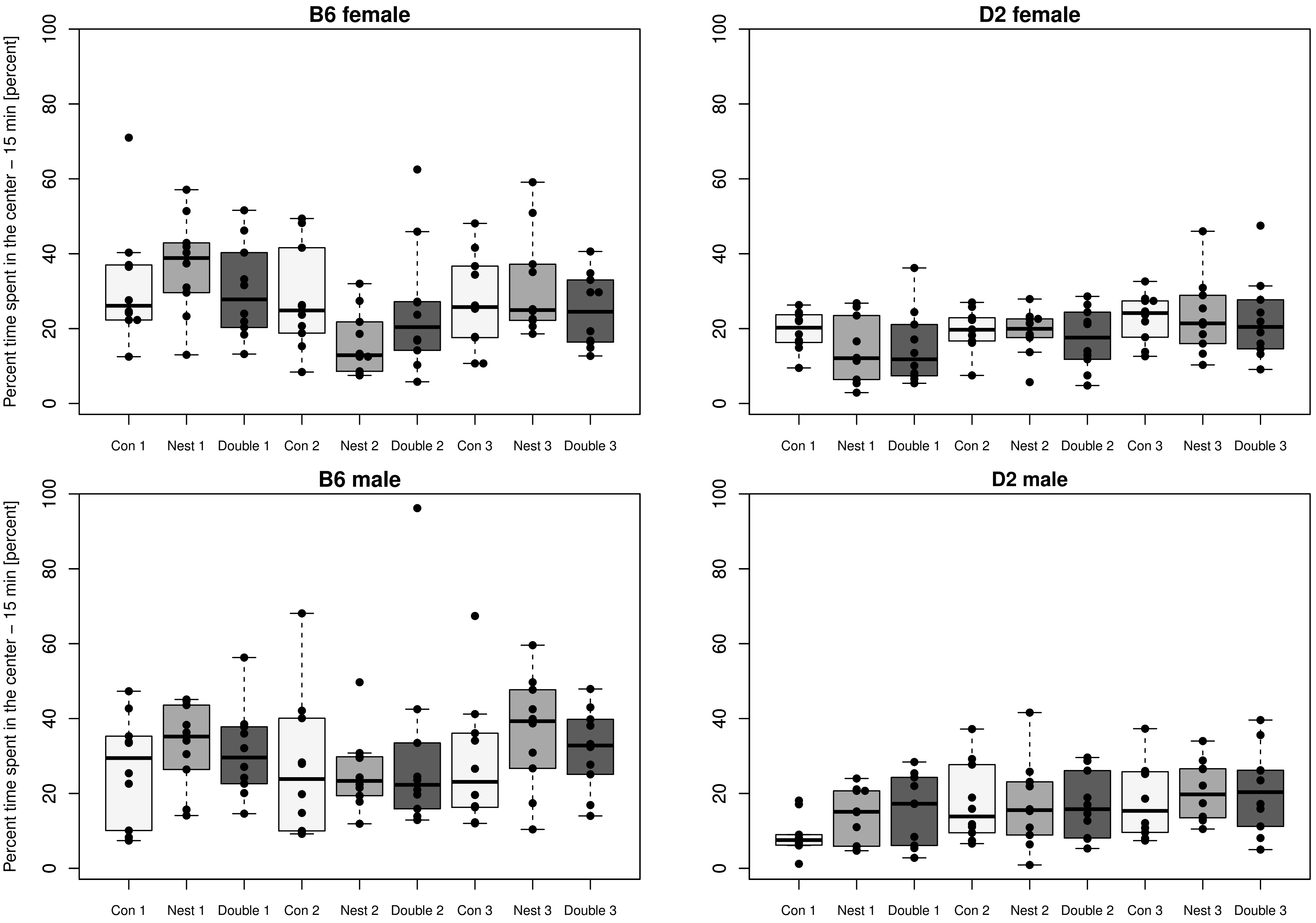
B6 female
D2 female
B6 male
D2 male
Percent time spent in the center – 15 min [percent]
0
20
40
60
80
100
Con 1
Nest 1
Double 1
Con 2
Nest 2
Double 2
Con 3
Nest 3
Double 3

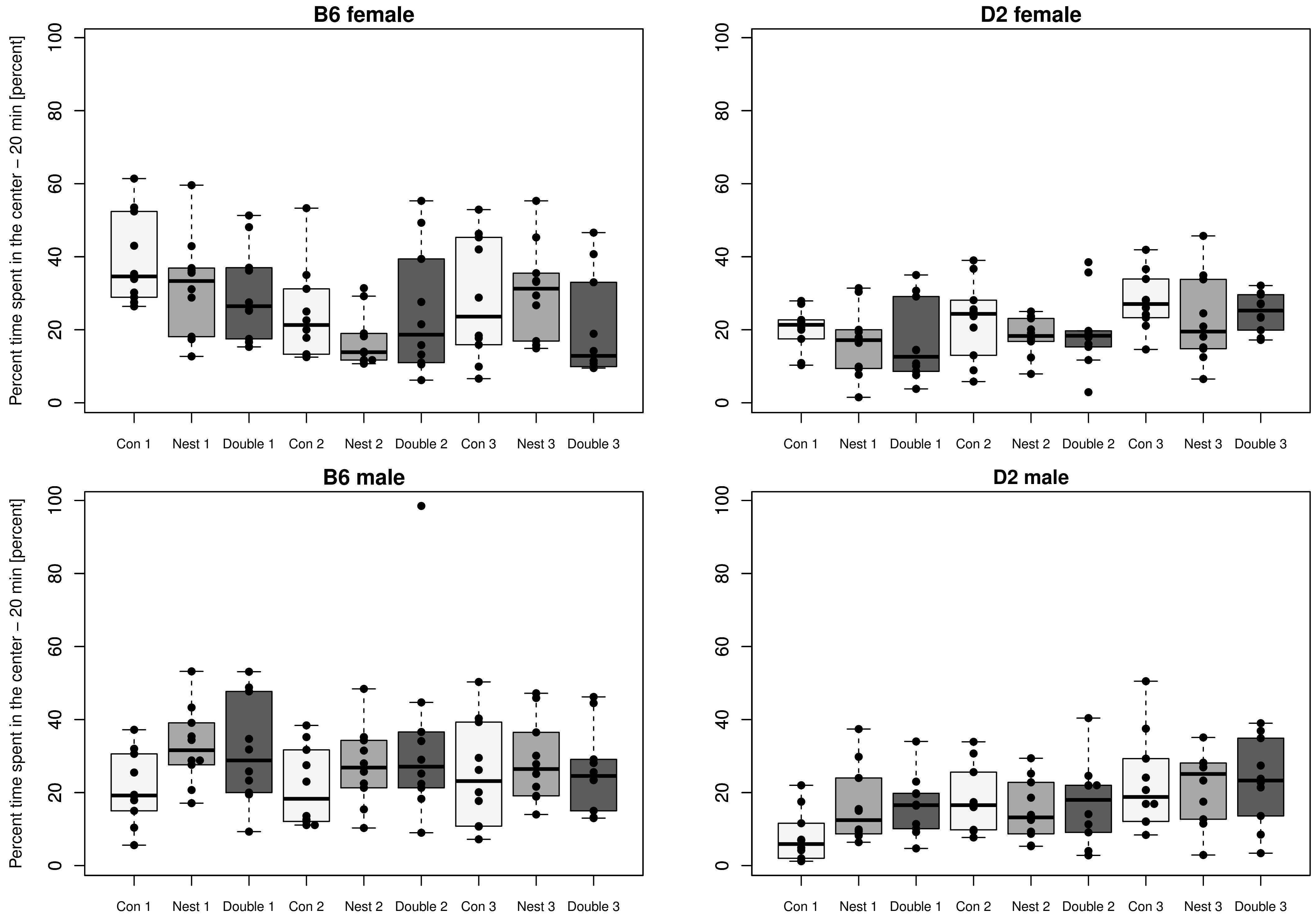
B6 female
D2 female
B6 male
D2 male
Percent time spent in the center – 20 min [percent]
0
20
40
60
80
100
Con 1
Nest 1
Double 1
Con 2
Nest 2
Double 2
Con 3
Nest 3
Double 3

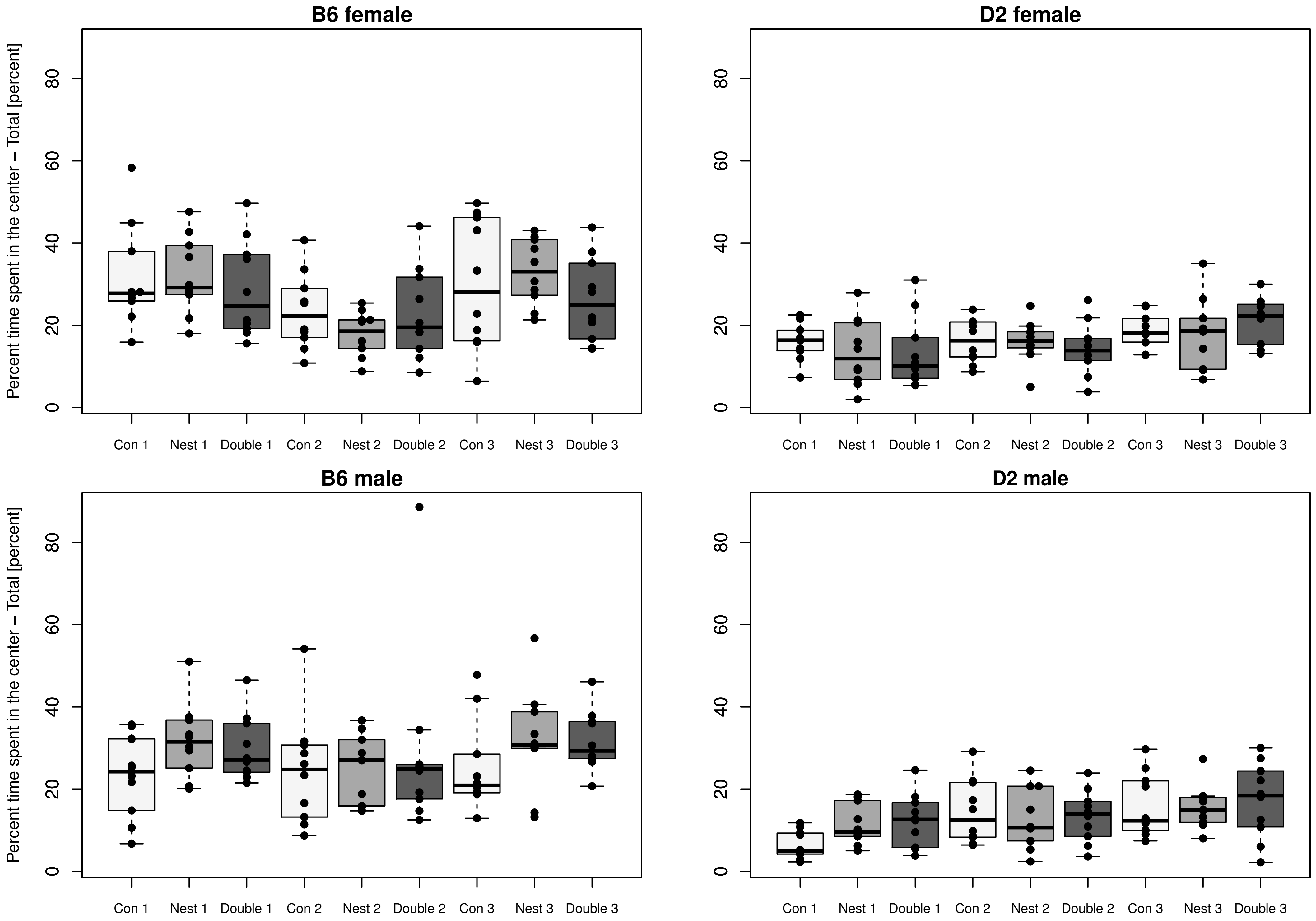

B6 female
D2 female
B6 male
D2 male
Percent time spent in the center – Total [percent]
0
20
40
60
80
Con 1
Nest 1
Double 1
Con 2
Nest 2
Double 2
Con 3
Nest 3
Double 3

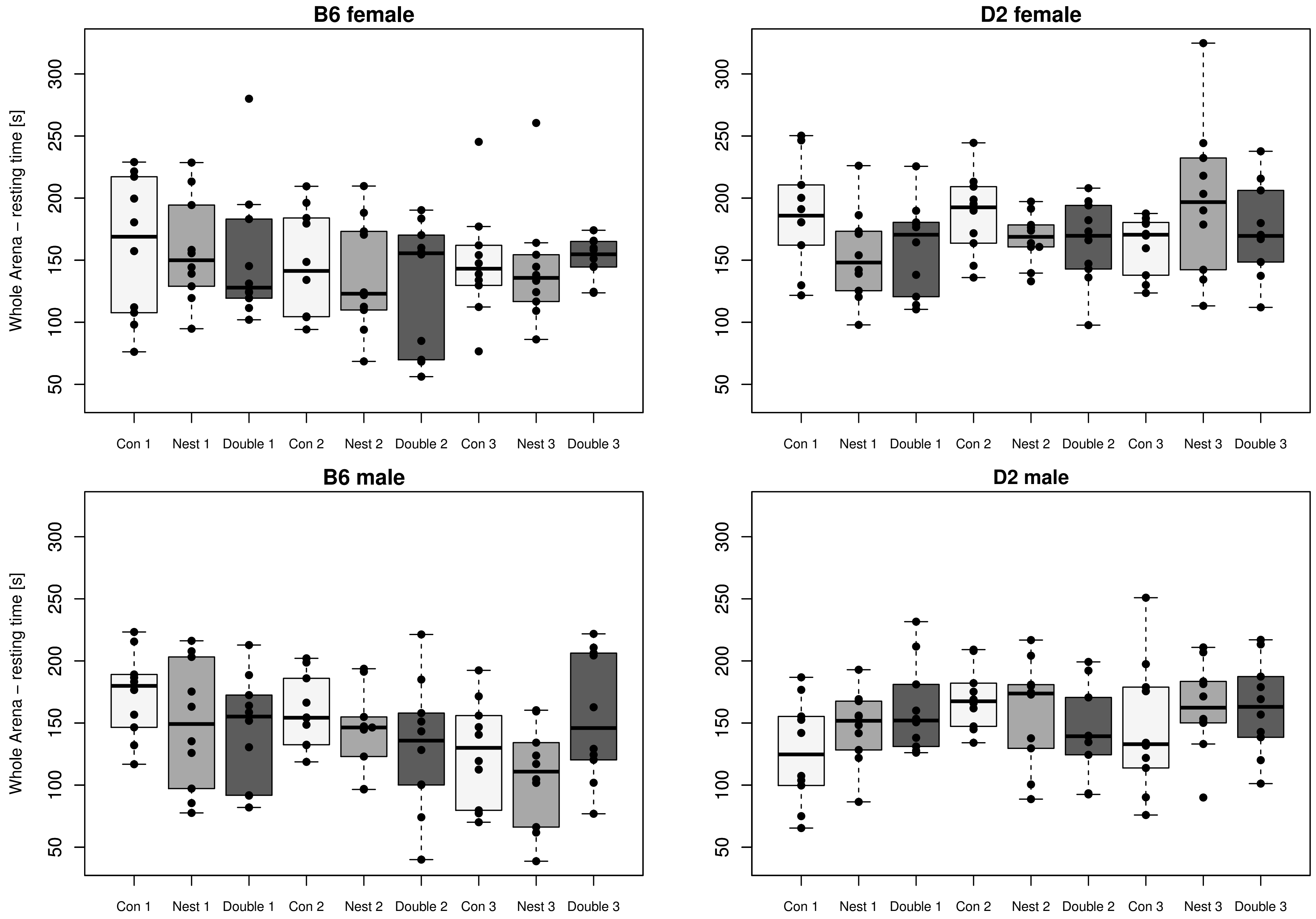
B6 female
D2 female
B6 male
D2 male
Whole Arena – resting time [s]
50
100
150
200
250
300
Con 1
Nest 1
Double 1
Con 2
Nest 2
Double 2
Con 3
Nest 3
Double 3

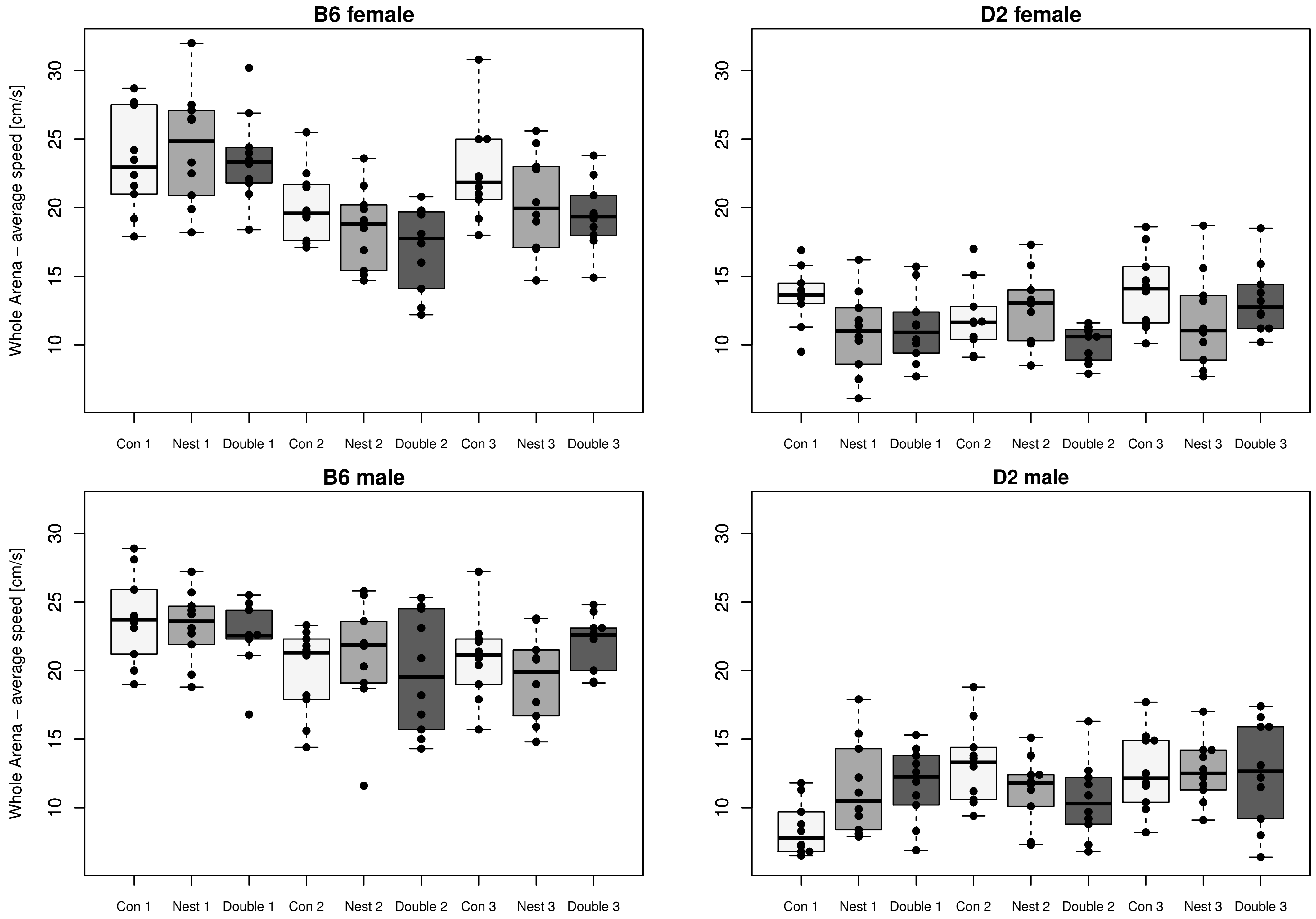

B6 female
D2 female
B6 male
D2 male
Whole Arena – average speed [cm/s]
10
15
20
25
30
Con 1
Nest 1
Double 1
Con 2
Nest 2
Double 2
Con 3
Nest 3
Double 3

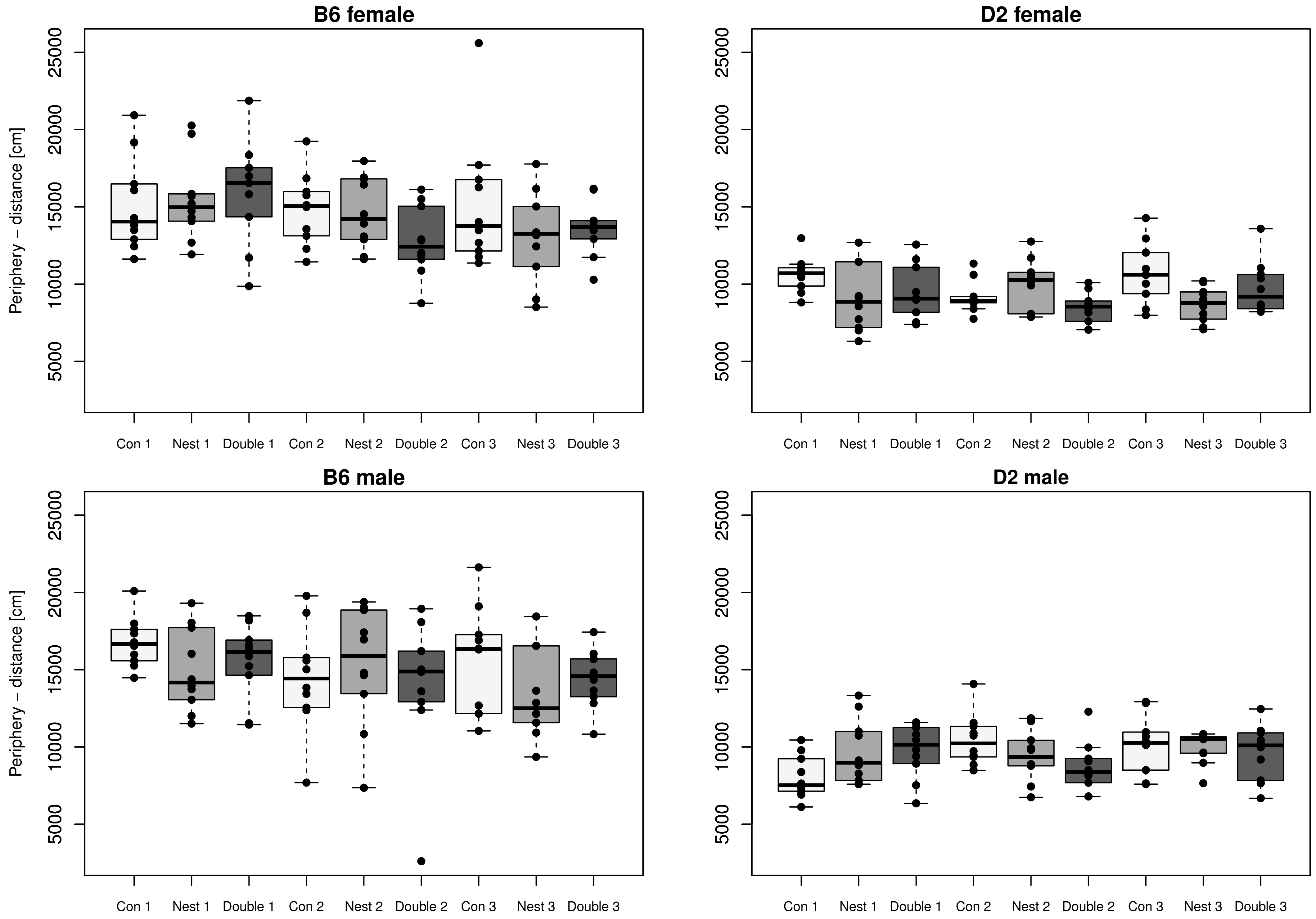

B6 female
D2 female
B6 male
D2 male
Periphery – distance [cm]
5000
10000
15000
20000
25000
Con 1
Nest 1
Double 1
Con 2
Nest 2
Double 2
Con 3
Nest 3
Double 3

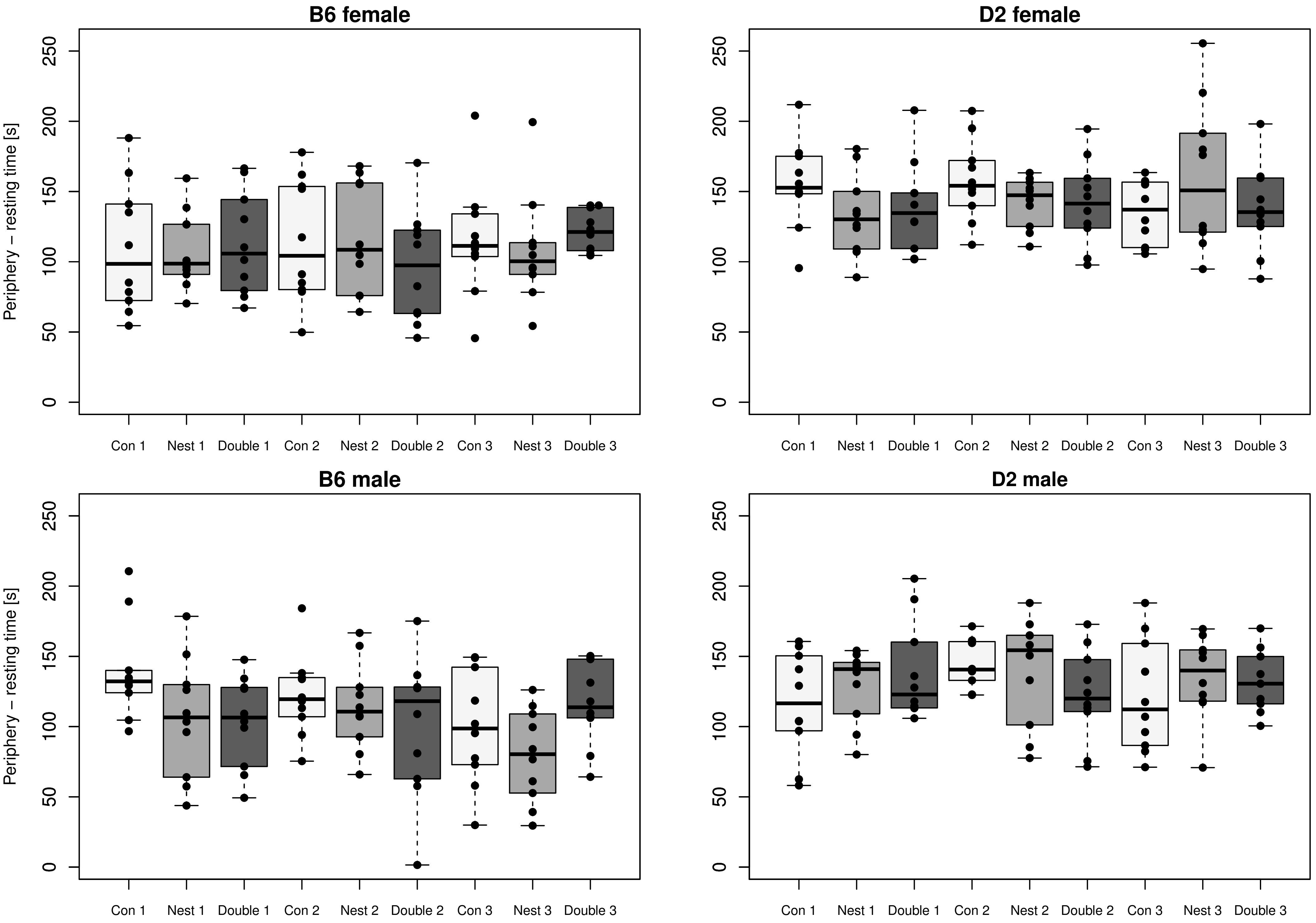
B6 female
D2 female
B6 male
D2 male
Periphery – resting time [s]
0
50
100
150
200
250
Con 1
Nest 1
Double 1
Con 2
Nest 2
Double 2
Con 3
Nest 3
Double 3

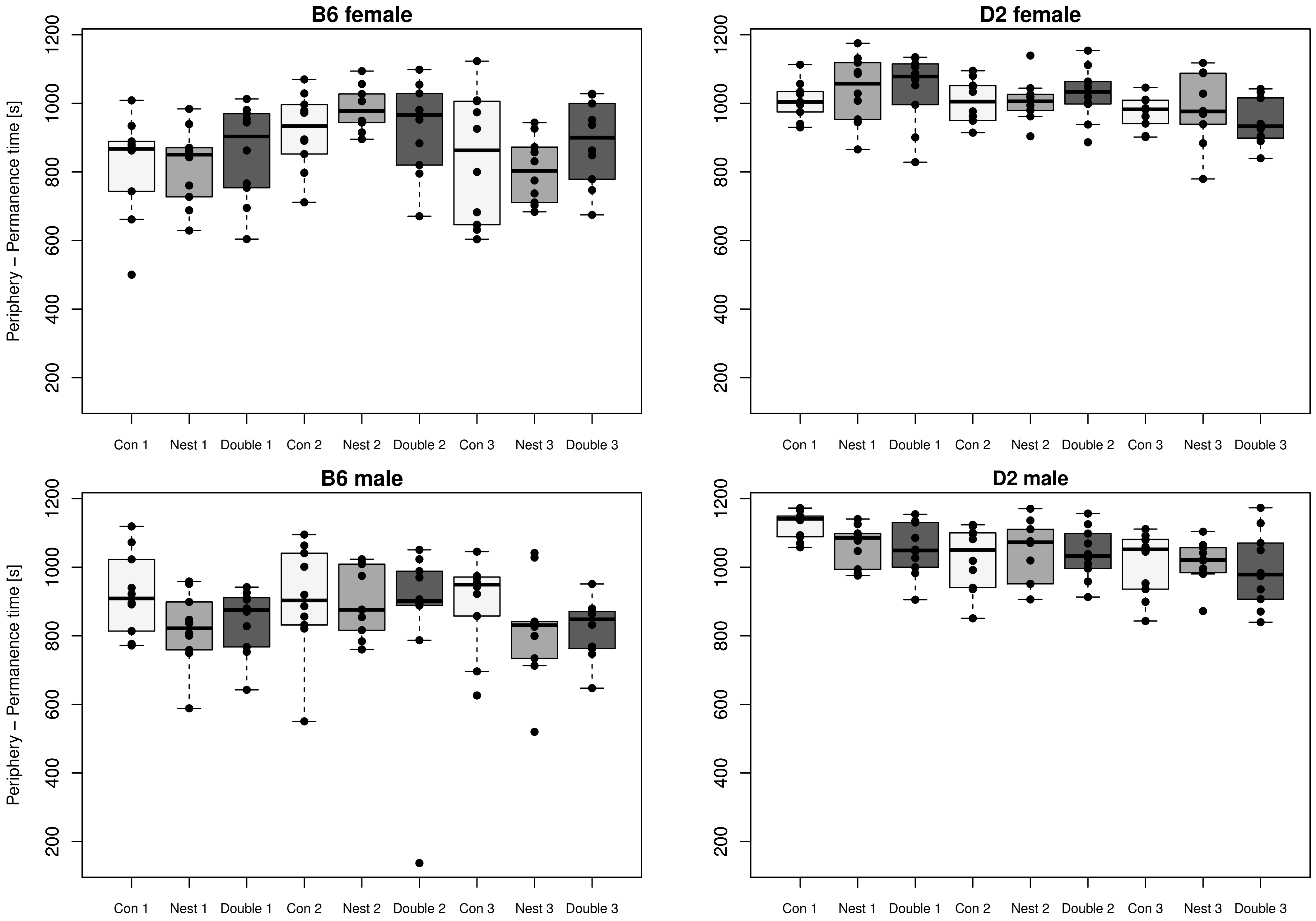
B6 female
D2 female
B6 male
D2 male
Periphery – Permanence time [s]
200
400
600
800
1000
1200
Con 1
Nest 1
Double 1
Con 2
Nest 2
Double 2
Con 3
Nest 3
Double 3

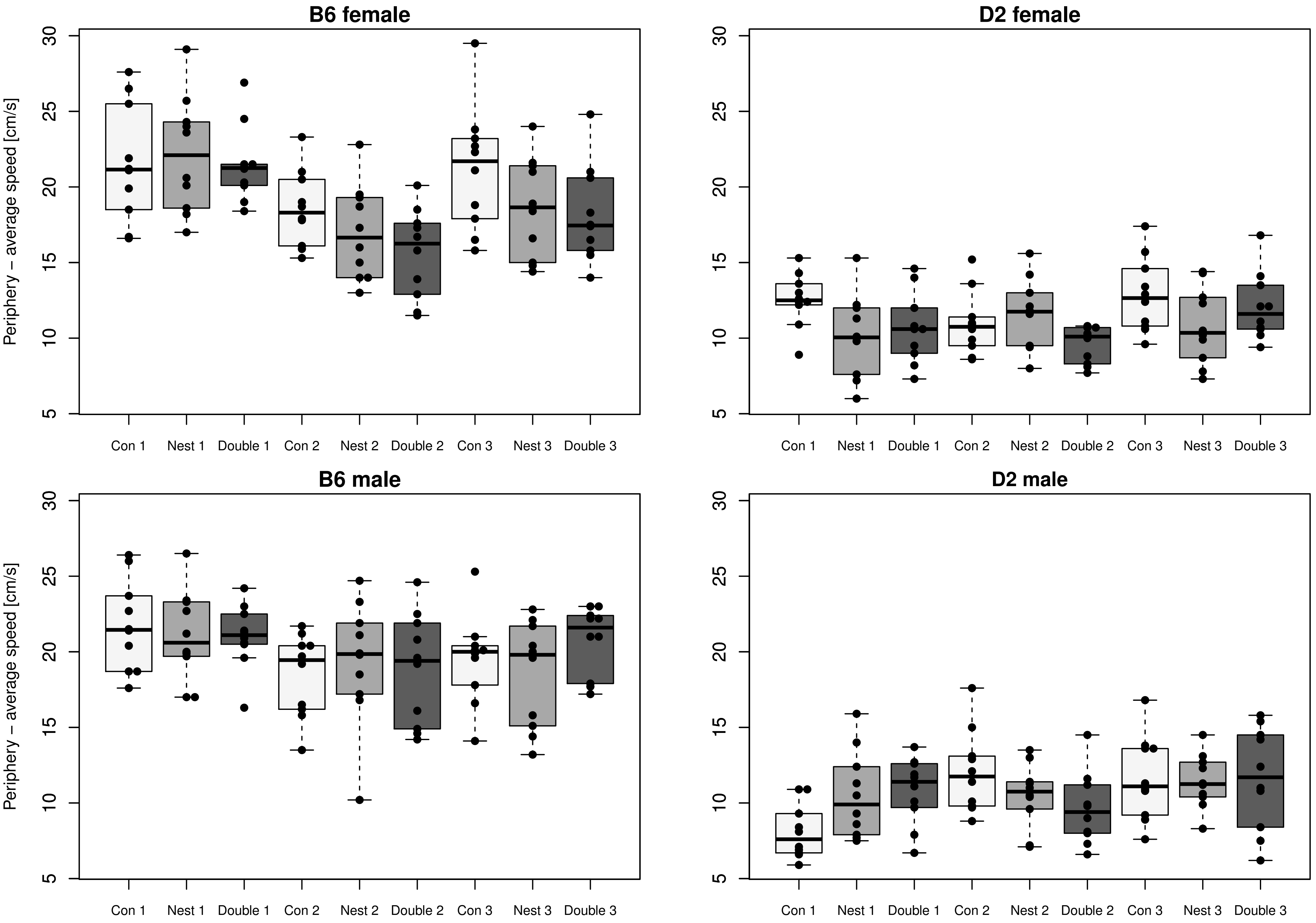

B6 female
D2 female
B6 male
D2 male
Periphery – average speed [cm/s]
Con 1
Nest 1
Double 1
Con 2
Nest 2
Double 2
Con 3
Nest 3
Double 3

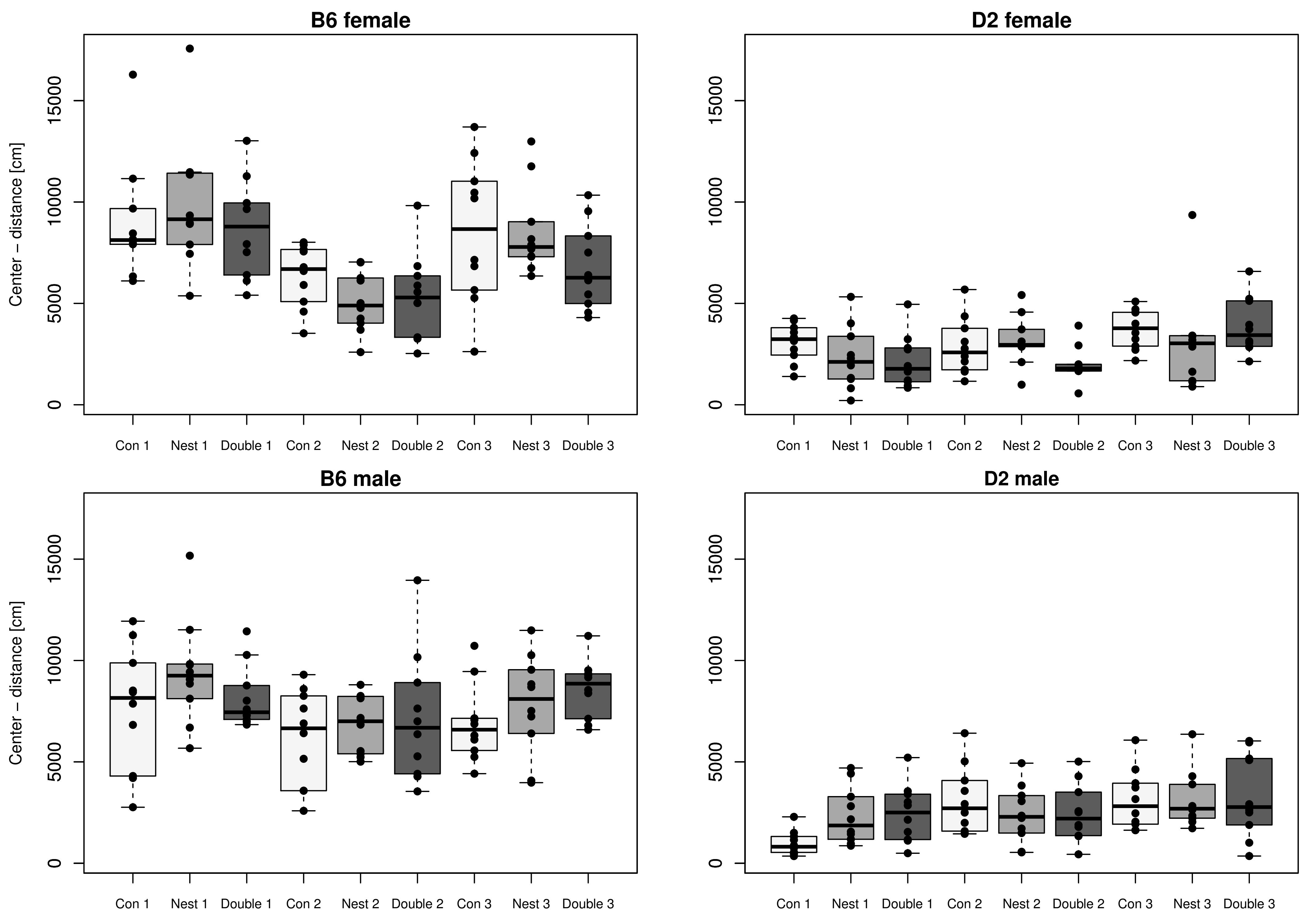
B6 female
D2 female
B6 male
D2 male
Center − distance [cm]
0
5000
10000
15000
Con 1
Nest 1
Double 1
Con 2
Nest 2
Double 2
Con 3
Nest 3
Double 3

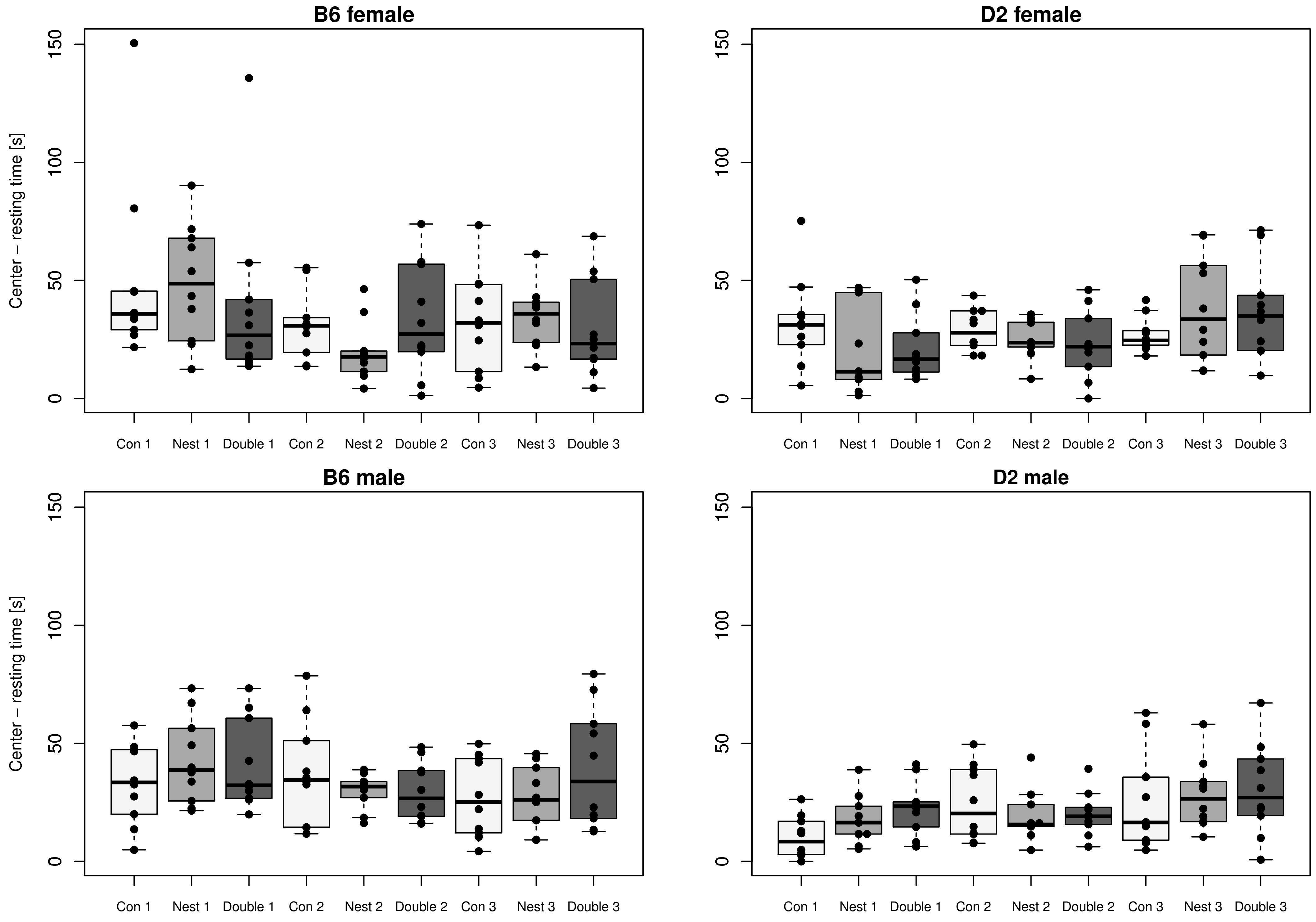

B6 female
D2 female
B6 male
D2 male
Center – resting time [s]
0
50
100
150
Con 1
Nest 1
Double 1
Con 2
Nest 2
Double 2
Con 3
Nest 3
Double 3

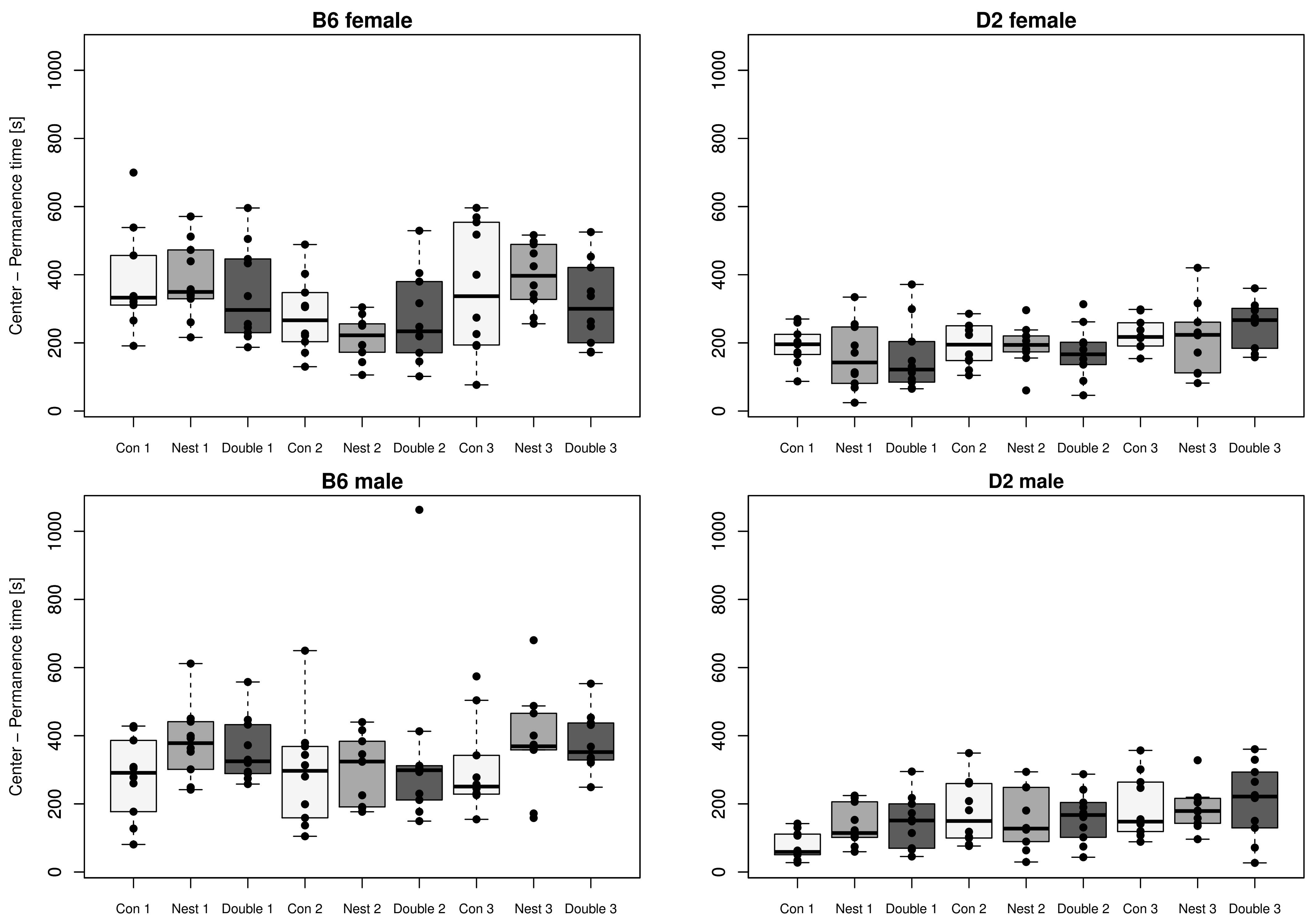
B6 female
D2 female
B6 male
D2 male
Center – Permanence time [s]
0
200
400
600
800
1000
Con 1
Nest 1
Double 1
Con 2
Nest 2
Double 2
Con 3
Nest 3
Double 3

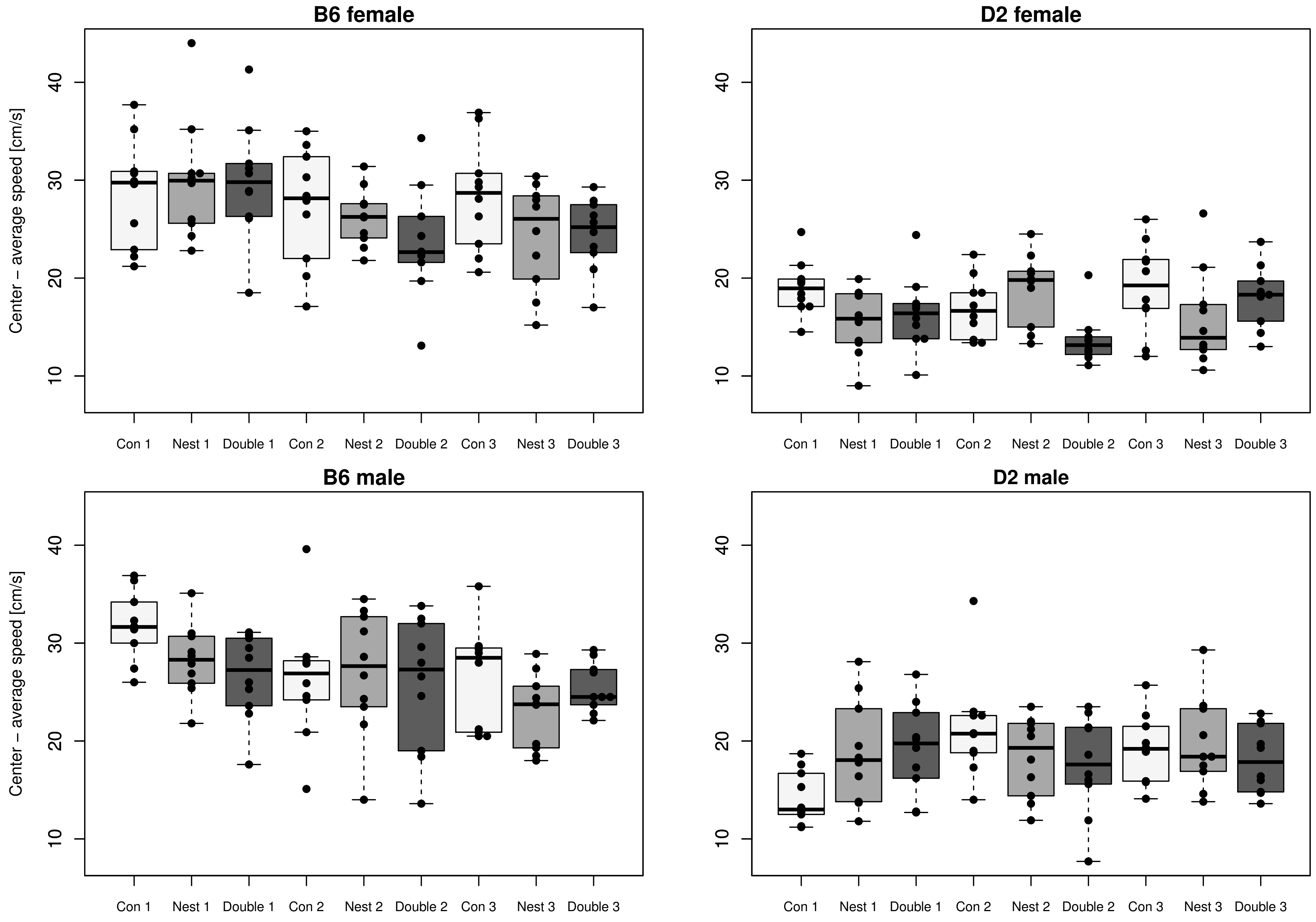

B6 female
D2 female
B6 male
D2 male
Center – average speed [cm/s]
10
20
30
40
Con 1
Nest 1
Double 1
Con 2
Nest 2
Double 2
Con 3
Nest 3
Double 3

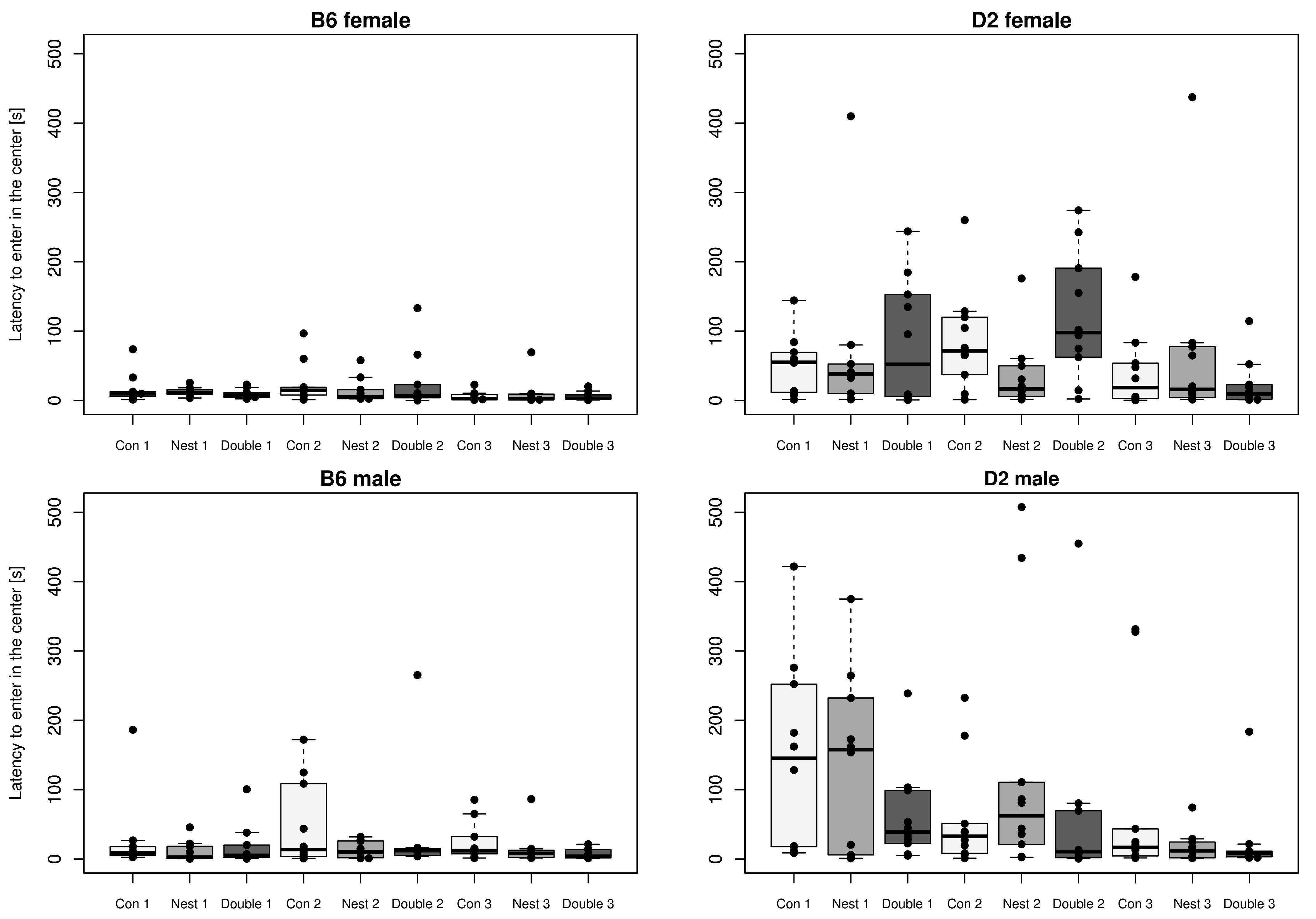
B6 female
D2 female
B6 male
D2 male
Latency to enter in the center [s]
0
100
200
300
400
500
Con 1
Nest 1
Double 1
Con 2
Nest 2
Double 2
Con 3
Nest 3
Double 3

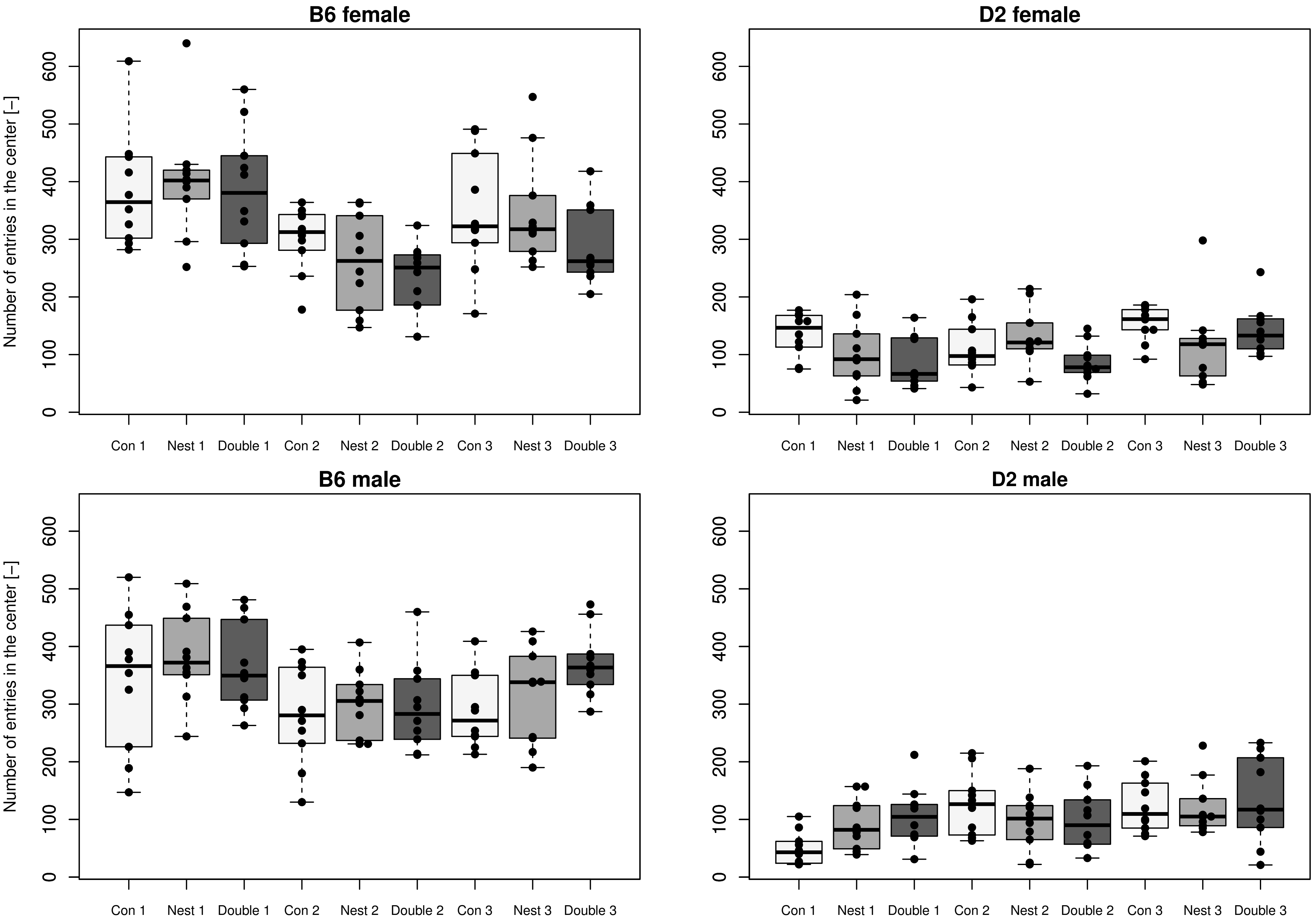
B6 female
D2 female
B6 male
D2 male
Number of entries in the center [–]
0
100
200
300
400
500
600
Con 1
Nest 1
Double 1
Con 2
Nest 2
Double 2
Con 3
Nest 3
Double 3

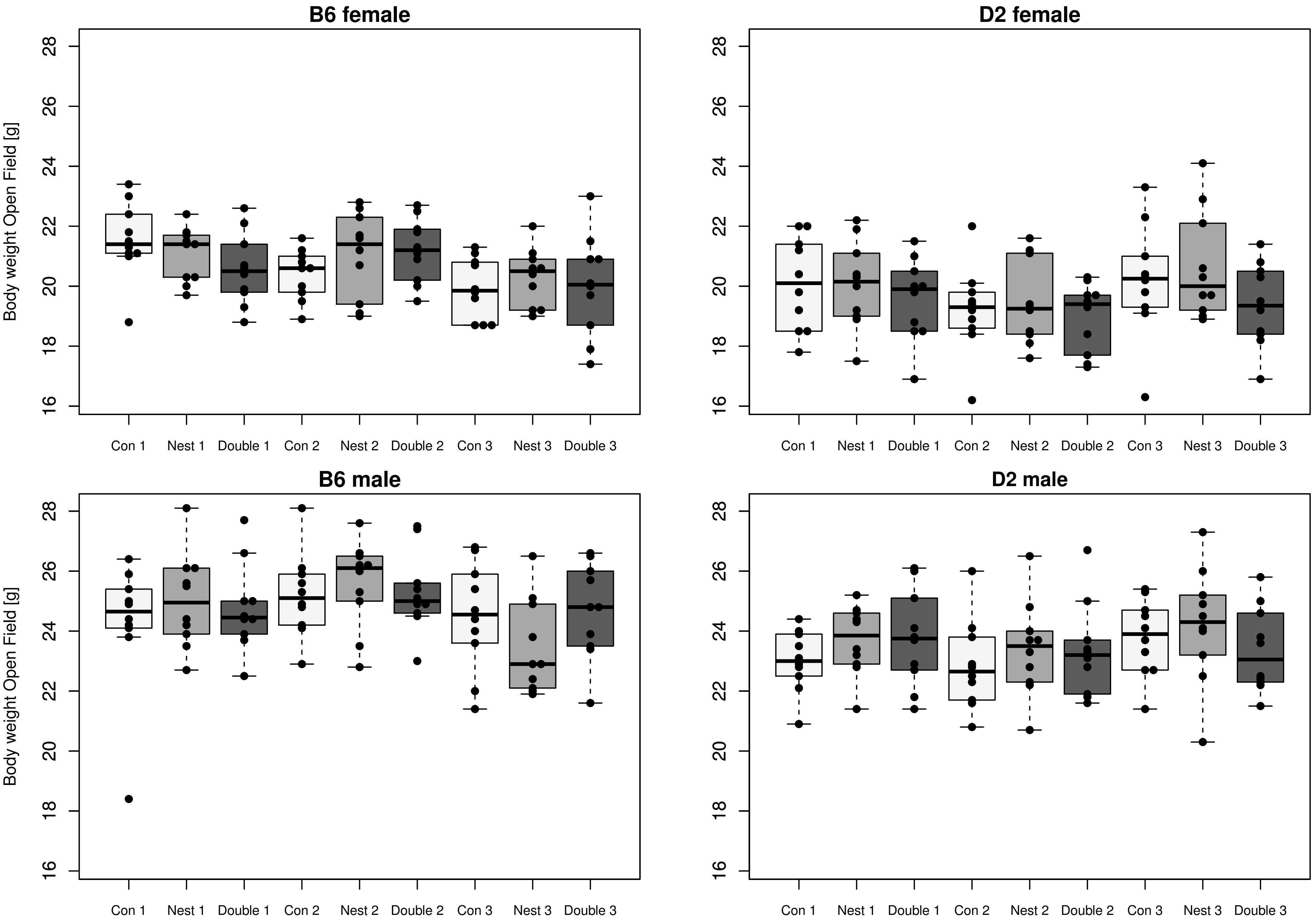
B6 female
D2 female
B6 male
D2 male
Body weight Open Field [g]
16
18
20
22
24
26
28
Con 1
Nest 1
Double 1
Con 2
Nest 2
Double 2
Con 3
Nest 3
Double 3

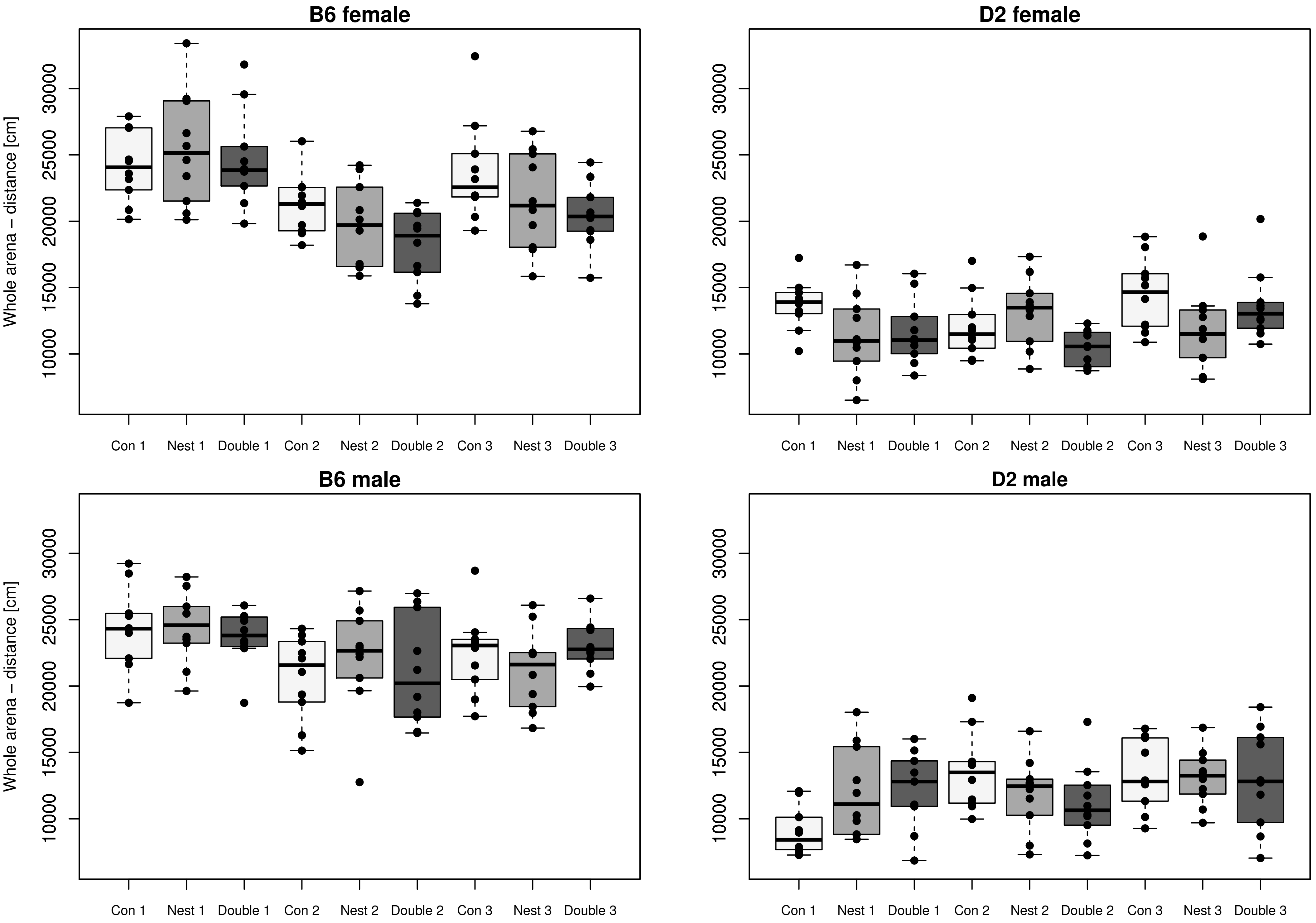

B6 female
D2 female
B6 male
D2 male
Whole arena – distance [cm]
10000
15000
20000
25000
30000
Con 1
Nest 1
Double 1
Con 2
Nest 2
Double 2
Con 3
Nest 3
Double 3

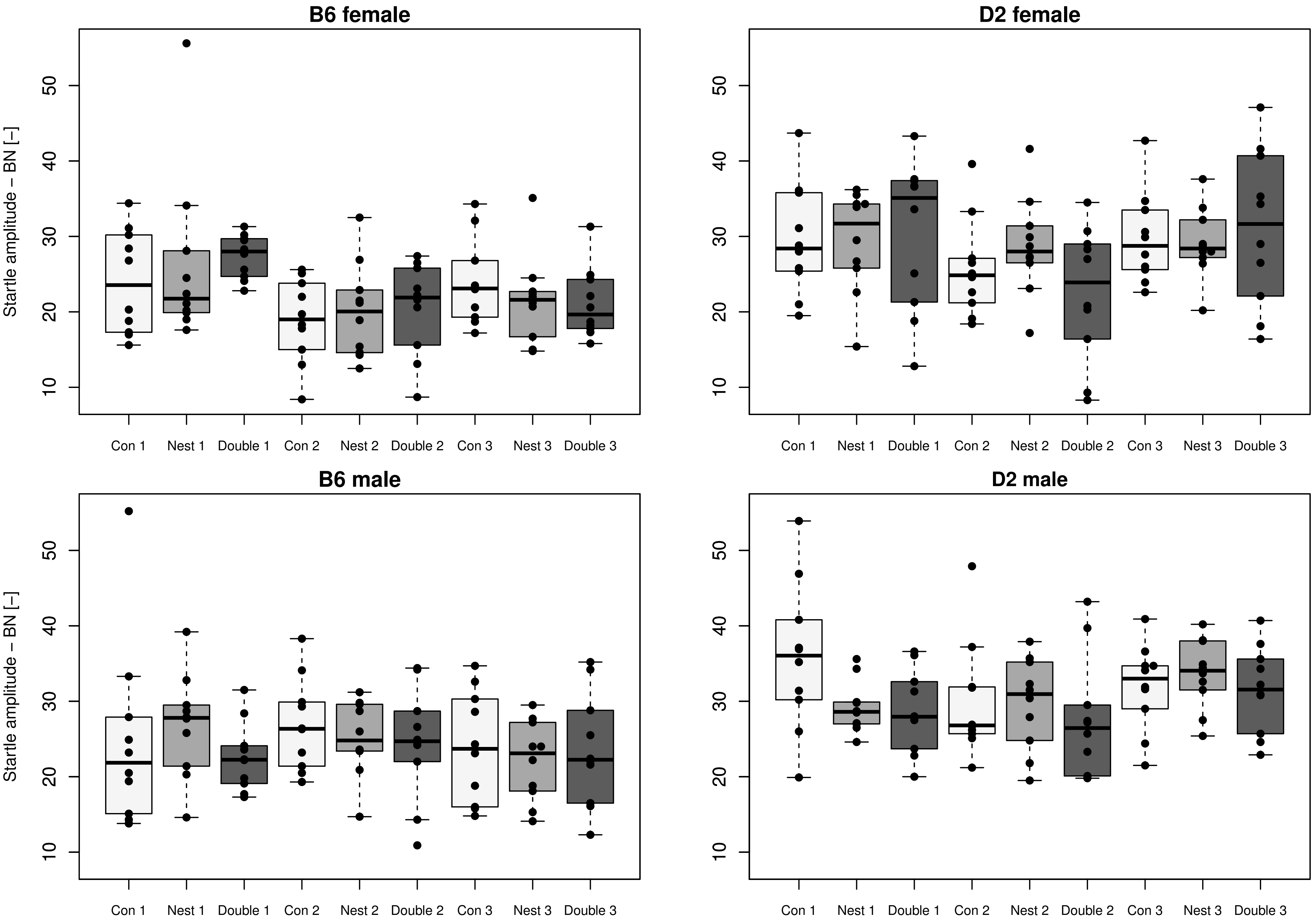
B6 female
D2 female
B6 male
D2 male
Startle amplitude – BN [–]
10
20
30
40
50
Con 1
Nest 1
Double 1
Con 2
Nest 2
Double 2
Con 3
Nest 3
Double 3

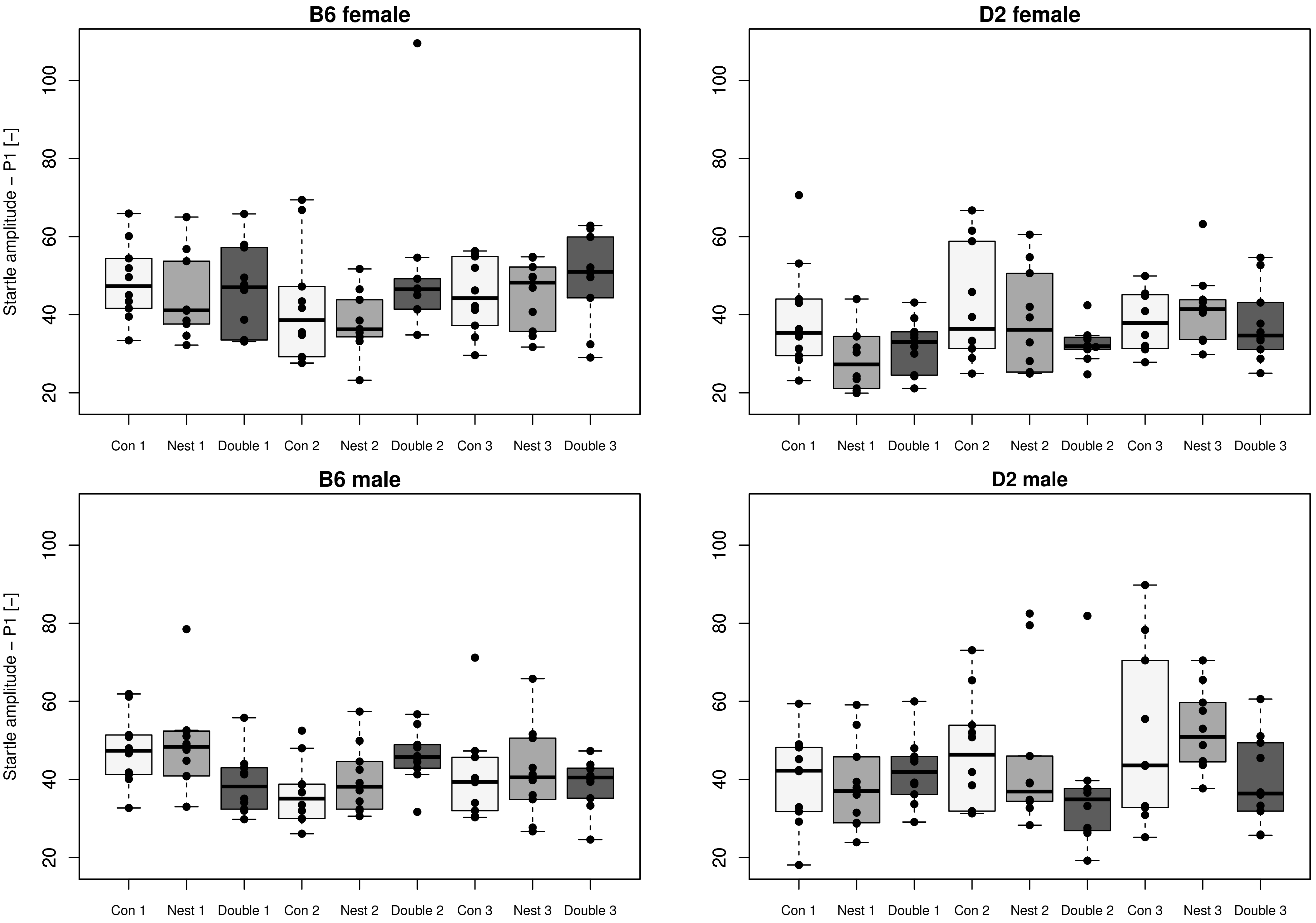
B6 female
D2 female
B6 male
D2 male
Startle amplitude – P1 [–]
20
40
60
80
100
Con 1
Nest 1
Double 1
Con 2
Nest 2
Double 2
Con 3
Nest 3
Double 3

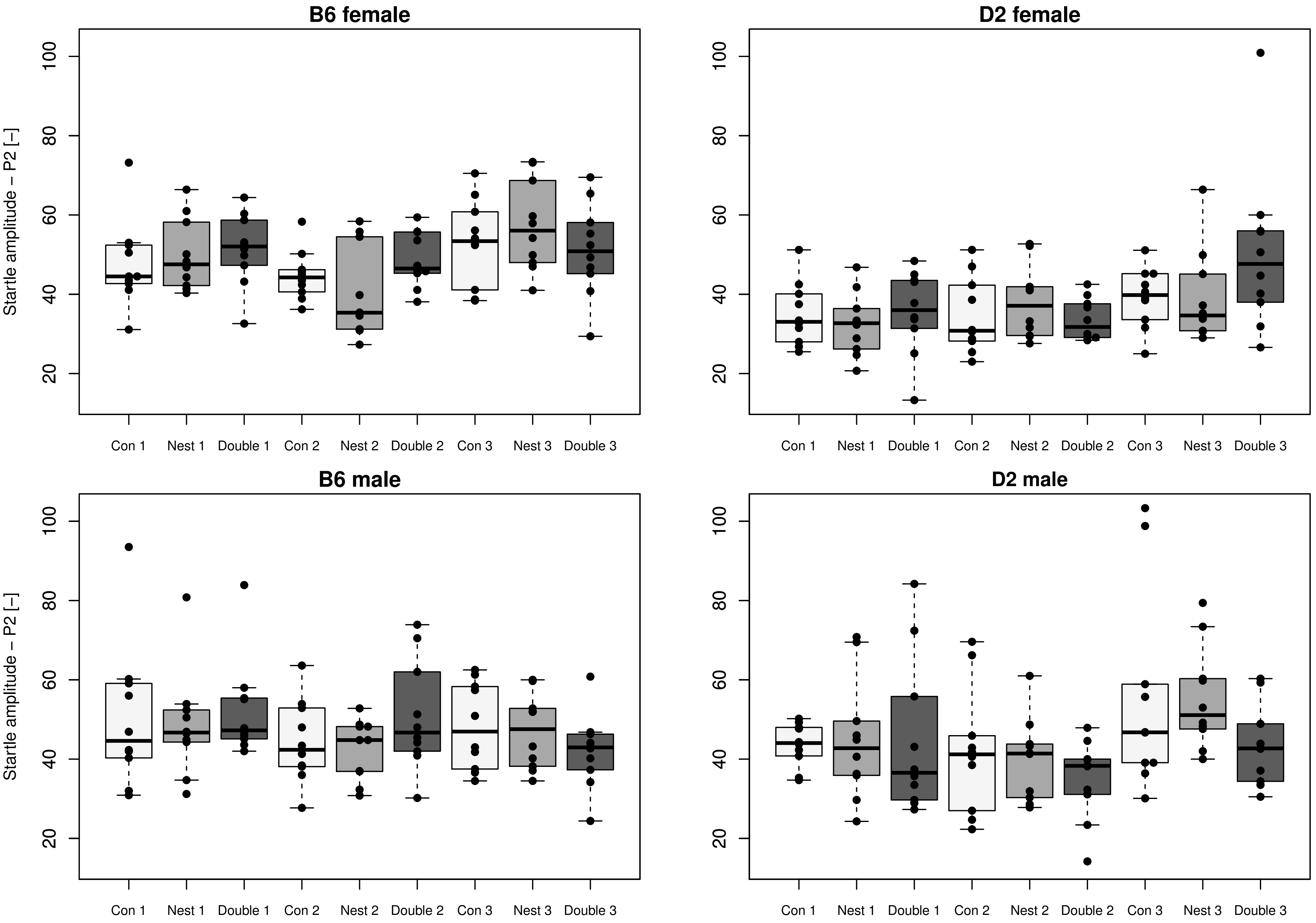
B6 female
D2 female
B6 male
D2 male
Startle amplitude – P2 [–]
Startle amplitude – P2 [–]
20
40
60
80
100
Con 1
Nest 1
Double 1
Con 2
Nest 2
Double 2
Con 3
Nest 3
Double 3

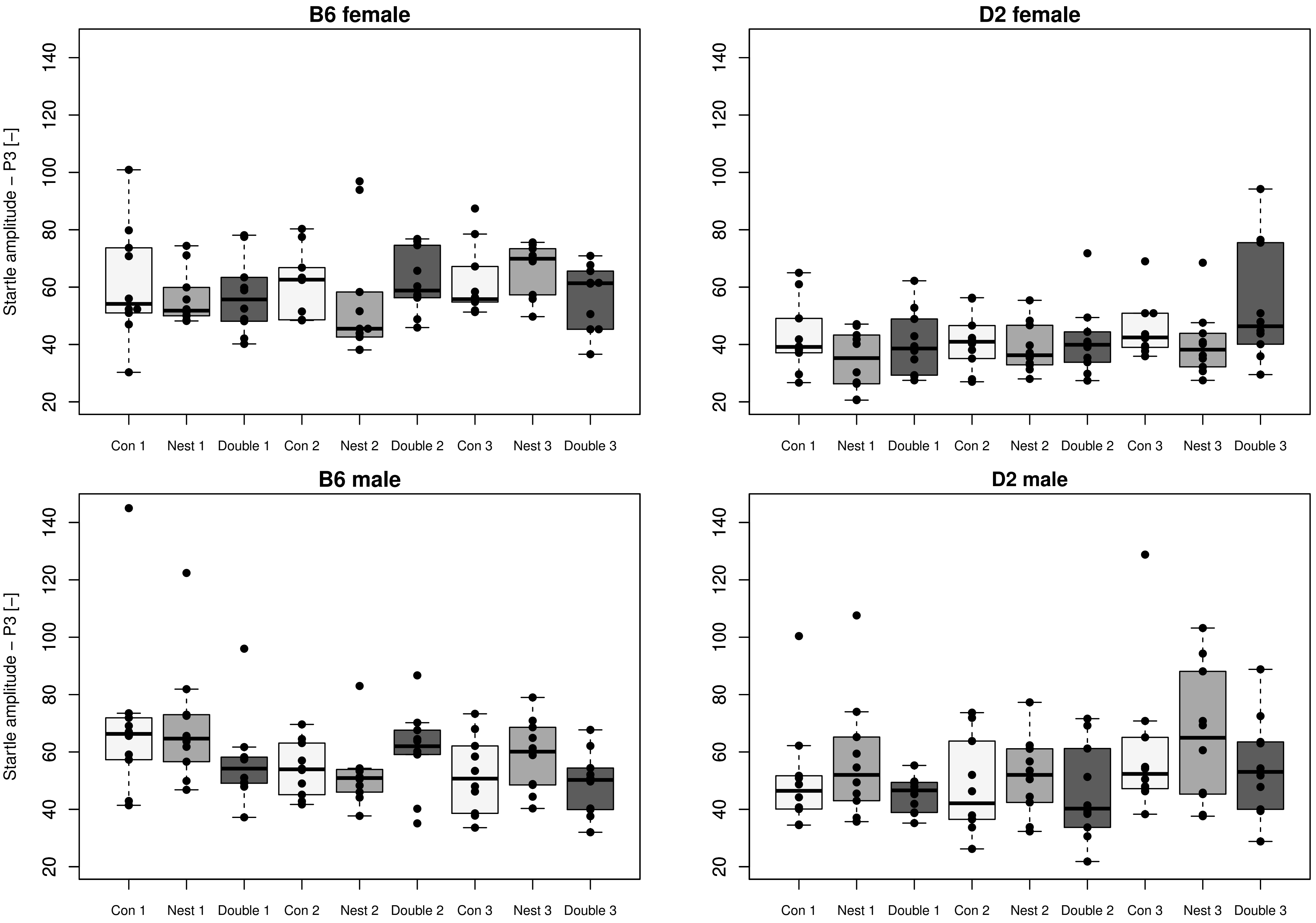
B6 female
D2 female
B6 male
D2 male
Startle amplitude – P3 [–]
20
40
60
80
100
120
140
Con 1
Nest 1
Double 1
Con 2
Nest 2
Double 2
Con 3
Nest 3
Double 3

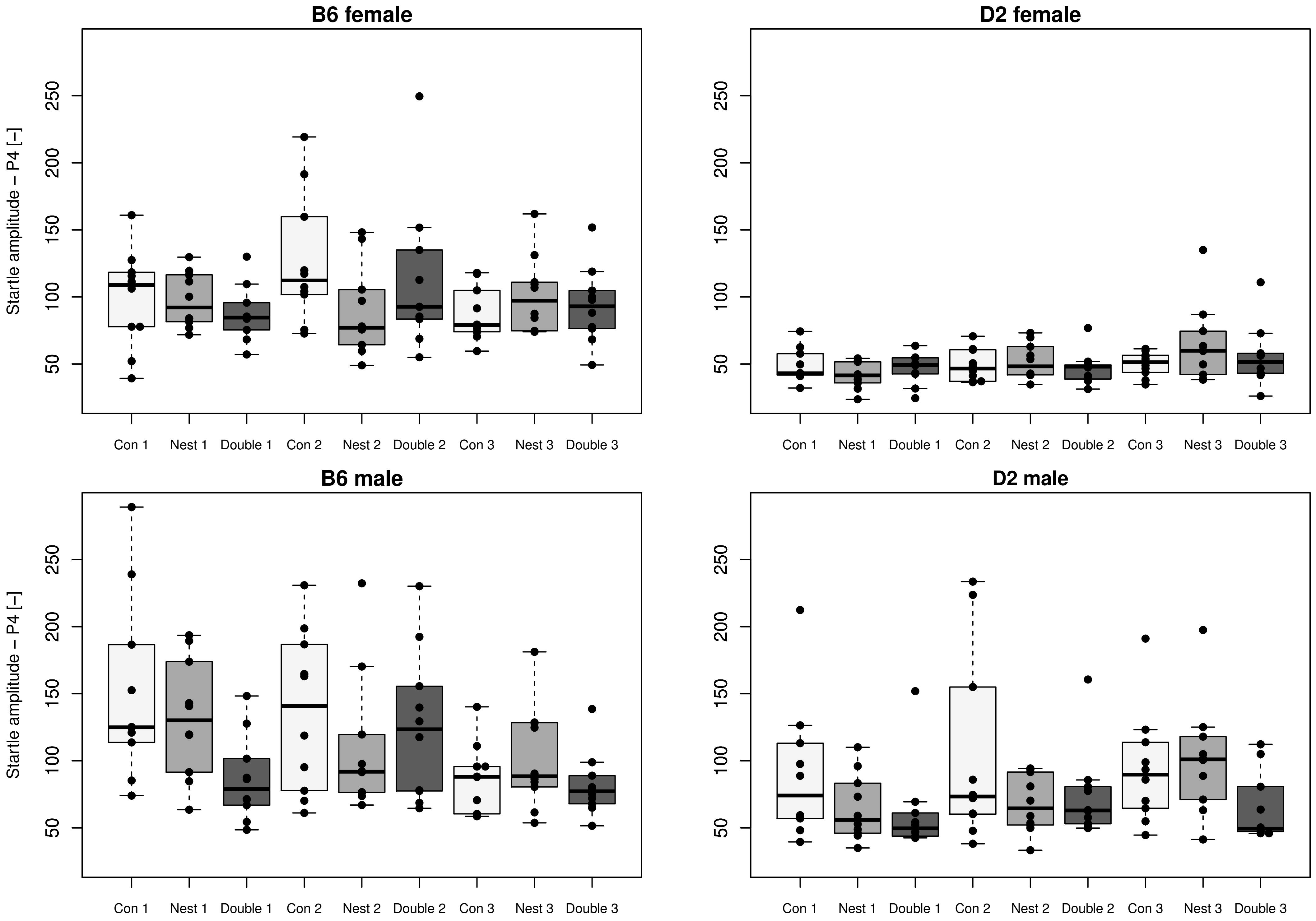
B6 female
D2 female
B6 male
D2 male
Startle amplitude – P4 [–]
50
100
150
200
250
Con 1
Nest 1
Double 1
Con 2
Nest 2
Double 2
Con 3
Nest 3
Double 3

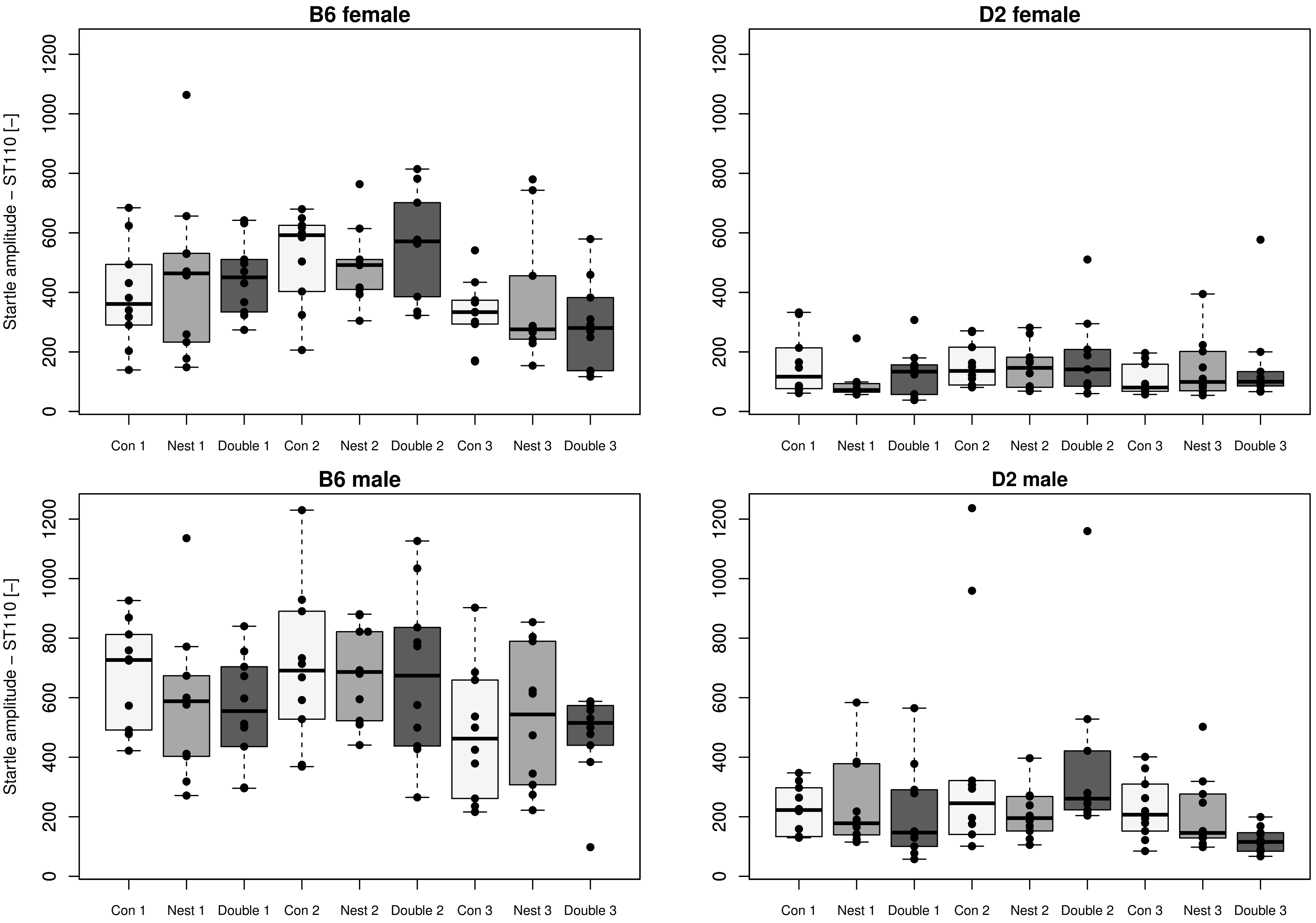
B6 female
D2 female
B6 male
D2 male
Startle amplitude – ST110 [–]
0
200
400
600
800
1000
1200
Con 1
Nest 1
Double 1
Con 2
Nest 2
Double 2
Con 3
Nest 3
Double 3

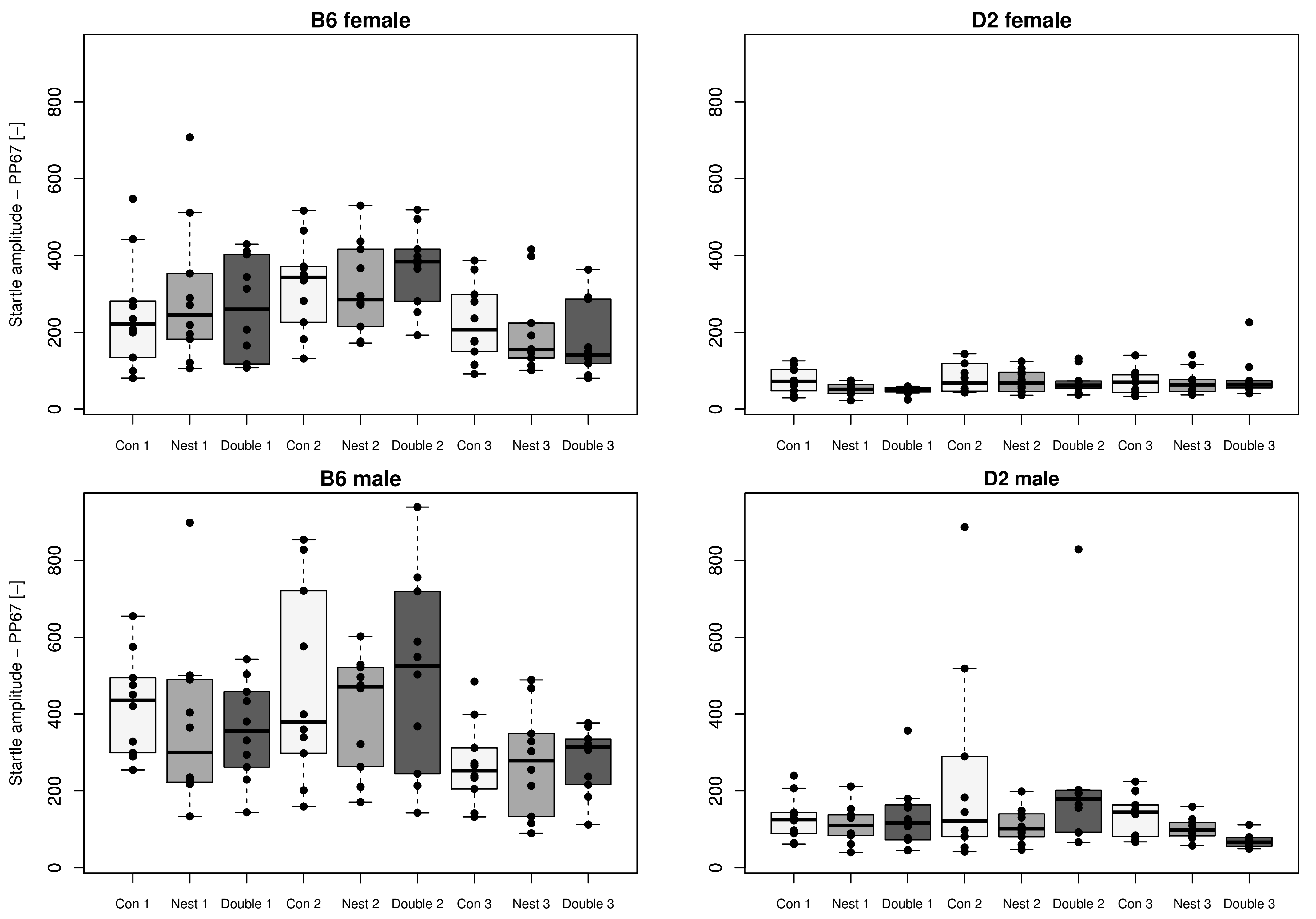

B6 female
D2 female
B6 male
D2 male
Startle amplitude – PP67 [–]
0
200
400
600
800
Con 1
Nest 1
Double 1
Con 2
Nest 2
Double 2
Con 3
Nest 3
Double 3

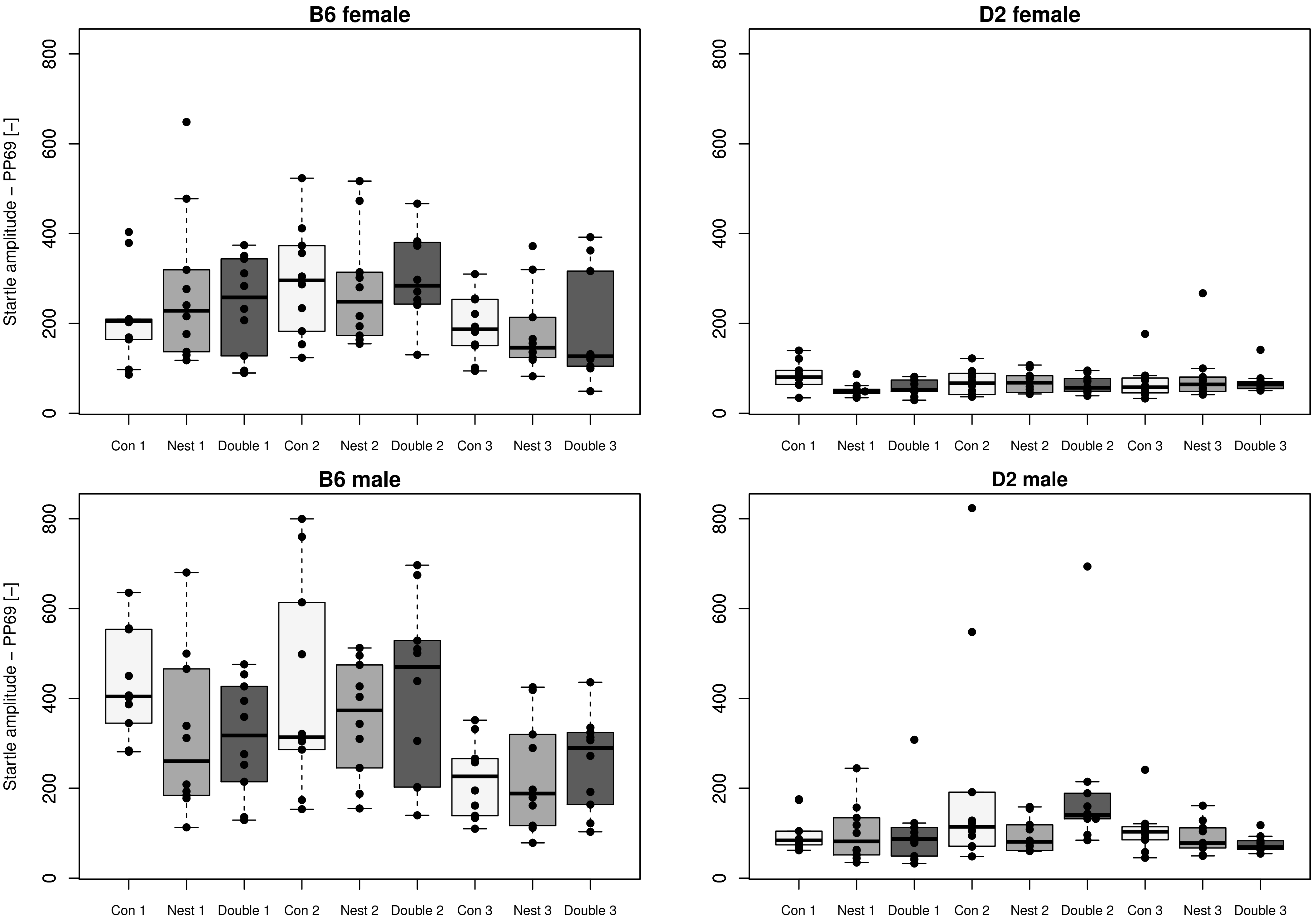
B6 female
D2 female
B6 male
D2 male
Startle amplitude – PP69 [–]
0
200
400
600
800
Con 1
Nest 1
Double 1
Con 2
Nest 2
Double 2
Con 3
Nest 3
Double 3

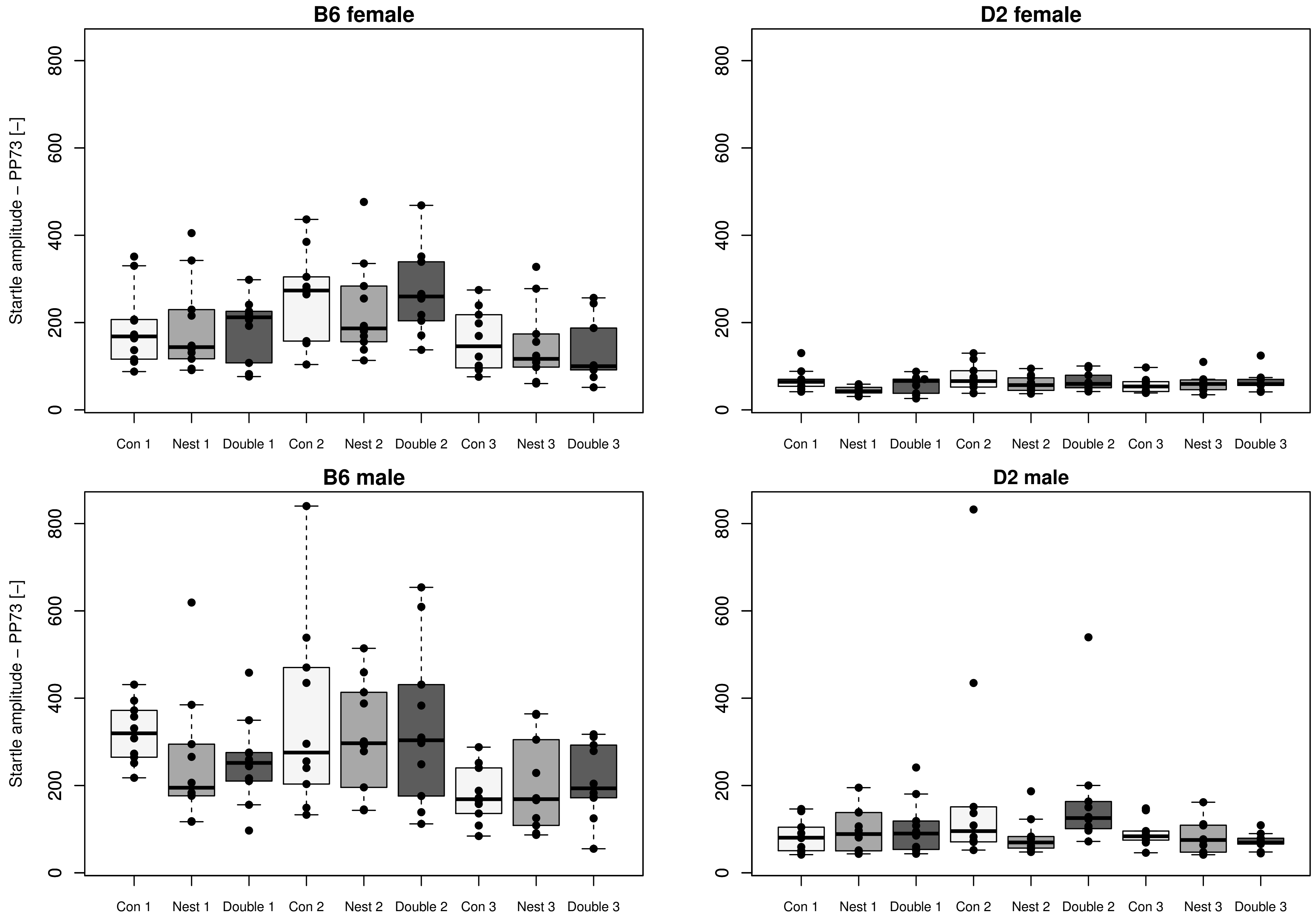

B6 female
D2 female
B6 male
D2 male
Startle amplitude – PP73 [–]
0
200
400
600
800
Con 1
Nest 1
Double 1
Con 2
Nest 2
Double 2
Con 3
Nest 3
Double 3

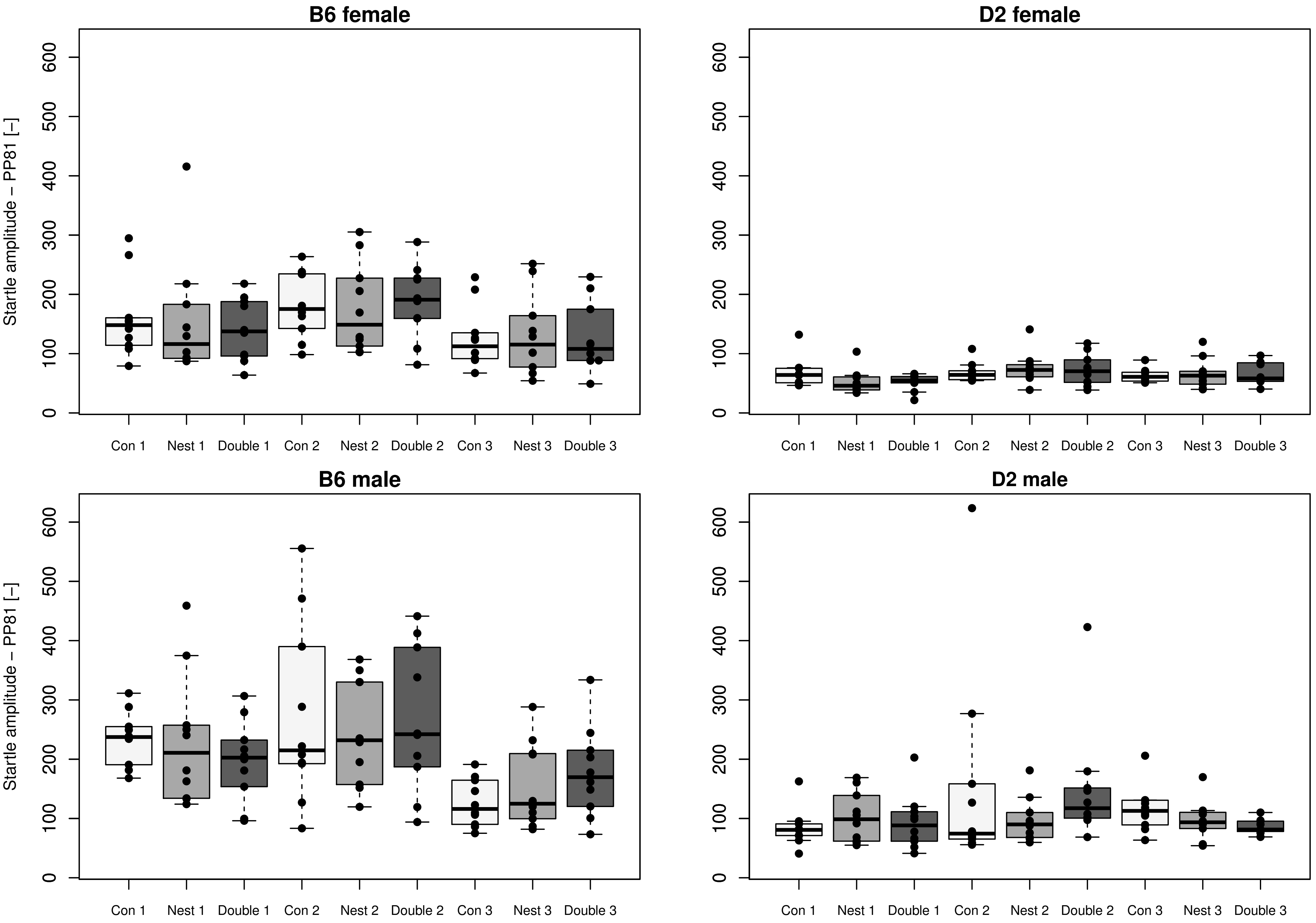
B6 female
D2 female
B6 male
D2 male
Startle amplitude – PP81 [–]
0
100
200
300
400
500
600
Con 1
Nest 1
Double 1
Con 2
Nest 2
Double 2
Con 3
Nest 3
Double 3

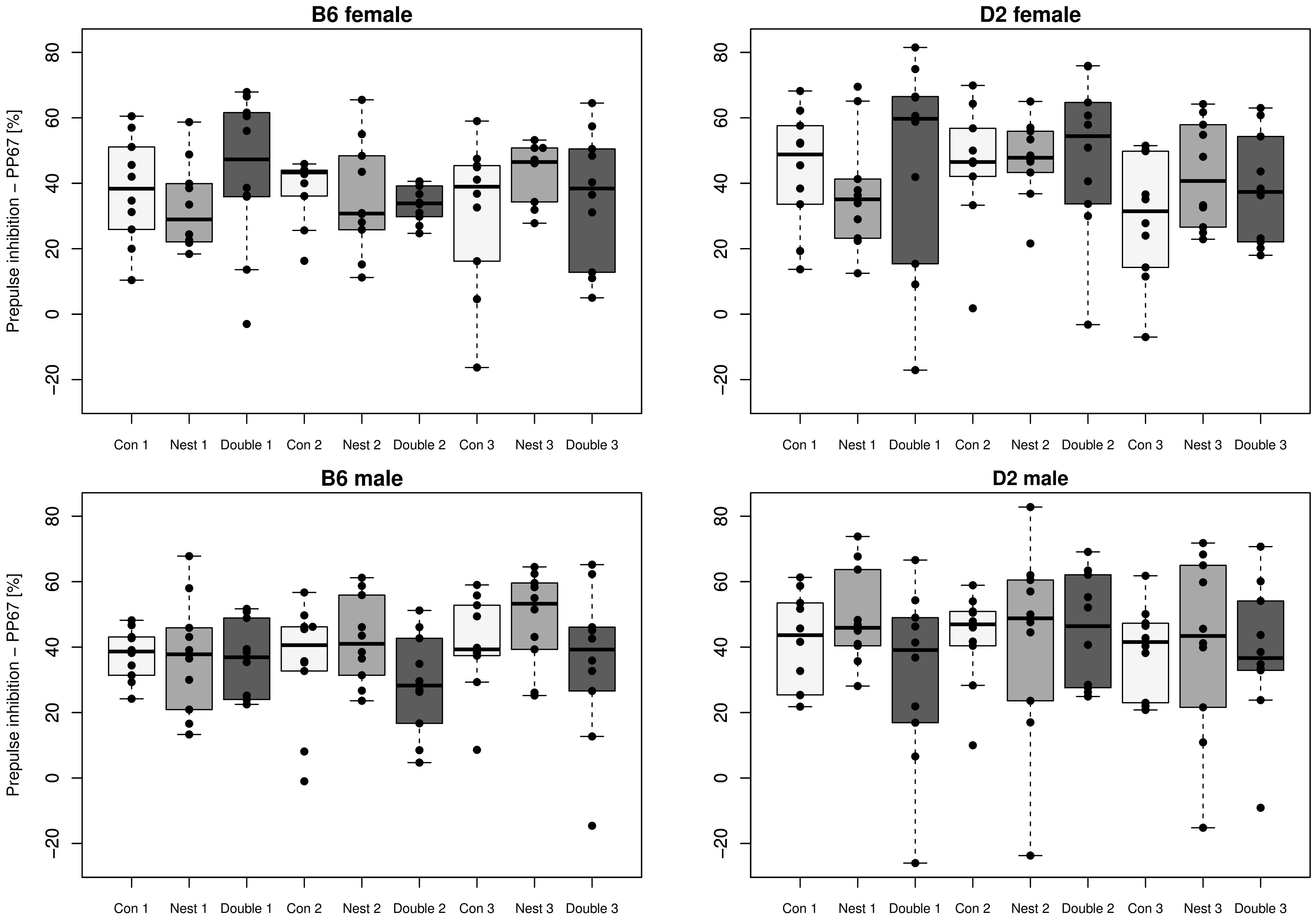
B6 female
D2 female
B6 male
D2 male
Prepulse inhibition – PP67 [%]
80
60
40
20
0
-20
Con 1
Nest 1
Double 1
Con 2
Nest 2
Double 2
Con 3
Nest 3
Double 3

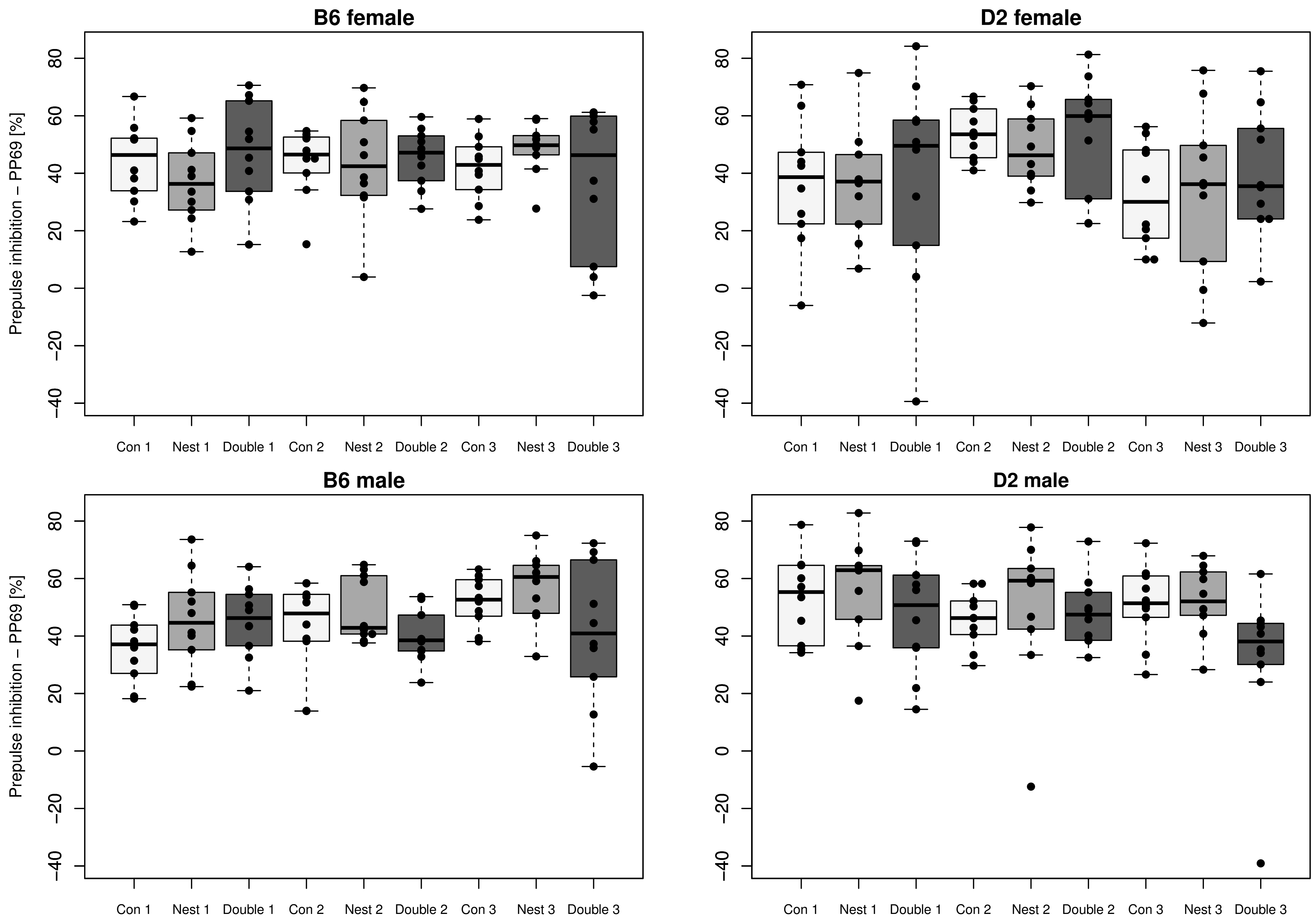
B6 female
D2 female
B6 male
D2 male
Prepulse inhibition – PP69 [%]
80
60
40
20
0
−20
−40
Con 1
Nest 1
Double 1
Con 2
Nest 2
Double 2
Con 3
Nest 3
Double 3

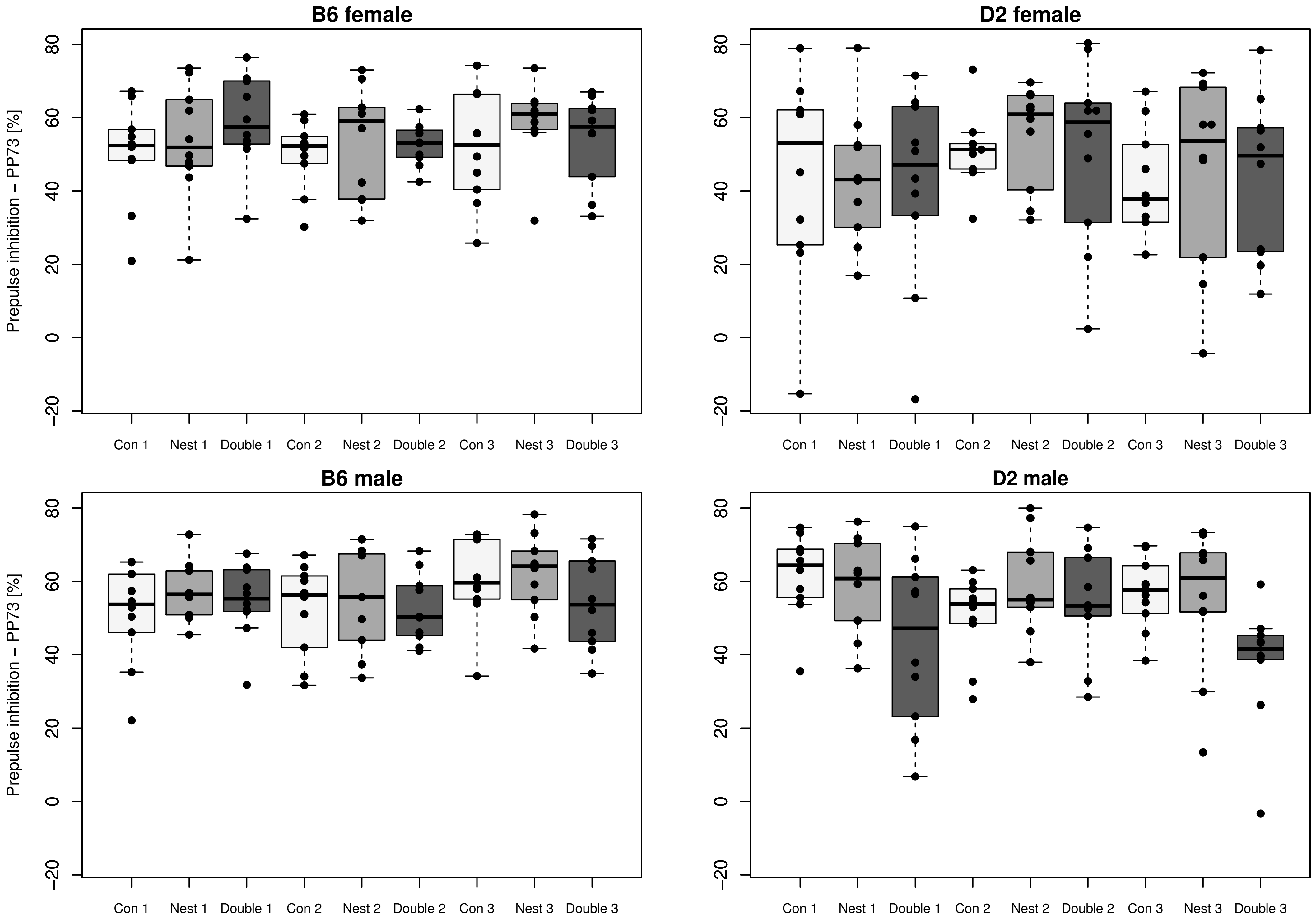
B6 female
D2 female
B6 male
D2 male
Prepulse inhibition − PP73 [%]
80
60
40
20
0
−20
Con 1
Nest 1
Double 1
Con 2
Nest 2
Double 2
Con 3
Nest 3
Double 3

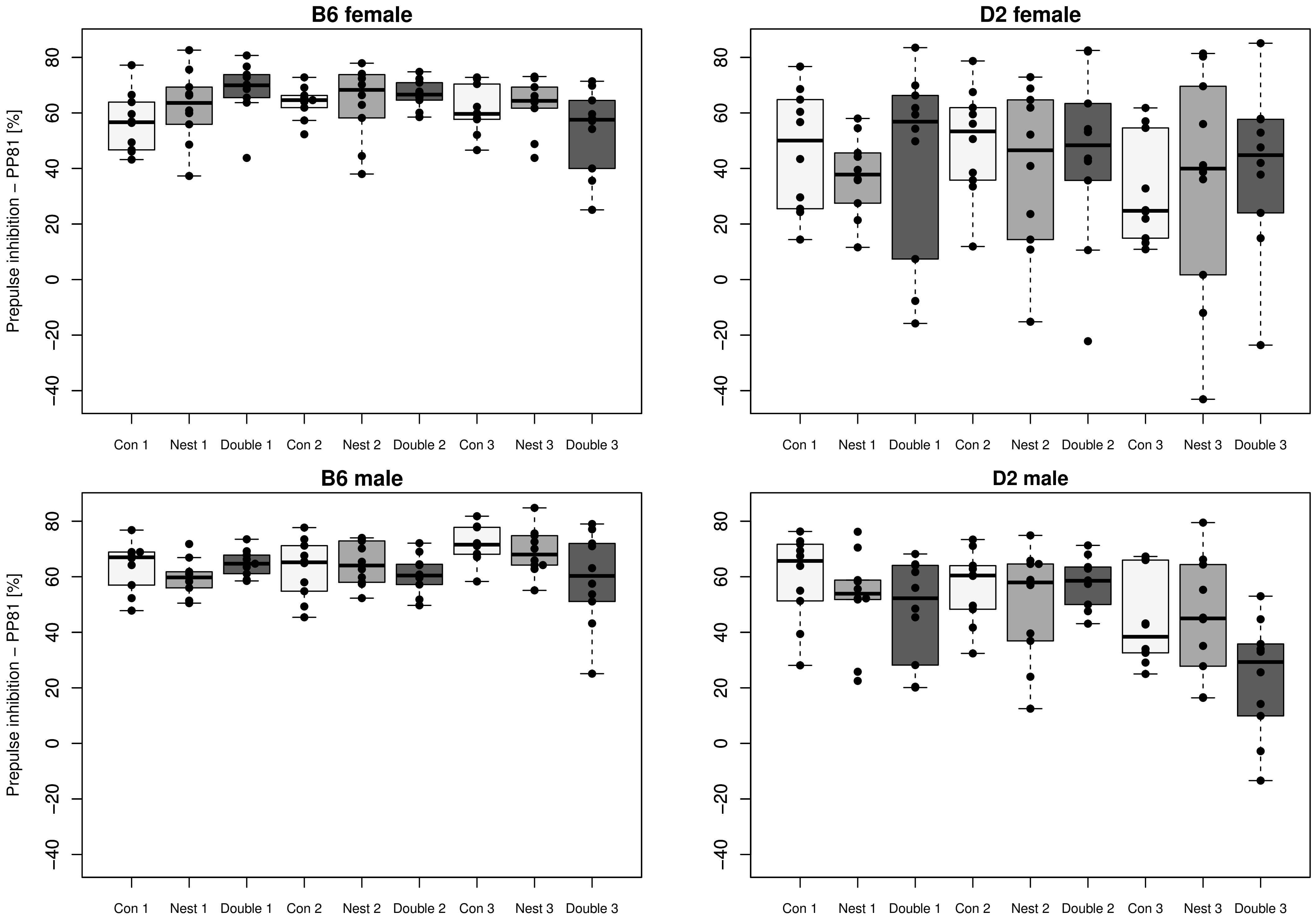
B6 female
D2 female
B6 male
D2 male
Prepulse inhibition – PP81 [%]
-40
-20
0
20
40
60
80
Con 1
Nest 1
Double 1
Con 2
Nest 2
Double 2
Con 3
Nest 3
Double 3

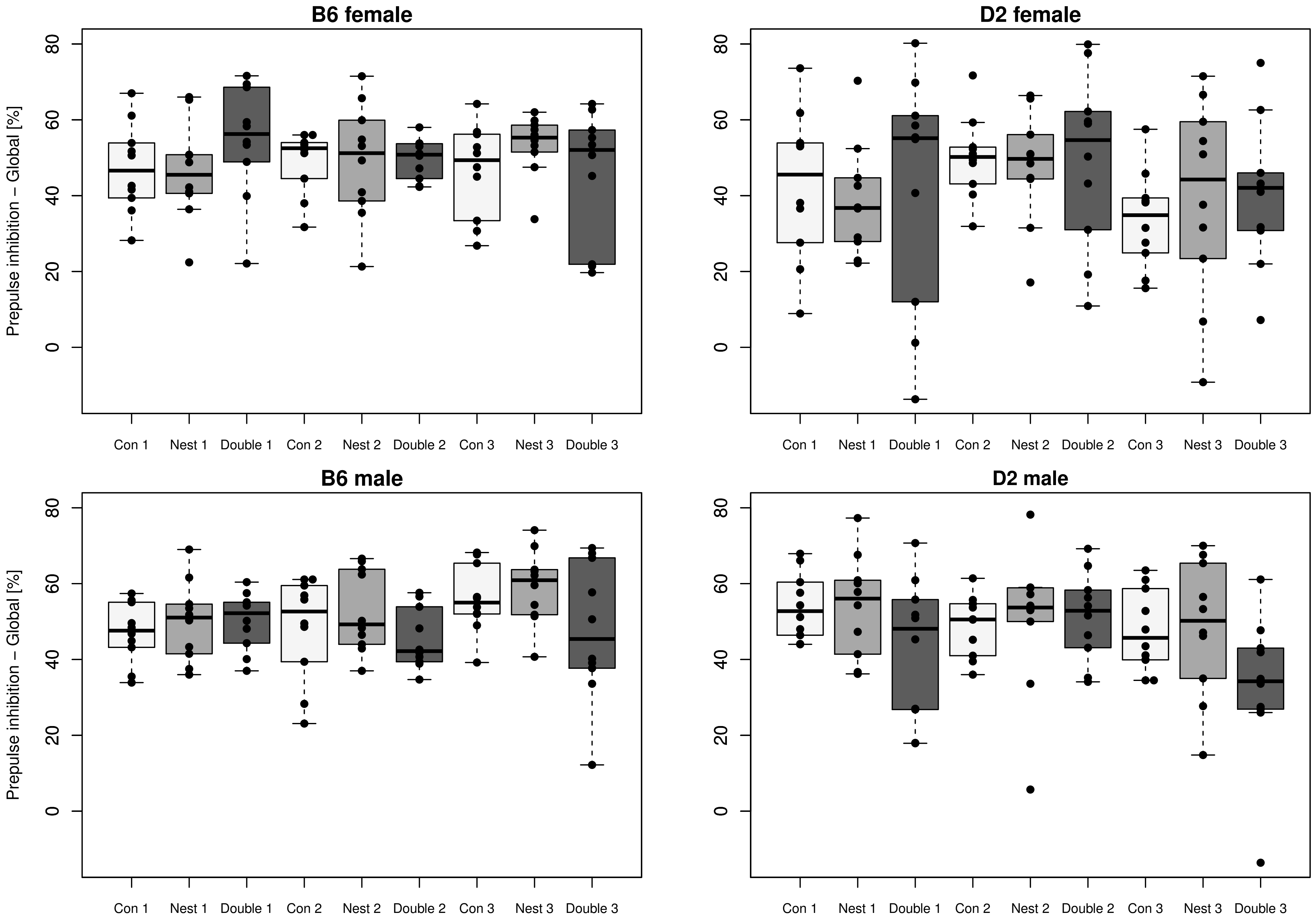

B6 female
D2 female
B6 male
D2 male
Prepulse inhibition – Global [%]
80
60
40
20
0
Con 1
Nest 1
Double 1
Con 2
Nest 2
Double 2
Con 3
Nest 3
Double 3

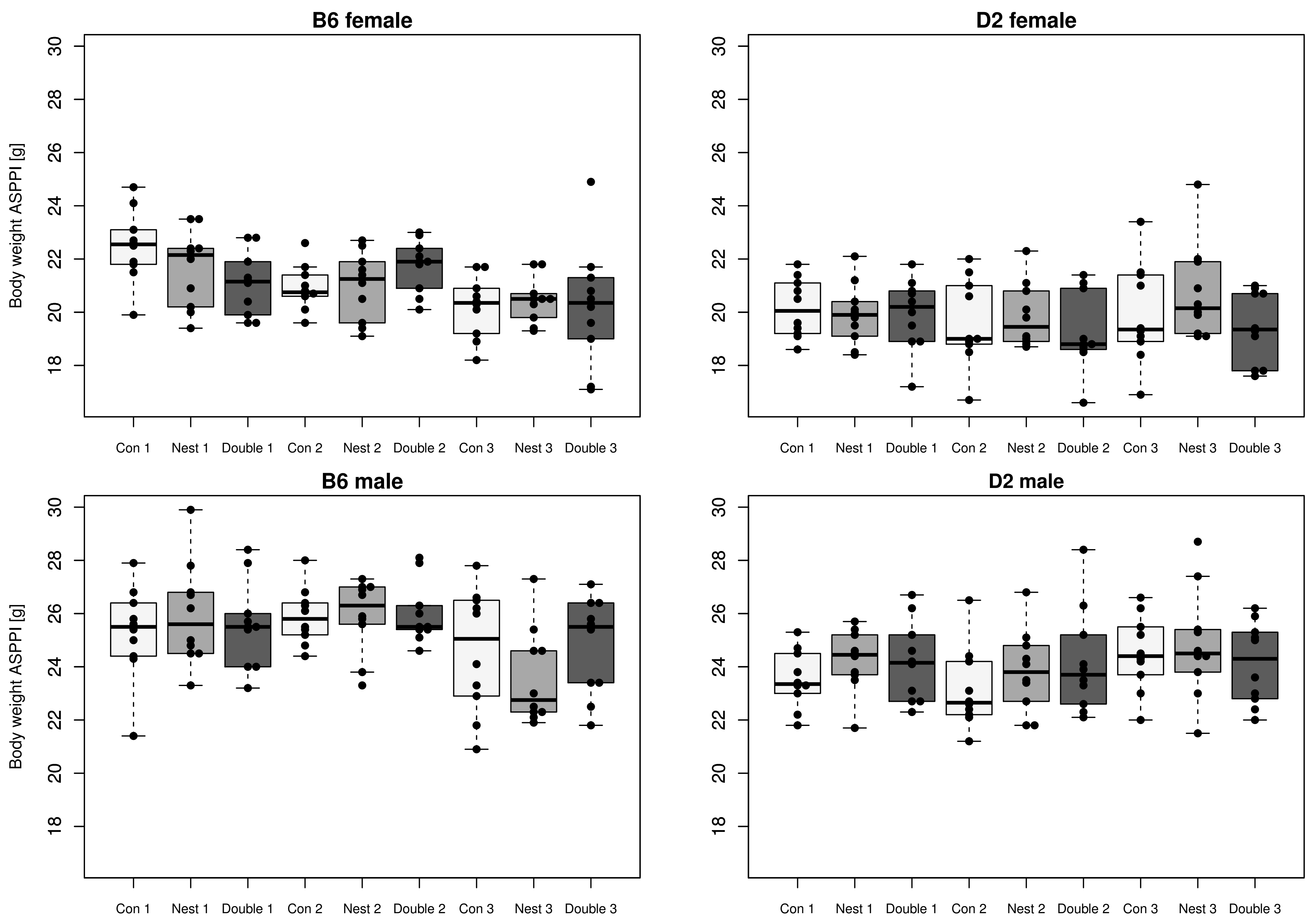
B6 female
D2 female
B6 male
D2 male
Body weight ASPPI [g]
30
28
26
24
22
20
18
Con 1
Nest 1
Double 1
Con 2
Nest 2
Double 2
Con 3
Nest 3
Double 3

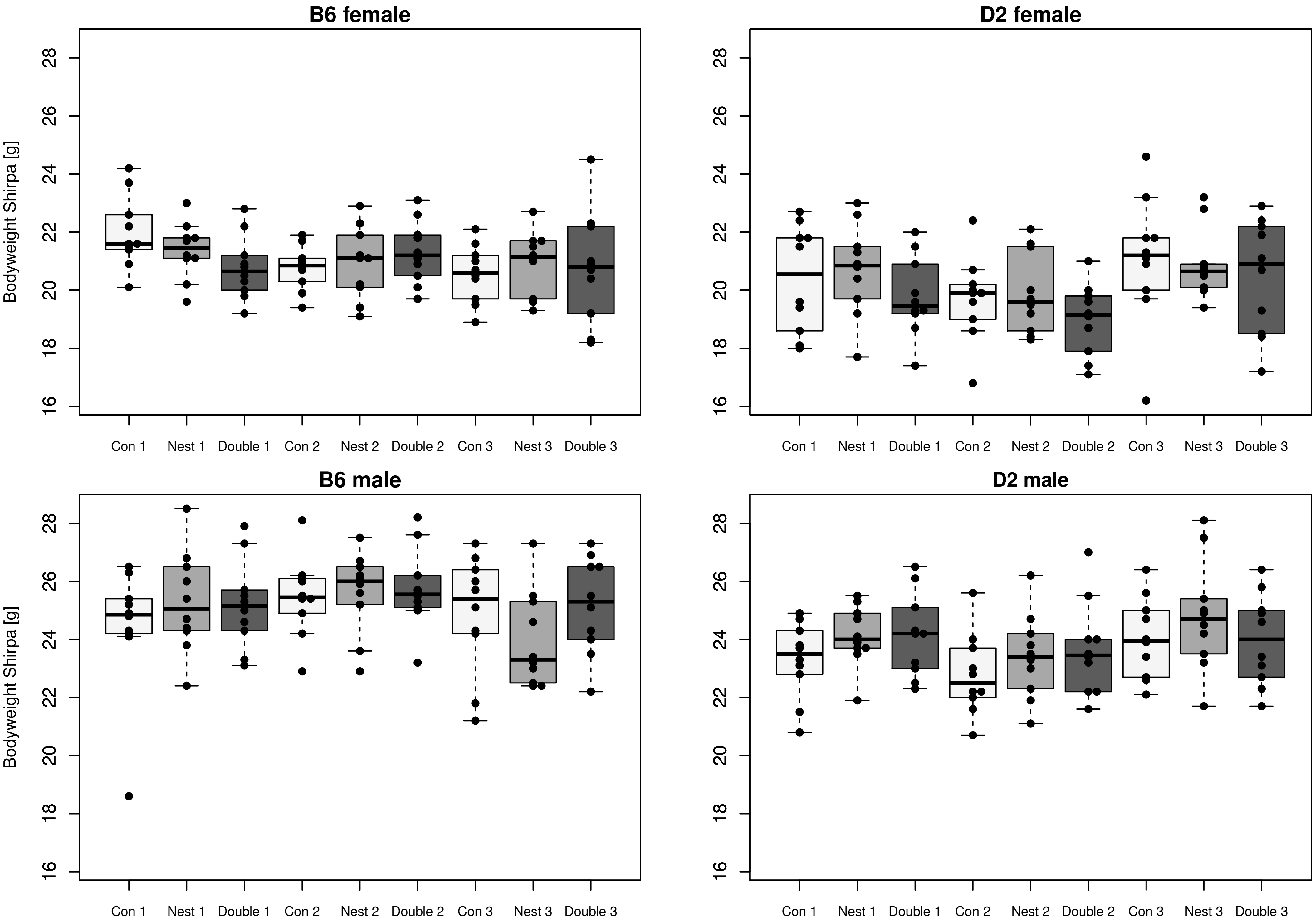

B6 female
D2 female
B6 male
D2 male
Bodyweight Shirpa [g]
16
18
20
22
24
26
28
Con 1
Nest 1
Double 1
Con 2
Nest 2
Double 2
Con 3
Nest 3
Double 3

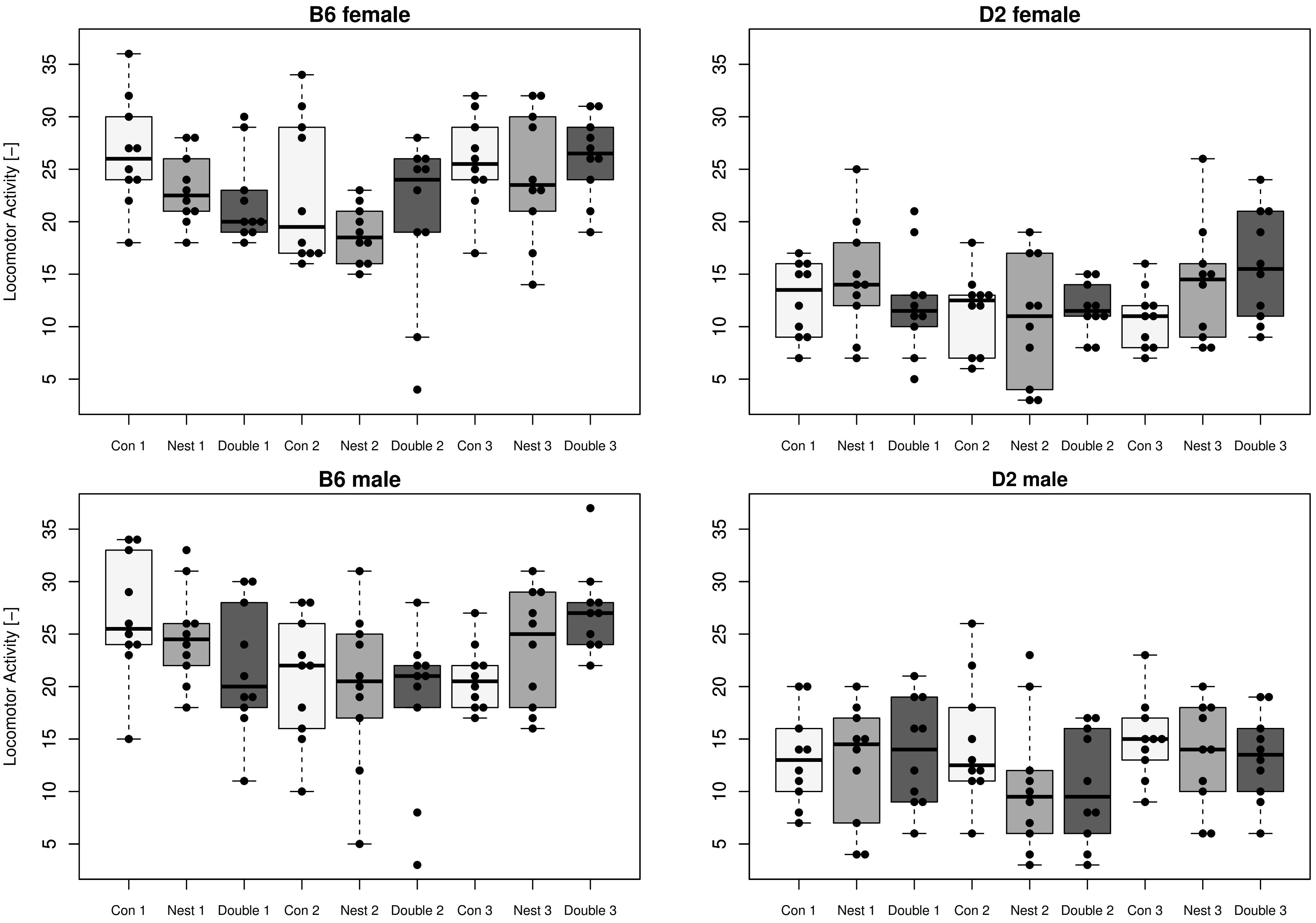

B6 female
D2 female
B6 male
D2 male
Locomotor Activity [–]
5
10
15
20
25
30
35
Con 1
Nest 1
Double 1
Con 2
Nest 2
Double 2
Con 3
Nest 3
Double 3

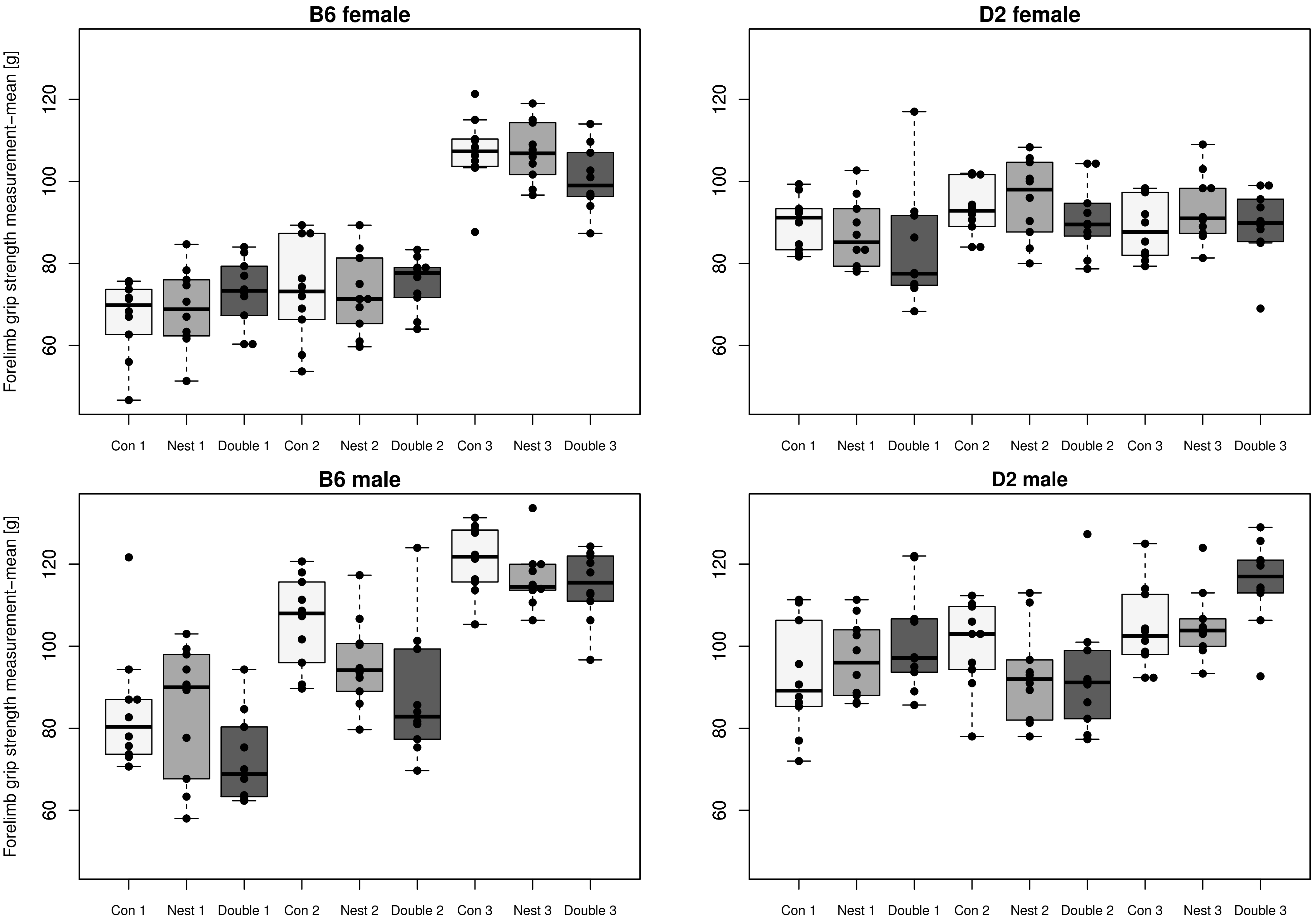

B6 female
D2 female
B6 male
D2 male
Forelimb grip strength measurement−mean [g]
60
80
100
120
Con 1
Nest 1
Double 1
Con 2
Nest 2
Double 2
Con 3
Nest 3
Double 3

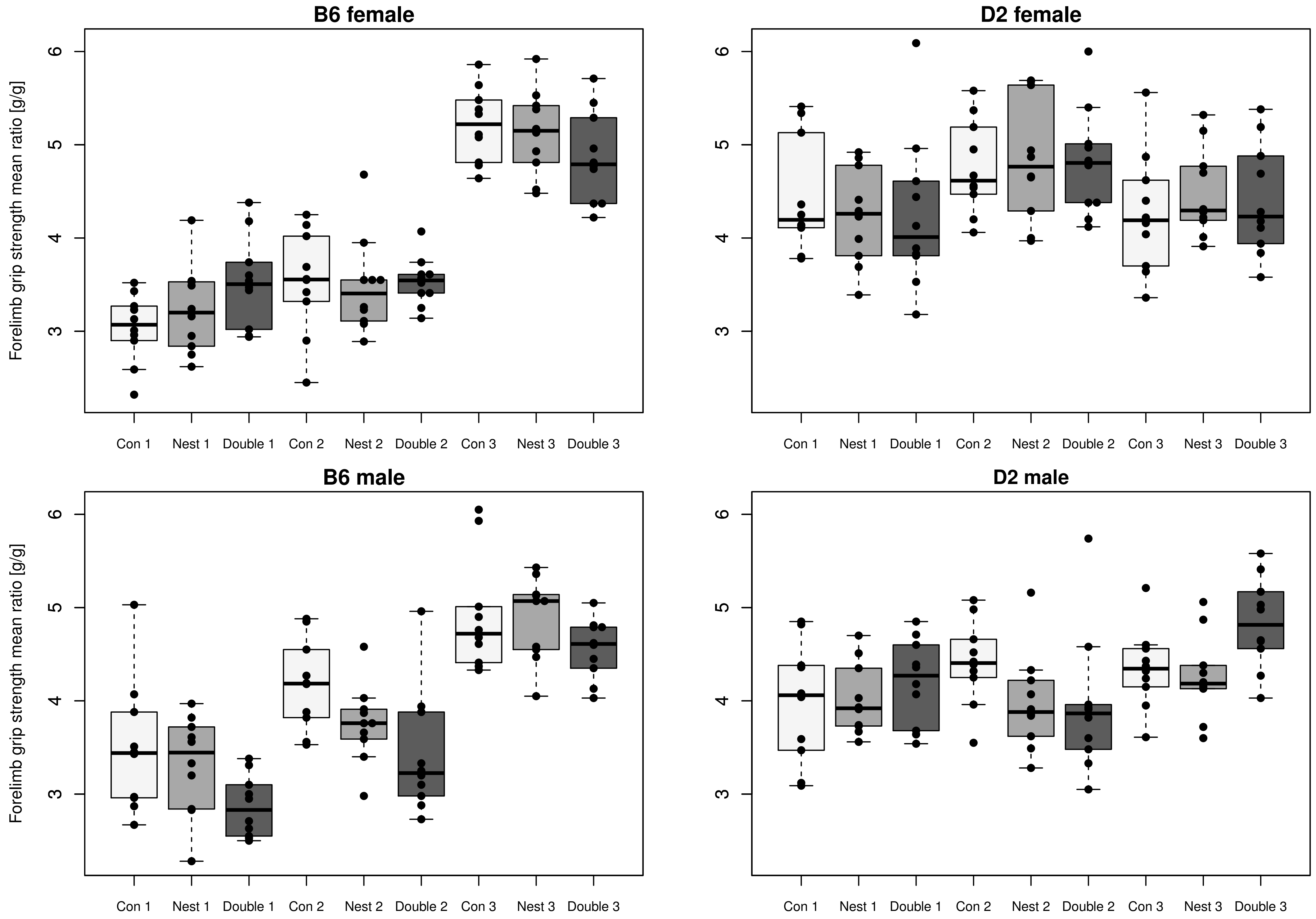

B6 female
D2 female
B6 male
D2 male
Forelimb grip strength mean ratio [g/g]
Con 1
Nest 1
Double 1
Con 2
Nest 2
Double 2
Con 3
Nest 3
Double 3

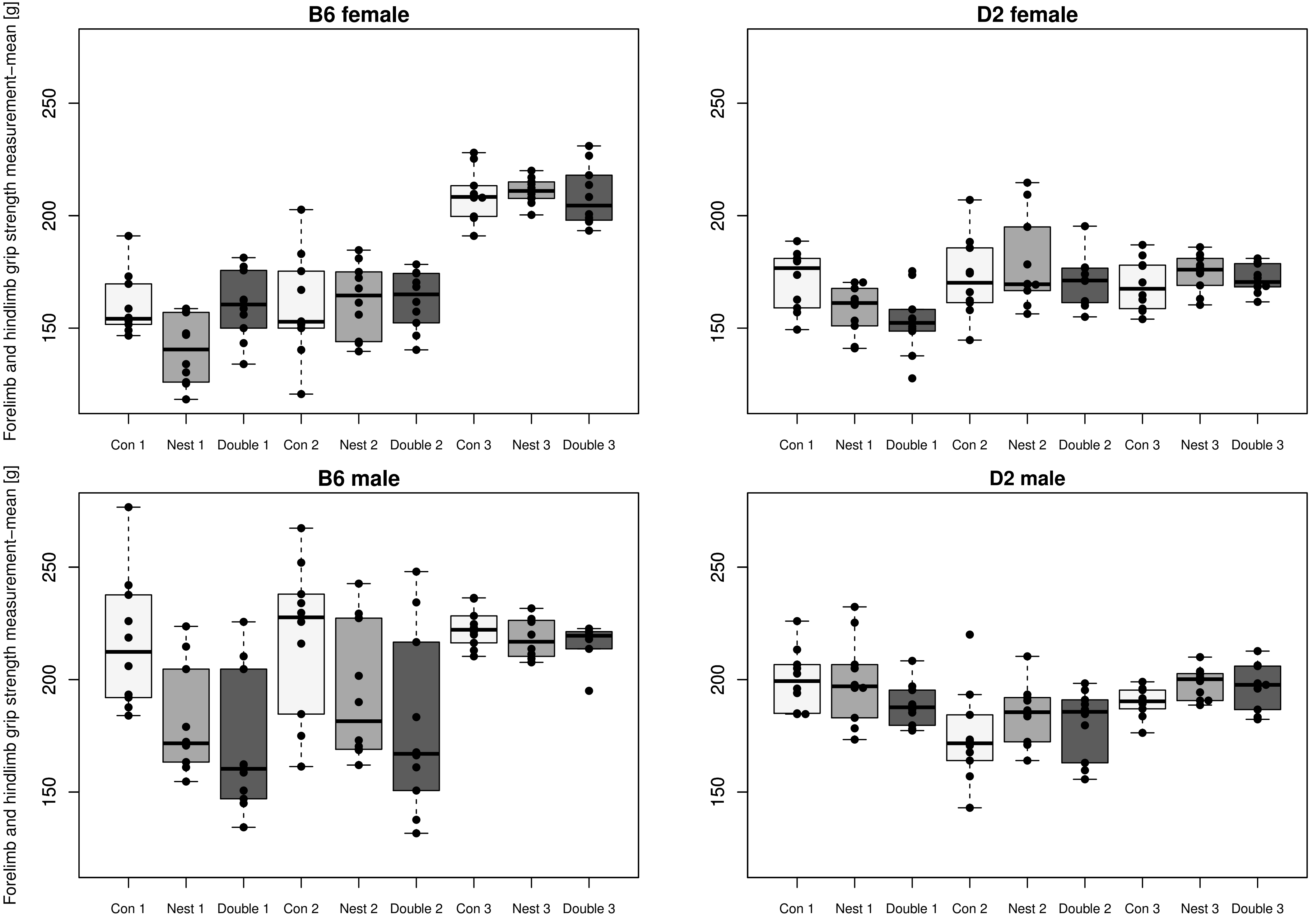
B6 female
D2 female
B6 male
D2 male
Forelimb and hindlimb grip strength measurement−mean [g]
Forelimb and hindlimb grip strength measurement−mean [g]
150
200
250
Con 1
Nest 1
Double 1
Con 2
Nest 2
Double 2
Con 3
Nest 3
Double 3

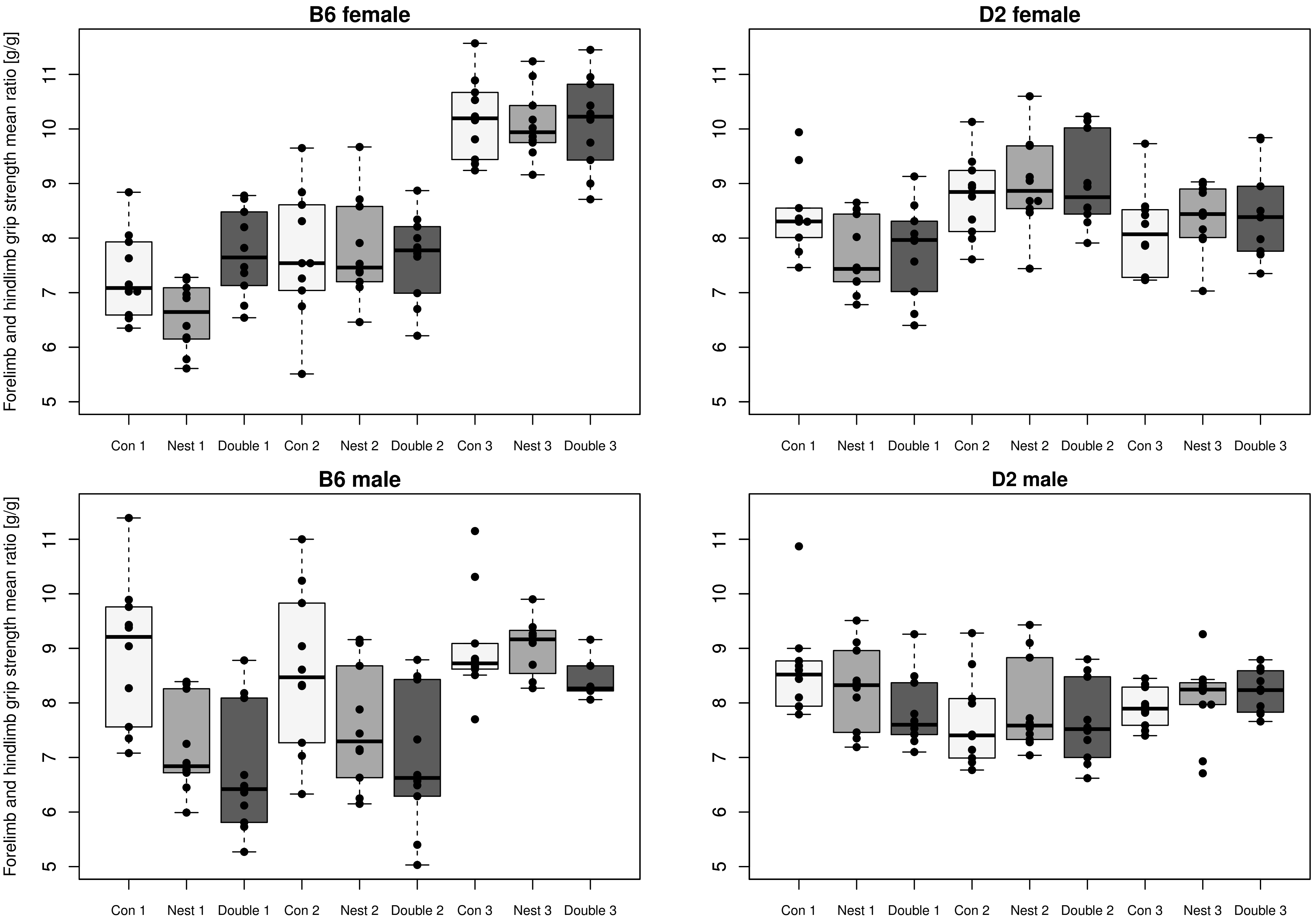

B6 female
D2 female
B6 male
D2 male
Forelimb and hindlimb grip strength mean ratio [g/g]
Con 1
Nest 1
Double 1
Con 2
Nest 2
Double 2
Con 3
Nest 3
Double 3
5
6
7
8
9
10
11

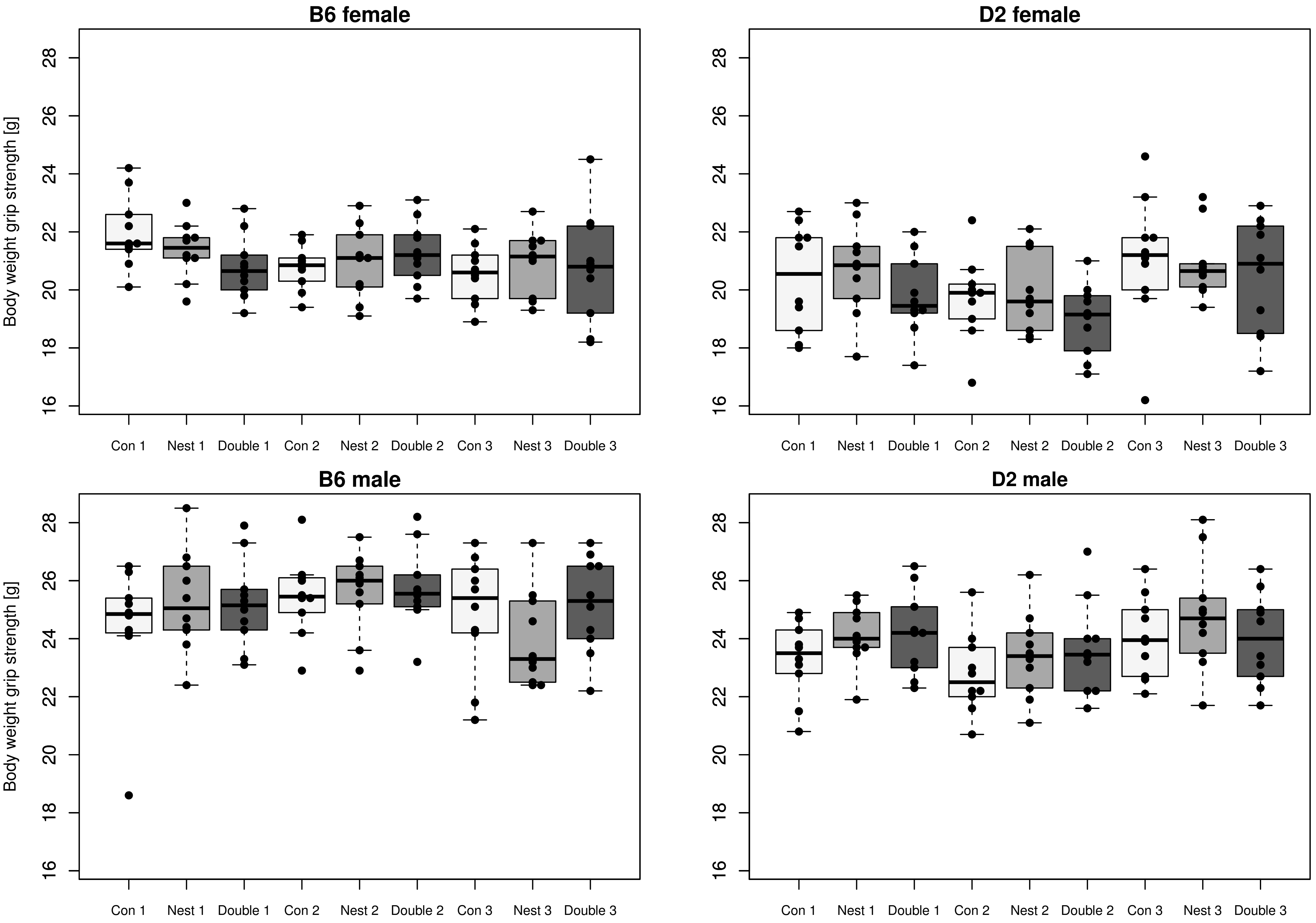

B6 female
D2 female
B6 male
D2 male
Body weight grip strength [g]
16
18
20
22
24
26
28
Con 1
Nest 1
Double 1
Con 2
Nest 2
Double 2
Con 3
Nest 3
Double 3

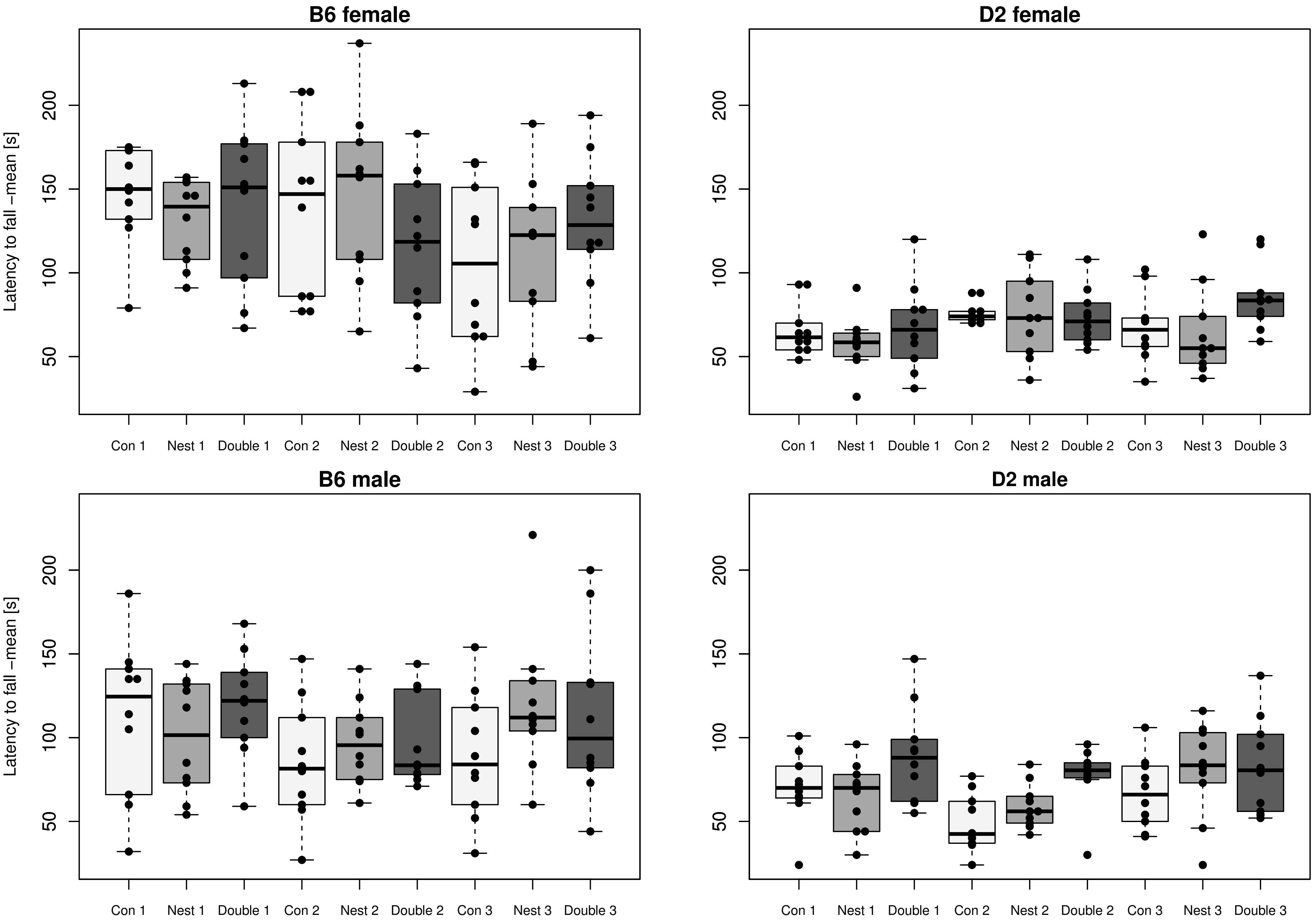

B6 female
D2 female
B6 male
D2 male
Latency to fall –mean [s]
50
100
150
200
Con 1
Nest 1
Double 1
Con 2
Nest 2
Double 2
Con 3
Nest 3
Double 3

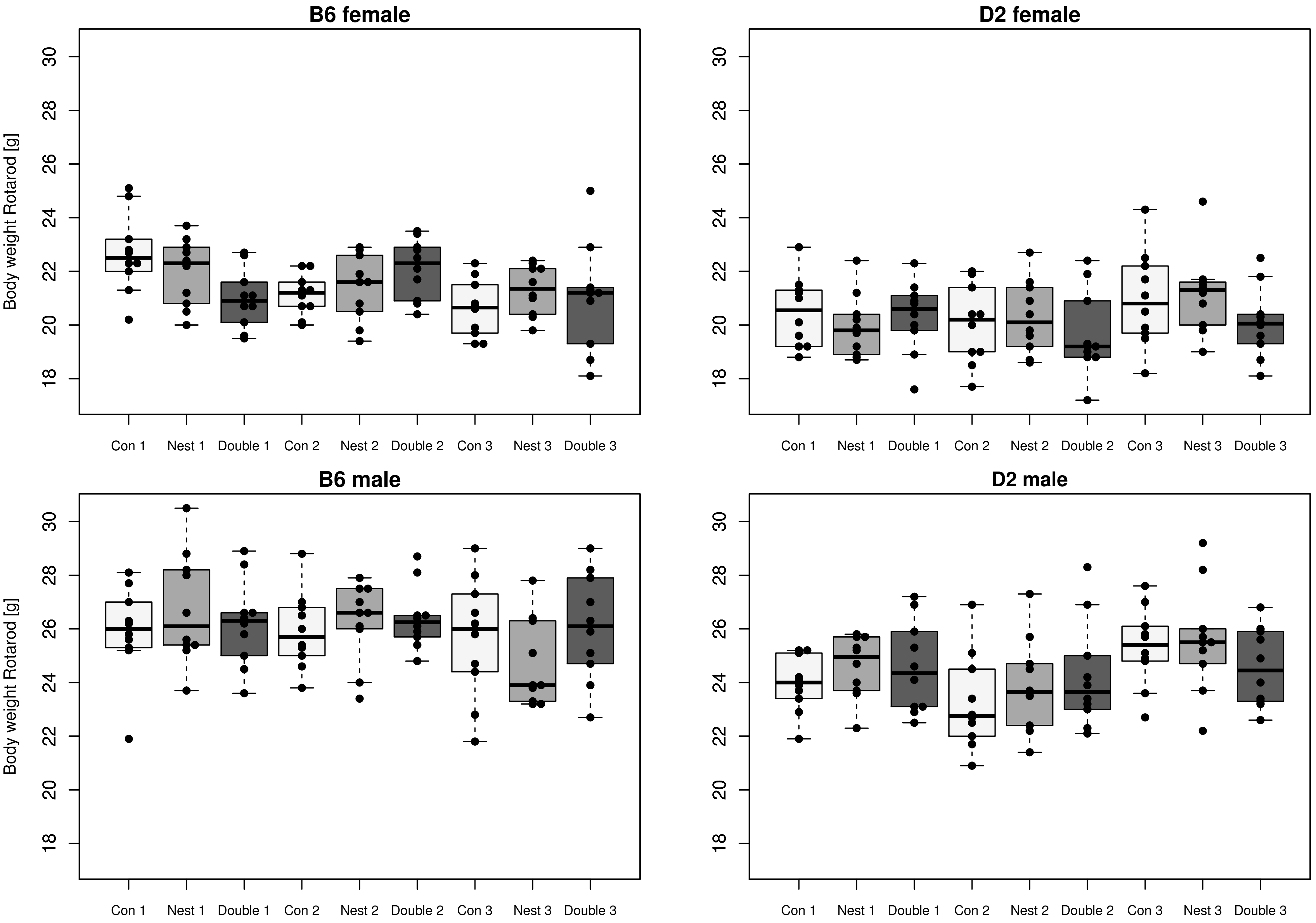

B6 female
D2 female
B6 male
D2 male
Body weight Rotarod [g]
18
20
22
24
26
28
30
Con 1
Nest 1
Double 1
Con 2
Nest 2
Double 2
Con 3
Nest 3
Double 3

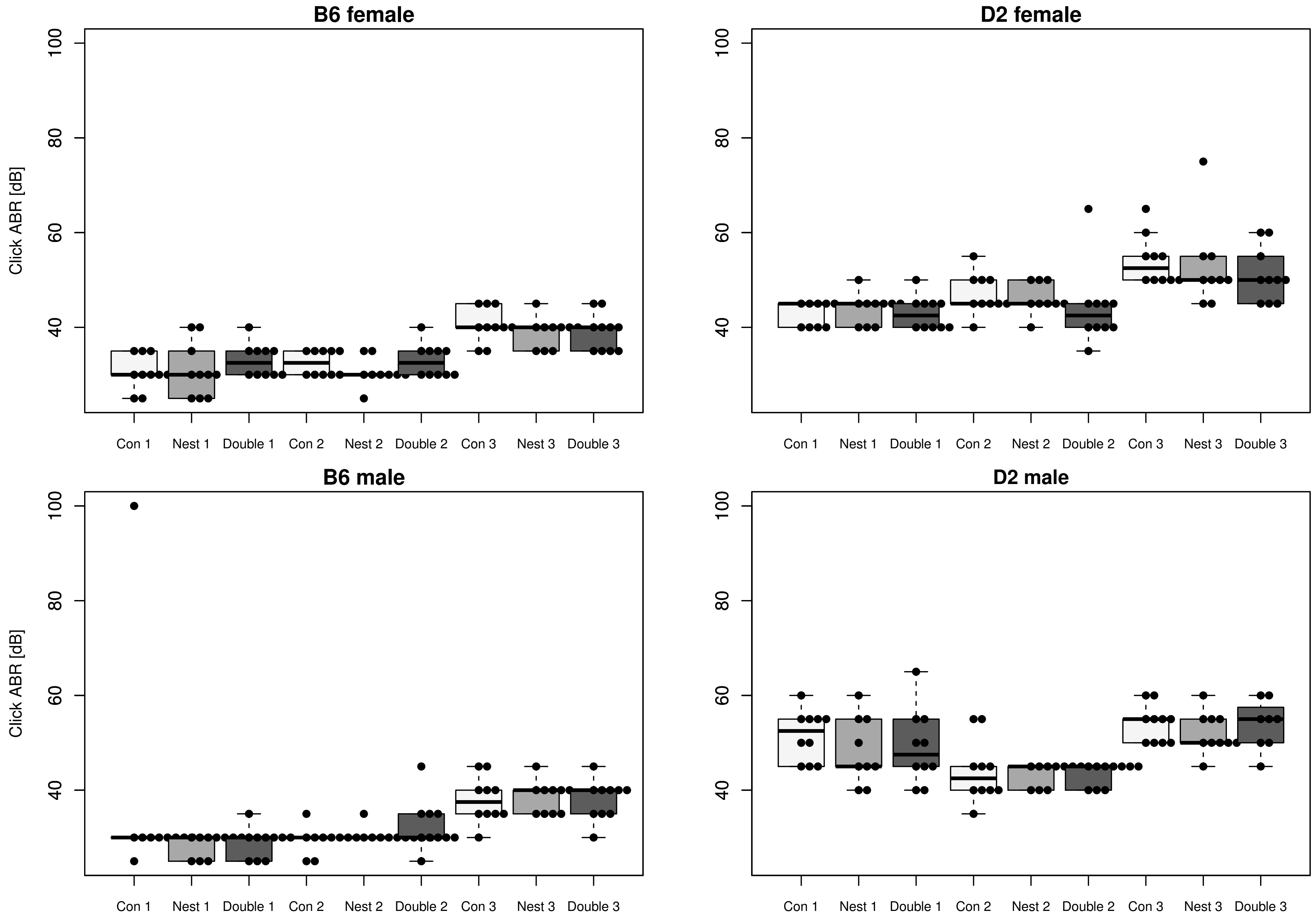
B6 female
D2 female
B6 male
D2 male
Click ABR [dB]
100
80
60
40
Con 1
Nest 1
Double 1
Con 2
Nest 2
Double 2
Con 3
Nest 3
Double 3

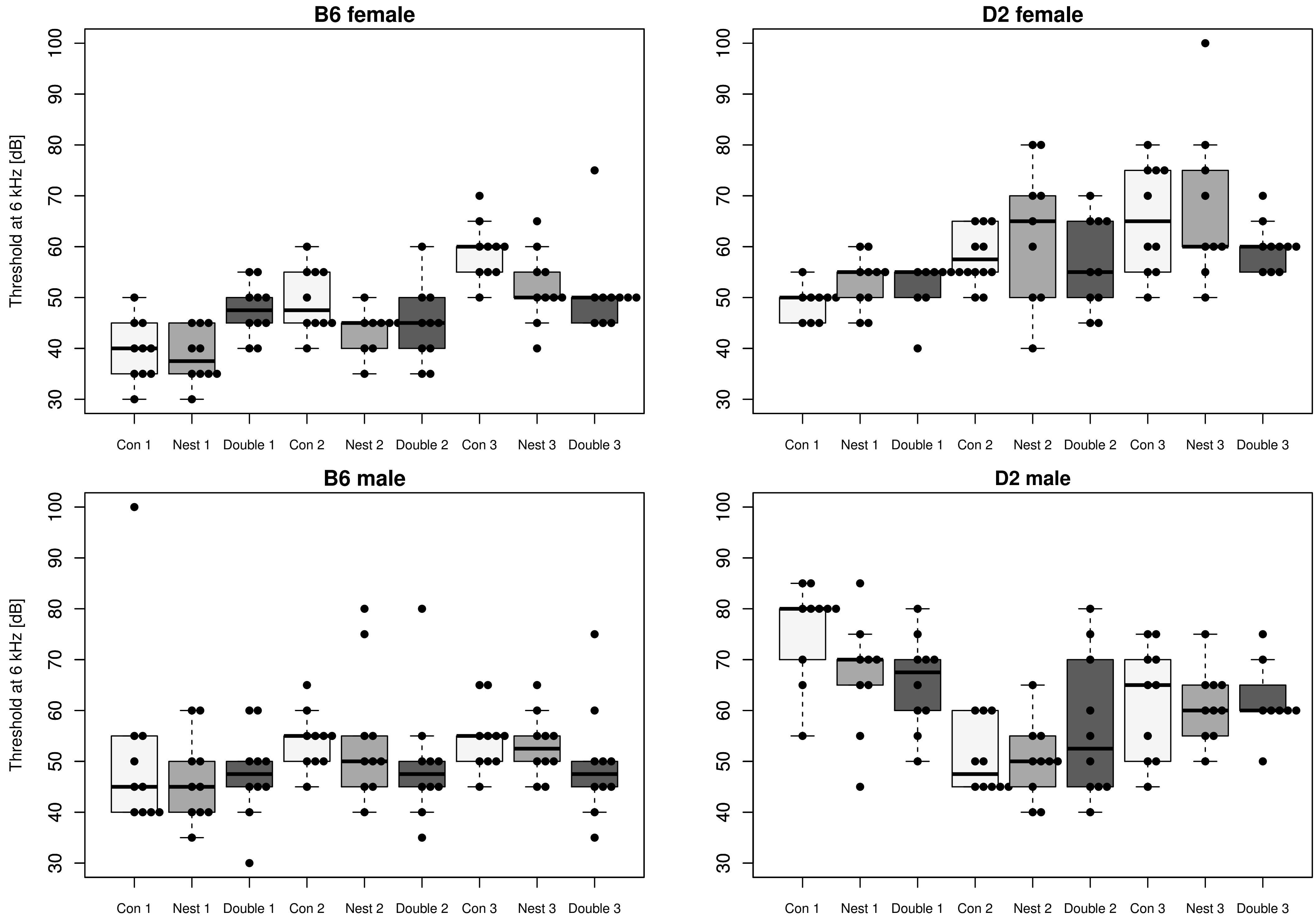

B6 female
D2 female
B6 male
D2 male
Threshold at 6 kHz [dB]
100
90
80
70
60
50
40
30
Con 1
Nest 1
Double 1
Con 2
Nest 2
Double 2
Con 3
Nest 3
Double 3

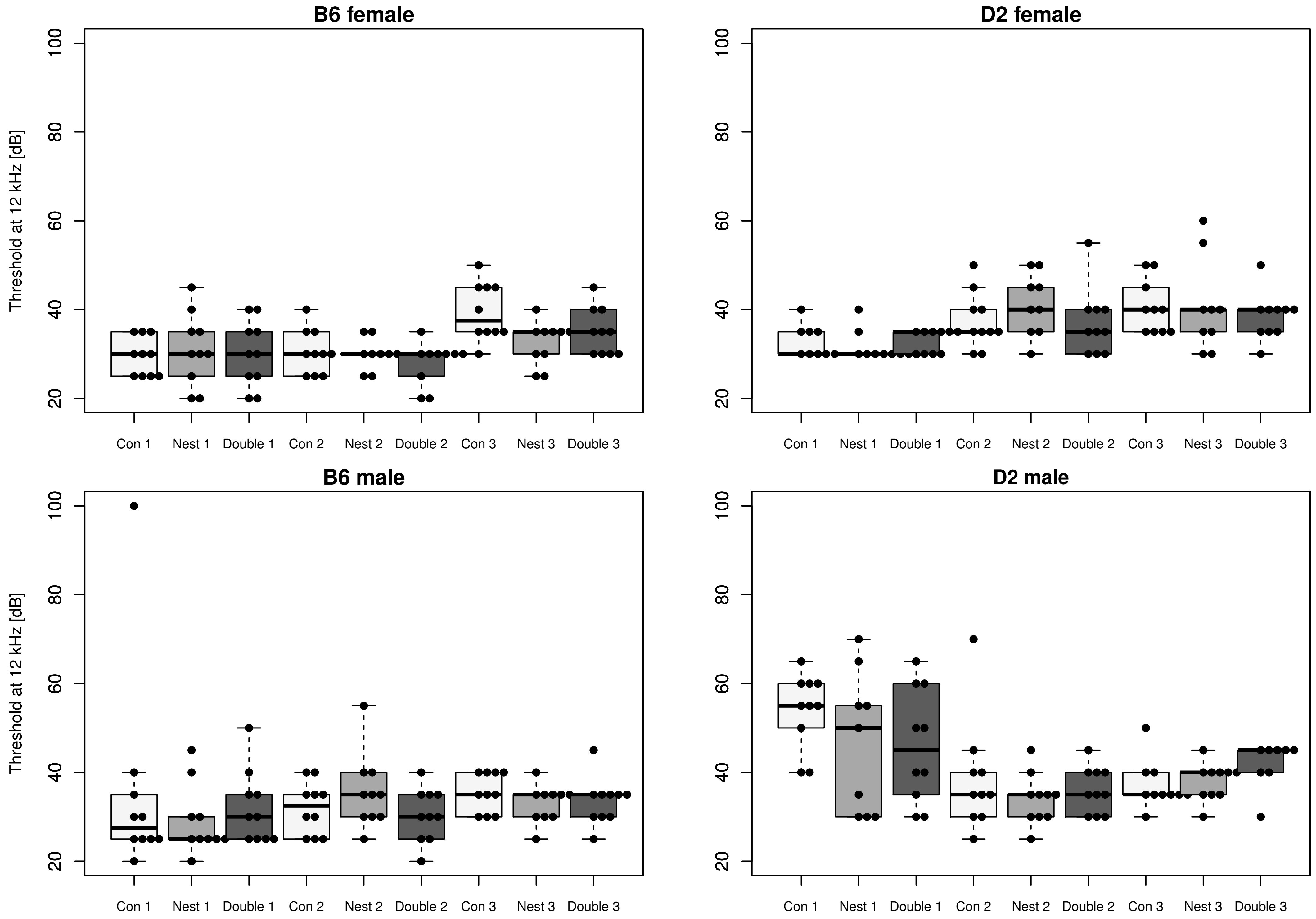

B6 female
D2 female
B6 male
D2 male
Threshold at 12 kHz [dB]
20
40
60
80
100
Con 1
Nest 1
Double 1
Con 2
Nest 2
Double 2
Con 3
Nest 3
Double 3

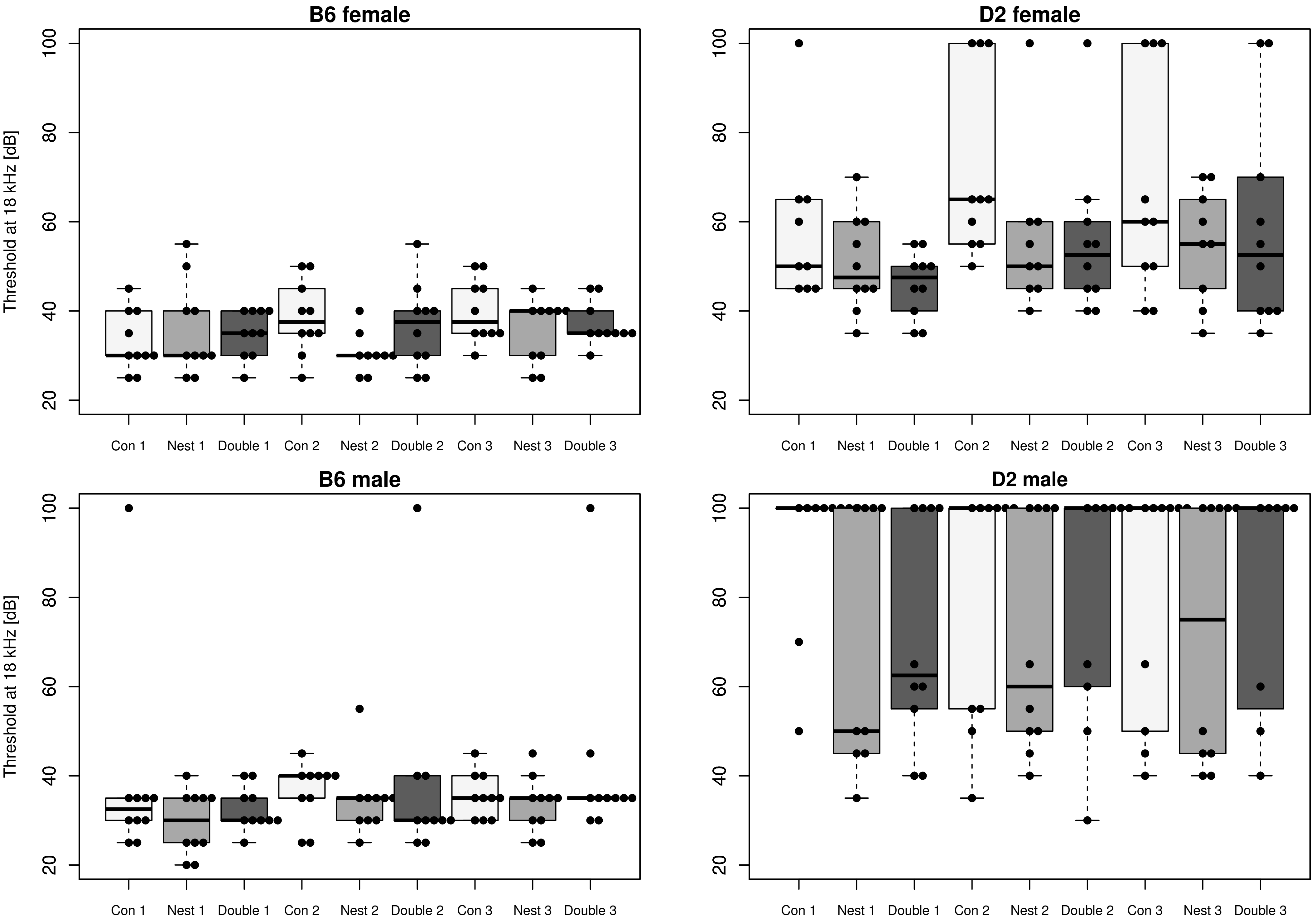

B6 female
D2 female
B6 male
D2 male
Threshold at 18 kHz [dB]
20
40
60
80
100
Con 1
Nest 1
Double 1
Con 2
Nest 2
Double 2
Con 3
Nest 3
Double 3

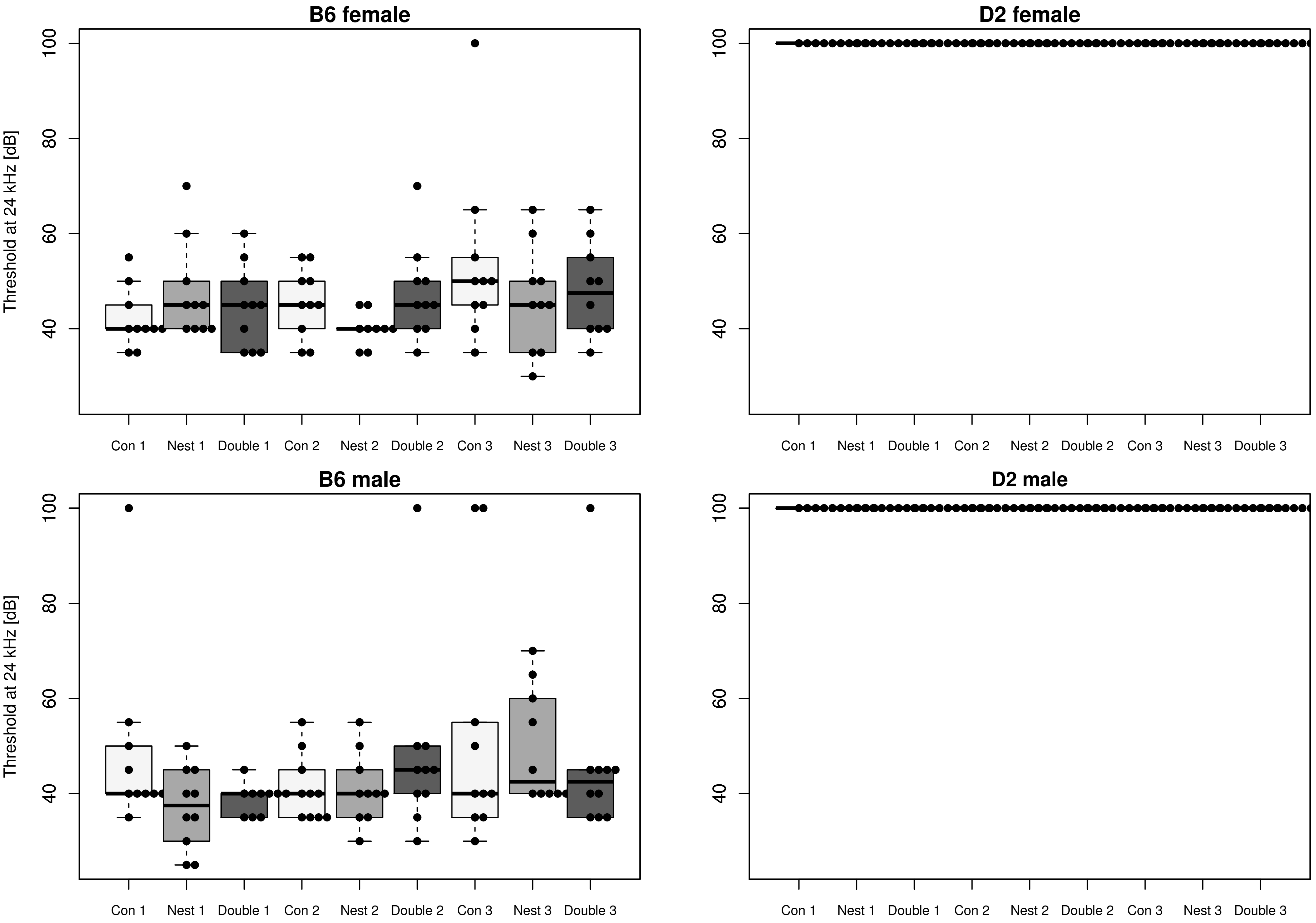
B6 female
D2 female
B6 male
D2 male
Threshold at 24 kHz [dB]
100
80
60
40
Con 1
Nest 1
Double 1
Con 2
Nest 2
Double 2
Con 3
Nest 3
Double 3

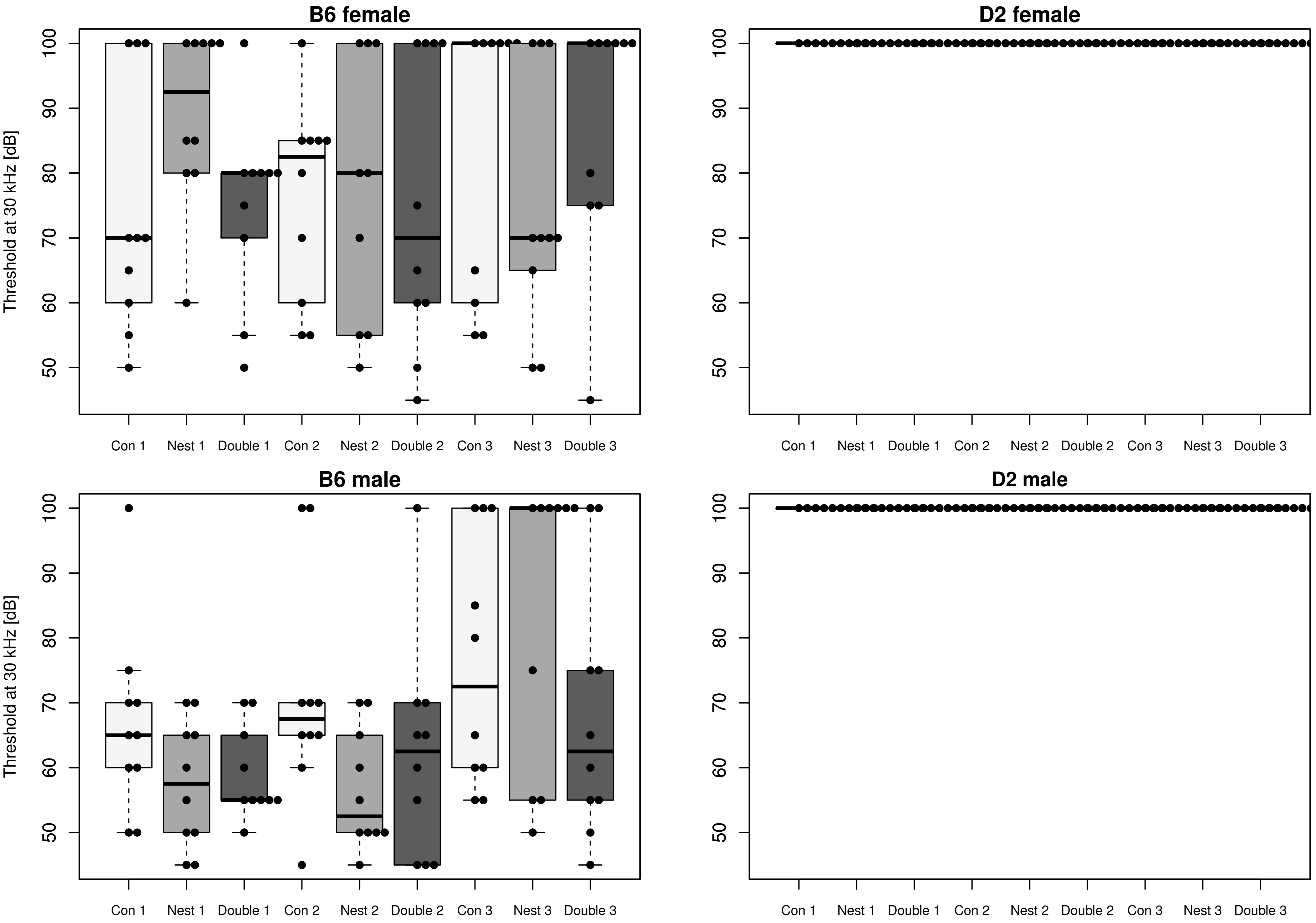

B6 female
D2 female
B6 male
D2 male
Threshold at 30 kHz [dB]
100
90
80
70
60
50
Con 1
Nest 1
Double 1
Con 2
Nest 2
Double 2
Con 3
Nest 3
Double 3

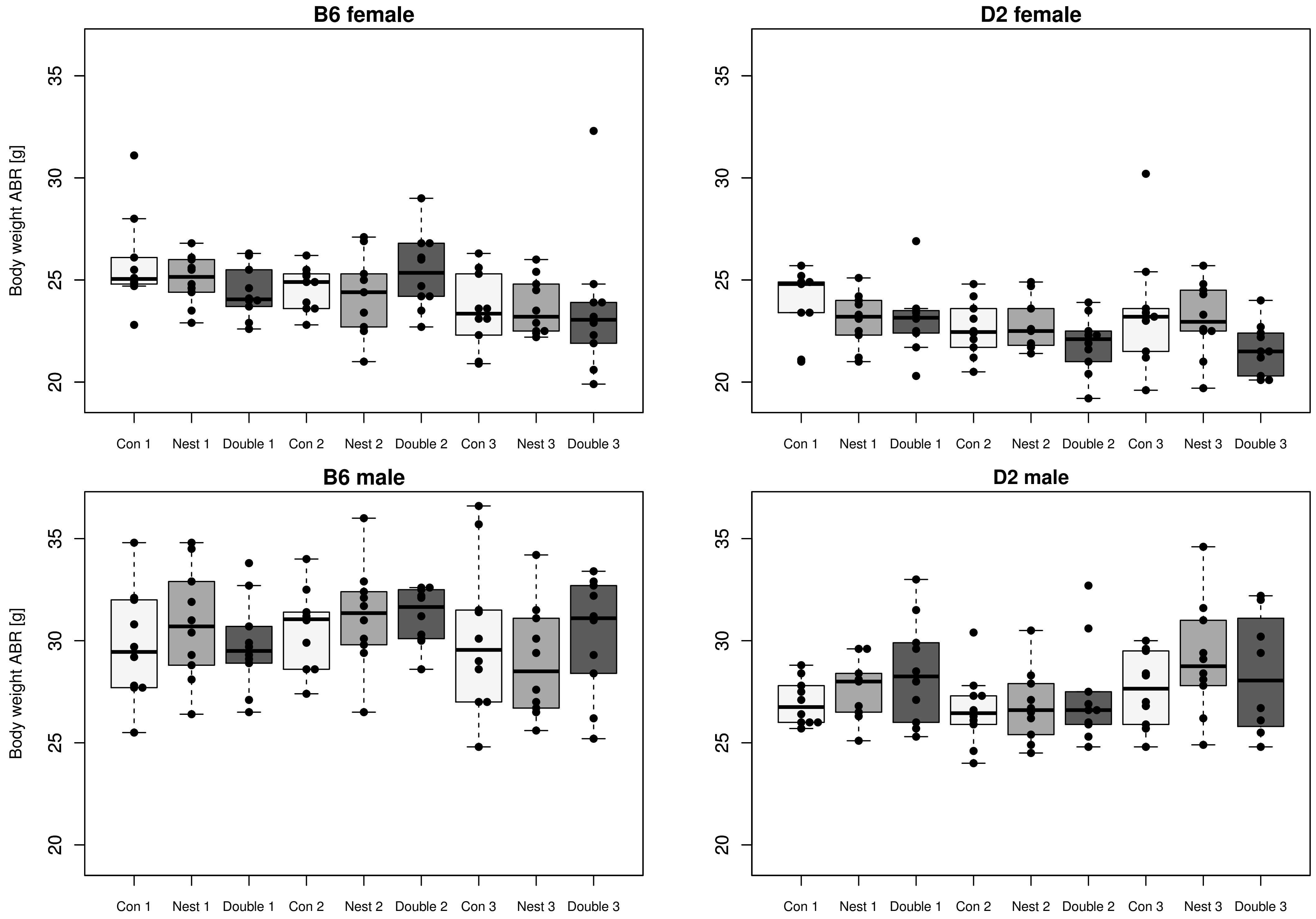

B6 female
D2 female
B6 male
D2 male
Body weight ABR [g]
20
25
30
35
Con 1
Nest 1
Double 1
Con 2
Nest 2
Double 2
Con 3
Nest 3
Double 3

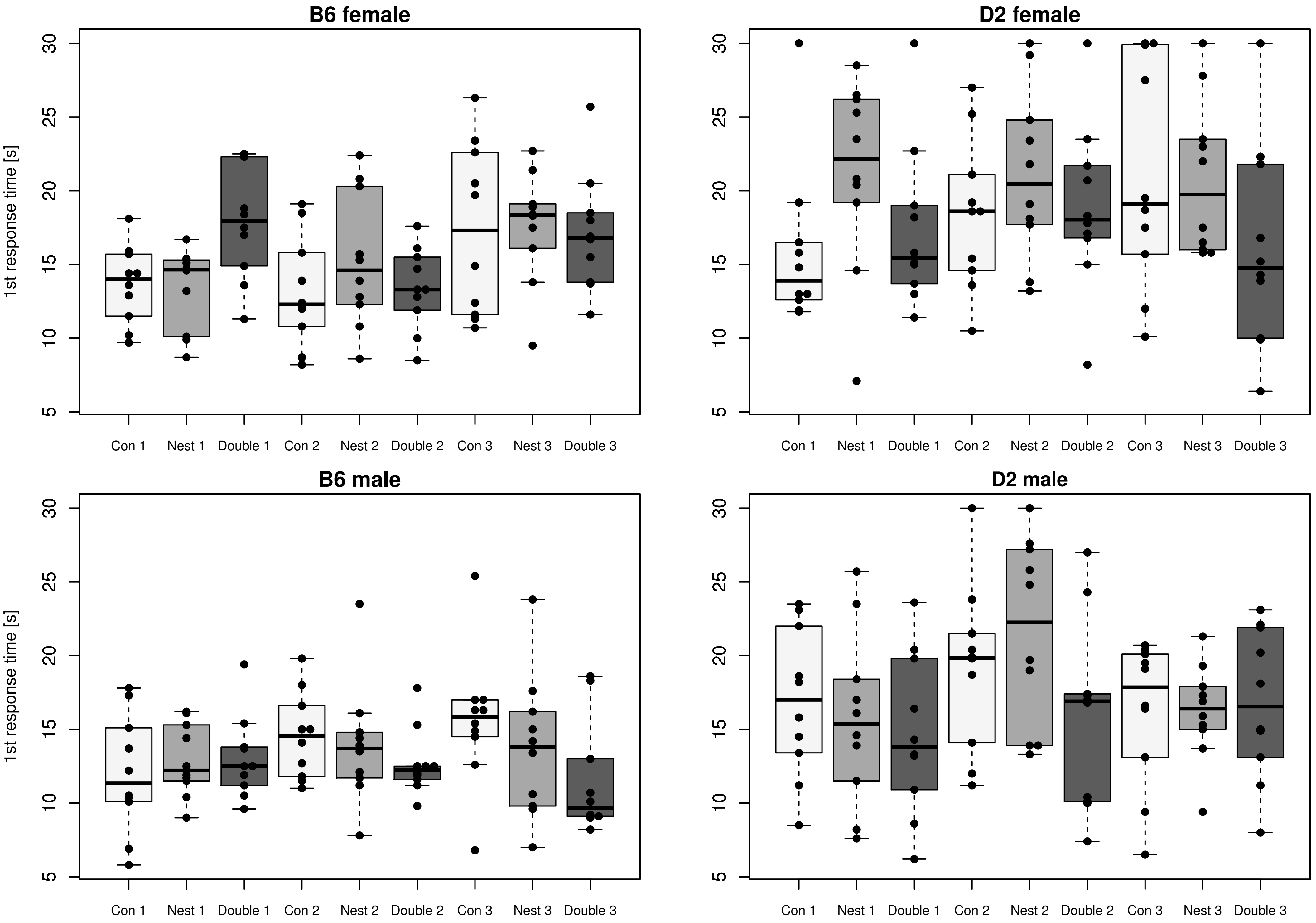
B6 female
D2 female
B6 male
D2 male
1st response time [s]
5
10
15
20
25
30
Con 1
Nest 1
Double 1
Con 2
Nest 2
Double 2
Con 3
Nest 3
Double 3

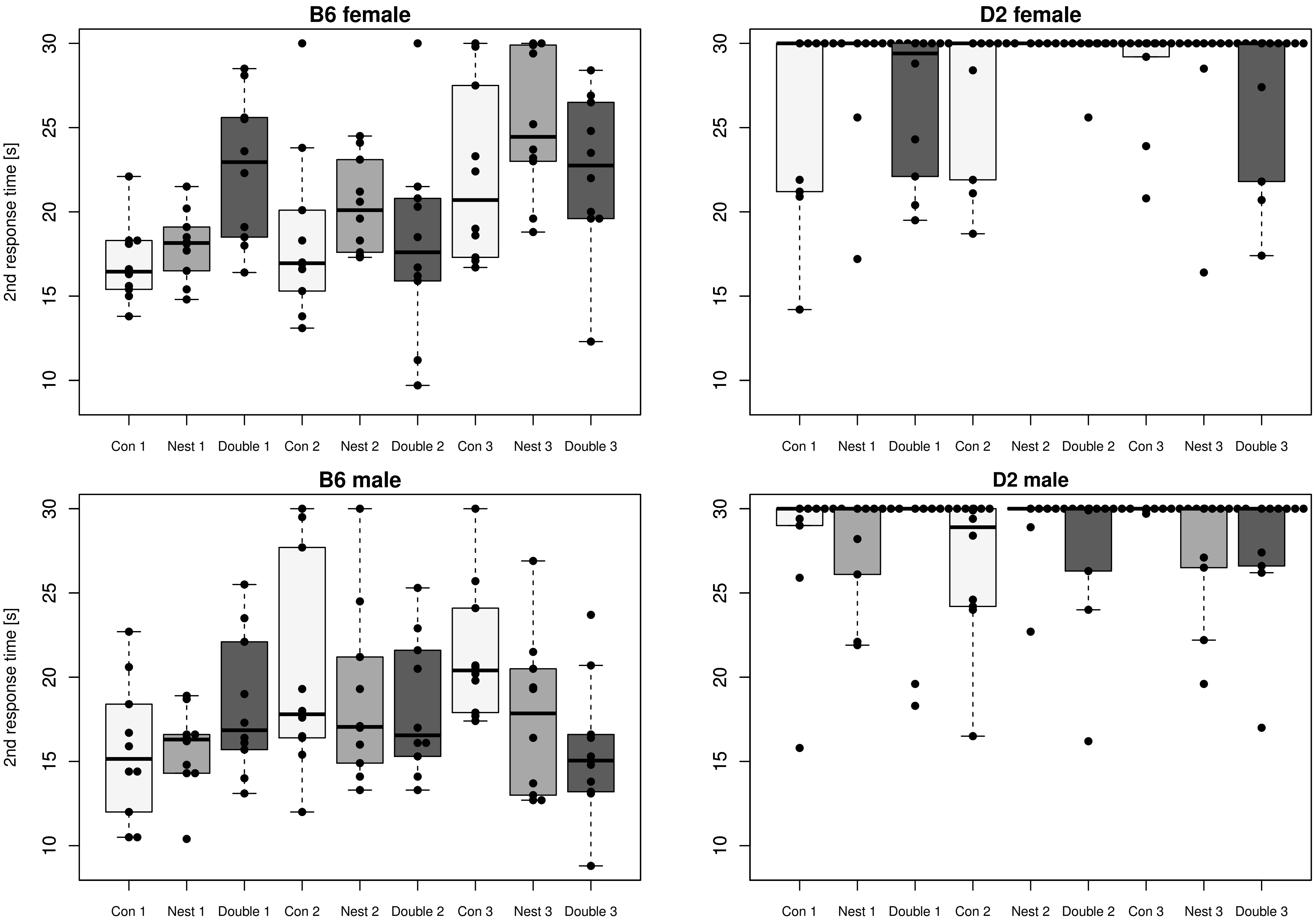
B6 female
D2 female
B6 male
D2 male
2nd response time [s]
30
25
20
15
10
Con 1
Nest 1
Double 1
Con 2
Nest 2
Double 2
Con 3
Nest 3
Double 3

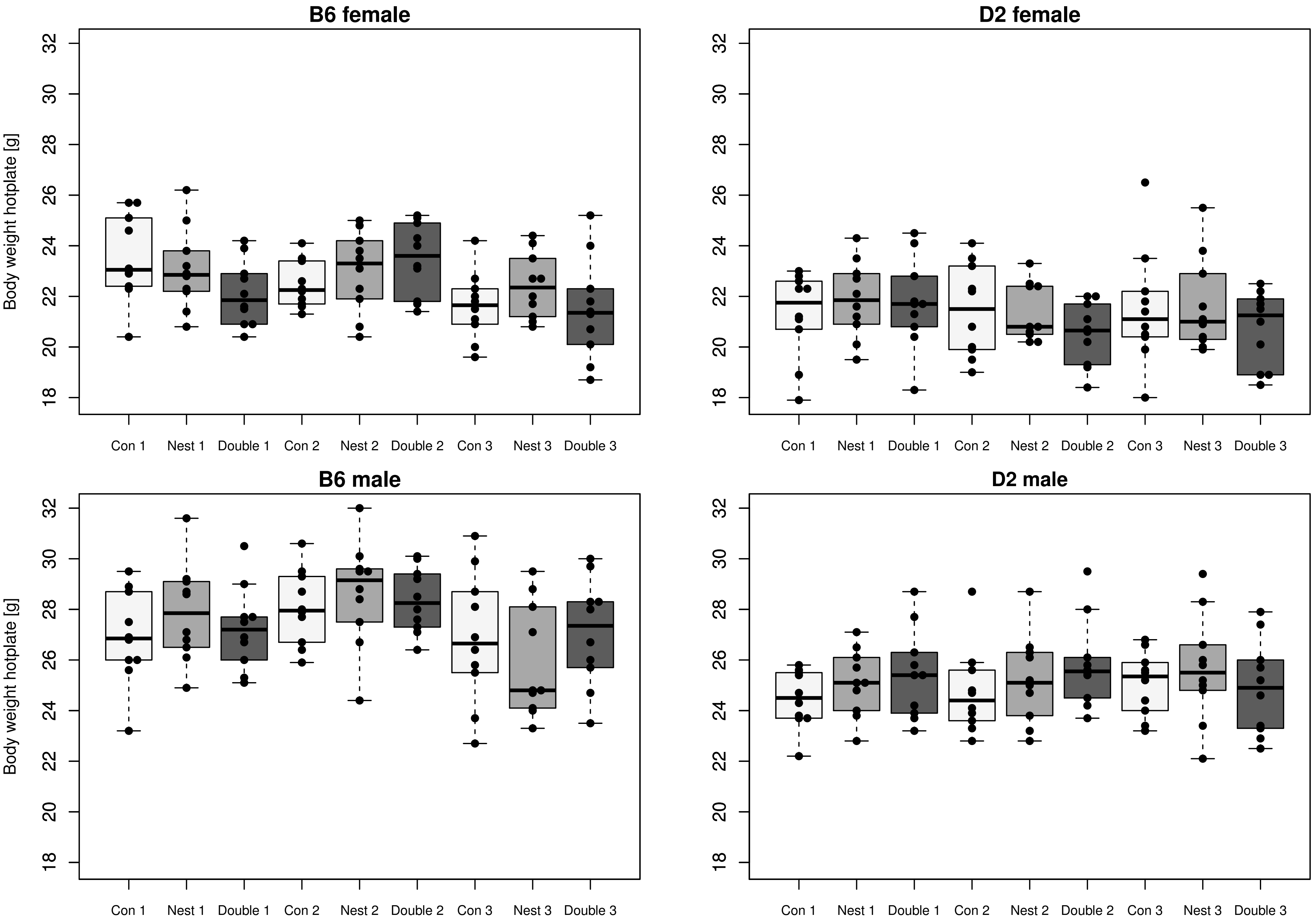

B6 female
D2 female
B6 male
D2 male
Body weight hotplate [g]
18
20
22
24
26
28
30
32
Con 1
Nest 1
Double 1
Con 2
Nest 2
Double 2
Con 3
Nest 3
Double 3

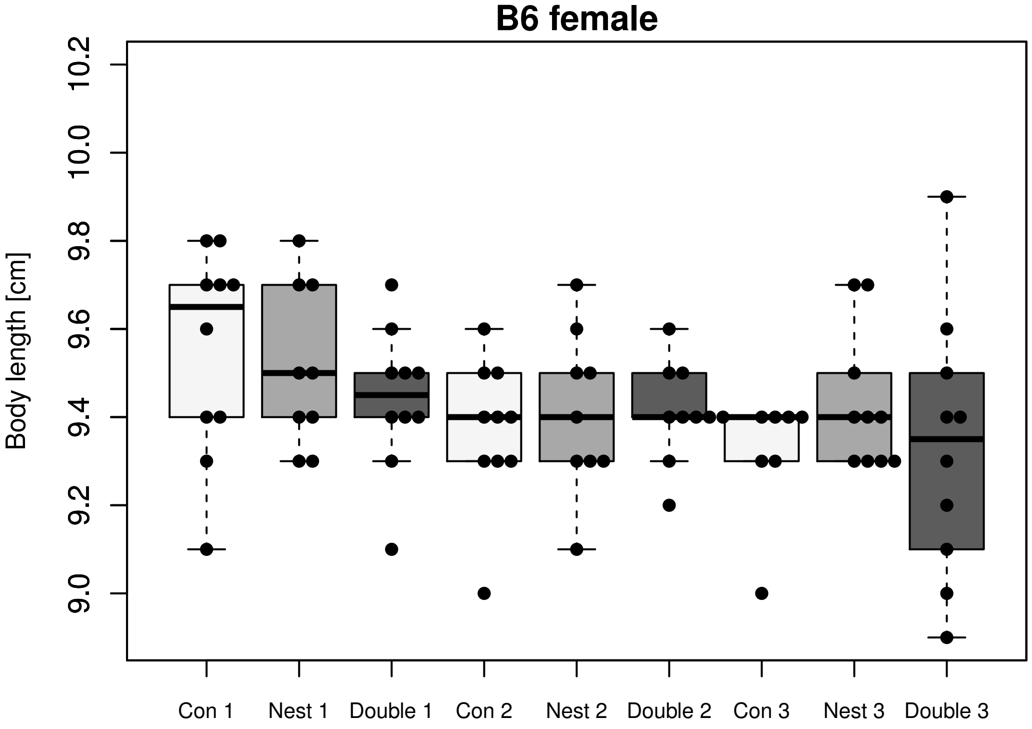

B6 female
Body length [cm]
10.2
10.0
9.8
9.6
9.4
9.2
9.0
Con 1
Nest 1
Double 1
Con 2
Nest 2
Double 2
Con 3
Nest 3
Double 3

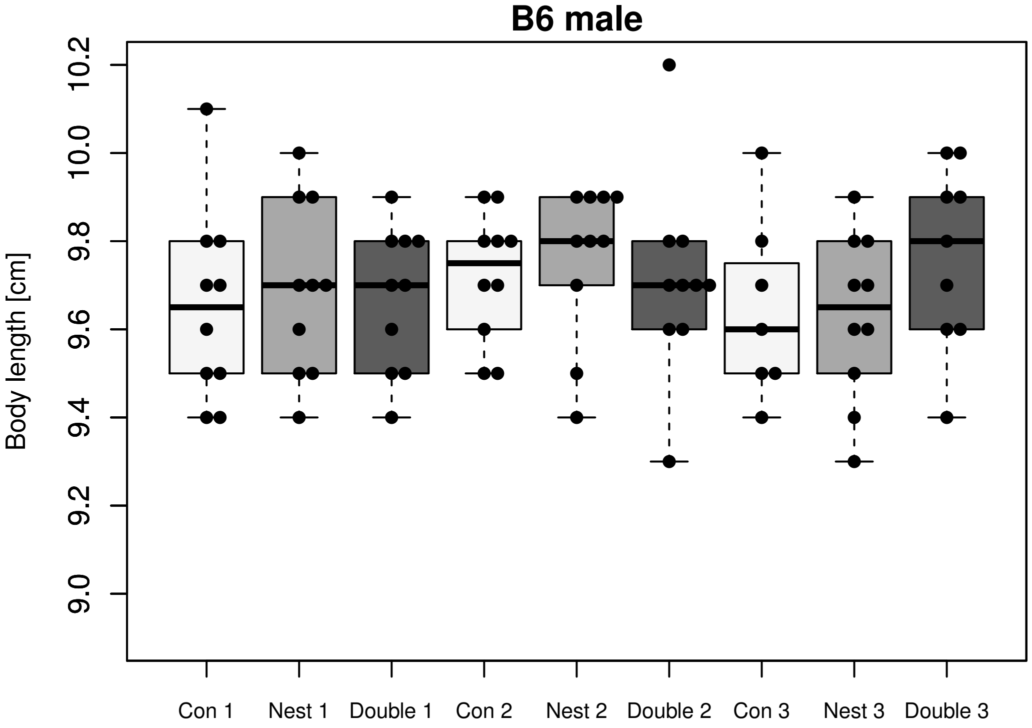

B6 male
Body length [cm]
10.2
10.0
9.8
9.6
9.4
9.2
9.0
Con 1
Nest 1
Double 1
Con 2
Nest 2
Double 2
Con 3
Nest 3
Double 3

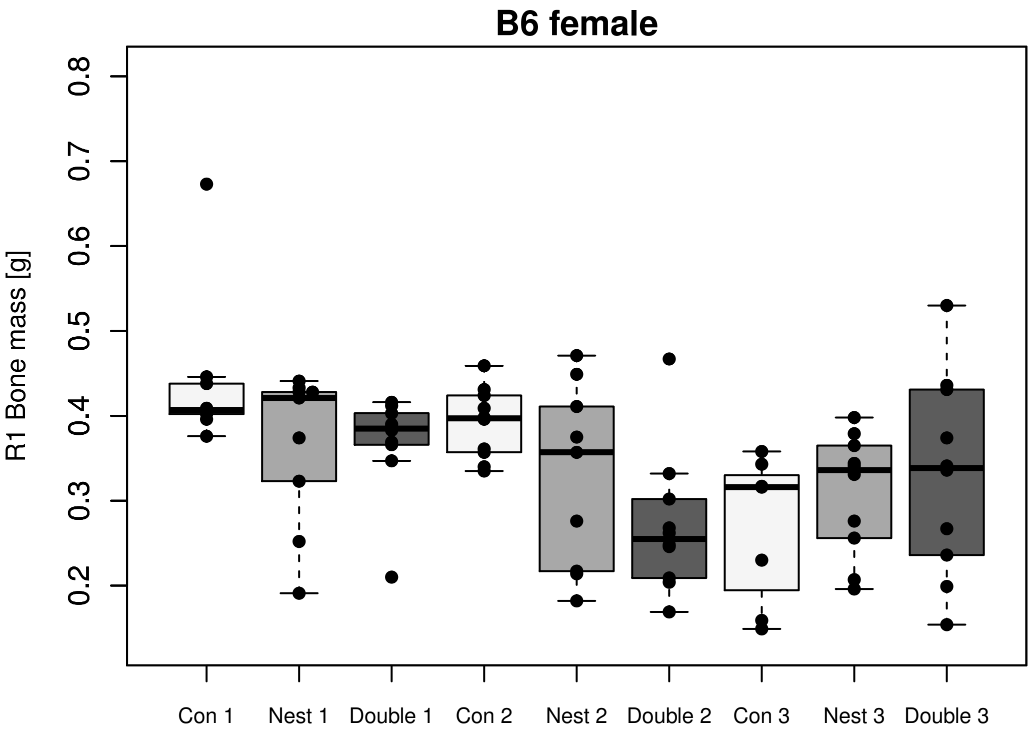
B6 female
R1 Bone mass [g]
0.2
0.3
0.4
0.5
0.6
0.7
0.8
Con 1
Nest 1
Double 1
Con 2
Nest 2
Double 2
Con 3
Nest 3
Double 3

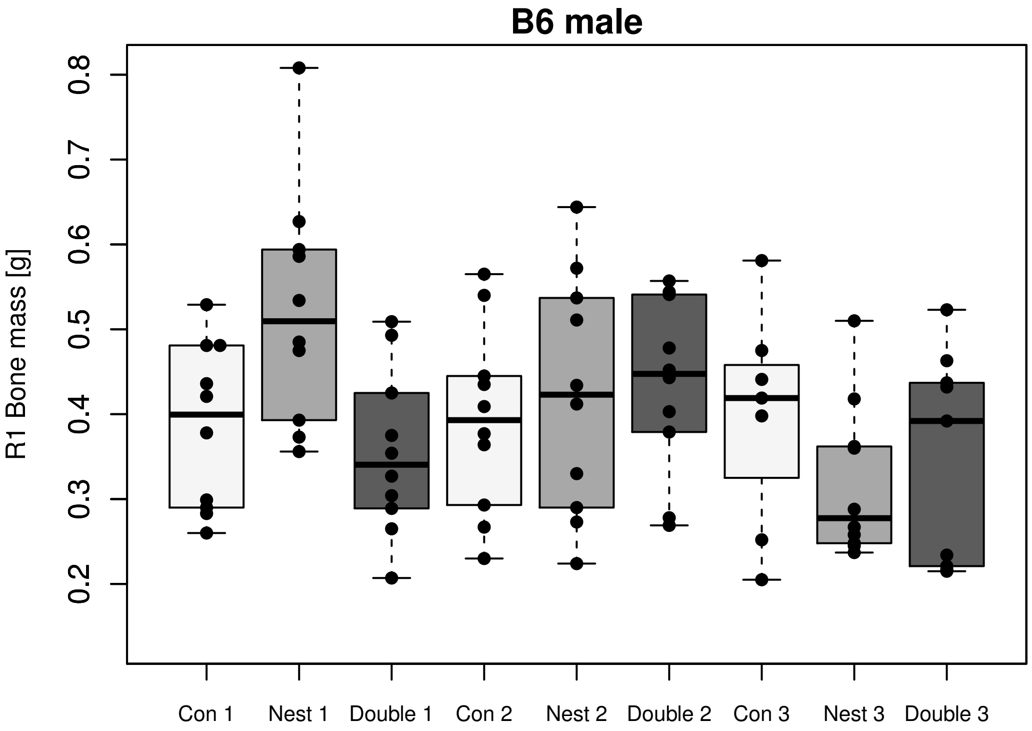
B6 male
R1 Bone mass [g]
0.2
0.3
0.4
0.5
0.6
0.7
0.8
Con 1
Nest 1
Double 1
Con 2
Nest 2
Double 2
Con 3
Nest 3
Double 3

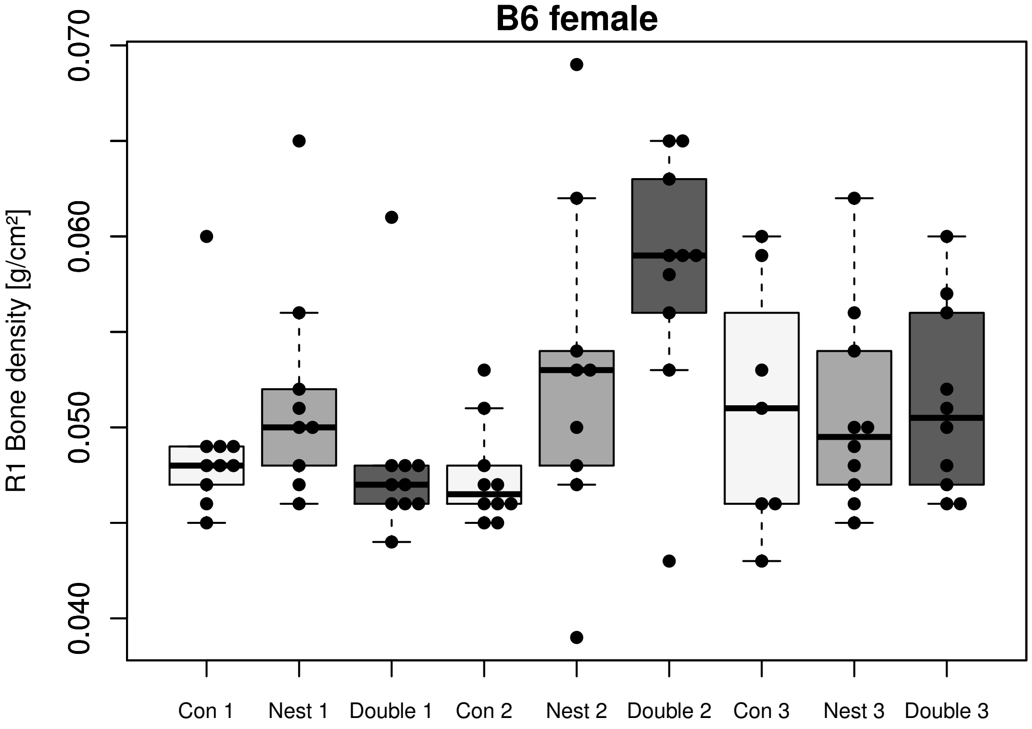
B6 female
R1 Bone density [g/cm²]
0.040
0.050
0.060
0.070
Con 1
Nest 1
Double 1
Con 2
Nest 2
Double 2
Con 3
Nest 3
Double 3

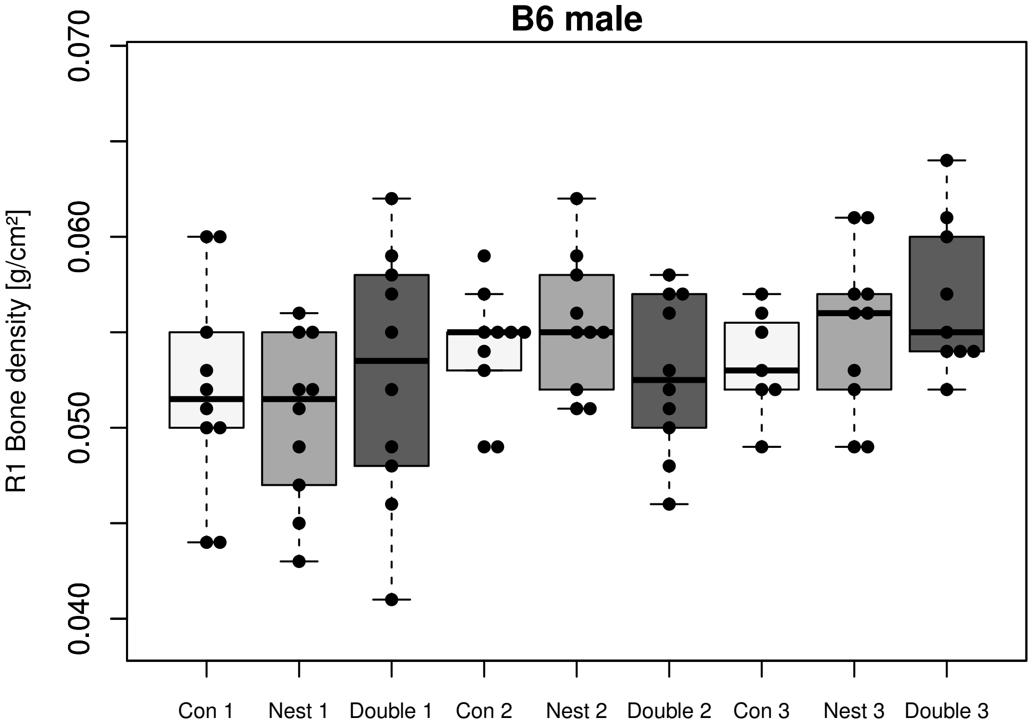
B6 male
R1 Bone density [g/cm²]
0.040
0.050
0.060
0.070
Con 1
Nest 1
Double 1
Con 2
Nest 2
Double 2
Con 3
Nest 3
Double 3

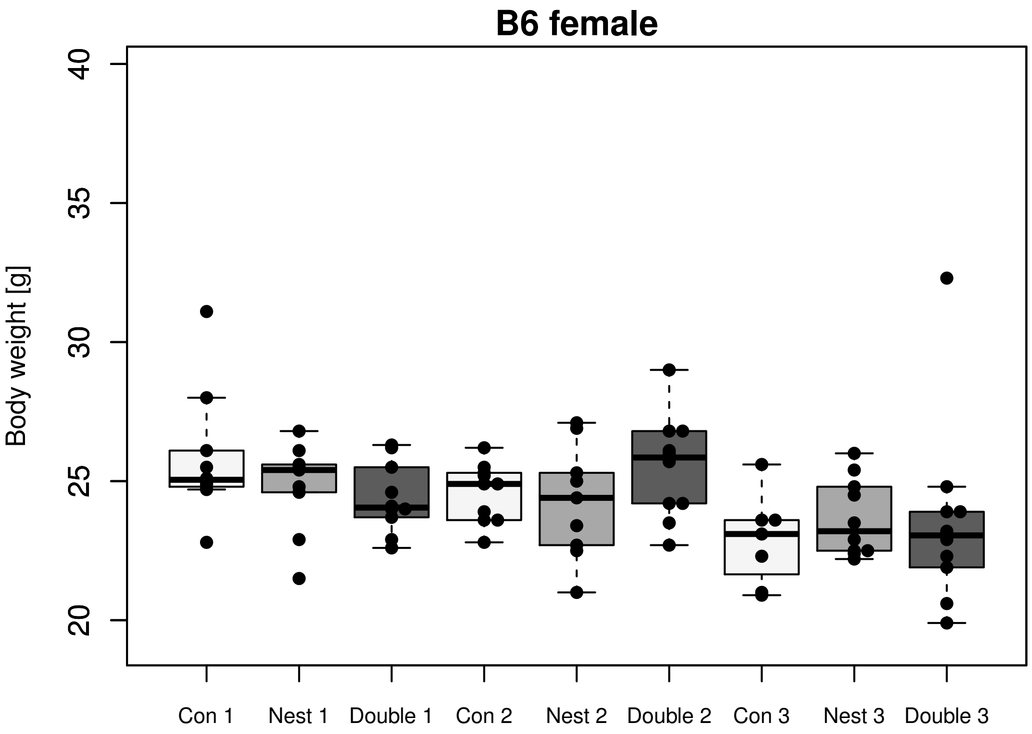
B6 female
Body weight [g]
40
35
30
25
20
Con 1
Nest 1
Double 1
Con 2
Nest 2
Double 2
Con 3
Nest 3
Double 3

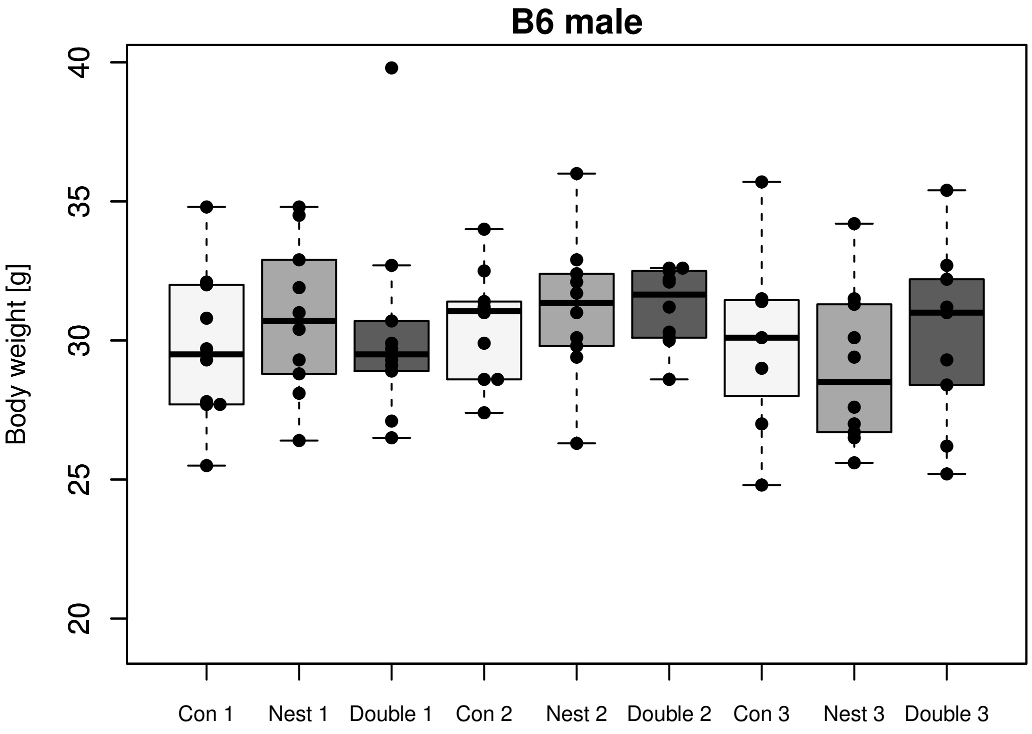
B6 male
Body weight [g]
40
35
30
25
20
Con 1
Nest 1
Double 1
Con 2
Nest 2
Double 2
Con 3
Nest 3
Double 3

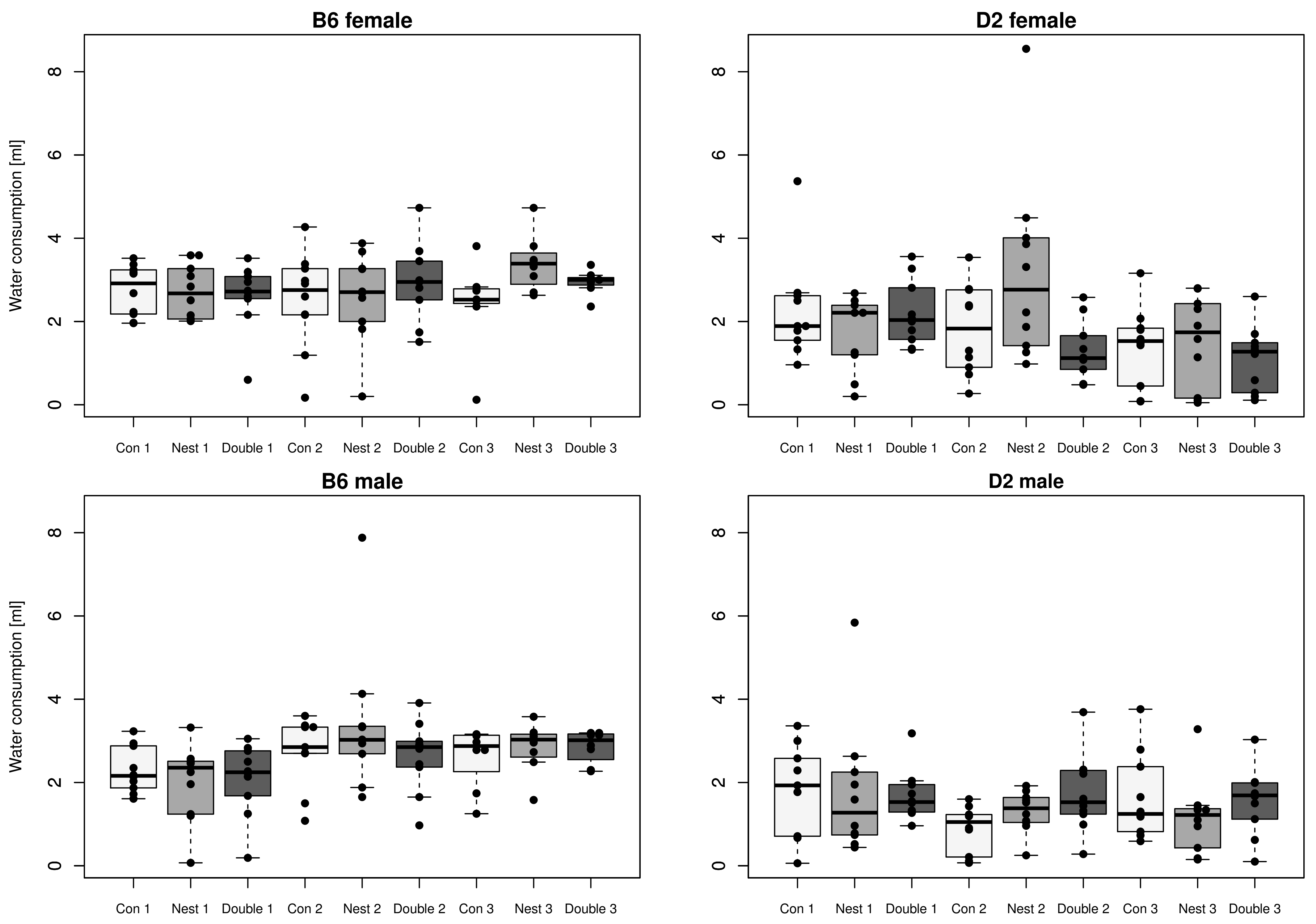
B6 female
D2 female
B6 male
D2 male
Water consumption [ml]
Water consumption [ml]
0
2
4
6
8
Con 1
Nest 1
Double 1
Con 2
Nest 2
Double 2
Con 3
Nest 3
Double 3

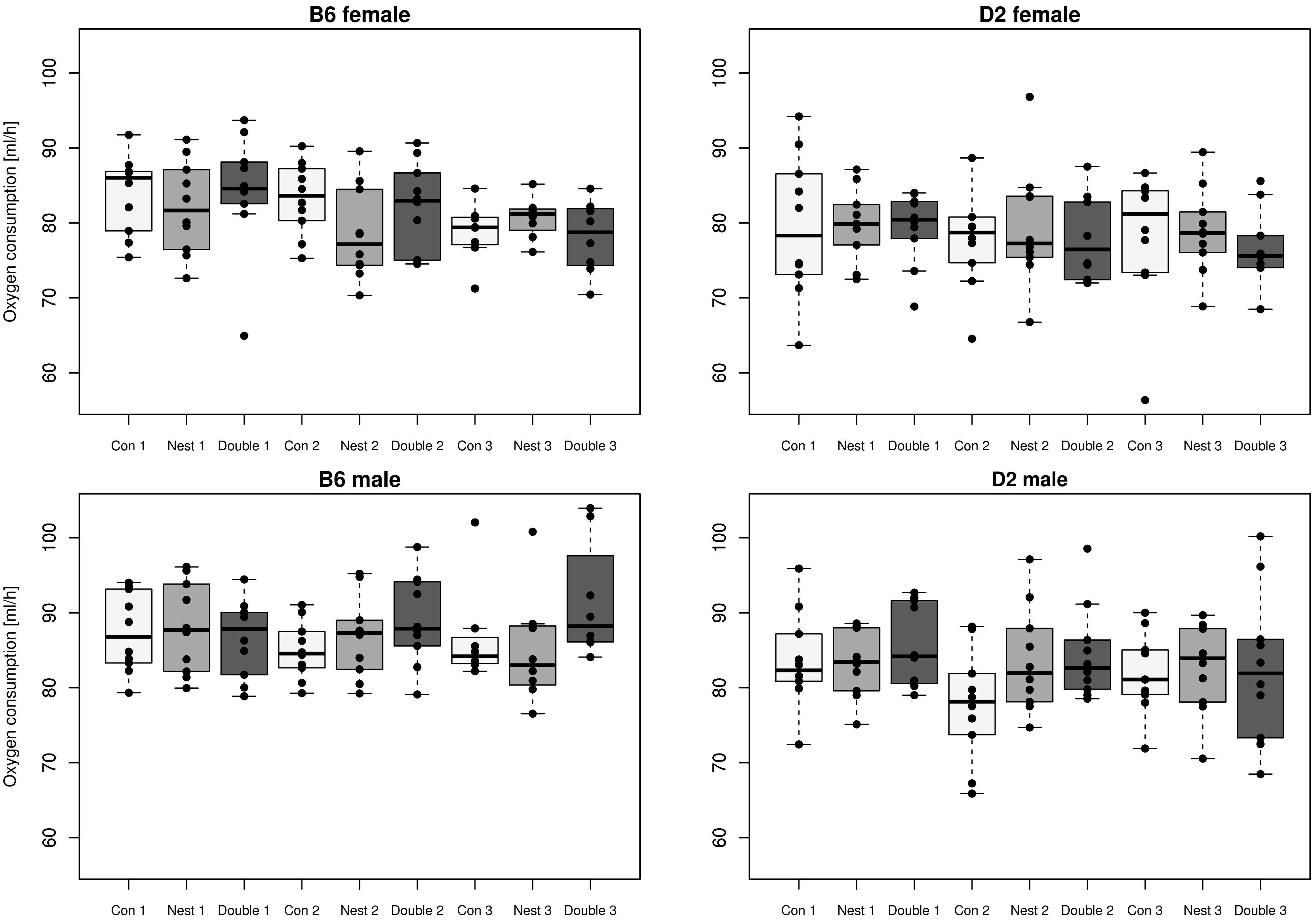

B6 female
D2 female
B6 male
D2 male
Oxygen consumption [ml/h]
60
70
80
90
100
Con 1
Nest 1
Double 1
Con 2
Nest 2
Double 2
Con 3
Nest 3
Double 3

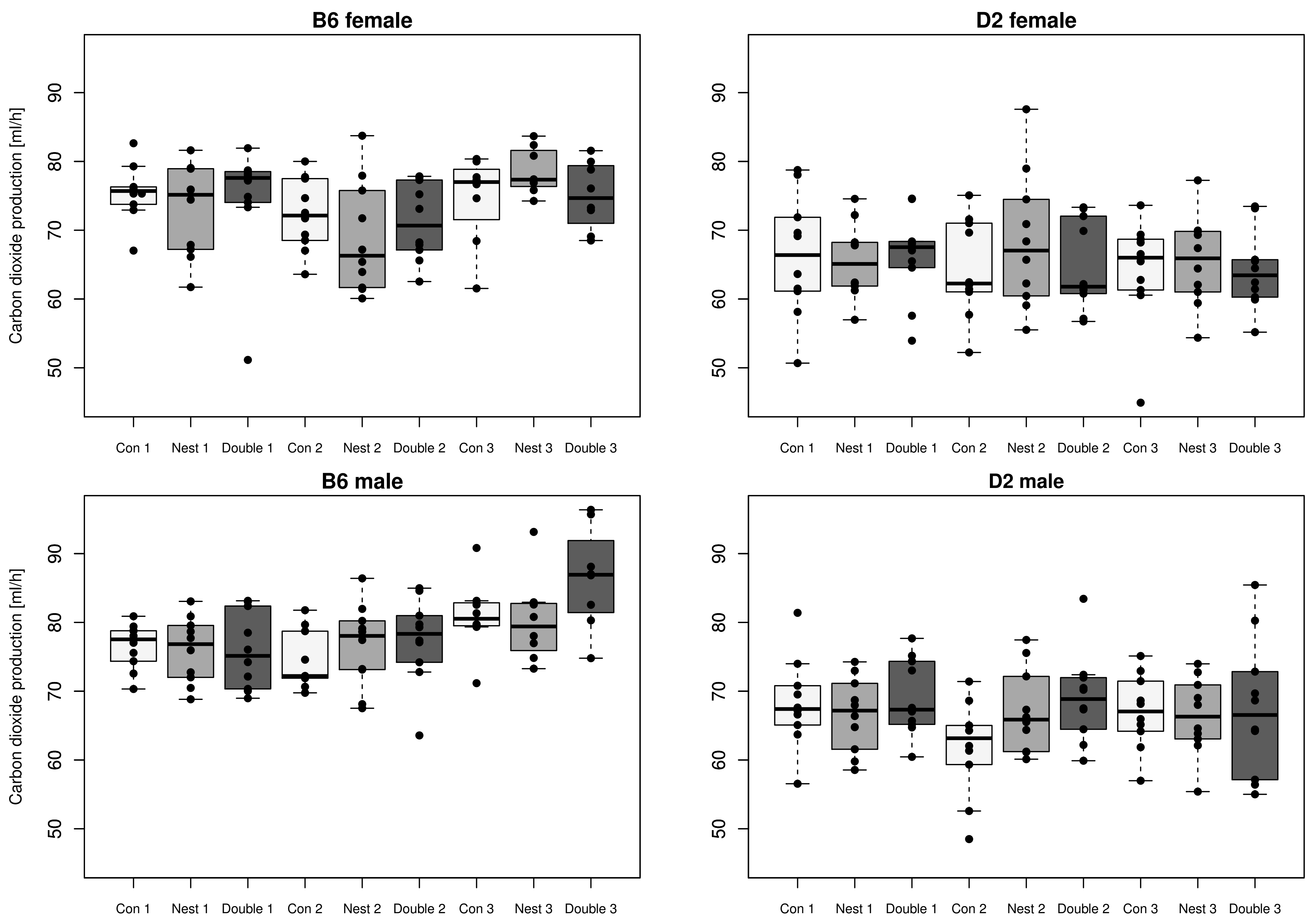
B6 female
D2 female
B6 male
D2 male
Carbon dioxide production [ml/h]
50
60
70
80
90
Con 1
Nest 1
Double 1
Con 2
Nest 2
Double 2
Con 3
Nest 3
Double 3

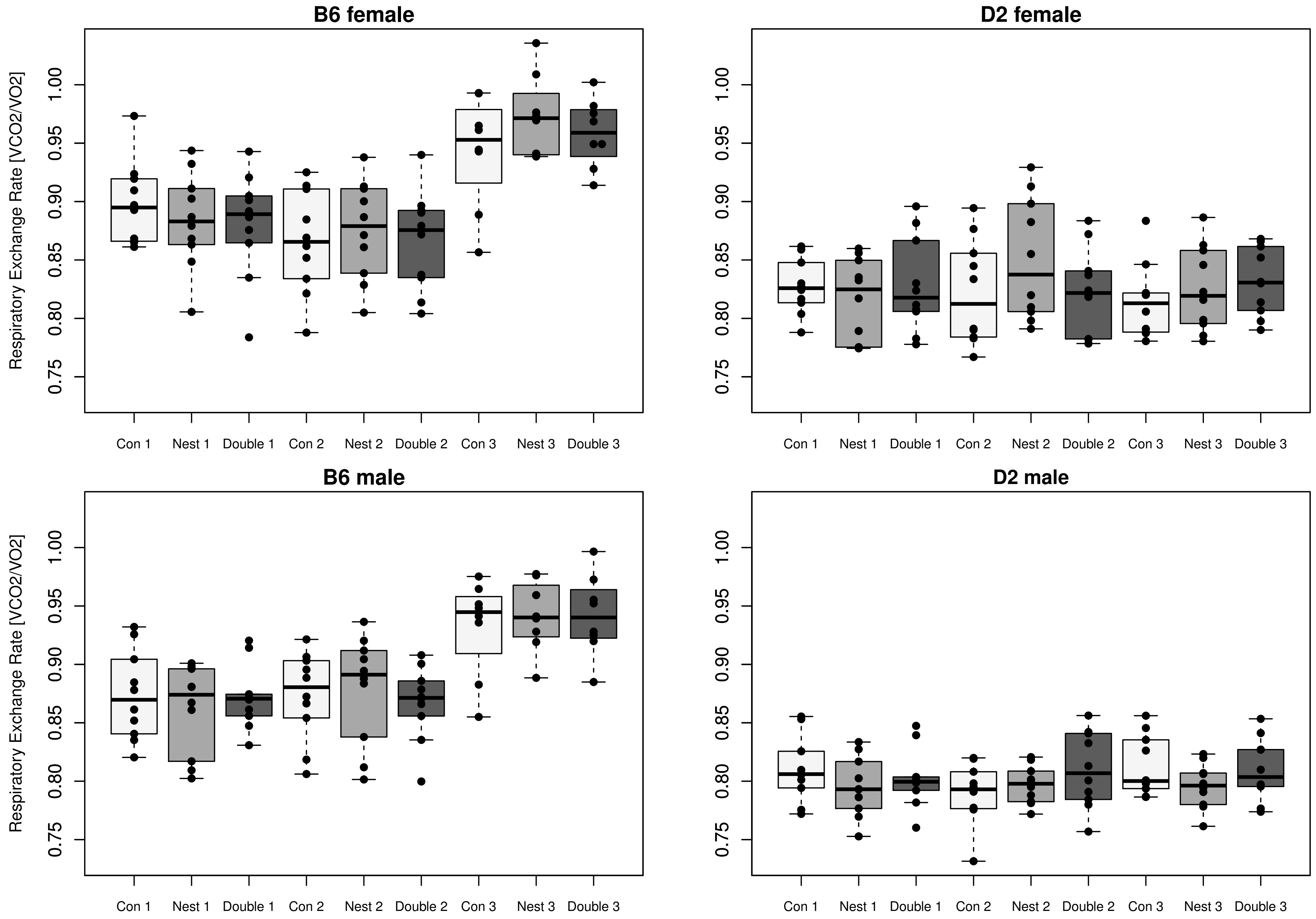
B6 female
D2 female
B6 male
D2 male
Respiratory Exchange Rate [VCO2/VO2]
0.75
0.80
0.85
0.90
0.95
1.00
Con 1
Nest 1
Double 1
Con 2
Nest 2
Double 2
Con 3
Nest 3
Double 3

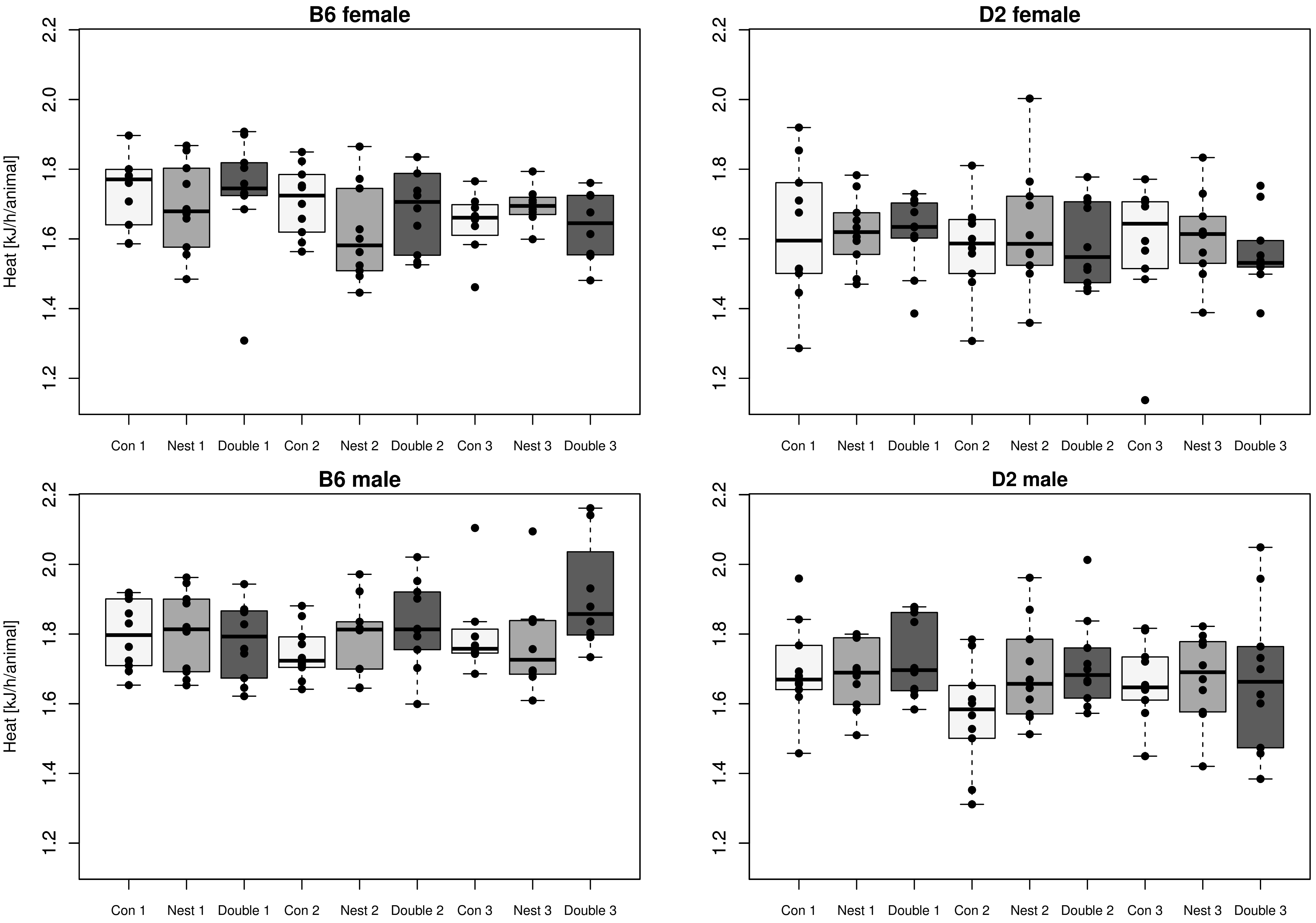
B6 female
D2 female
B6 male
D2 male
Heat [kJ/h/animal]
1.2
1.4
1.6
1.8
2.0
2.2
Con 1
Nest 1
Double 1
Con 2
Nest 2
Double 2
Con 3
Nest 3
Double 3

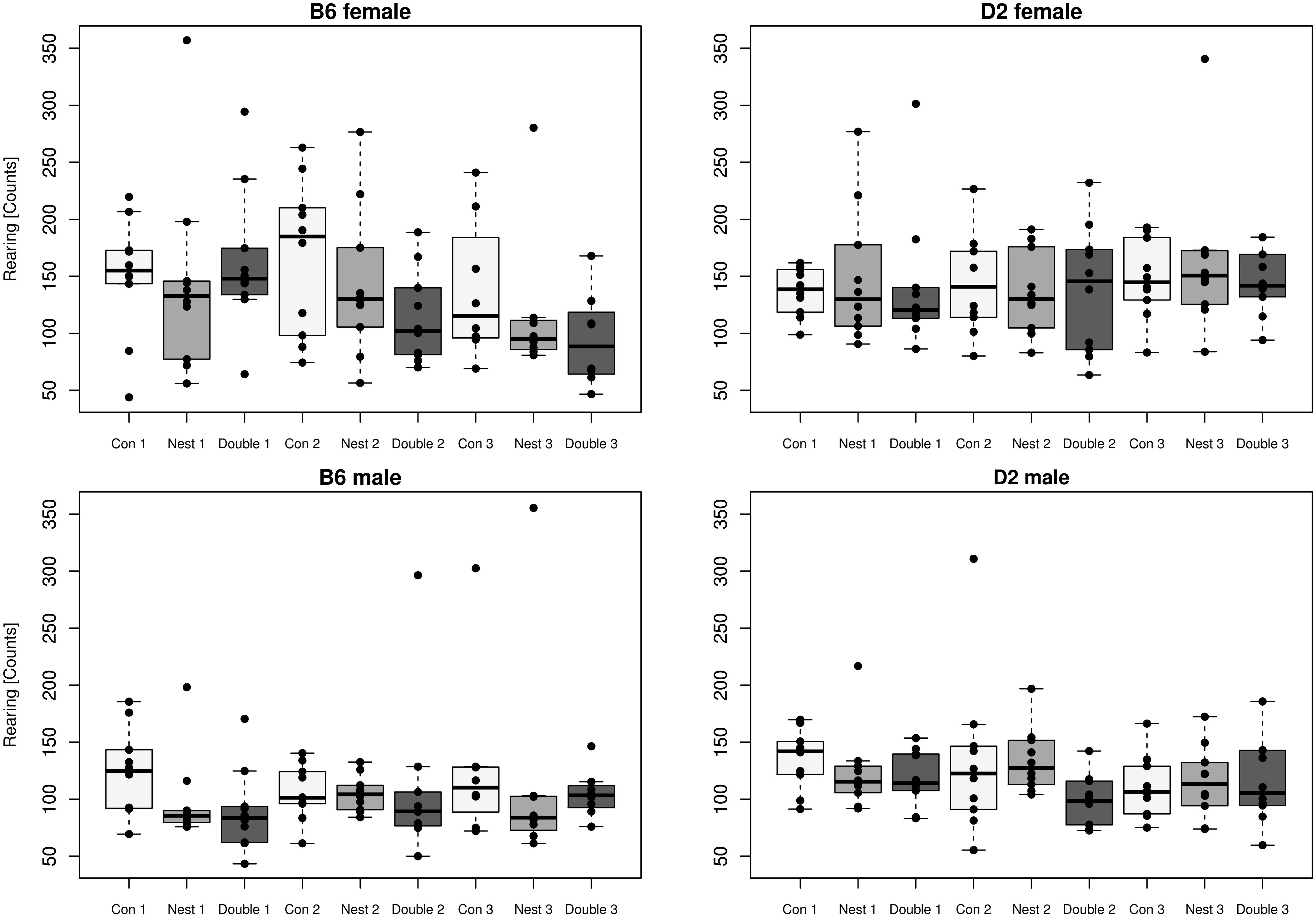
B6 female
D2 female
B6 male
D2 male
Rearing [Counts]
350
300
250
200
150
100
50
Con 1
Nest 1
Double 1
Con 2
Nest 2
Double 2
Con 3
Nest 3
Double 3

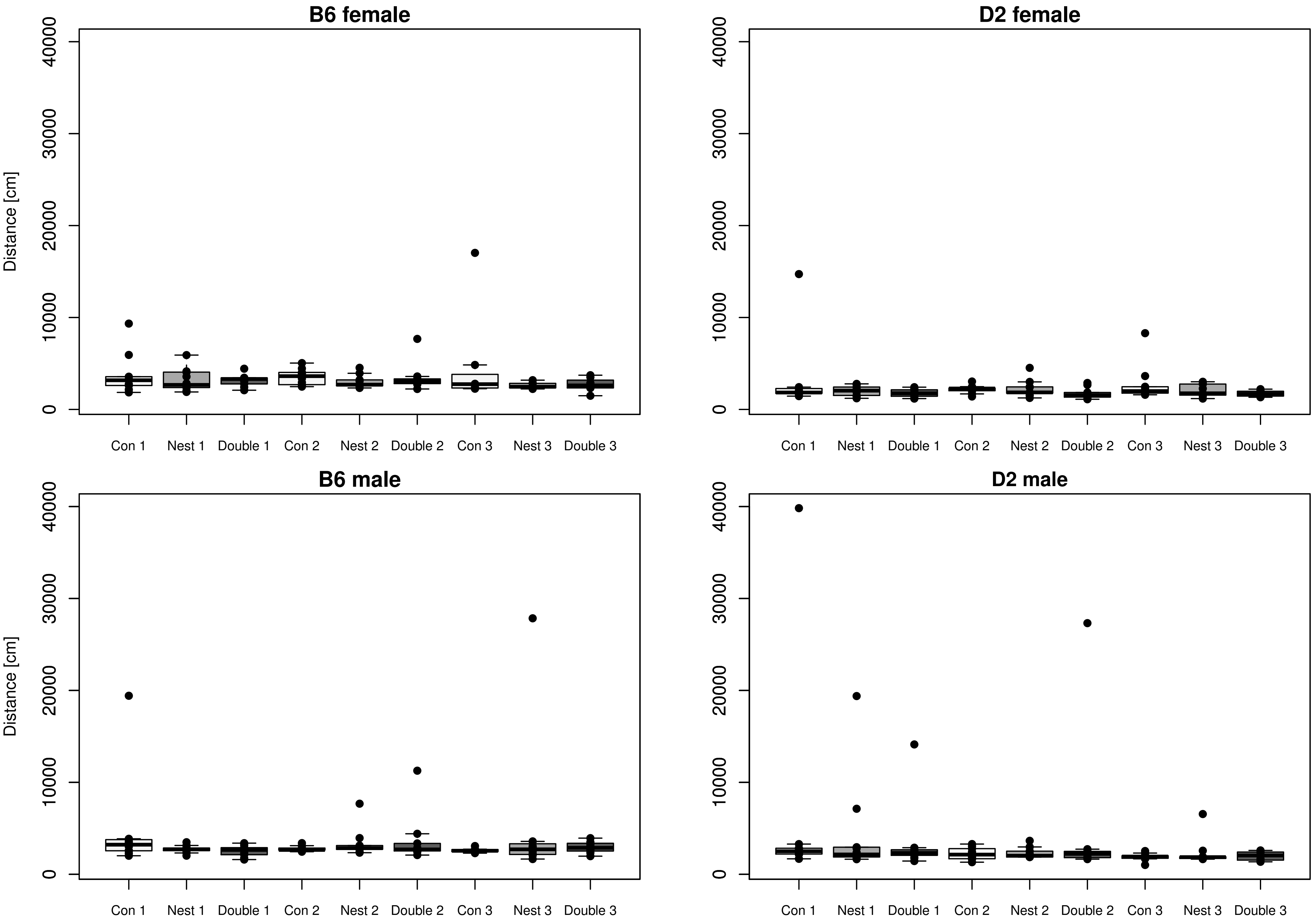
B6 female
D2 female
B6 male
D2 male
Distance [cm]
0
10000
20000
30000
40000
Con 1
Nest 1
Double 1
Con 2
Nest 2
Double 2
Con 3
Nest 3
Double 3

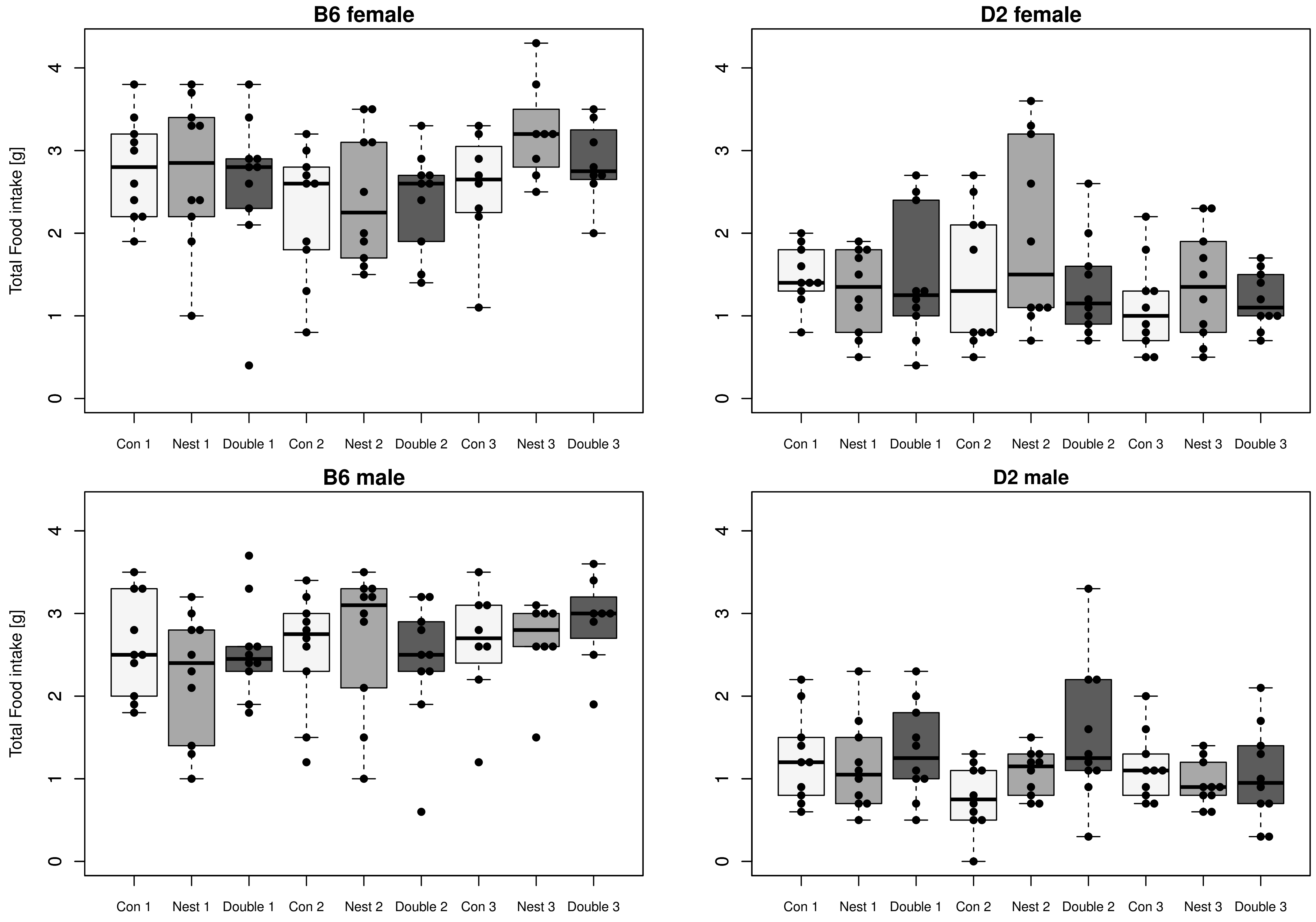
B6 female
D2 female
B6 male
D2 male
Total Food intake [g]
0
1
2
3
4
Con 1
Nest 1
Double 1
Con 2
Nest 2
Double 2
Con 3
Nest 3
Double 3

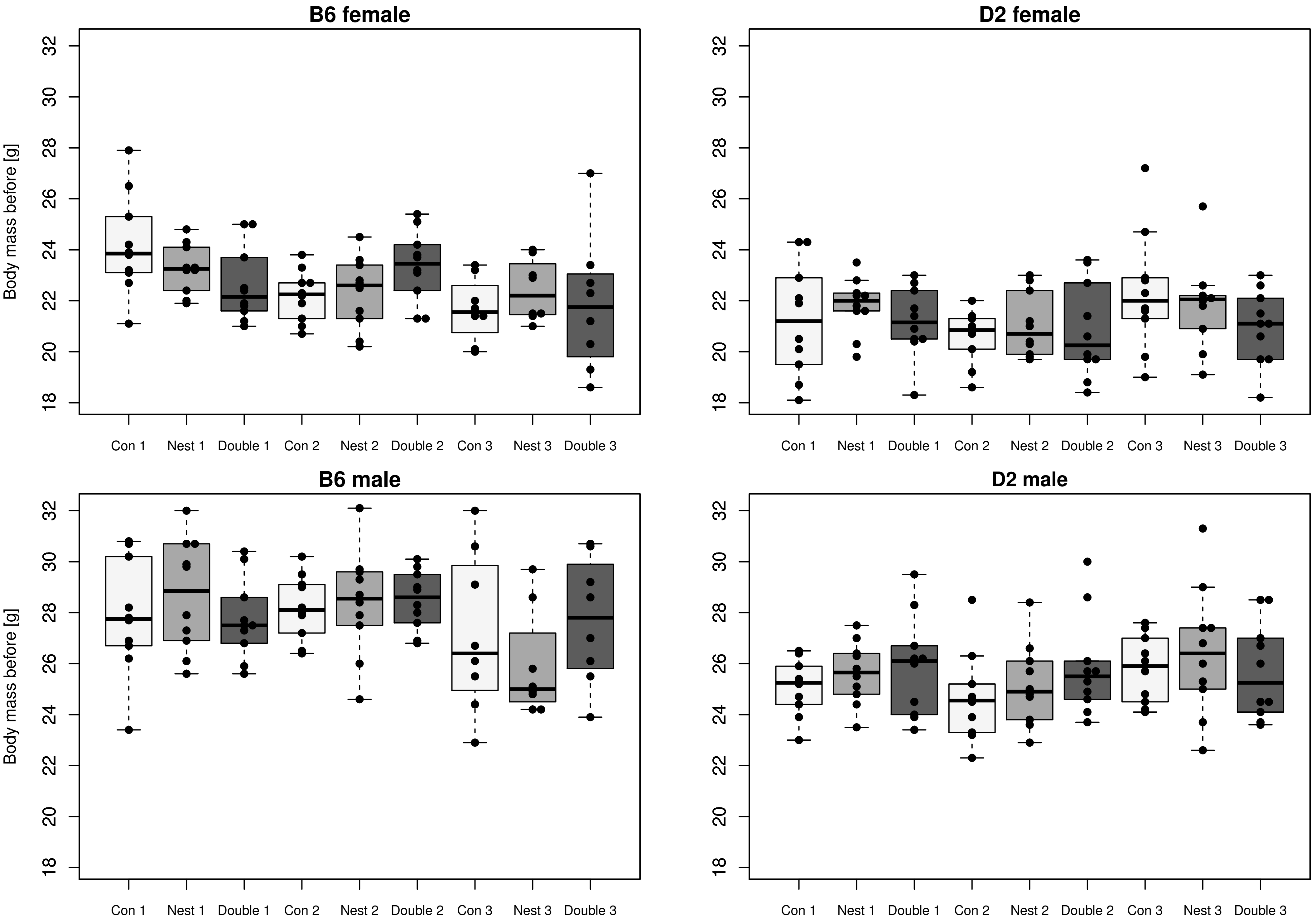
B6 female
D2 female
B6 male
D2 male
Body mass before [g]
18
20
22
24
26
28
30
32
Con 1
Nest 1
Double 1
Con 2
Nest 2
Double 2
Con 3
Nest 3
Double 3

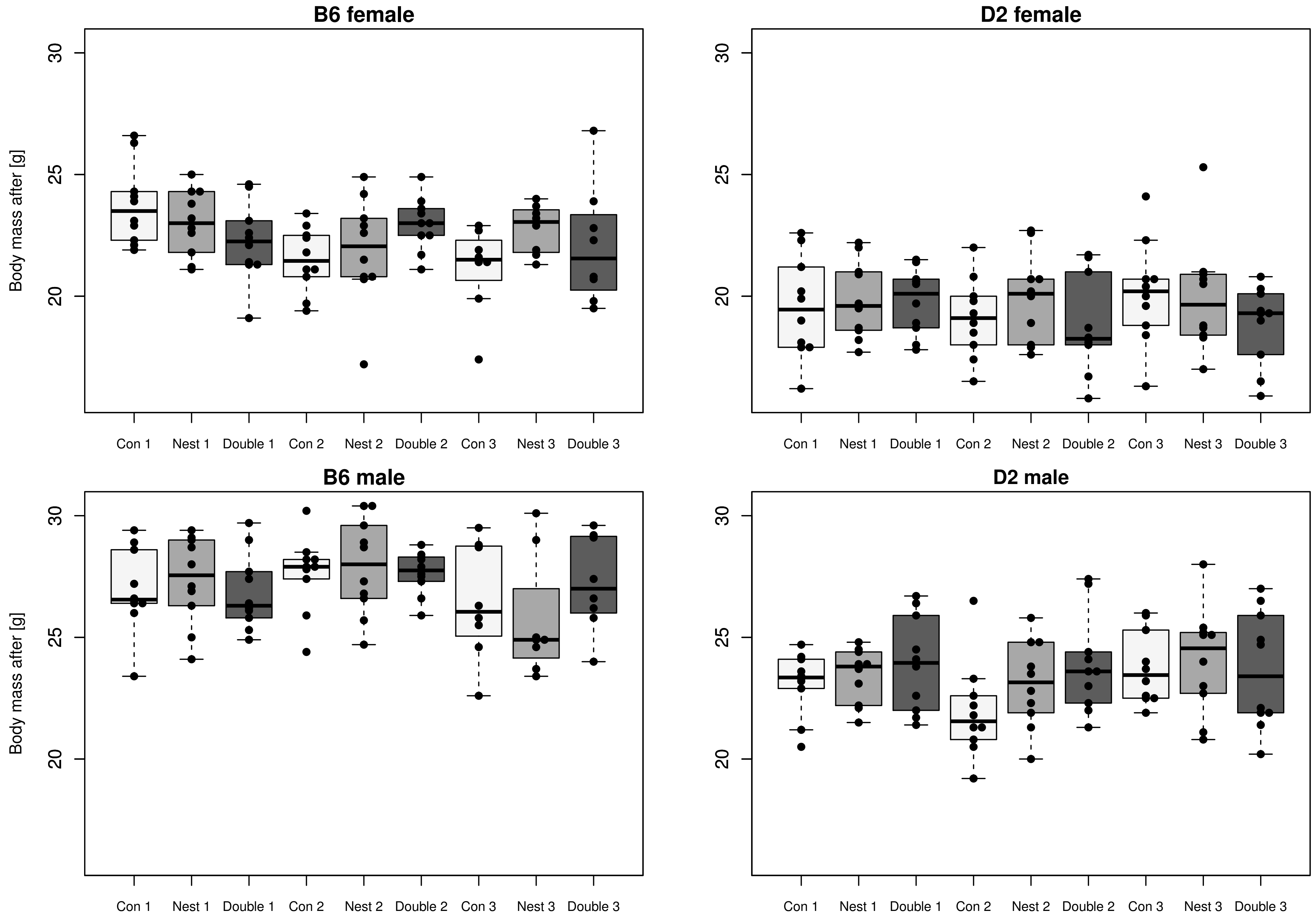
B6 female
D2 female
B6 male
D2 male
Body mass after [g]
30
25
20
Con 1
Nest 1
Double 1
Con 2
Nest 2
Double 2
Con 3
Nest 3
Double 3

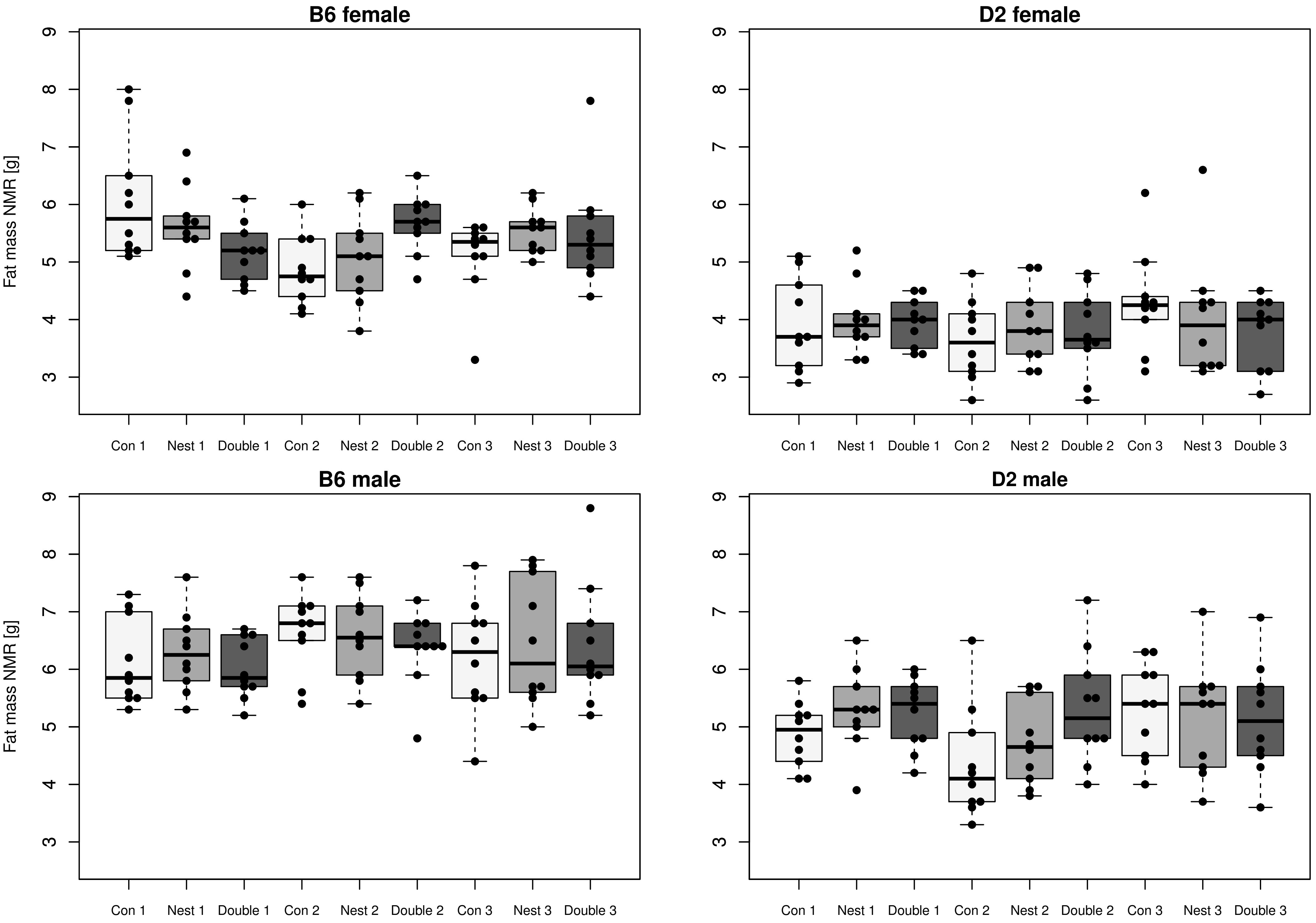
B6 female
D2 female
B6 male
D2 male
Fat mass NMR [g]
Con 1
Nest 1
Double 1
Con 2
Nest 2
Double 2
Con 3
Nest 3
Double 3

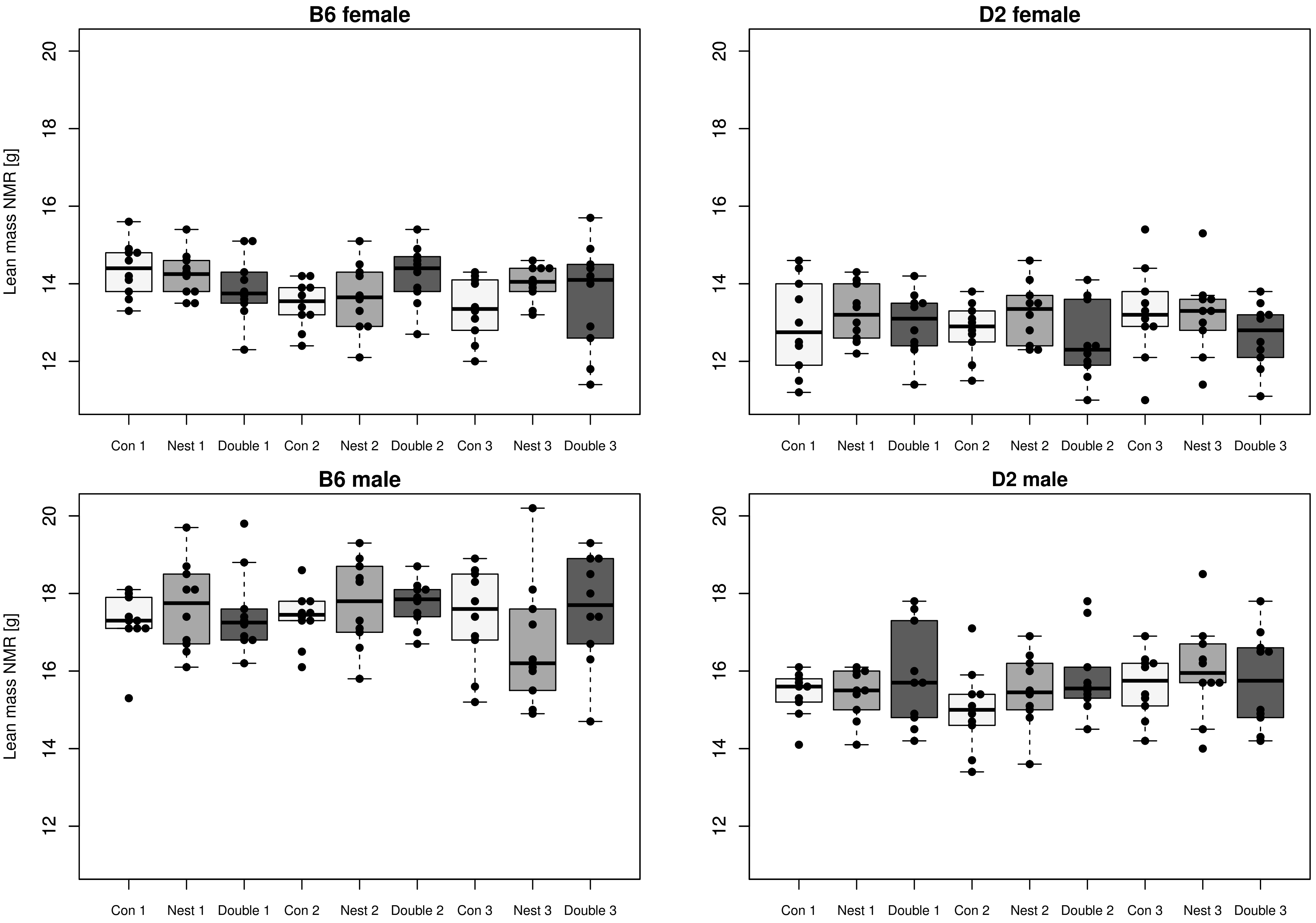

B6 female
D2 female
B6 male
D2 male
Lean mass NMR [g]
20
18
16
14
12
Con 1
Nest 1
Double 1
Con 2
Nest 2
Double 2
Con 3
Nest 3
Double 3

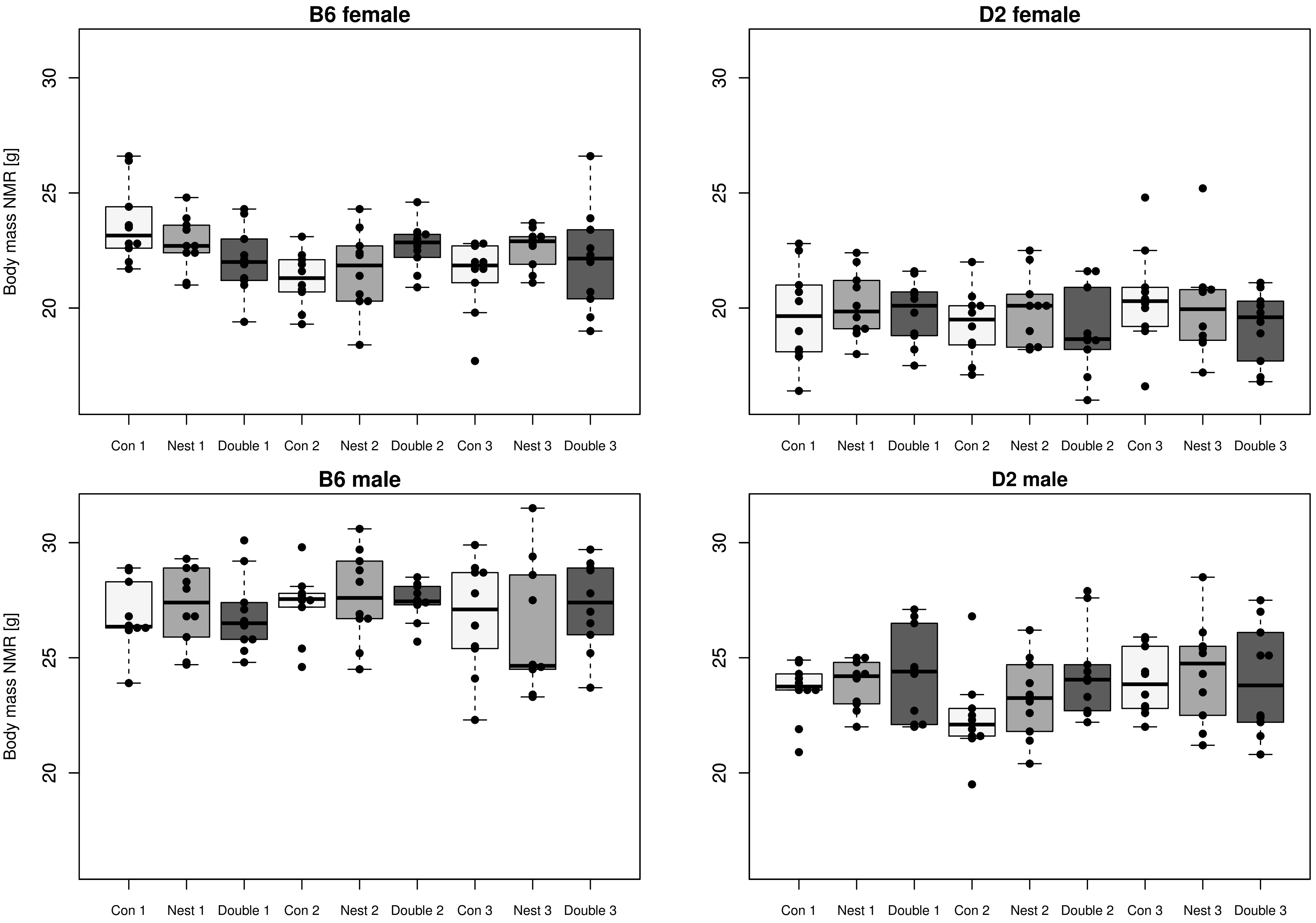

B6 female
D2 female
B6 male
D2 male
Body mass NMR [g]
20
25
30
Con 1
Nest 1
Double 1
Con 2
Nest 2
Double 2
Con 3
Nest 3
Double 3

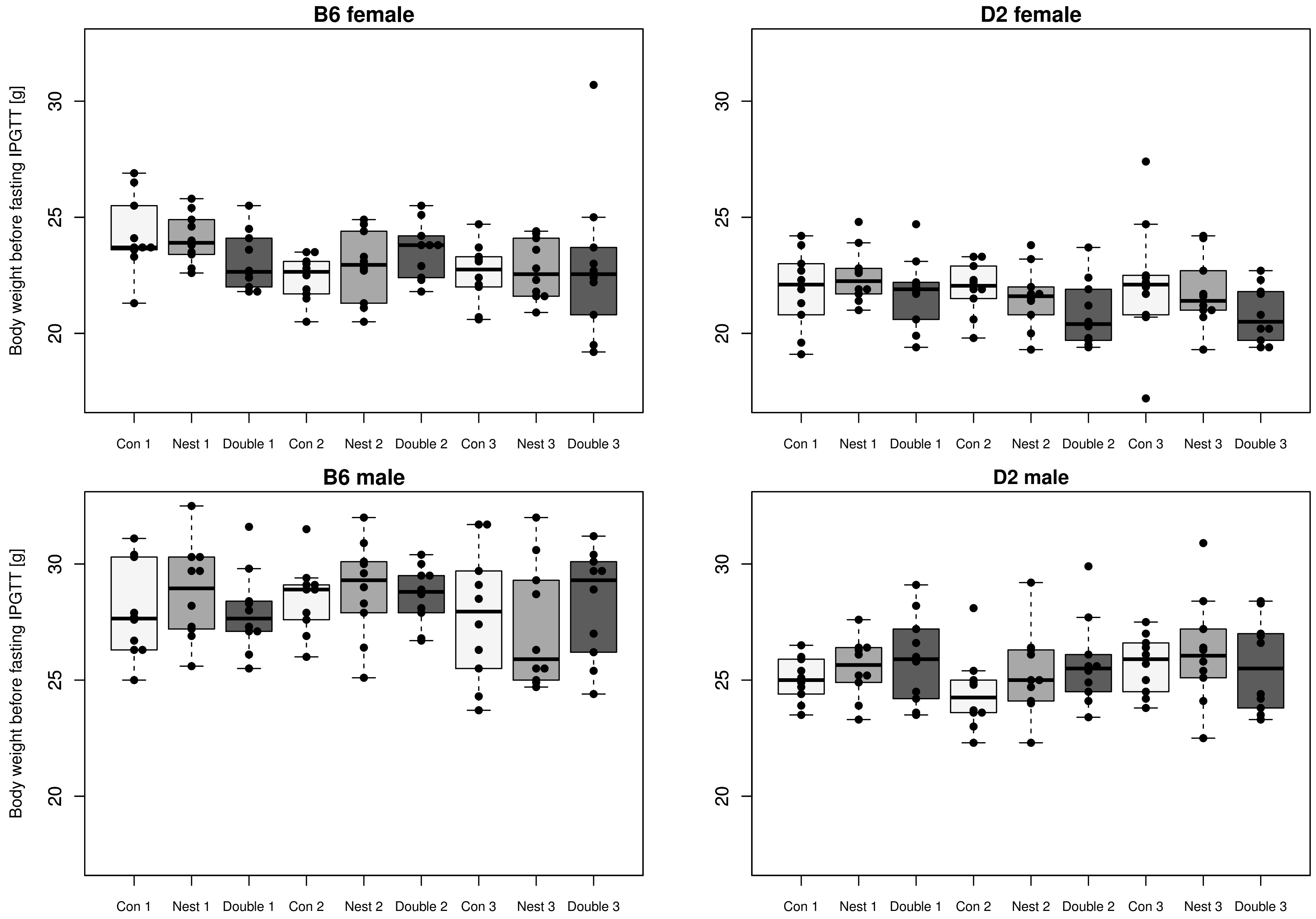
B6 female
D2 female
B6 male
D2 male
Body weight before fasting IPGTT [g]
20
25
30
Con 1
Nest 1
Double 1
Con 2
Nest 2
Double 2
Con 3
Nest 3
Double 3

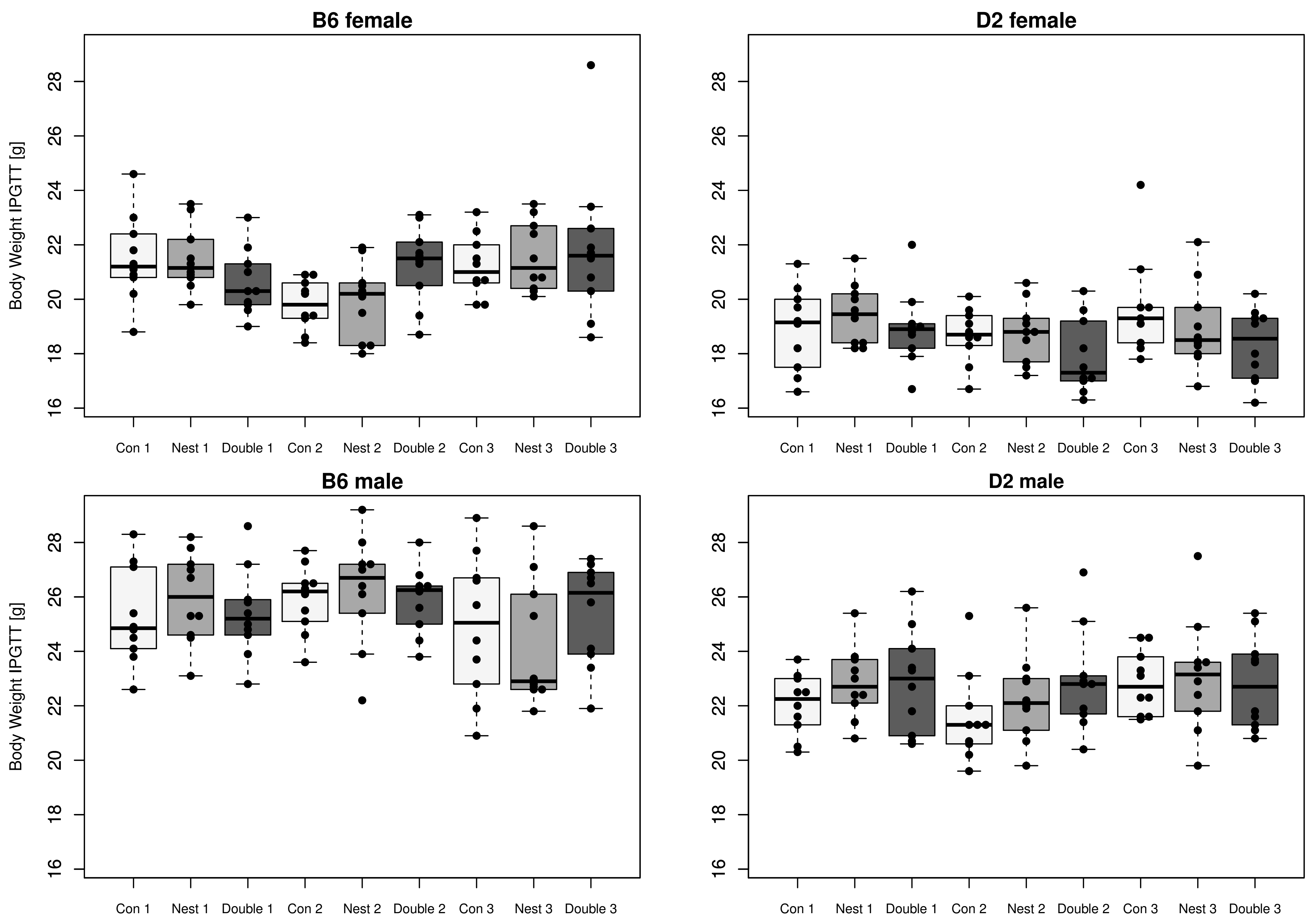
B6 female
D2 female
B6 male
D2 male
Body Weight IPGTT [g]
16
18
20
22
24
26
28
Con 1
Nest 1
Double 1
Con 2
Nest 2
Double 2
Con 3
Nest 3
Double 3

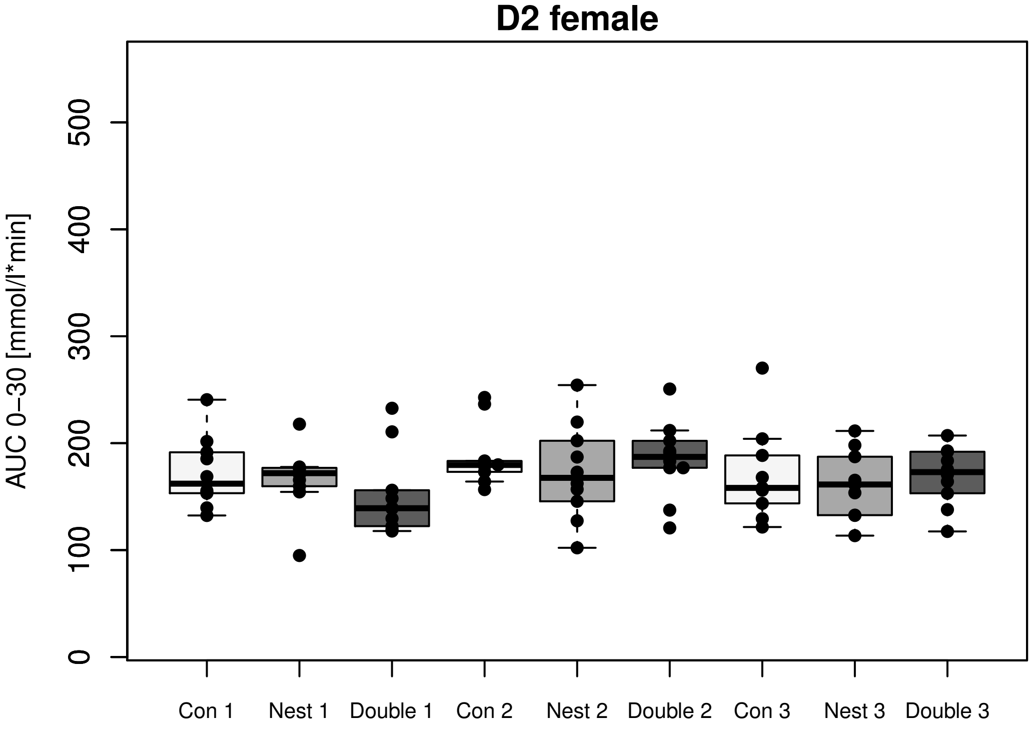
D2 female
AUC 0–30 [mmol/l*min]
0
100
200
300
400
500
Con 1
Nest 1
Double 1
Con 2
Nest 2
Double 2
Con 3
Nest 3
Double 3

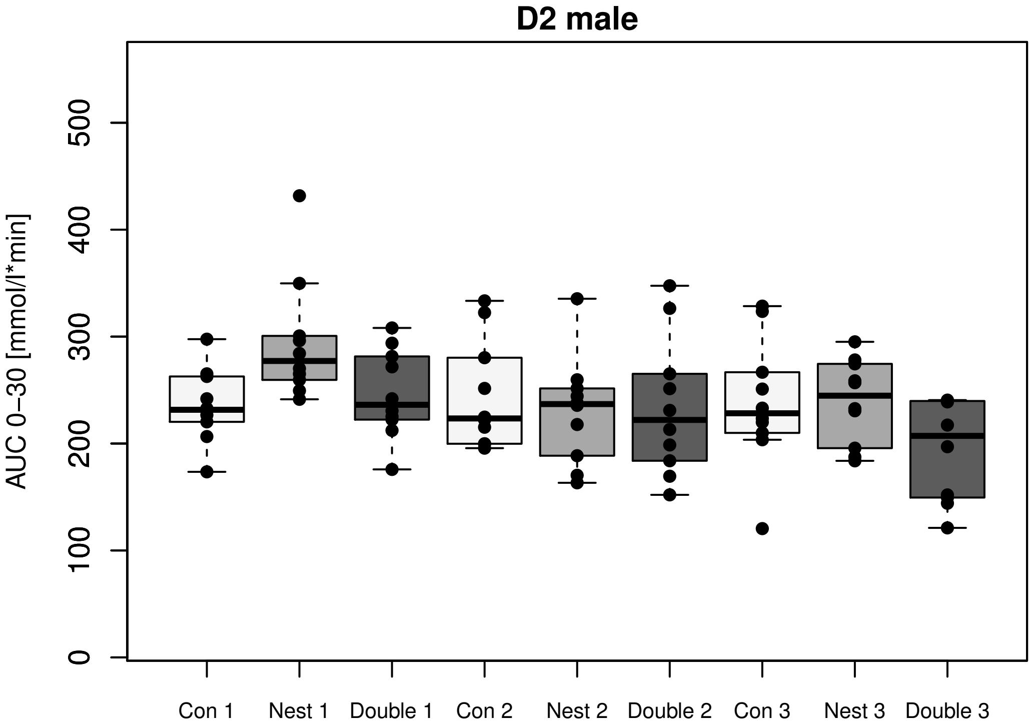
D2 male
AUC 0–30 [mmol/l*min]
0
100
200
300
400
500
Con 1
Nest 1
Double 1
Con 2
Nest 2
Double 2
Con 3
Nest 3
Double 3

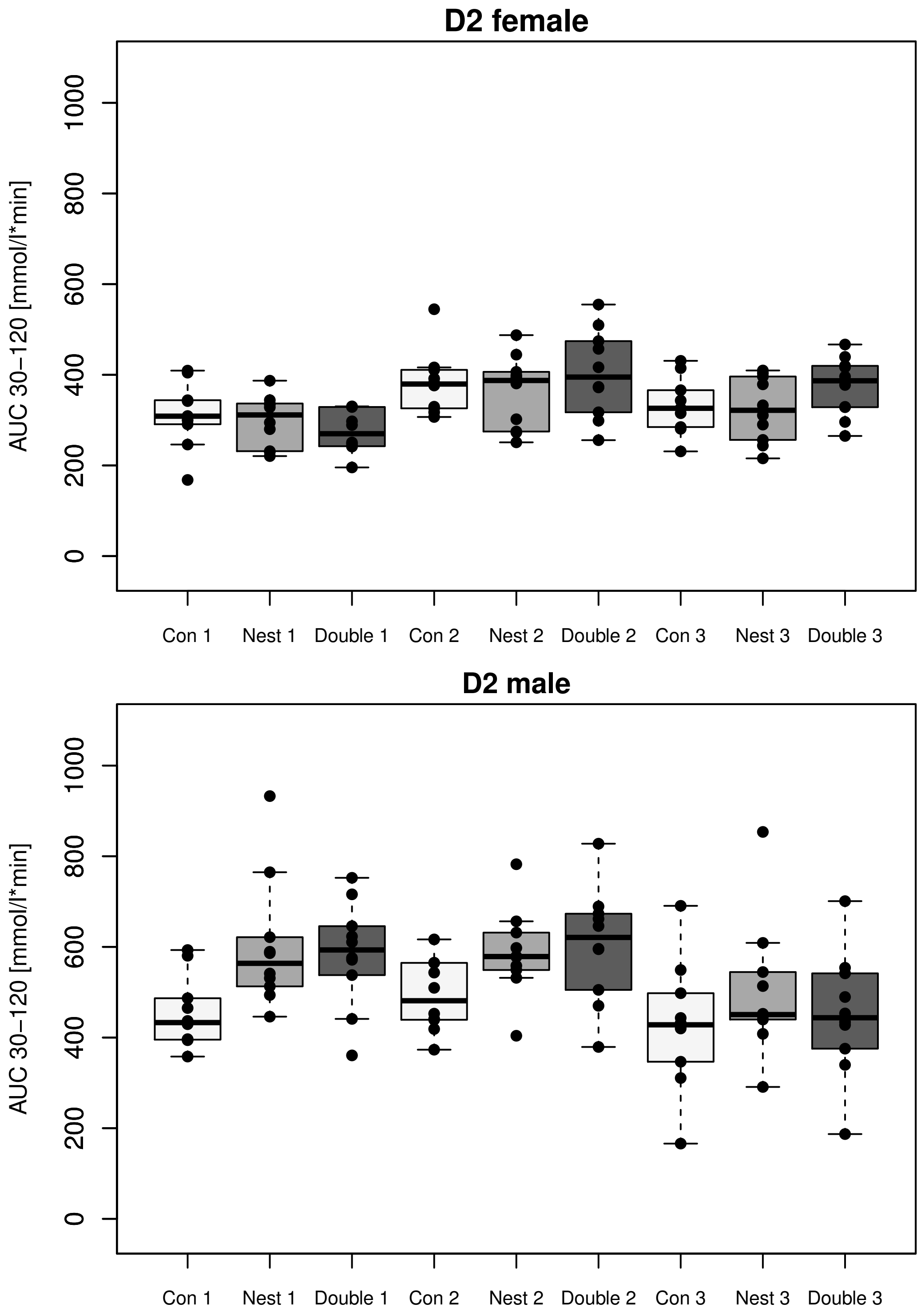
D2 female
AUC 30−120 [mmol/l*min]
0
200
400
600
800
1000
Con 1
Nest 1
Double 1
Con 2
Nest 2
Double 2
Con 3
Nest 3
Double 3
D2 male
AUC 30−120 [mmol/l*min]
0
200
400
600
800
1000
Con 1
Nest 1
Double 1
Con 2
Nest 2
Double 2
Con 3
Nest 3
Double 3

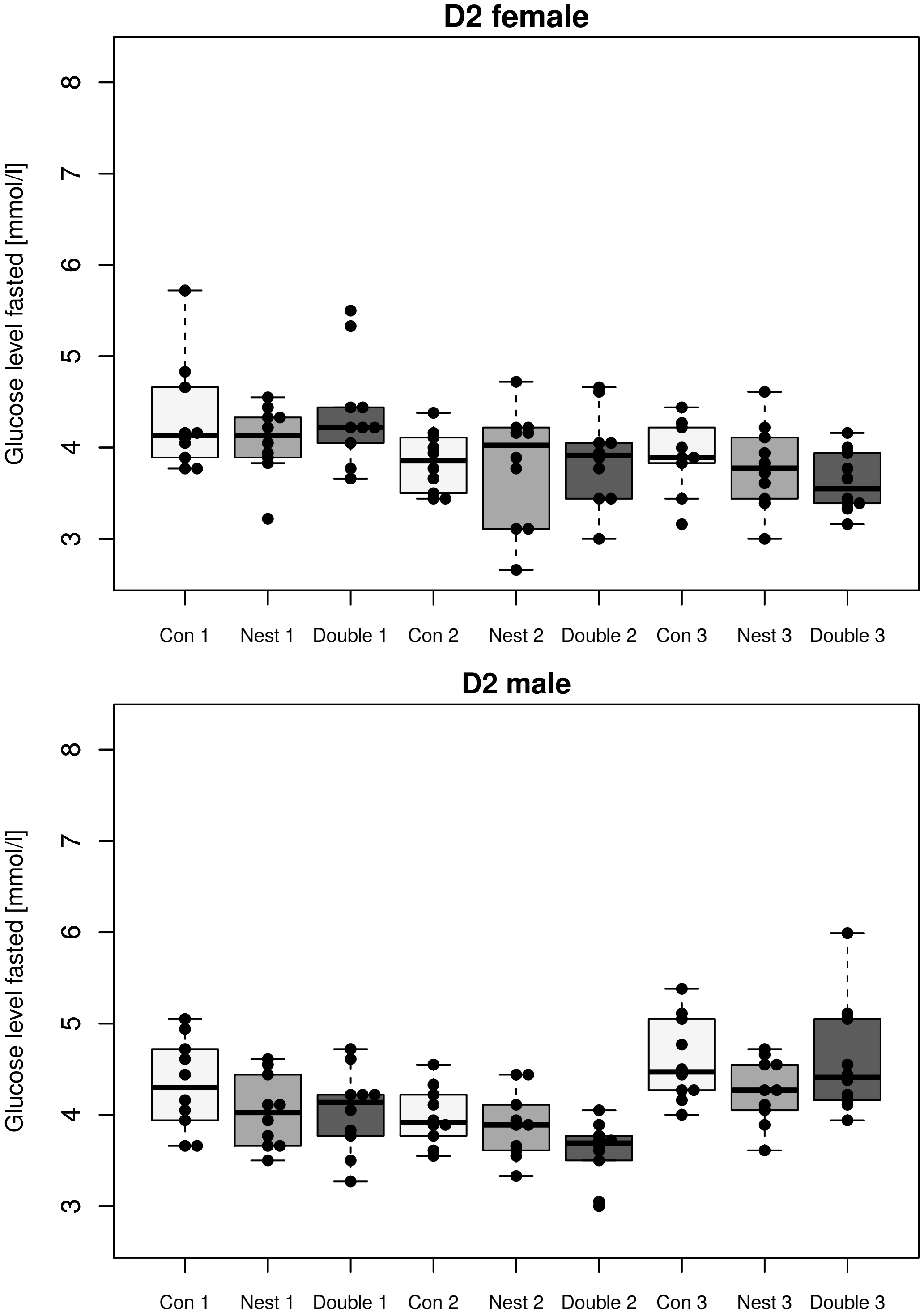
D2 female
Glucose level fasted [mmol/l]
3
4
5
6
7
8
Con 1
Nest 1
Double 1
Con 2
Nest 2
Double 2
Con 3
Nest 3
Double 3
D2 male
Glucose level fasted [mmol/l]
3
4
5
6
7
8
Con 1
Nest 1
Double 1
Con 2
Nest 2
Double 2
Con 3
Nest 3
Double 3

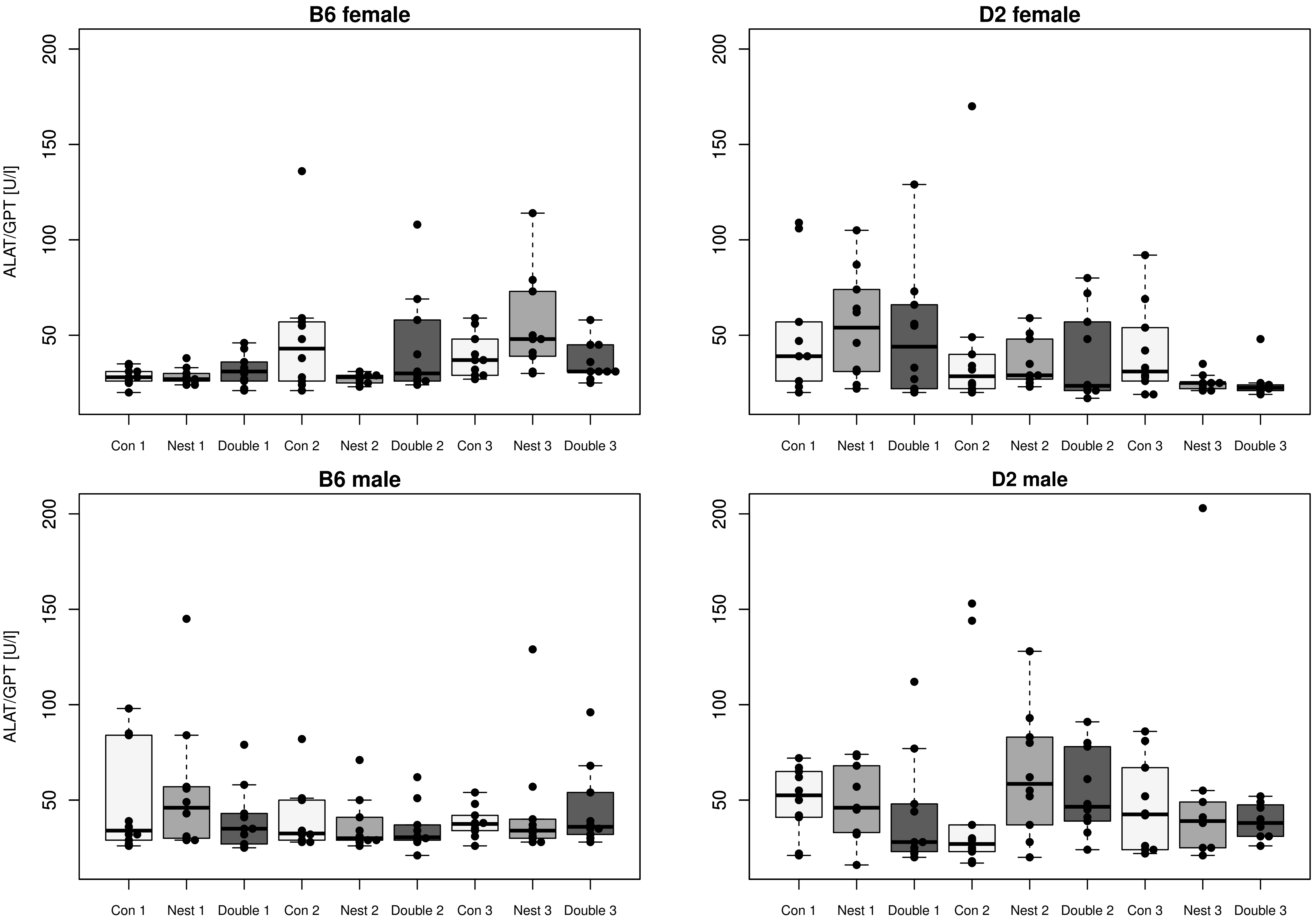
B6 female
D2 female
B6 male
D2 male
ALAT/GPT [U/l]
200
150
100
50
Con 1
Nest 1
Double 1
Con 2
Nest 2
Double 2
Con 3
Nest 3
Double 3

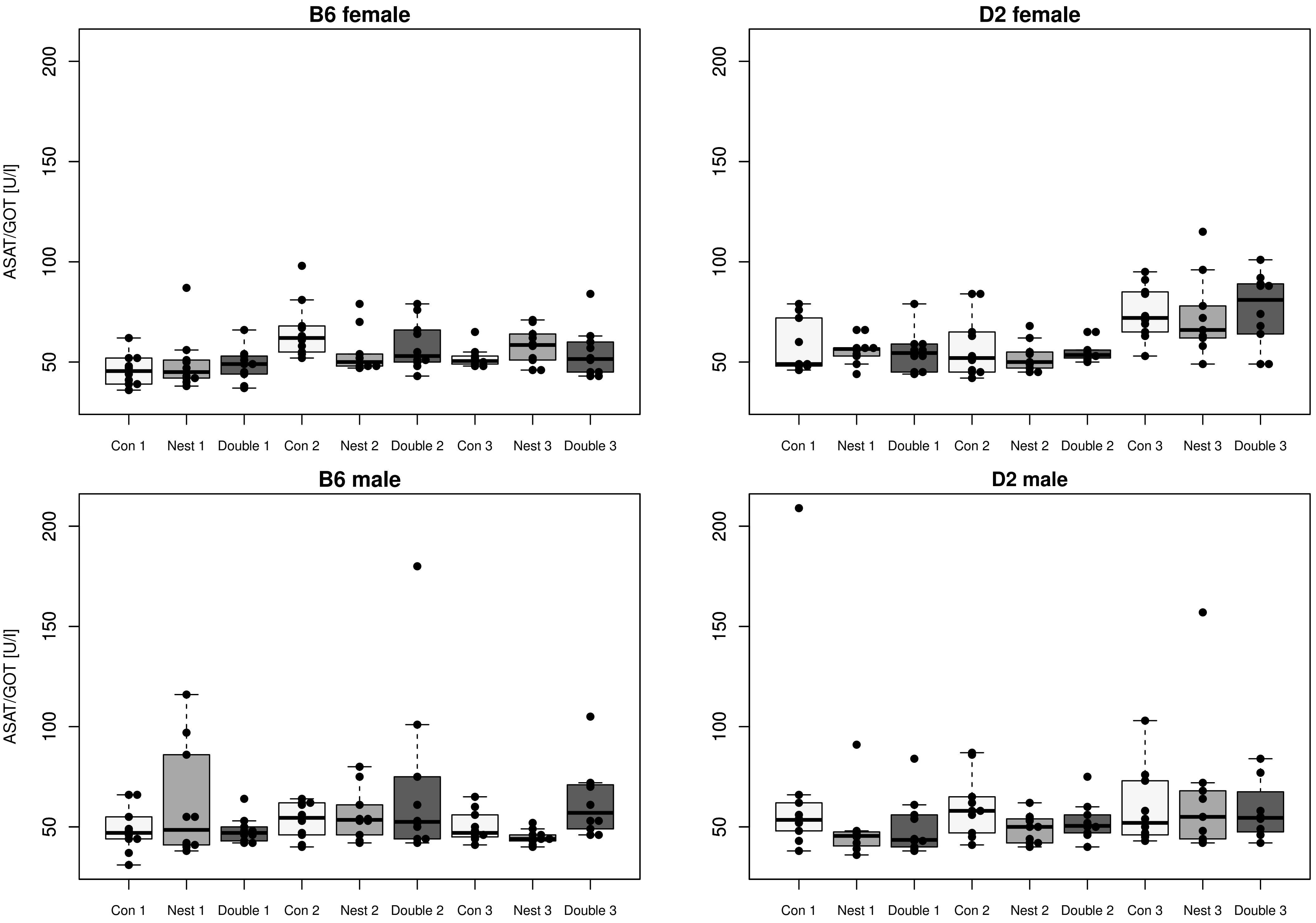
B6 female
D2 female
B6 male
D2 male
ASAT/GOT [U/l]
200
150
100
50
Con 1
Nest 1
Double 1
Con 2
Nest 2
Double 2
Con 3
Nest 3
Double 3

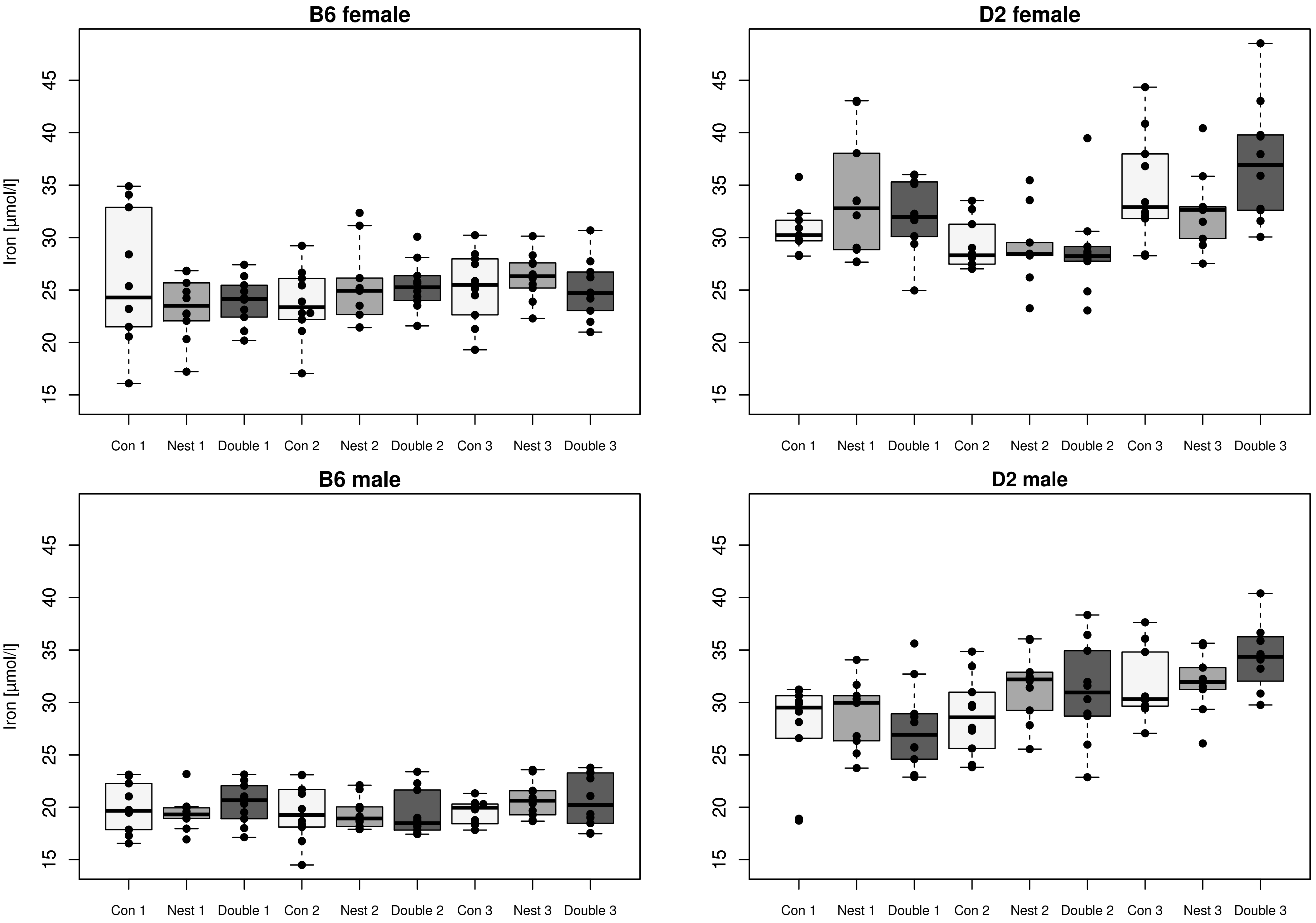
B6 female
D2 female
B6 male
D2 male
Iron [µmol/l]
15
20
25
30
35
40
45
Con 1
Nest 1
Double 1
Con 2
Nest 2
Double 2
Con 3
Nest 3
Double 3

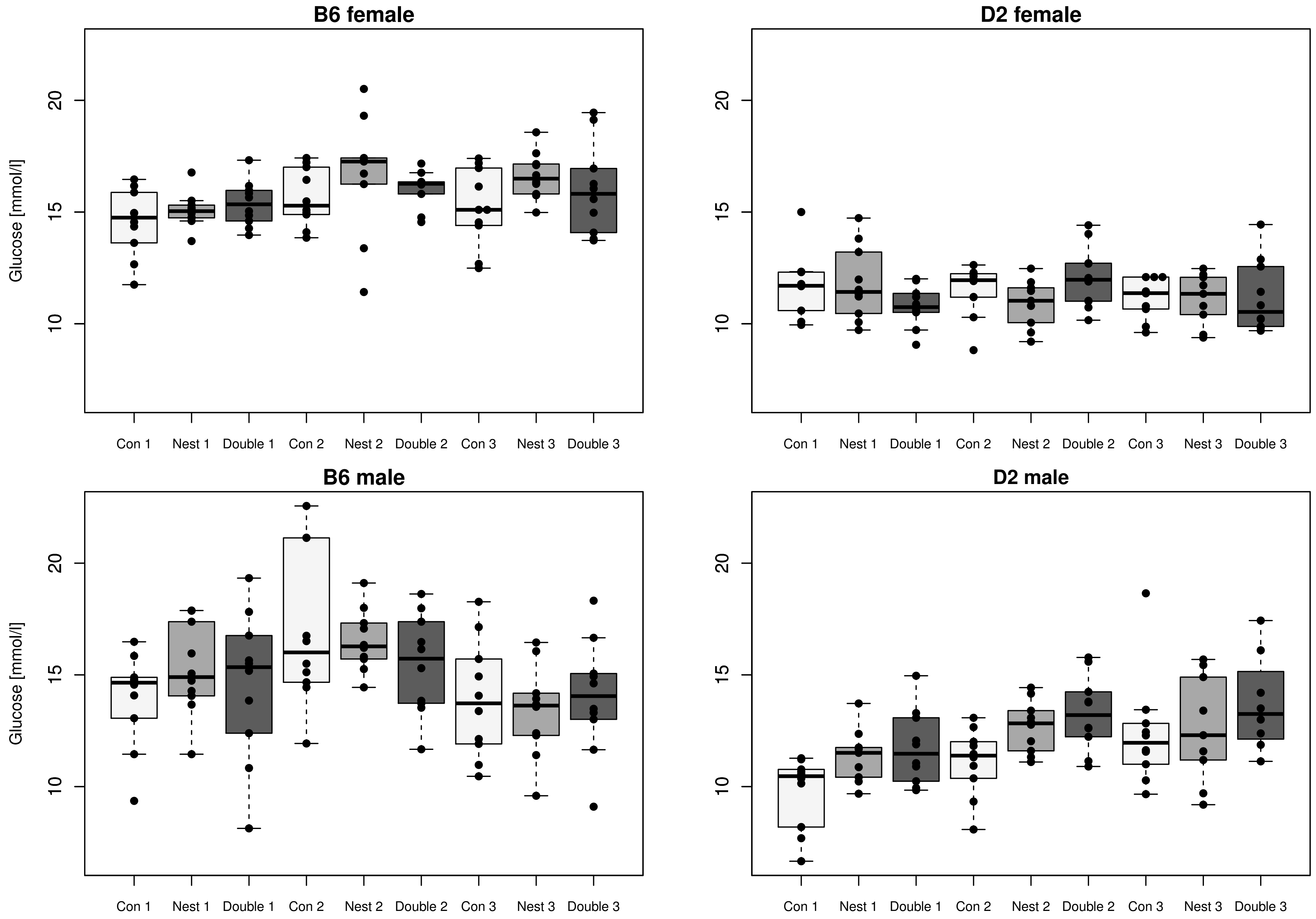
B6 female
D2 female
B6 male
D2 male
Glucose [mmol/l]
10
15
20
Con 1
Nest 1
Double 1
Con 2
Nest 2
Double 2
Con 3
Nest 3
Double 3

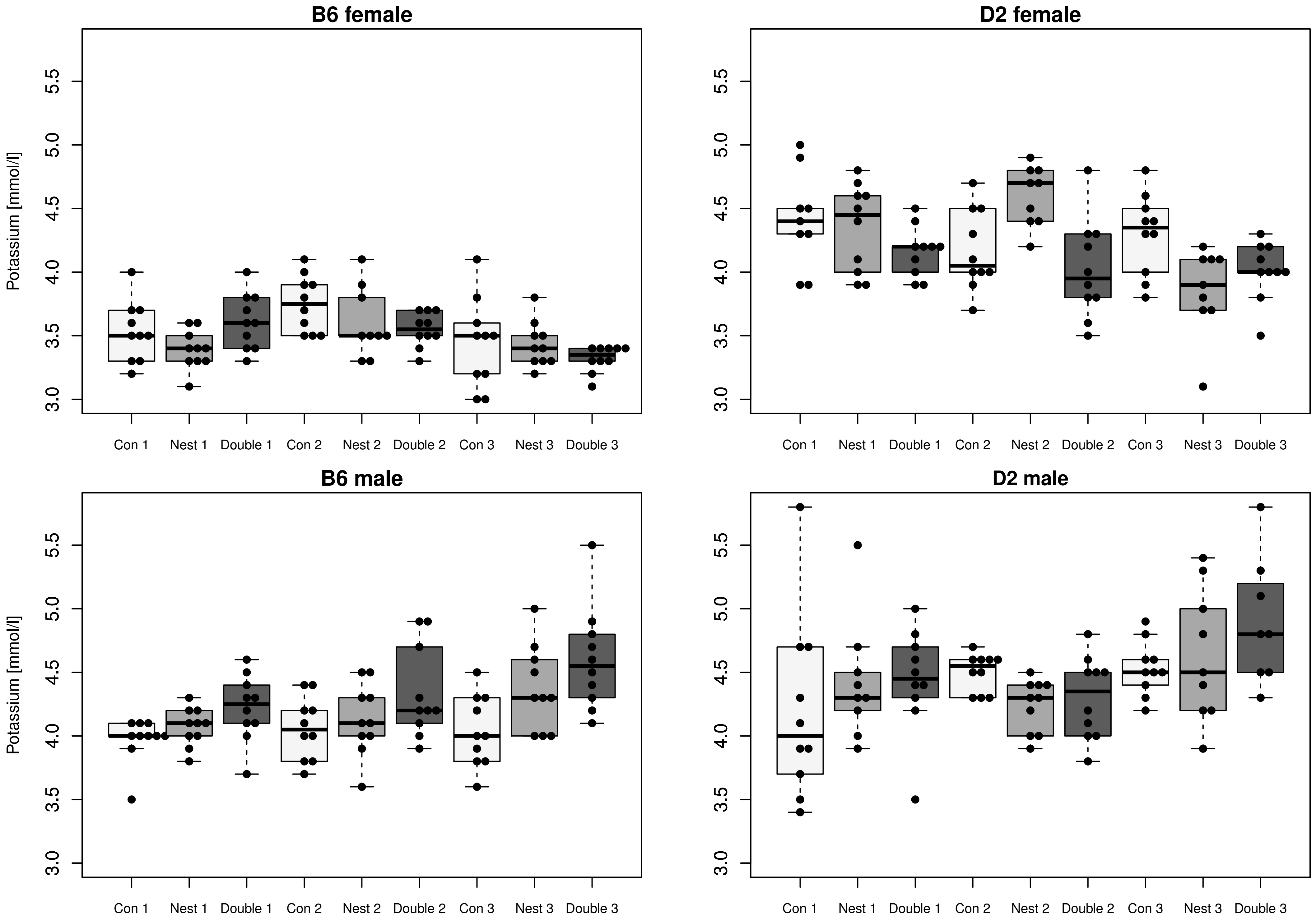

B6 female
D2 female
B6 male
D2 male
Potassium [mmol/l]
3.0
3.5
4.0
4.5
5.0
5.5
Con 1
Nest 1
Double 1
Con 2
Nest 2
Double 2
Con 3
Nest 3
Double 3

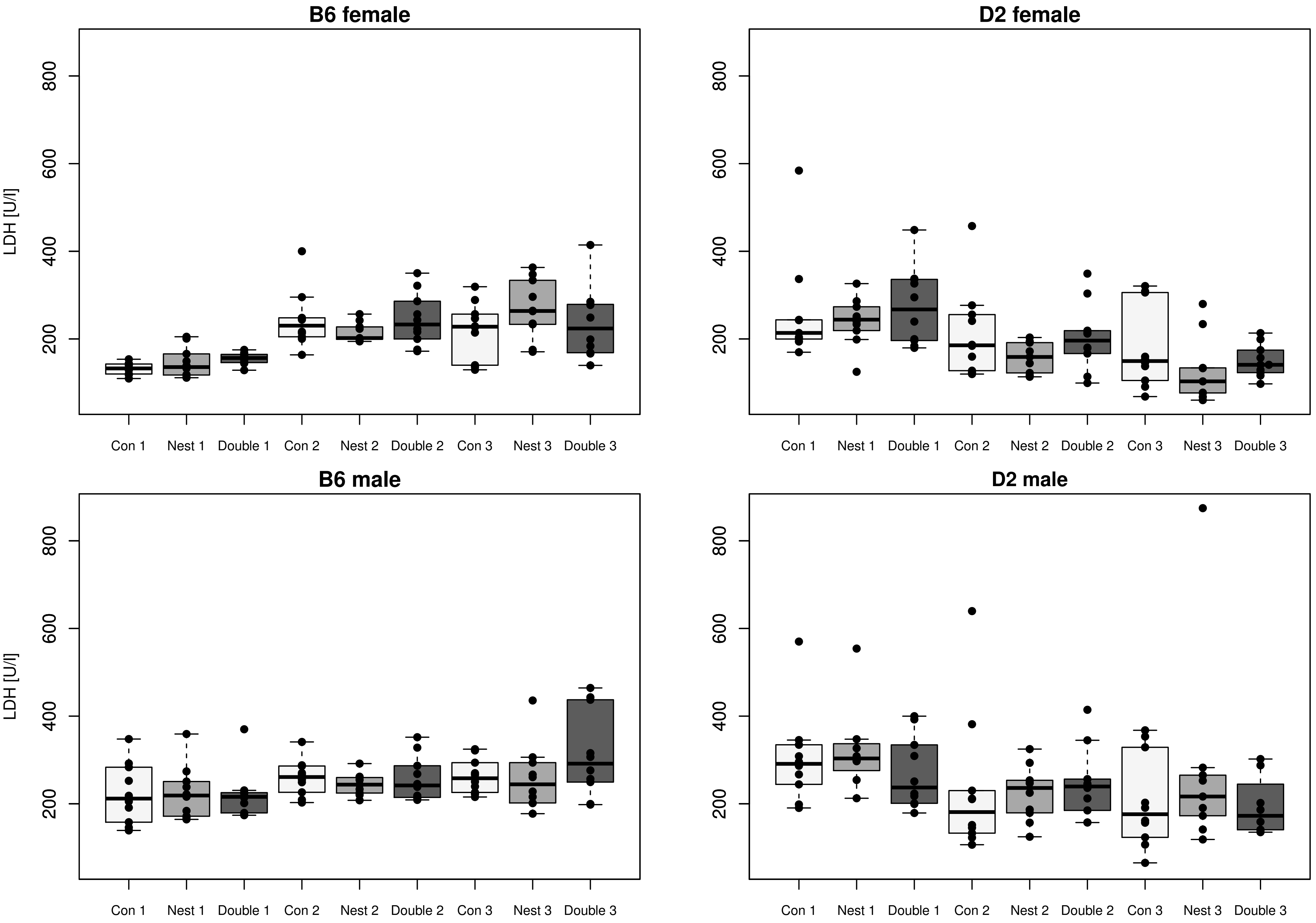
B6 female
D2 female
B6 male
D2 male
LDH [U/l]
200
400
600
800
Con 1
Nest 1
Double 1
Con 2
Nest 2
Double 2
Con 3
Nest 3
Double 3

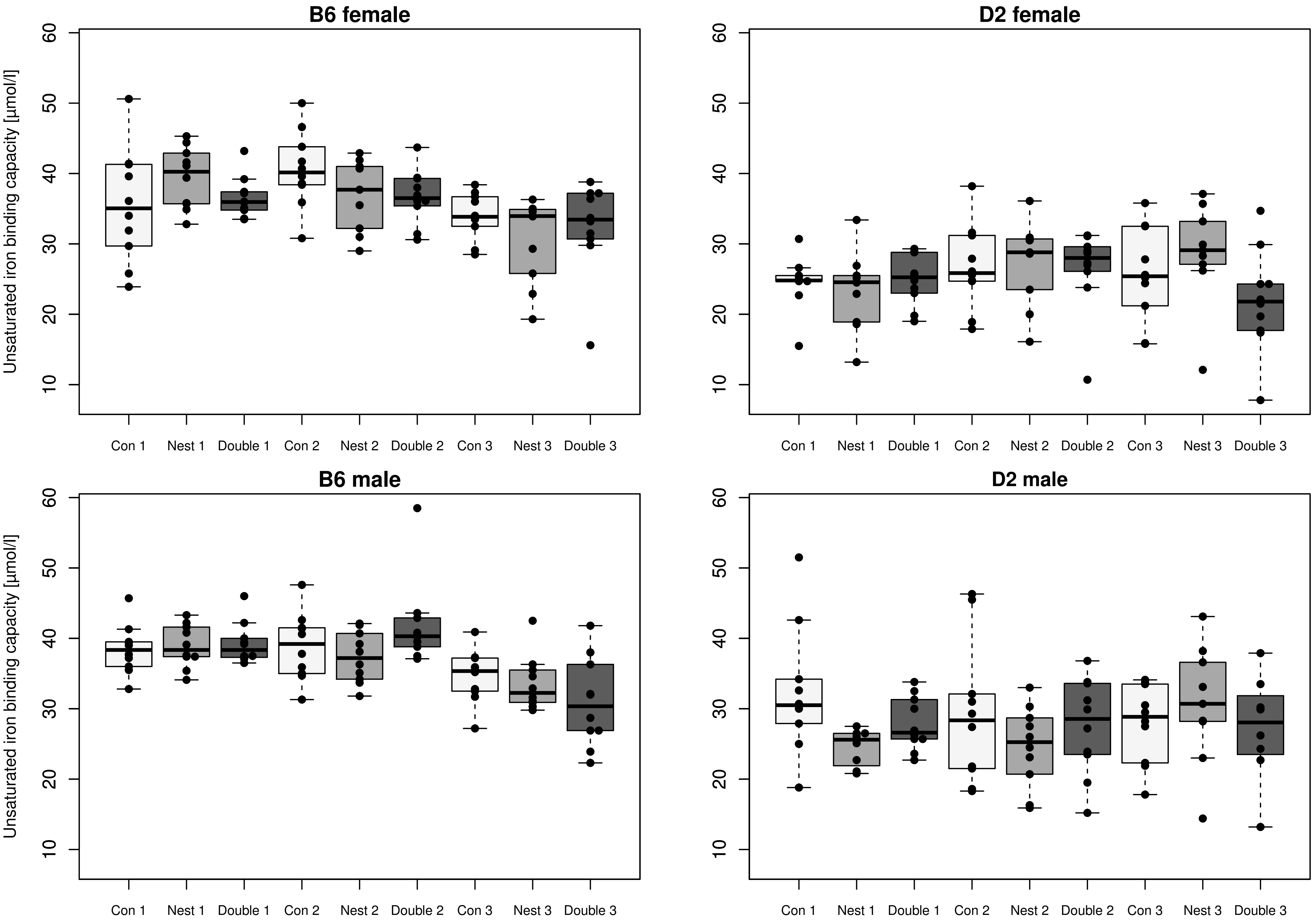

B6 female
D2 female
B6 male
D2 male
Unsaturated iron binding capacity [µmol/l]
10
20
30
40
50
60
Con 1
Nest 1
Double 1
Con 2
Nest 2
Double 2
Con 3
Nest 3
Double 3

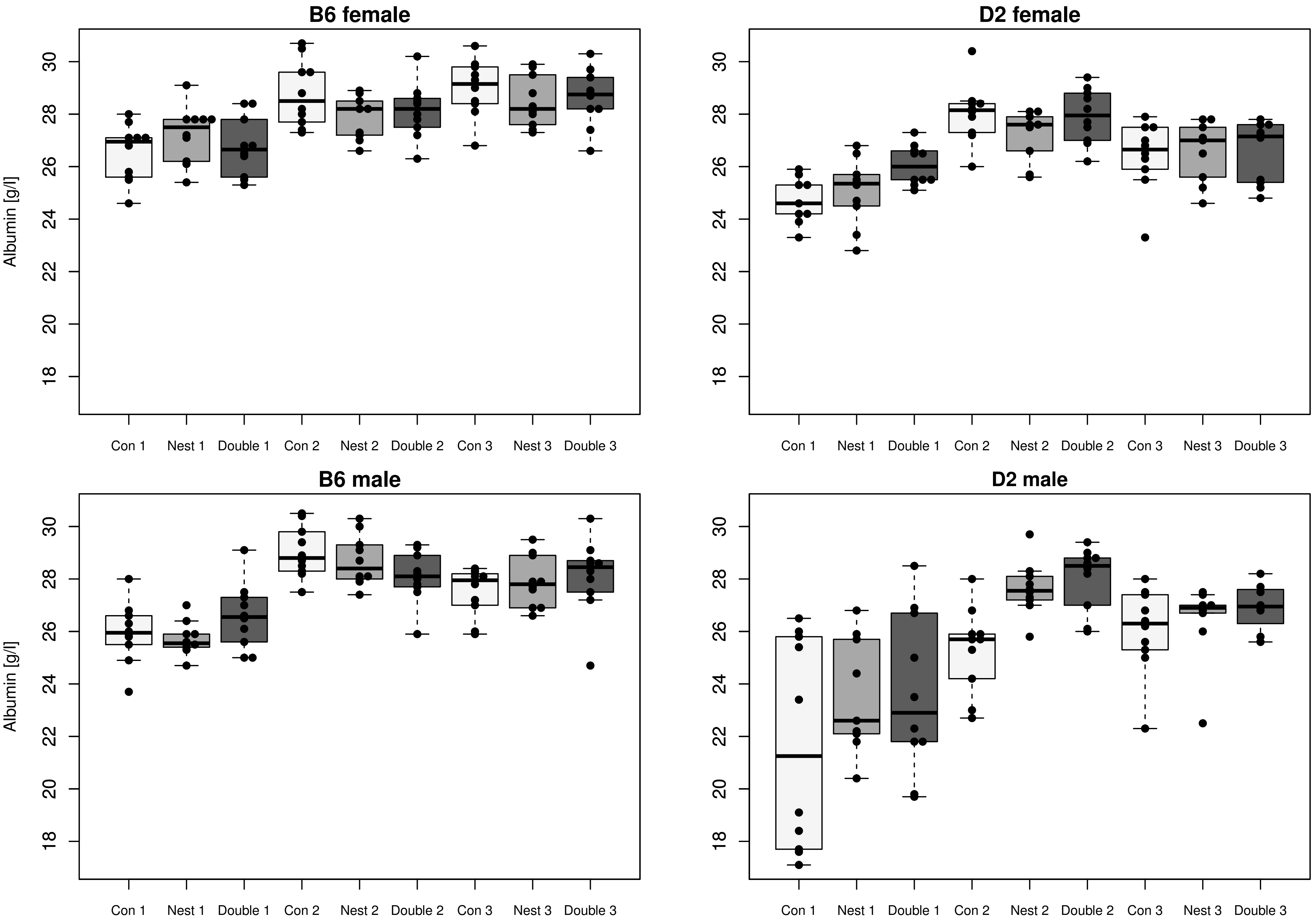
B6 female
D2 female
B6 male
D2 male
Albumin [g/l]
Albumin [g/l]
30
28
26
24
22
20
18
Con 1
Nest 1
Double 1
Con 2
Nest 2
Double 2
Con 3
Nest 3
Double 3

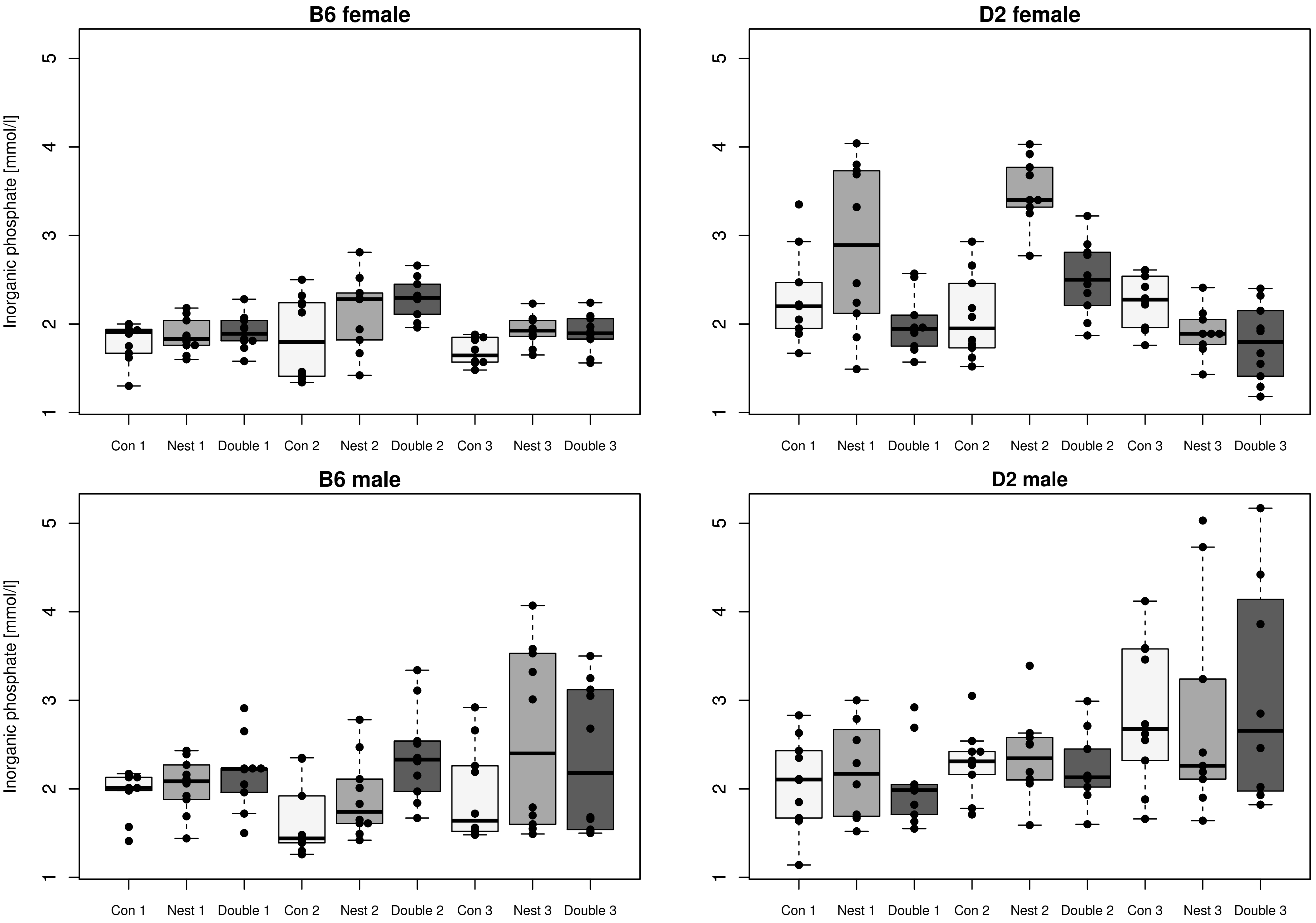
B6 female
D2 female
B6 male
D2 male
Inorganic phosphate [mmol/l]
1
2
3
4
5
Con 1
Nest 1
Double 1
Con 2
Nest 2
Double 2
Con 3
Nest 3
Double 3

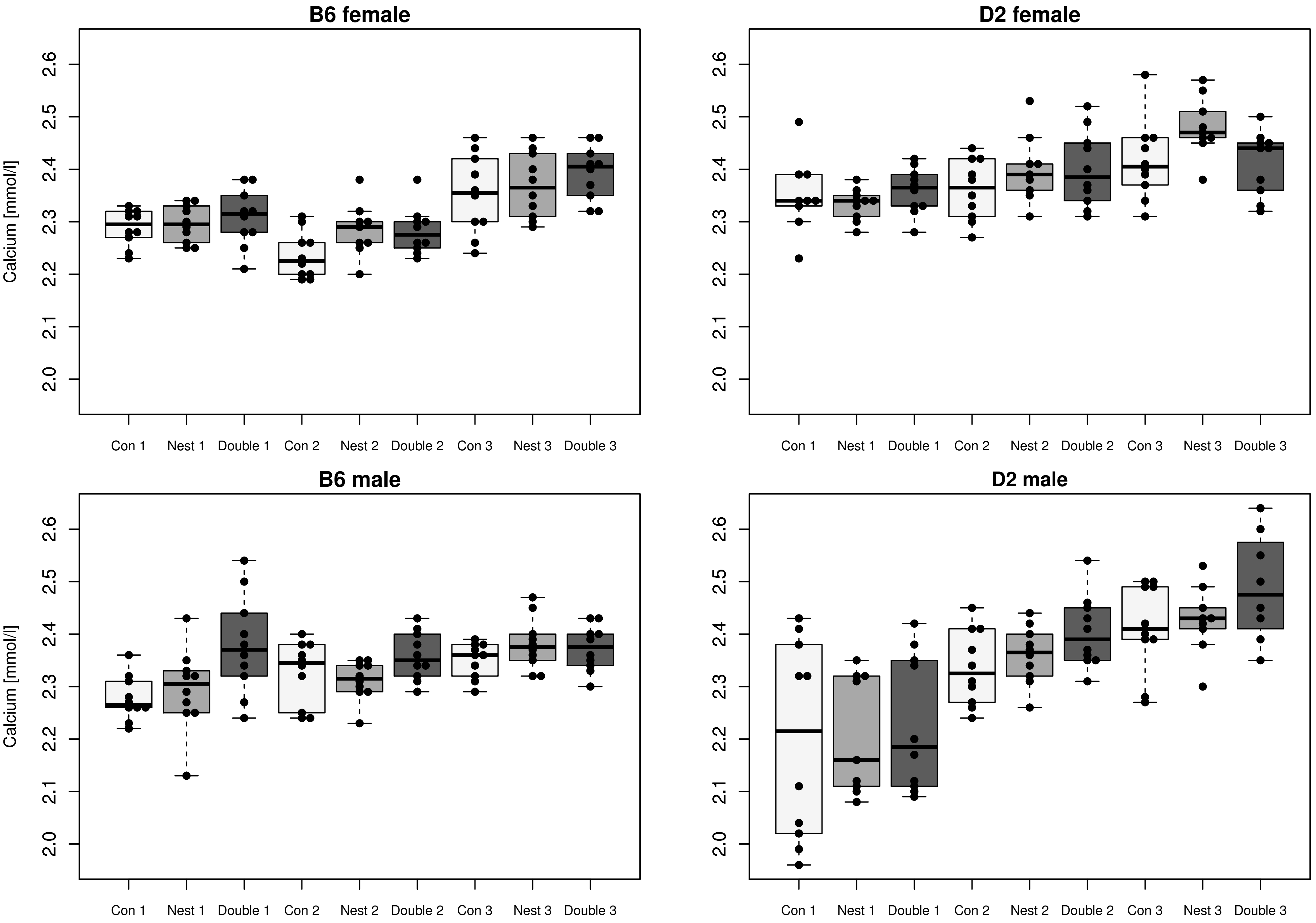
B6 female
D2 female
B6 male
D2 male
Calcium [mmol/l]
Calcium [mmol/l]
2.0
2.1
2.2
2.3
2.4
2.5
2.6
Con 1
Nest 1
Double 1
Con 2
Nest 2
Double 2
Con 3
Nest 3
Double 3

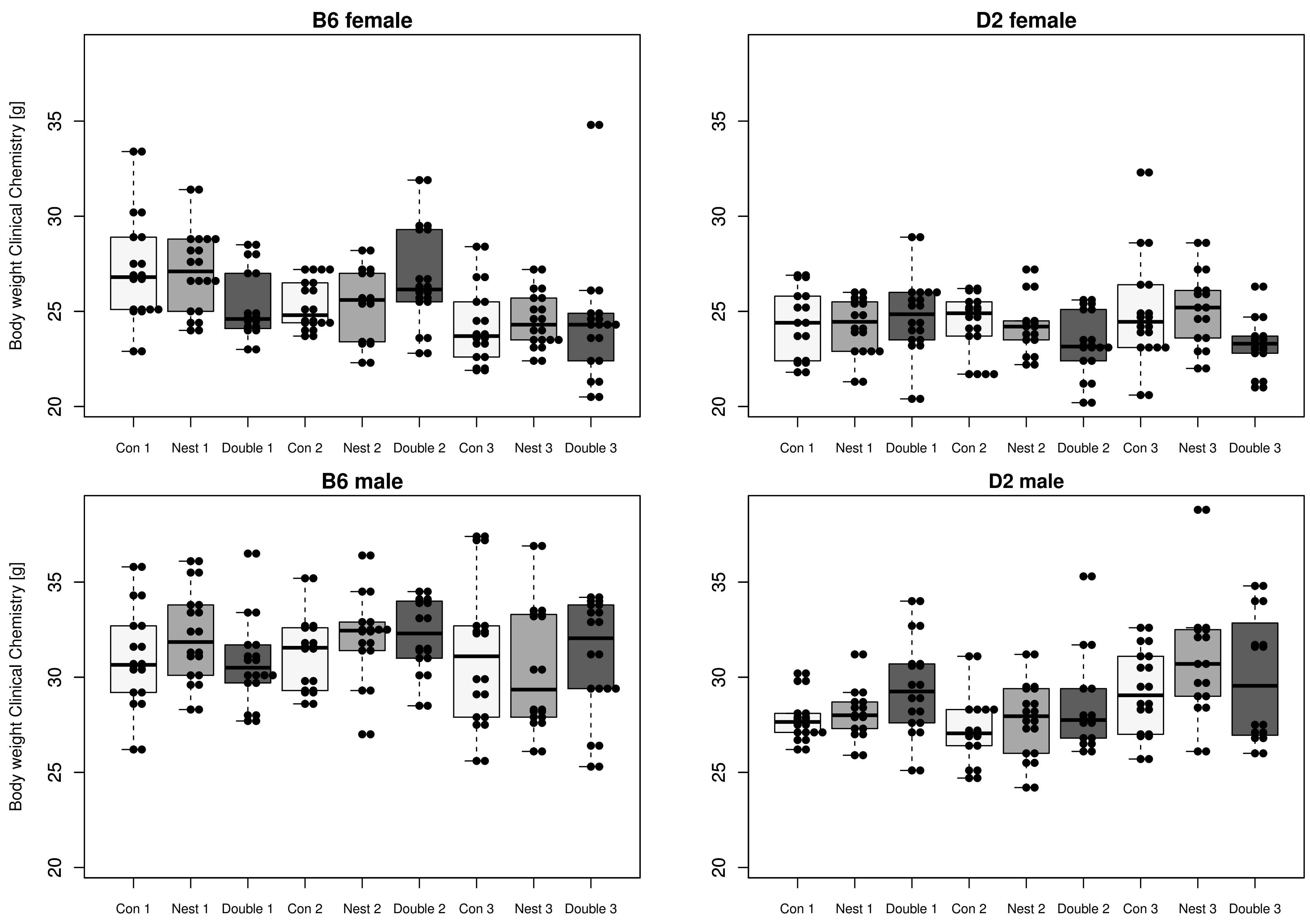

B6 female
D2 female
B6 male
D2 male
Body weight Clinical Chemistry [g]
20
25
30
35
Con 1
Nest 1
Double 1
Con 2
Nest 2
Double 2
Con 3
Nest 3
Double 3

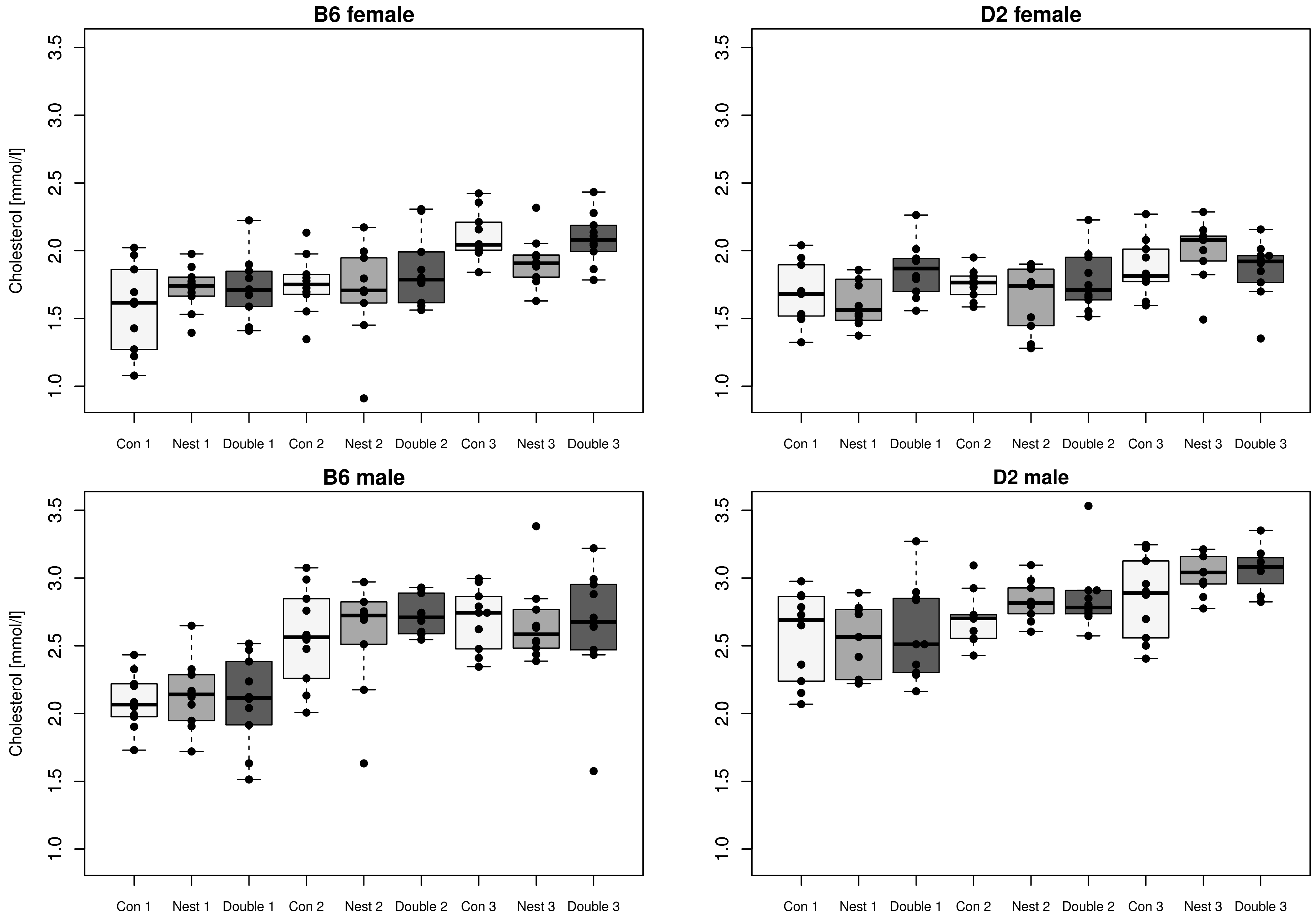
B6 female
D2 female
B6 male
D2 male
Cholesterol [mmol/l]
1.0
1.5
2.0
2.5
3.0
3.5
Con 1
Nest 1
Double 1
Con 2
Nest 2
Double 2
Con 3
Nest 3
Double 3

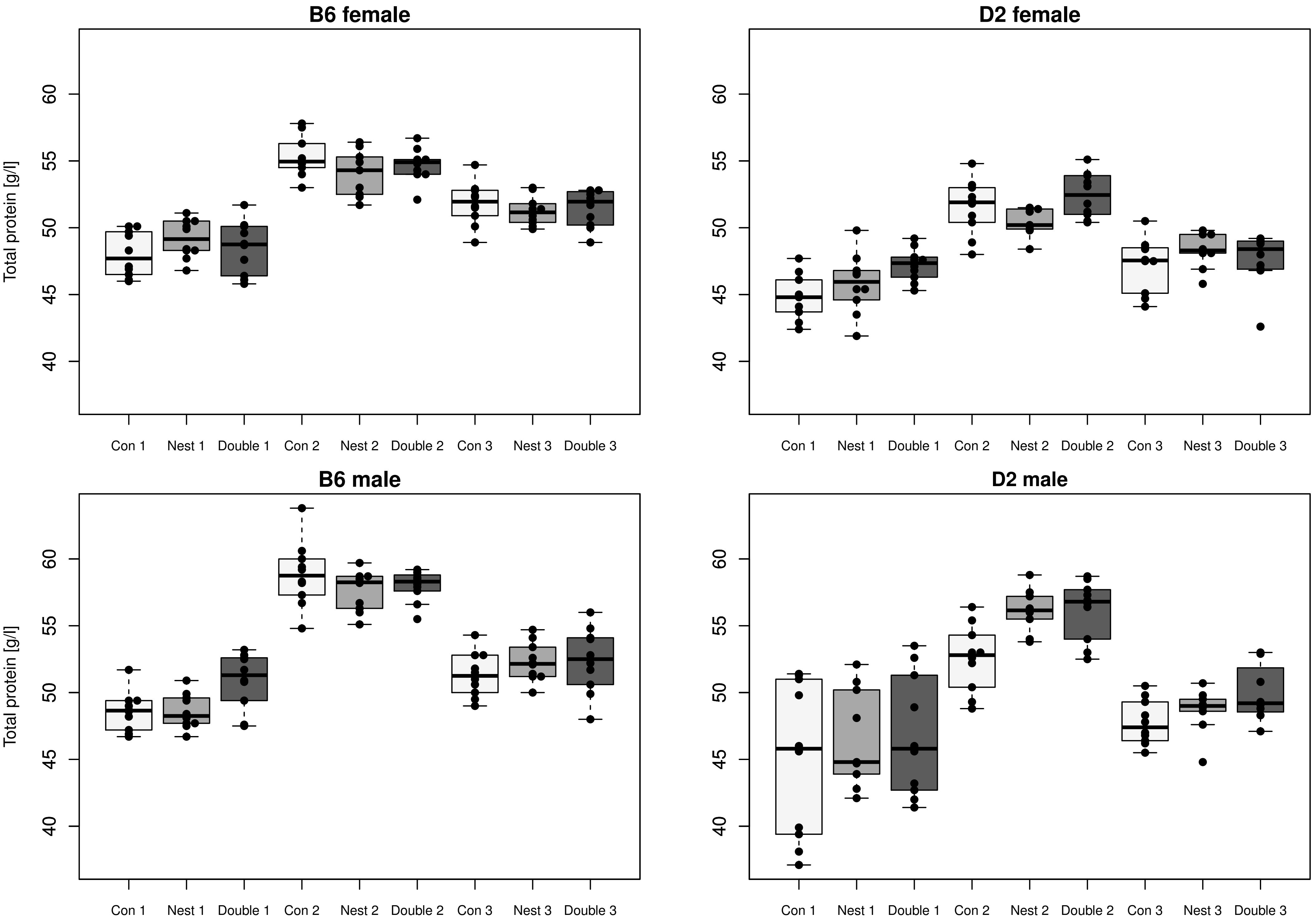

B6 female
D2 female
B6 male
D2 male
Total protein [g/l]
40
45
50
55
60
Con 1
Nest 1
Double 1
Con 2
Nest 2
Double 2
Con 3
Nest 3
Double 3

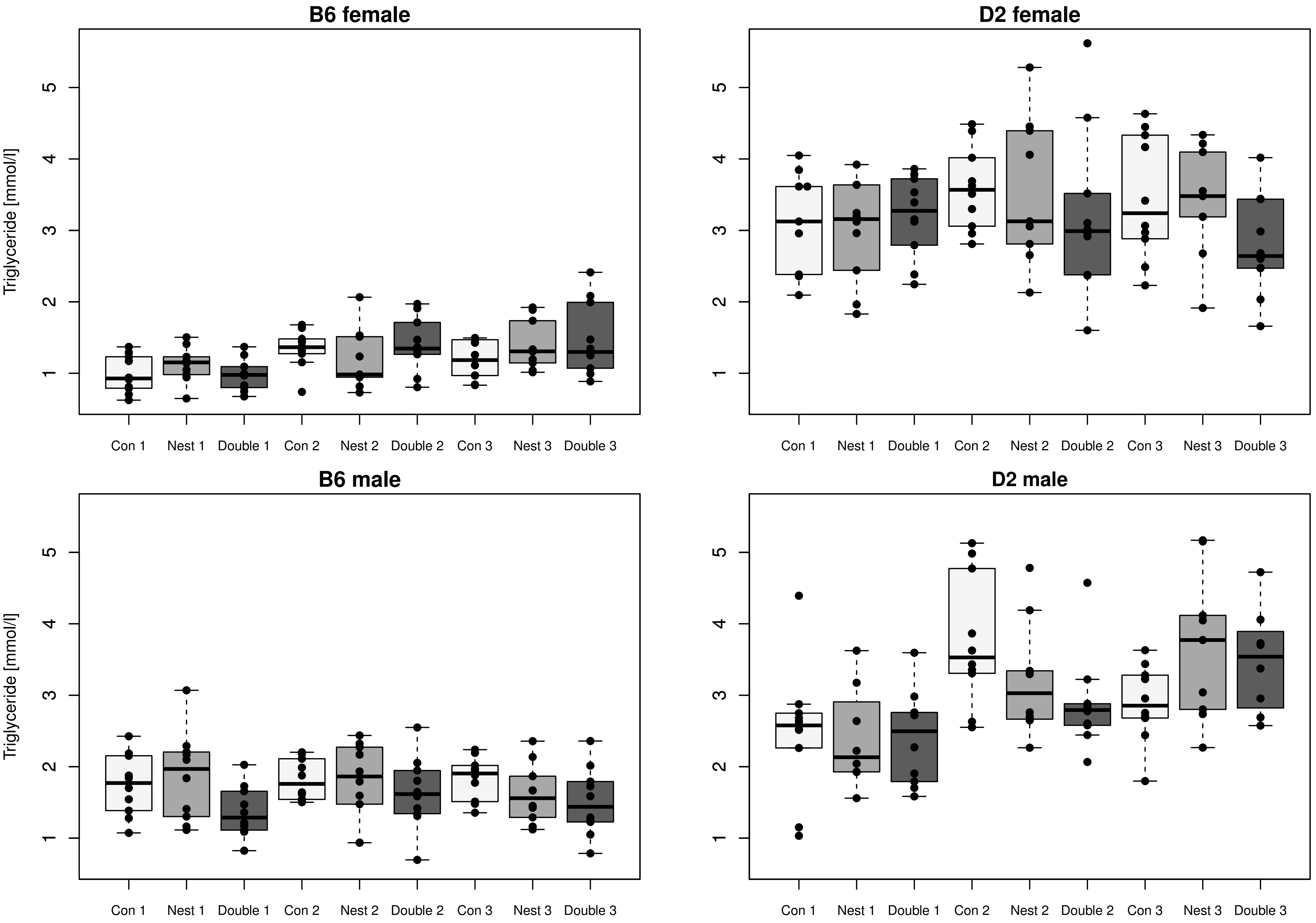

B6 female
D2 female
B6 male
D2 male
Triglyceride [mmol/l]
1
2
3
4
5
Con 1
Nest 1
Double 1
Con 2
Nest 2
Double 2
Con 3
Nest 3
Double 3

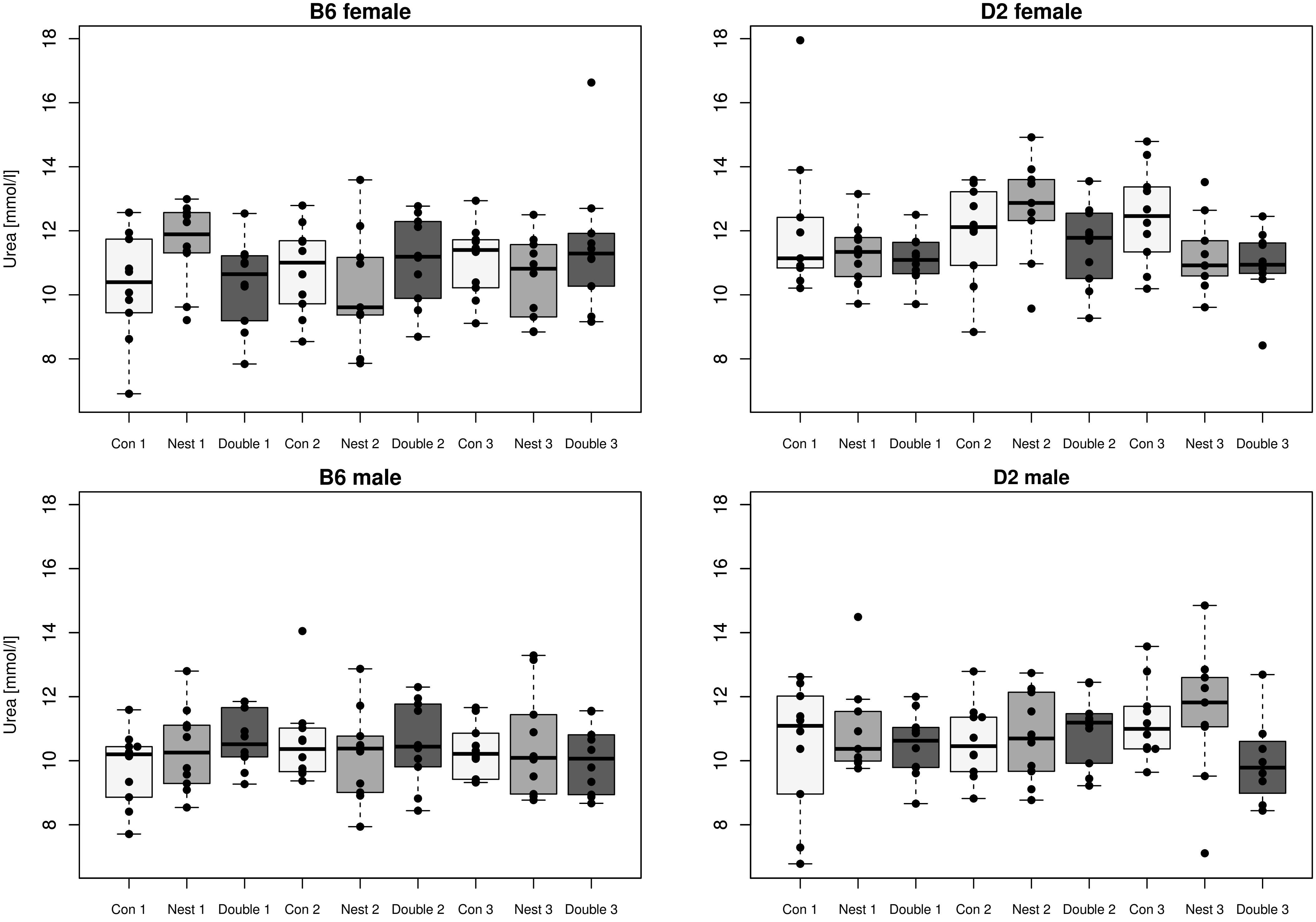
B6 female
D2 female
B6 male
D2 male
Urea [mmol/l]
8
10
12
14
16
18
Con 1
Nest 1
Double 1
Con 2
Nest 2
Double 2
Con 3
Nest 3
Double 3

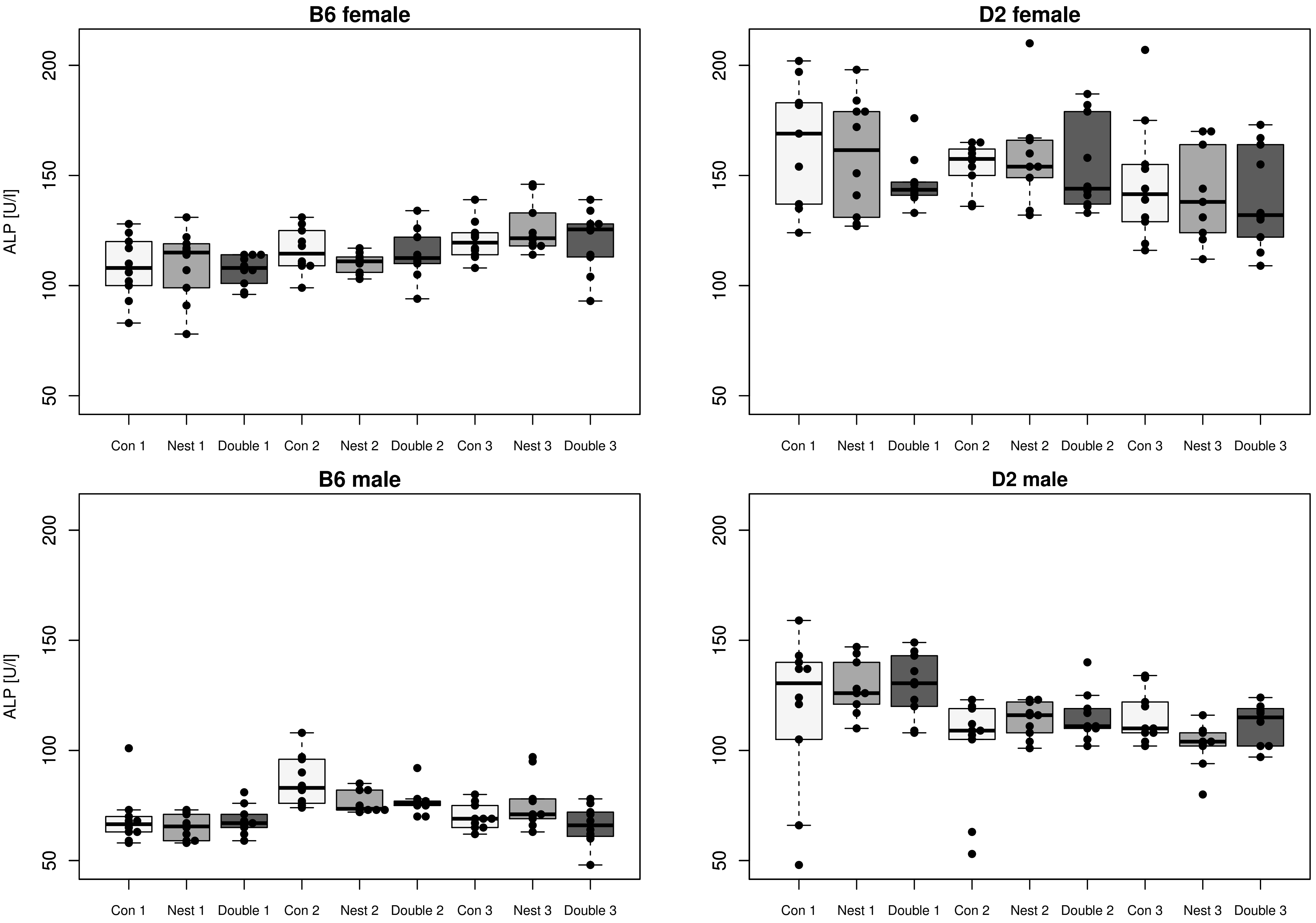

B6 female
D2 female
B6 male
D2 male
ALP [U/l]
50
100
150
200
Con 1
Nest 1
Double 1
Con 2
Nest 2
Double 2
Con 3
Nest 3
Double 3

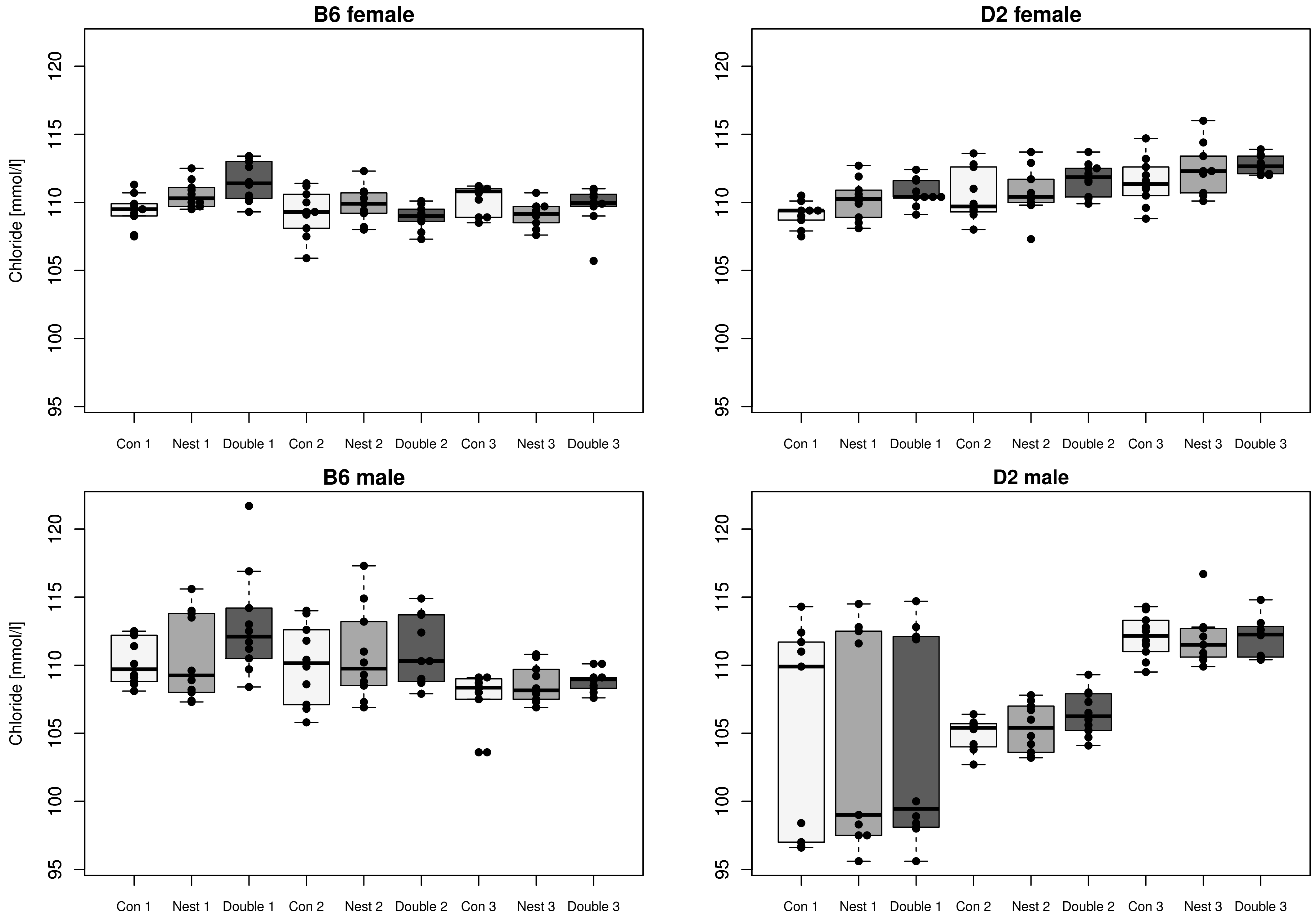
B6 female
D2 female
B6 male
D2 male
Chloride [mmol/l]
95
100
105
110
115
120
Con 1
Nest 1
Double 1
Con 2
Nest 2
Double 2
Con 3
Nest 3
Double 3

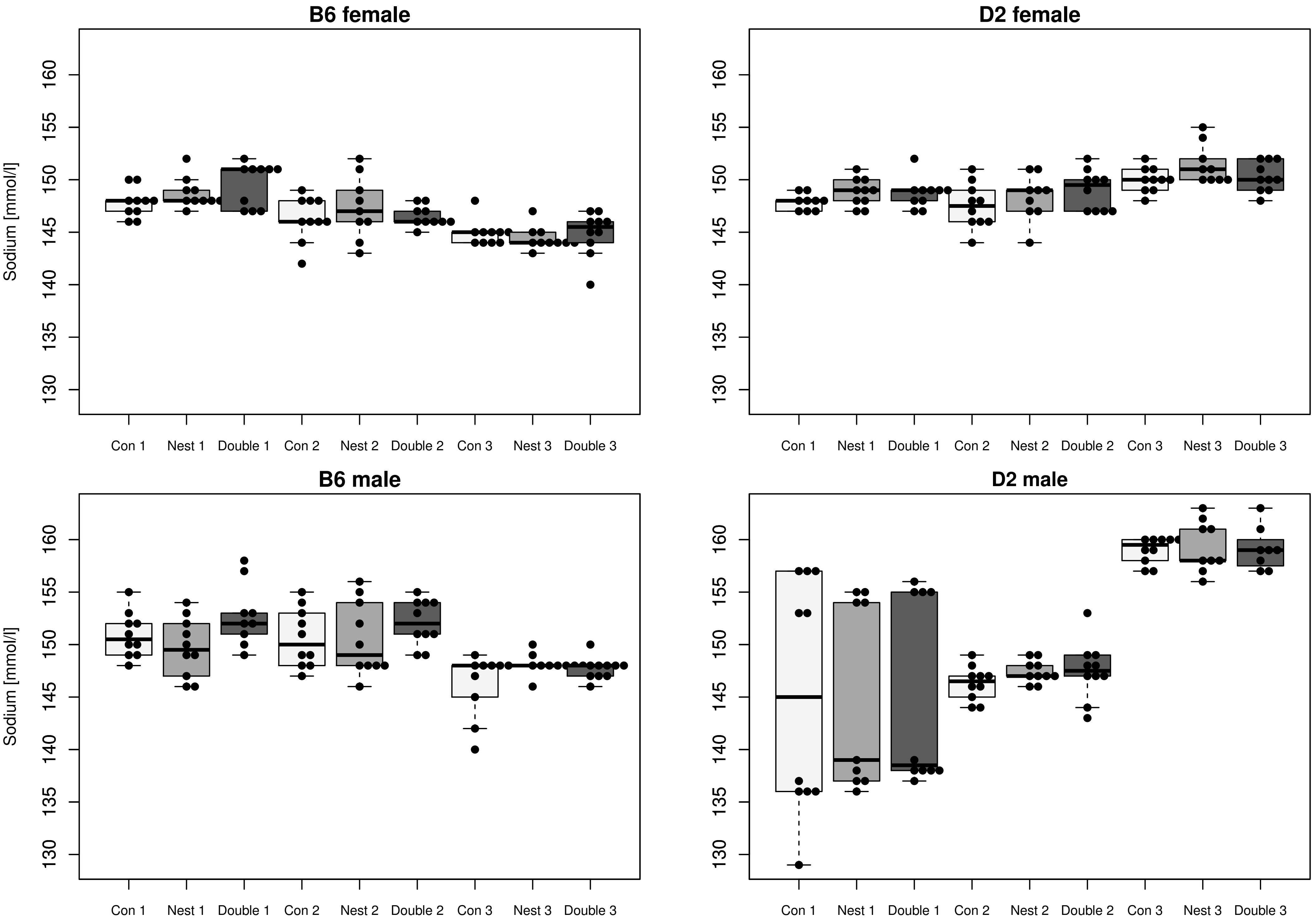

B6 female
D2 female
B6 male
D2 male
Sodium [mmol/l]
130
135
140
145
150
155
160
Con 1
Nest 1
Double 1
Con 2
Nest 2
Double 2
Con 3
Nest 3
Double 3

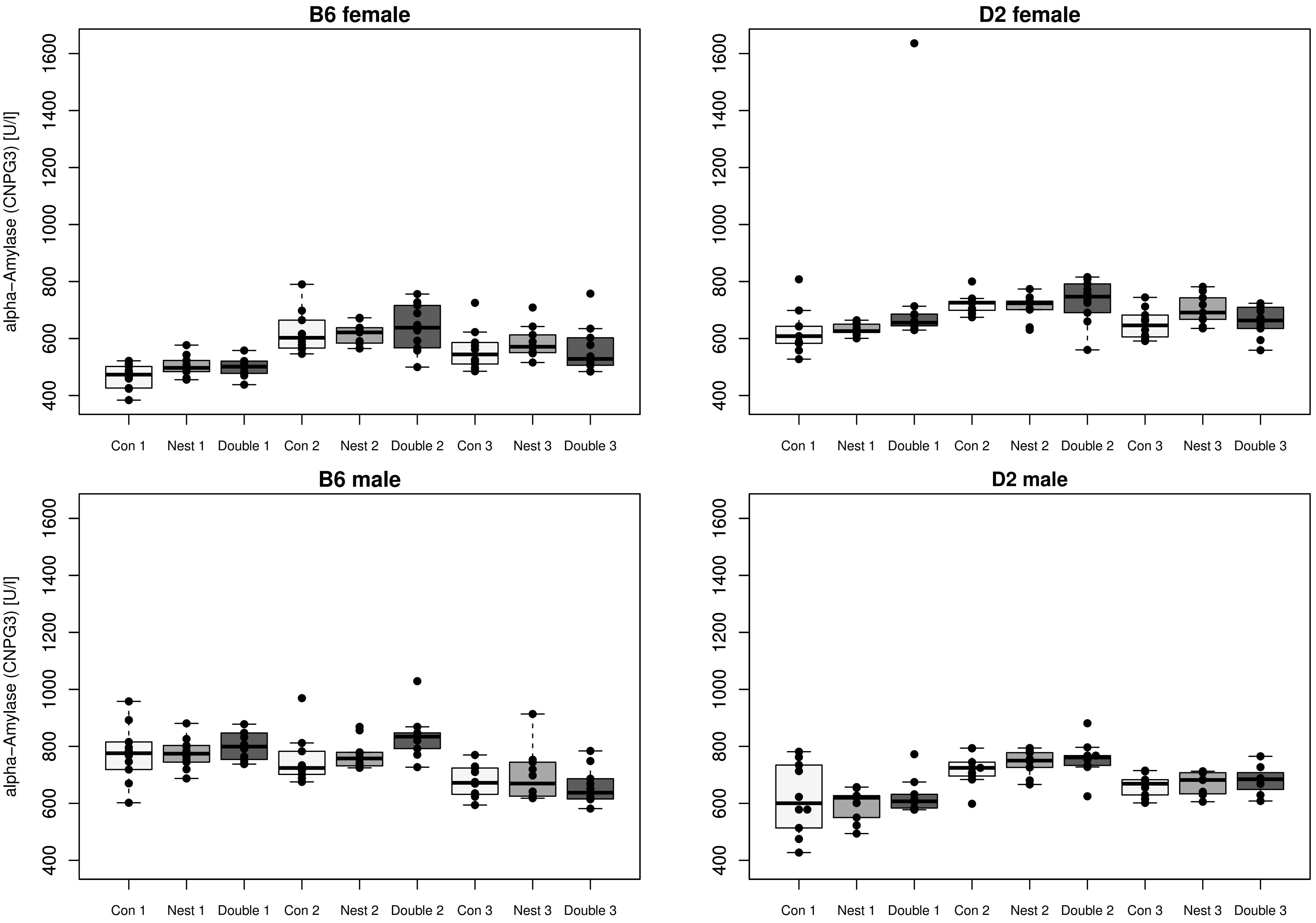

B6 female
D2 female
B6 male
D2 male
alpha−Amylase (CNPG3) [U/l]
400
600
800
1000
1200
1400
1600
Con 1
Nest 1
Double 1
Con 2
Nest 2
Double 2
Con 3
Nest 3
Double 3

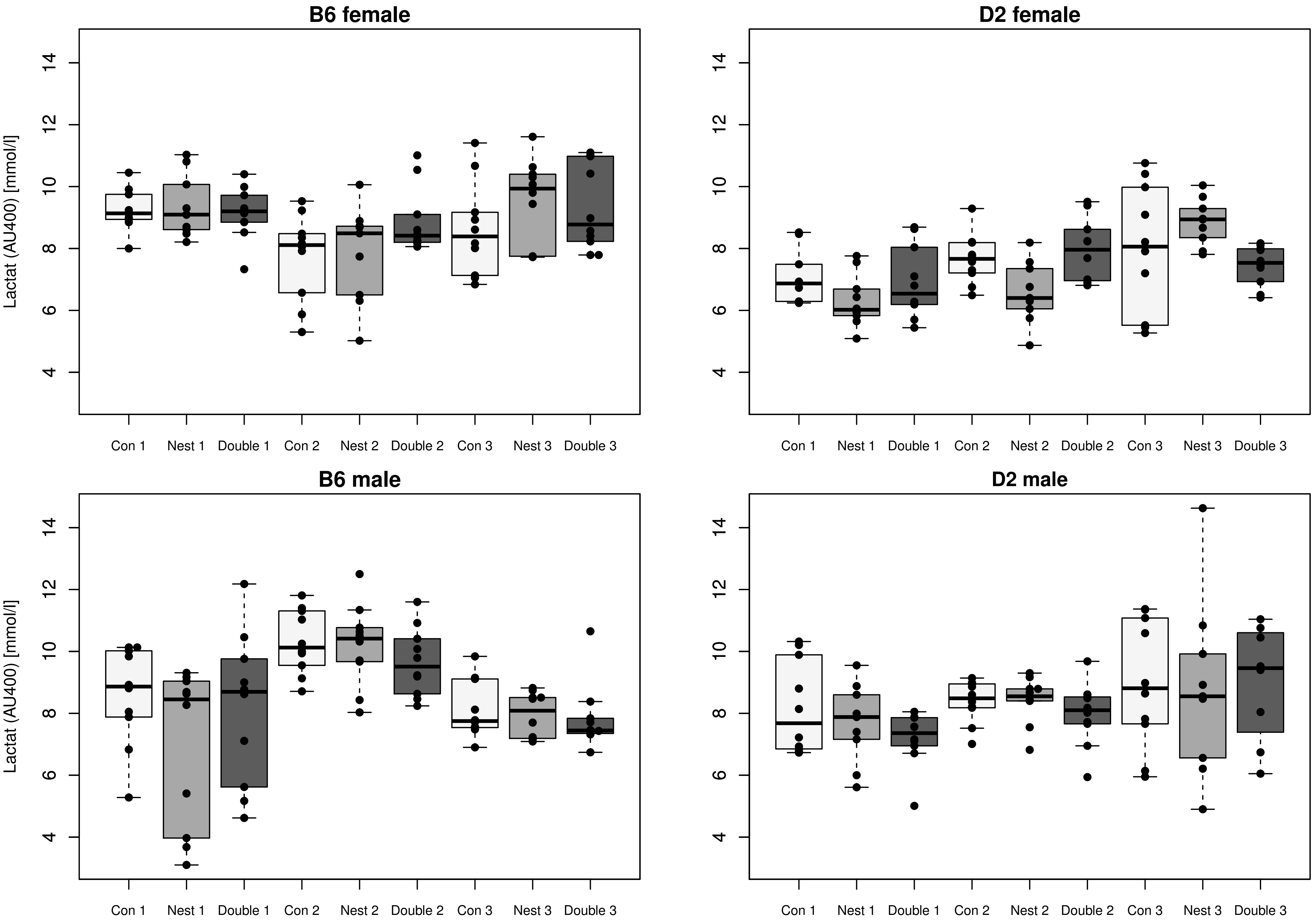
B6 female
D2 female
B6 male
D2 male
Lactat (AU400) [mmol/l]
4
6
8
10
12
14
Con 1
Nest 1
Double 1
Con 2
Nest 2
Double 2
Con 3
Nest 3
Double 3

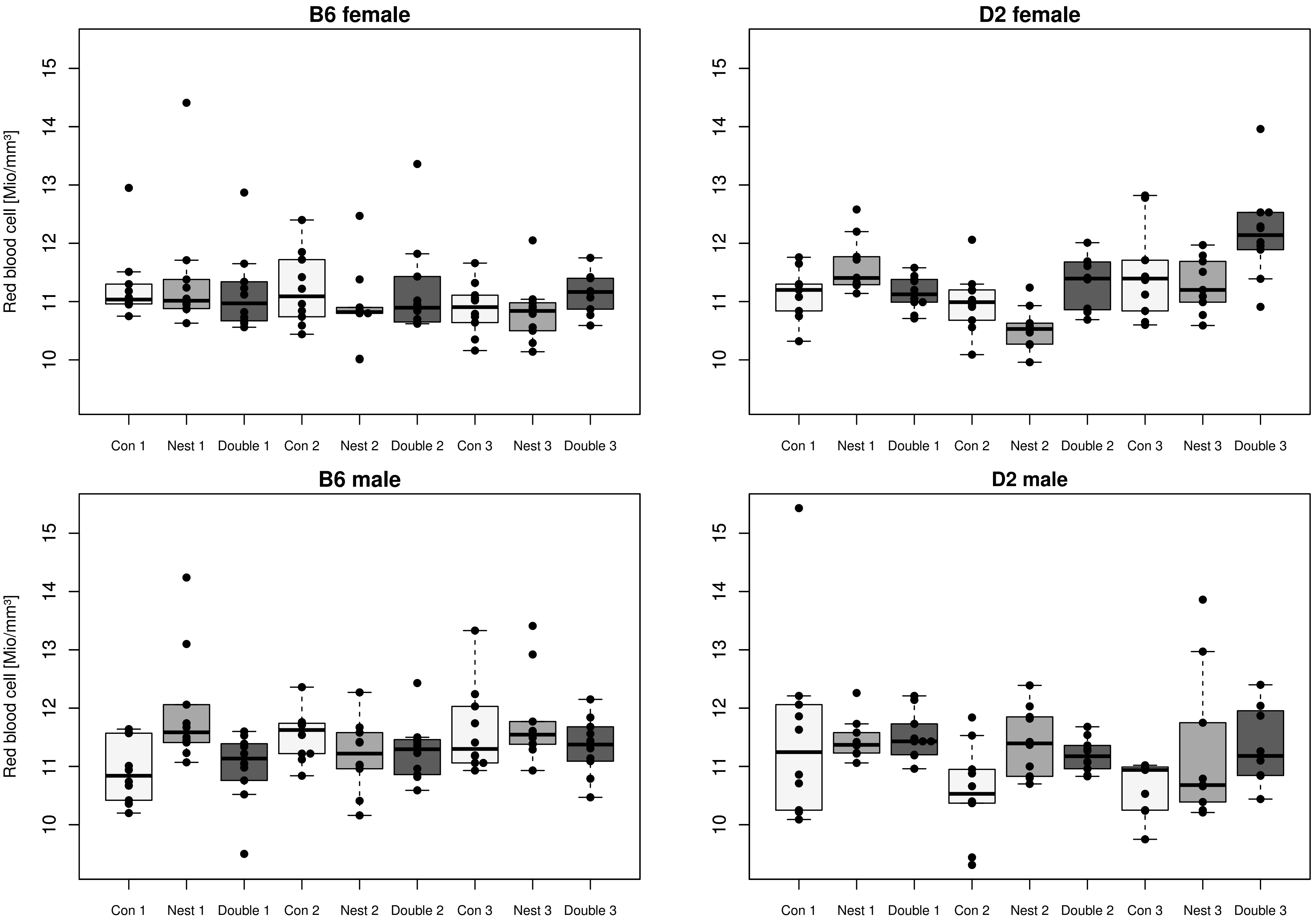
B6 female
D2 female
B6 male
D2 male
Red blood cell [Mio/mm³]
10
11
12
13
14
15
Con 1
Nest 1
Double 1
Con 2
Nest 2
Double 2
Con 3
Nest 3
Double 3

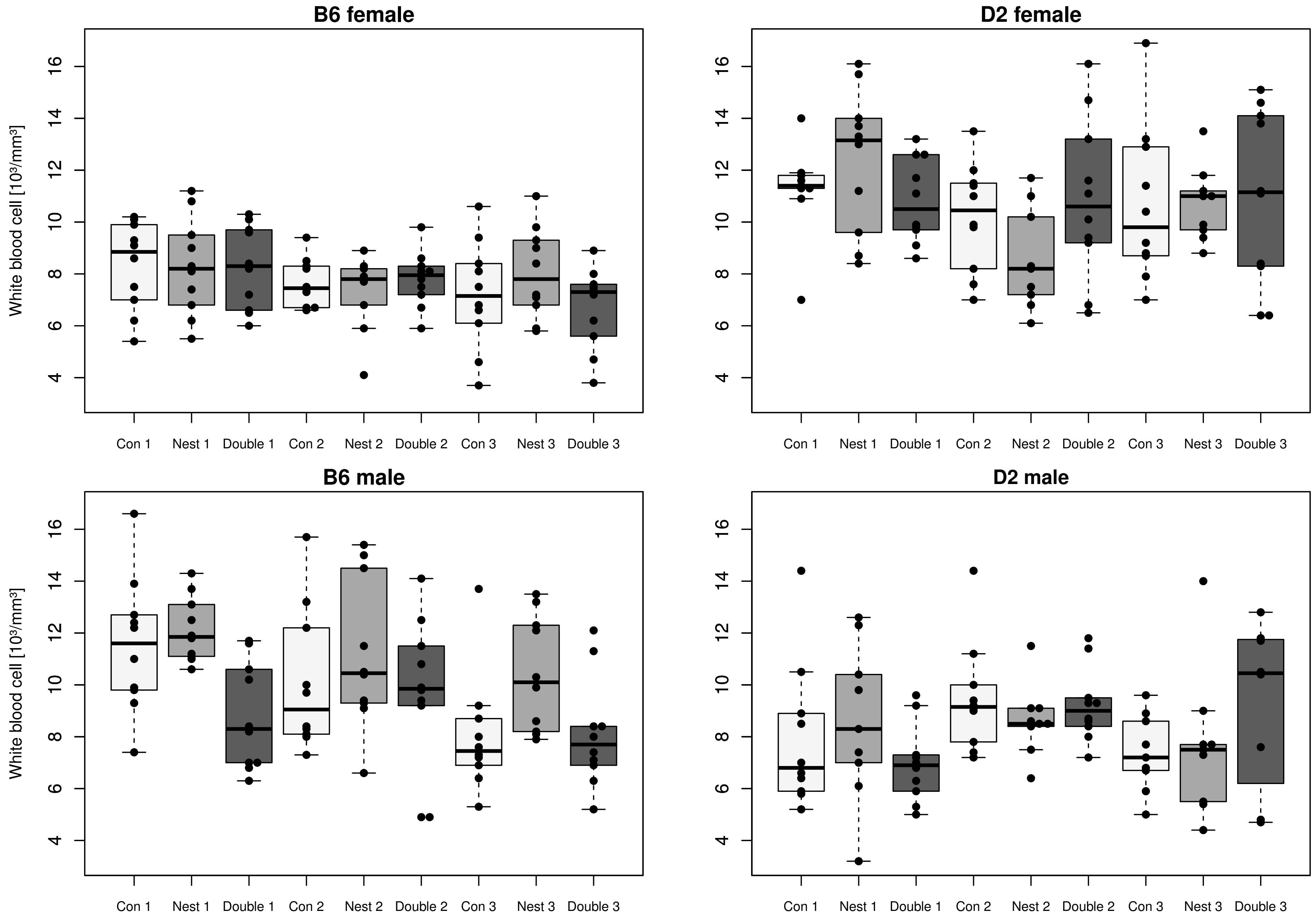

B6 female
D2 female
B6 male
D2 male
White blood cell [10³/mm³]
4
6
8
10
12
14
16
Con 1
Nest 1
Double 1
Con 2
Nest 2
Double 2
Con 3
Nest 3
Double 3

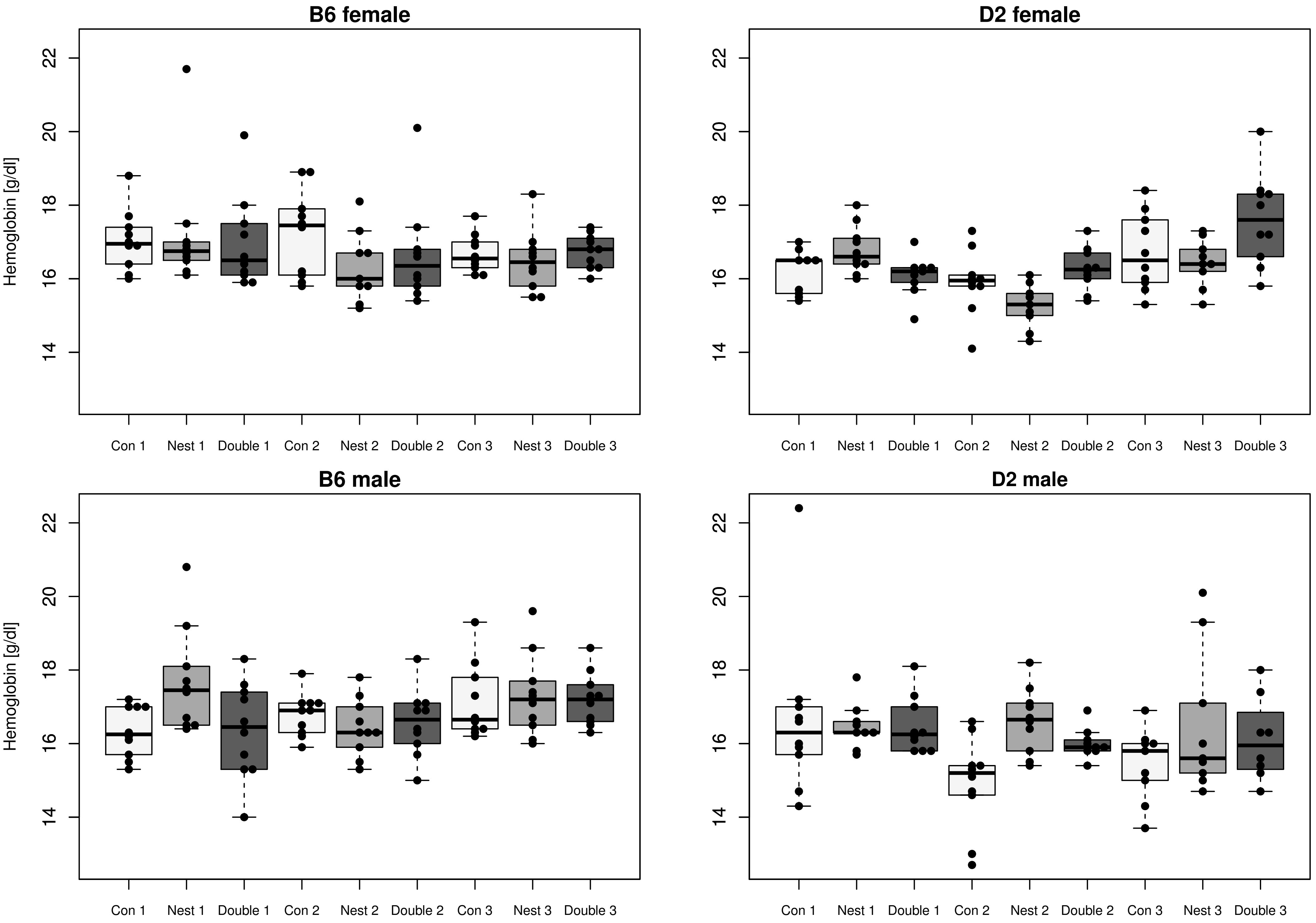

B6 female
D2 female
B6 male
D2 male
Hemoglobin [g/dl]
14
16
18
20
22
Con 1
Nest 1
Double 1
Con 2
Nest 2
Double 2
Con 3
Nest 3
Double 3

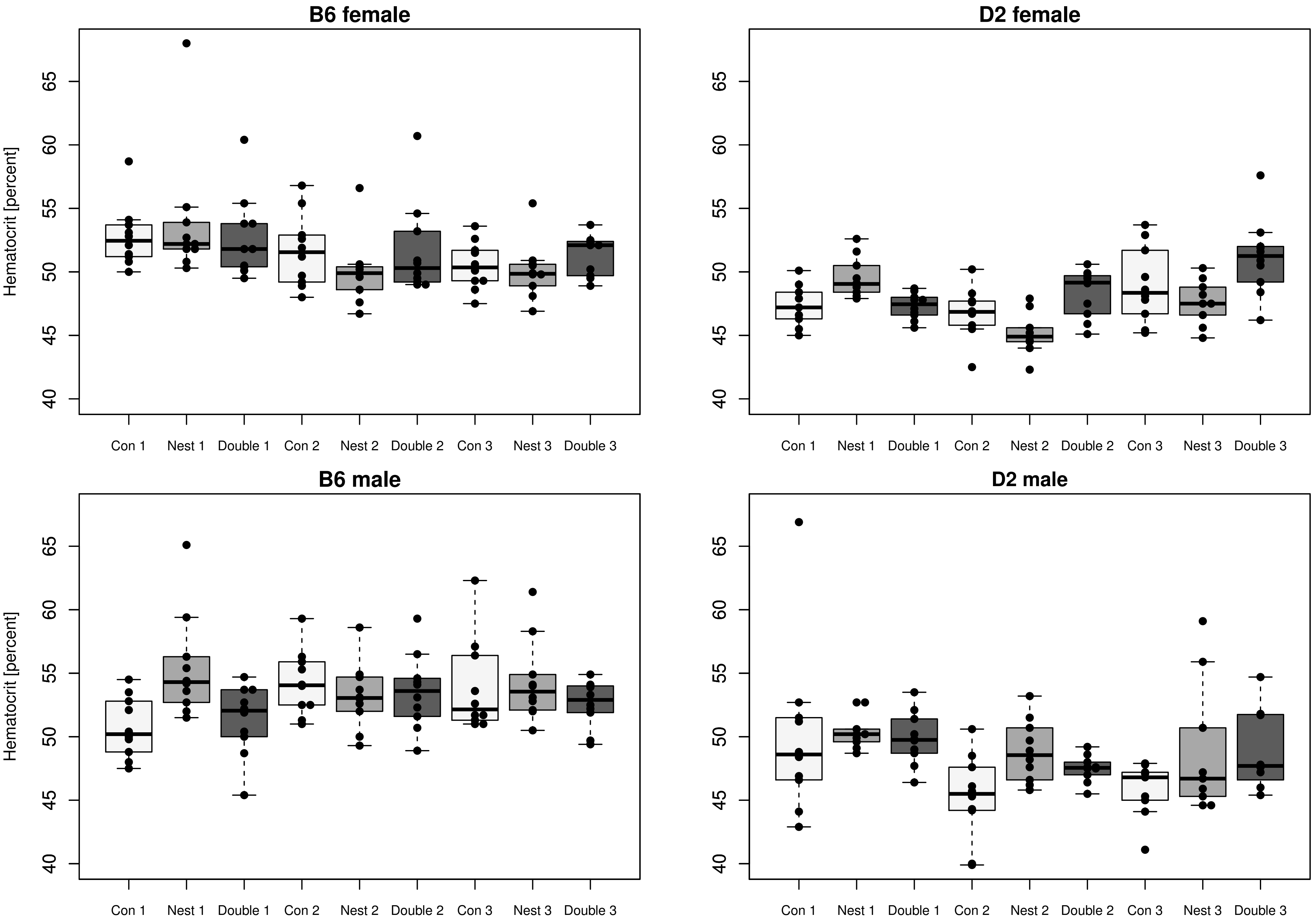
B6 female
D2 female
B6 male
D2 male
Hematocrit [percent]
40
45
50
55
60
65
Con 1
Nest 1
Double 1
Con 2
Nest 2
Double 2
Con 3
Nest 3
Double 3

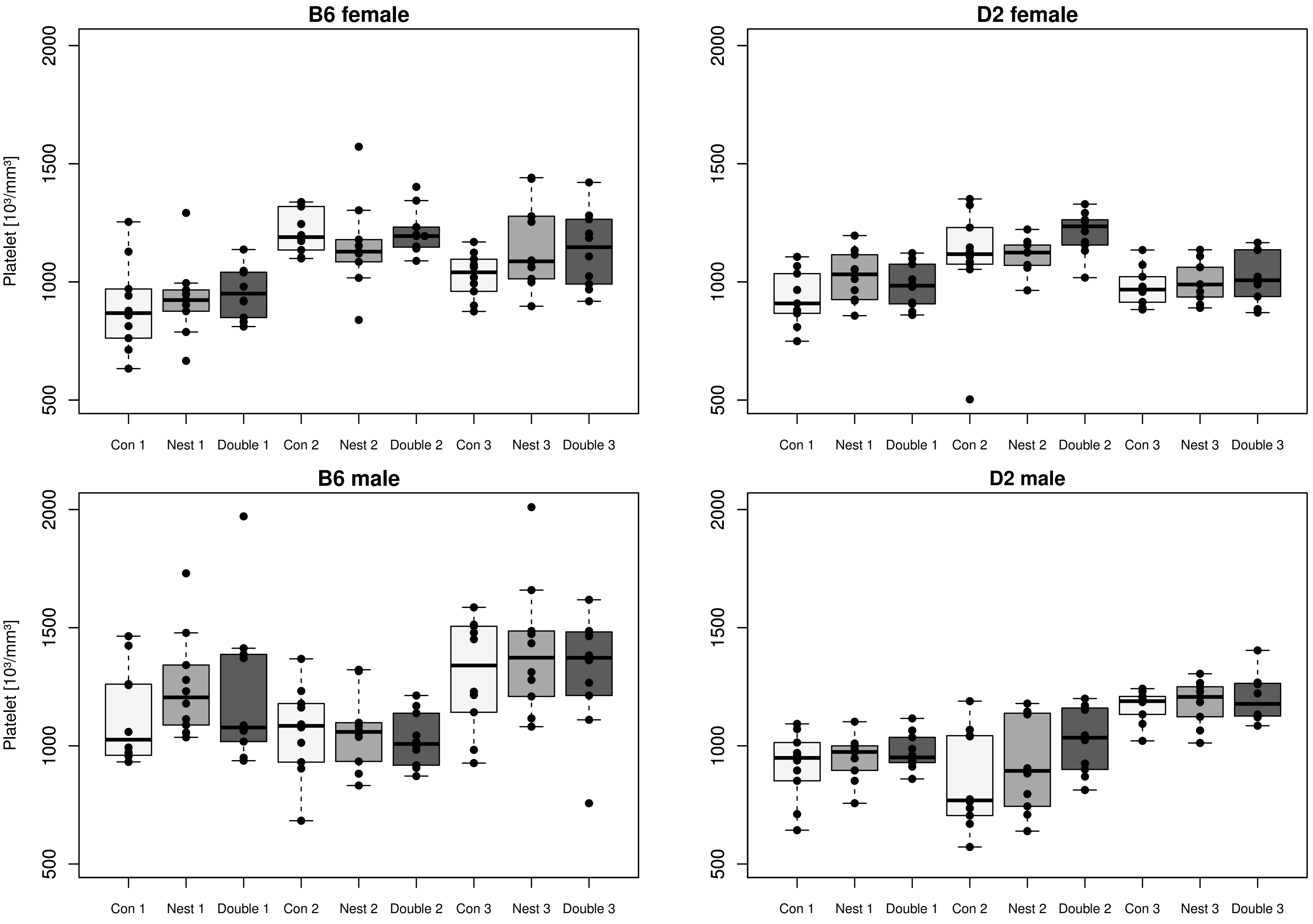

B6 female
D2 female
B6 male
D2 male
Platelet [10³/mm³]
500
1000
1500
2000
Con 1
Nest 1
Double 1
Con 2
Nest 2
Double 2
Con 3
Nest 3
Double 3

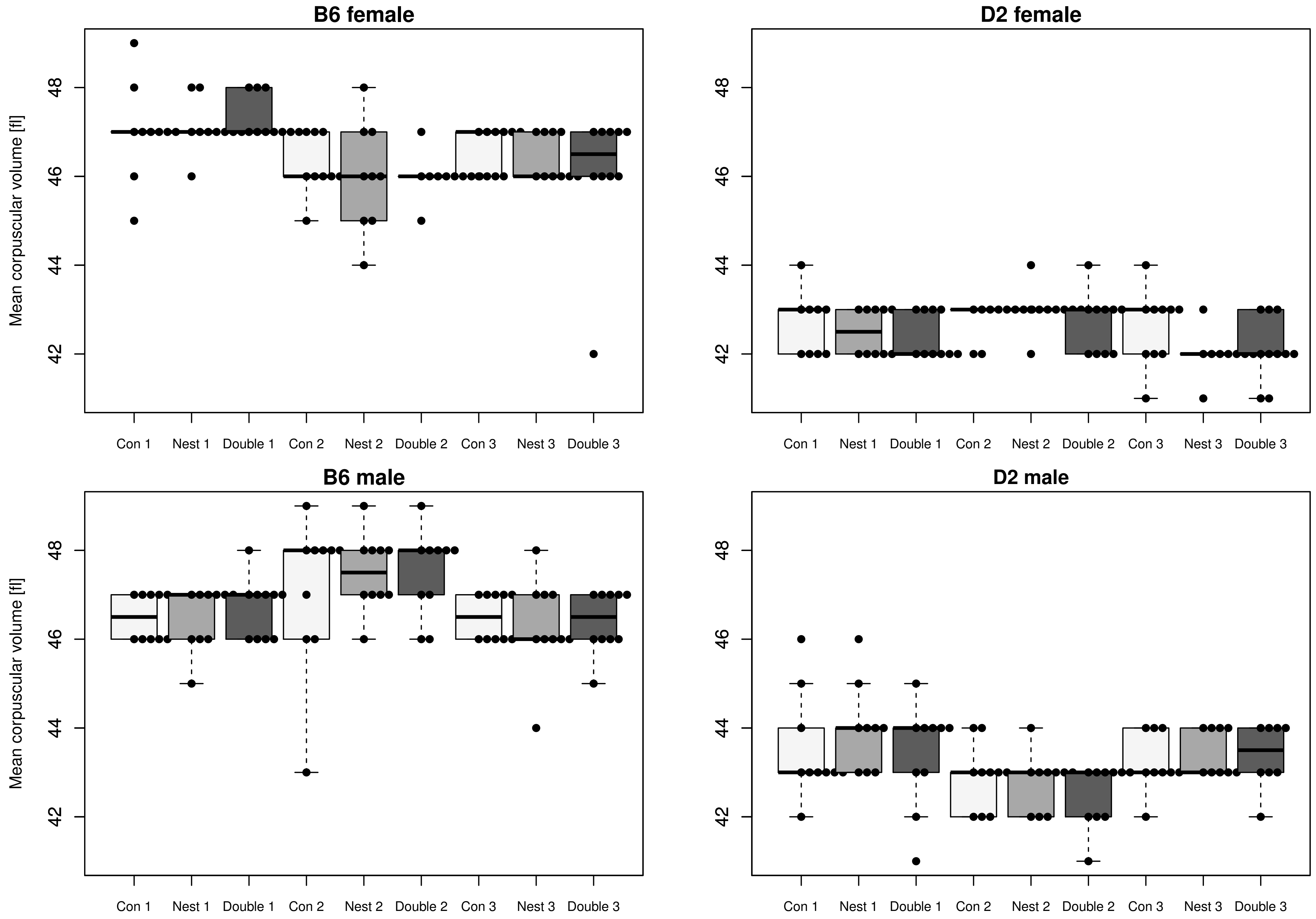
B6 female
D2 female
B6 male
D2 male
Mean corpuscular volume [fl]
42
44
46
48
Con 1
Nest 1
Double 1
Con 2
Nest 2
Double 2
Con 3
Nest 3
Double 3

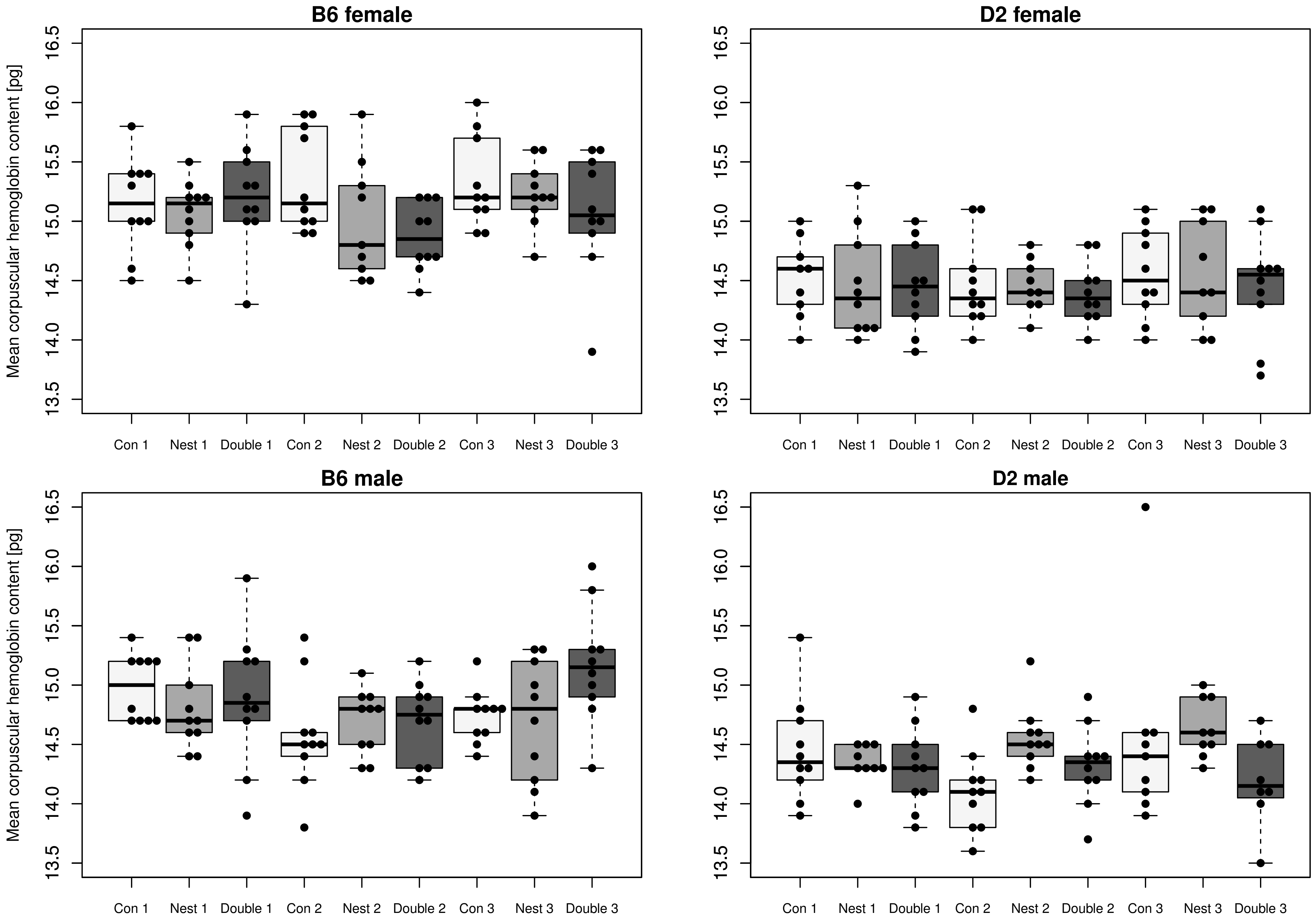

B6 female
D2 female
B6 male
D2 male
Mean corpuscular hemoglobin content [pg]
13.5
14.0
14.5
15.0
15.5
16.0
16.5
Con 1
Nest 1
Double 1
Con 2
Nest 2
Double 2
Con 3
Nest 3
Double 3

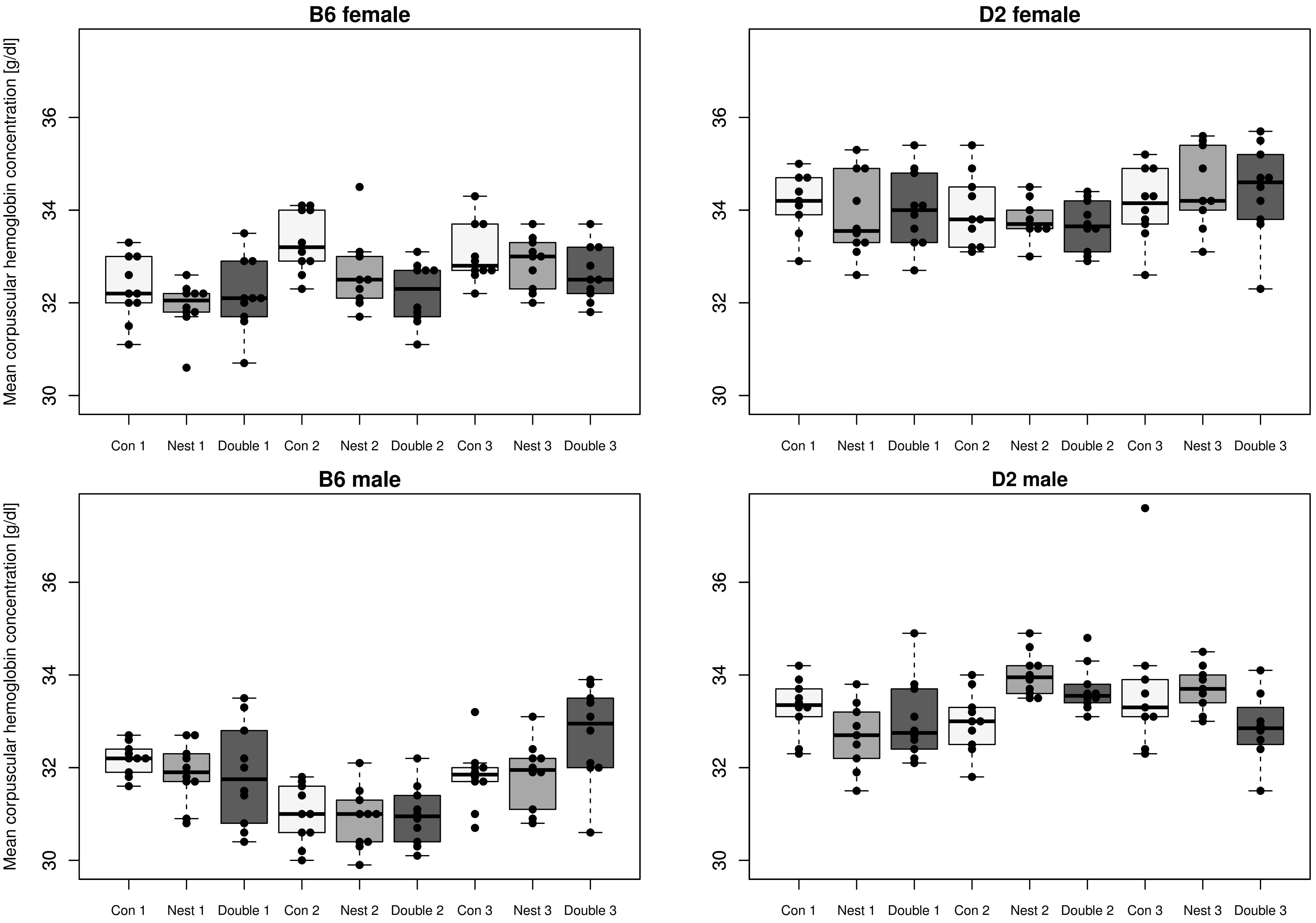
B6 female
D2 female
B6 male
D2 male
Mean corpuscular hemoglobin concentration [g/dl]
30
32
34
36
Con 1
Nest 1
Double 1
Con 2
Nest 2
Double 2
Con 3
Nest 3
Double 3

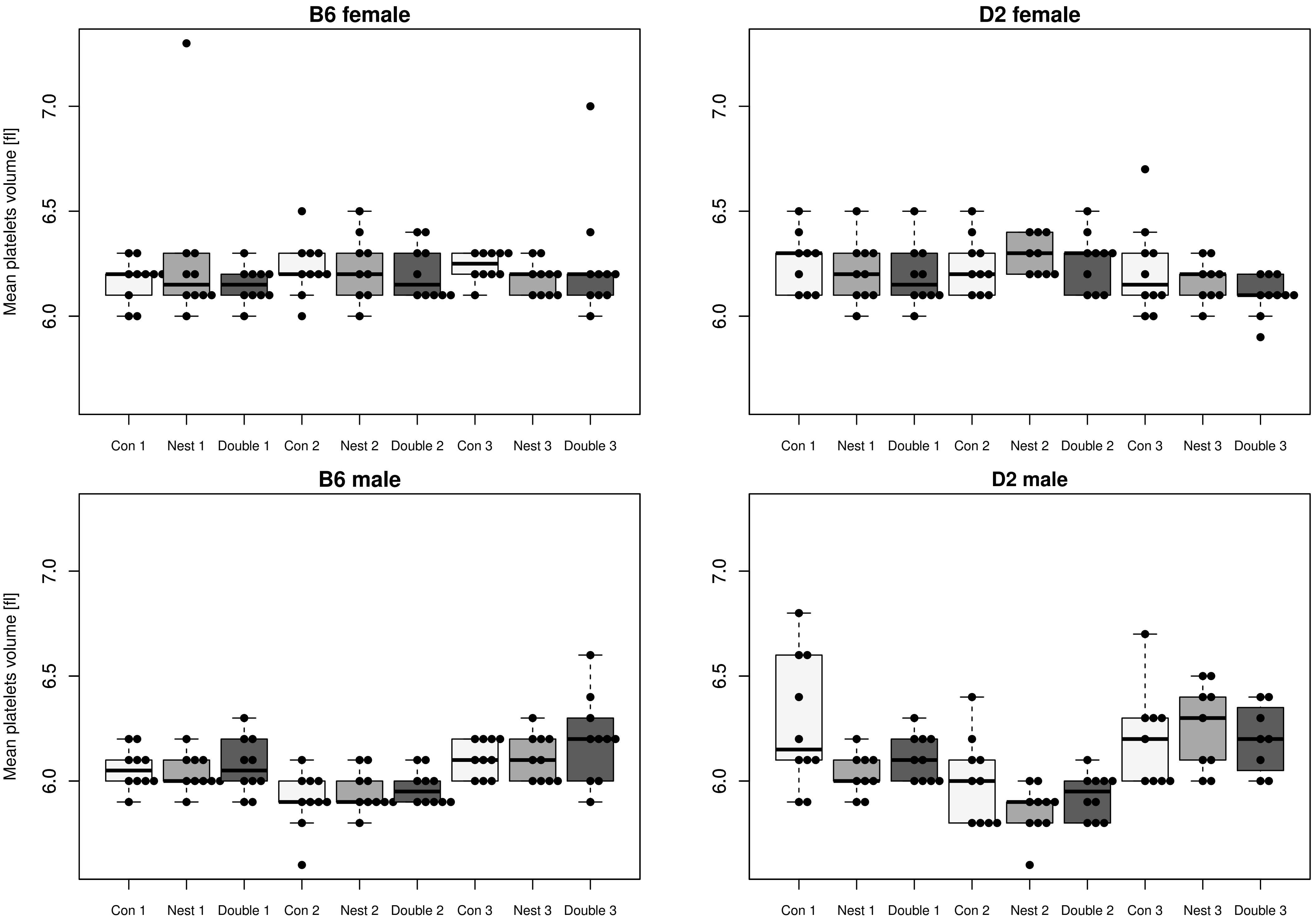
B6 female
D2 female
B6 male
D2 male
Mean platelets volume [fl]
7.0
6.5
6.0
Con 1
Nest 1
Double 1
Con 2
Nest 2
Double 2
Con 3
Nest 3
Double 3

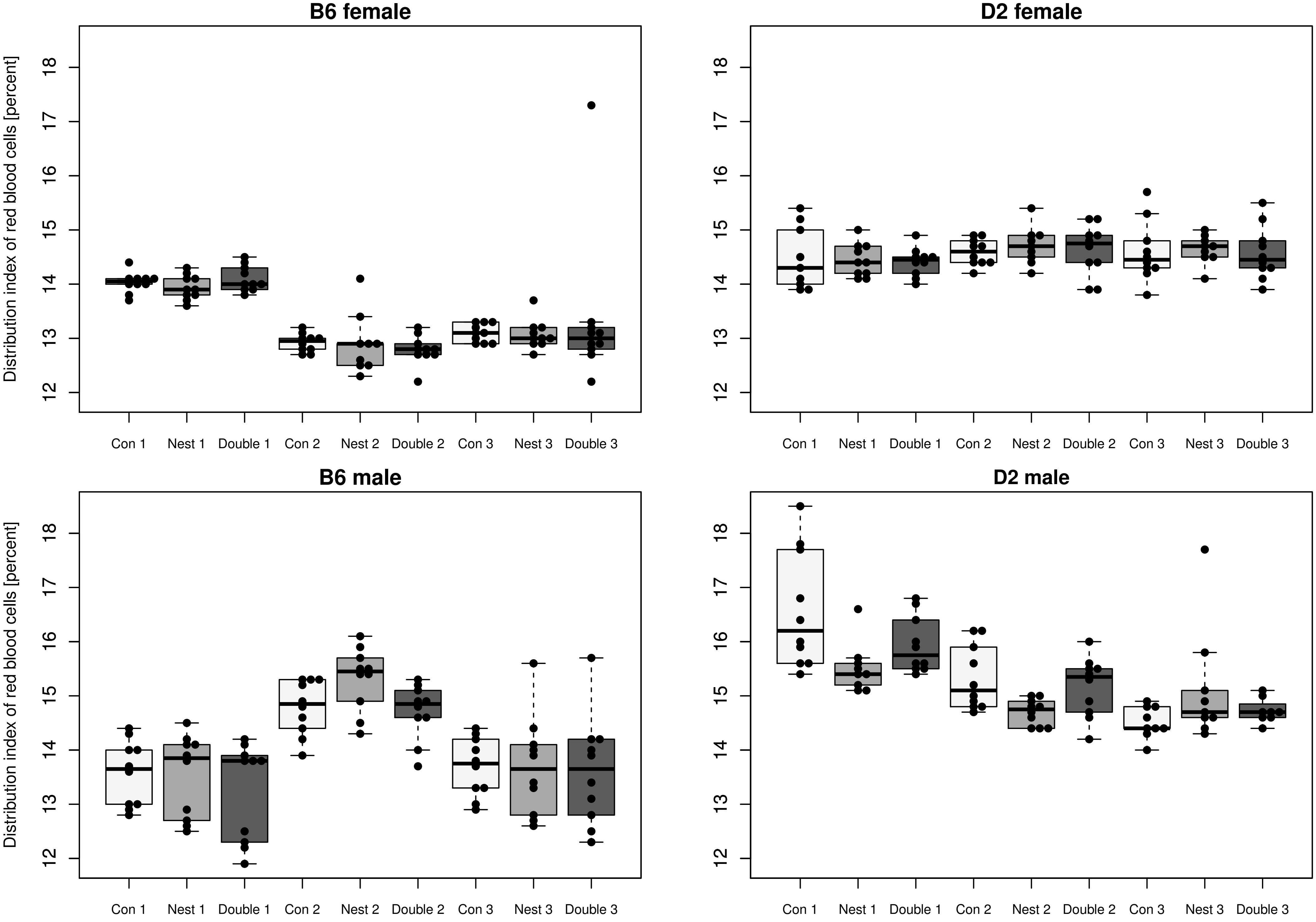
B6 female
D2 female
B6 male
D2 male
Distribution index of red blood cells [percent]
12
13
14
15
16
17
18
Con 1
Nest 1
Double 1
Con 2
Nest 2
Double 2
Con 3
Nest 3
Double 3

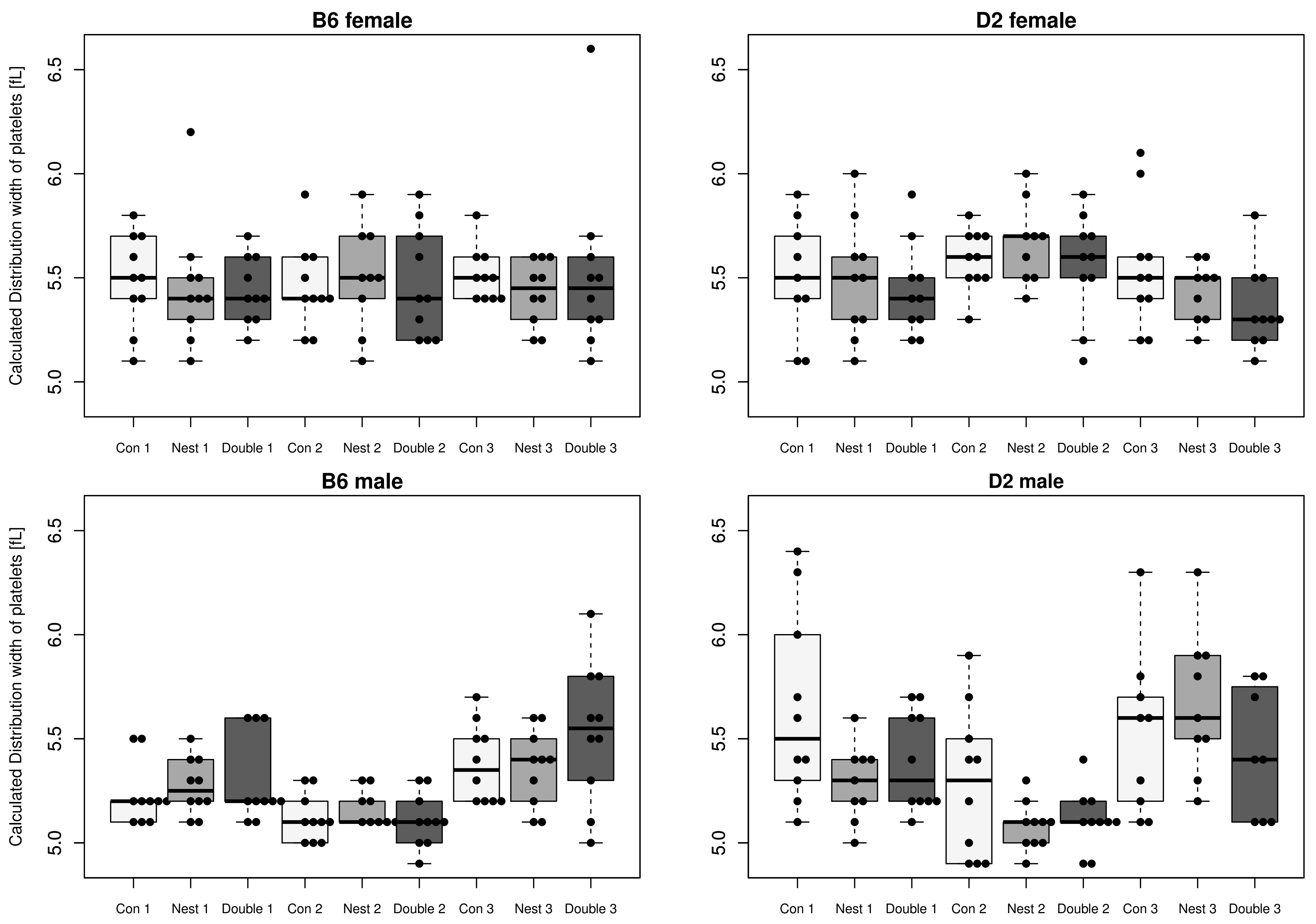

B6 female
D2 female
B6 male
D2 male
Calculated Distribution width of platelets [fL]
5.0
5.5
6.0
6.5
Con 1
Nest 1
Double 1
Con 2
Nest 2
Double 2
Con 3
Nest 3
Double 3

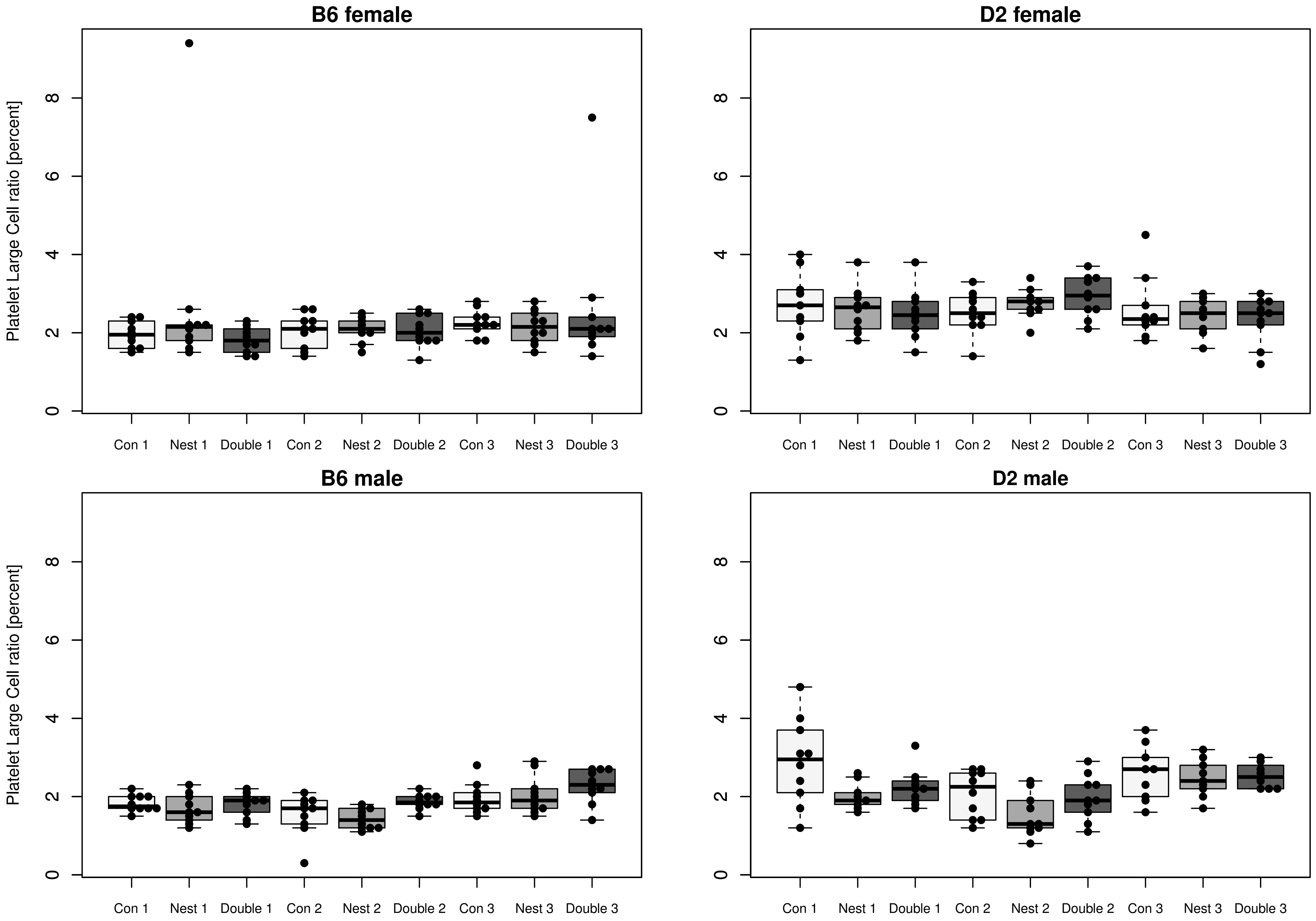
B6 female
D2 female
B6 male
D2 male
Platelet Large Cell ratio [percent]
0
2
4
6
8
Con 1
Nest 1
Double 1
Con 2
Nest 2
Double 2
Con 3
Nest 3
Double 3

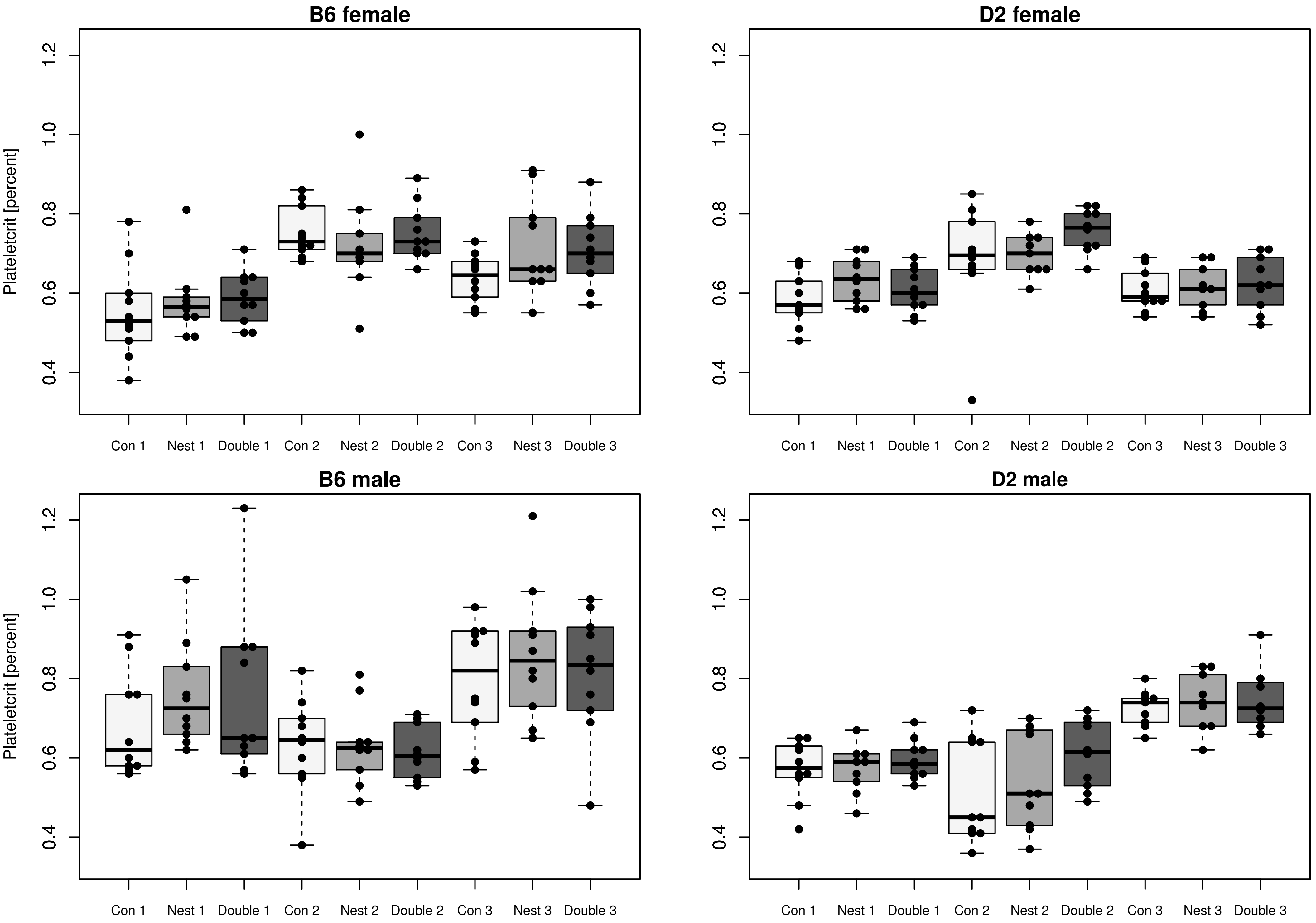

B6 female
D2 female
B6 male
D2 male
Plateletcrit [percent]
0.4
0.6
0.8
1.0
1.2
Con 1
Nest 1
Double 1
Con 2
Nest 2
Double 2
Con 3
Nest 3
Double 3

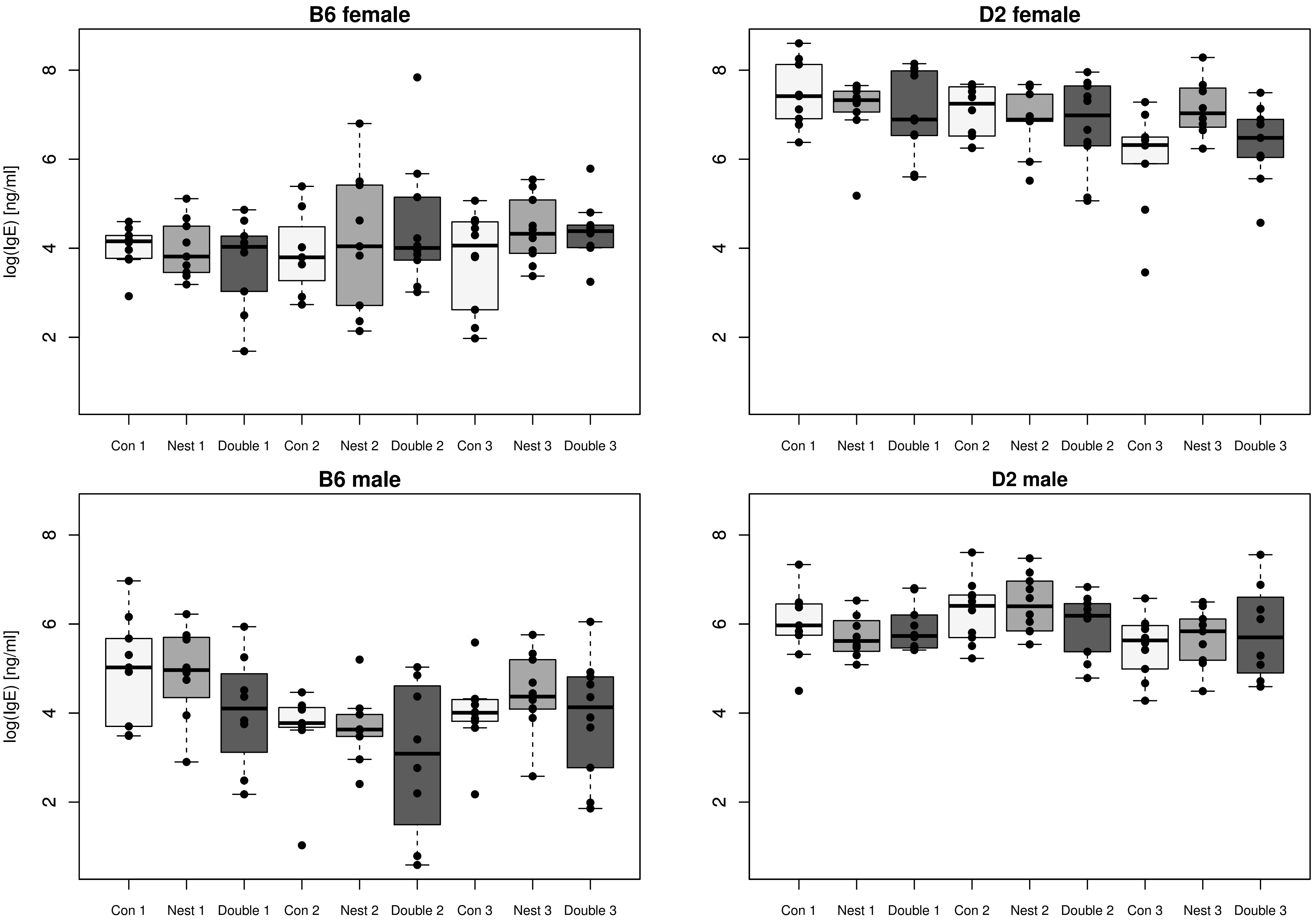
B6 female
D2 female
B6 male
D2 male
log(IgE) [ng/ml]
2
4
6
8
Con 1
Nest 1
Double 1
Con 2
Nest 2
Double 2
Con 3
Nest 3
Double 3

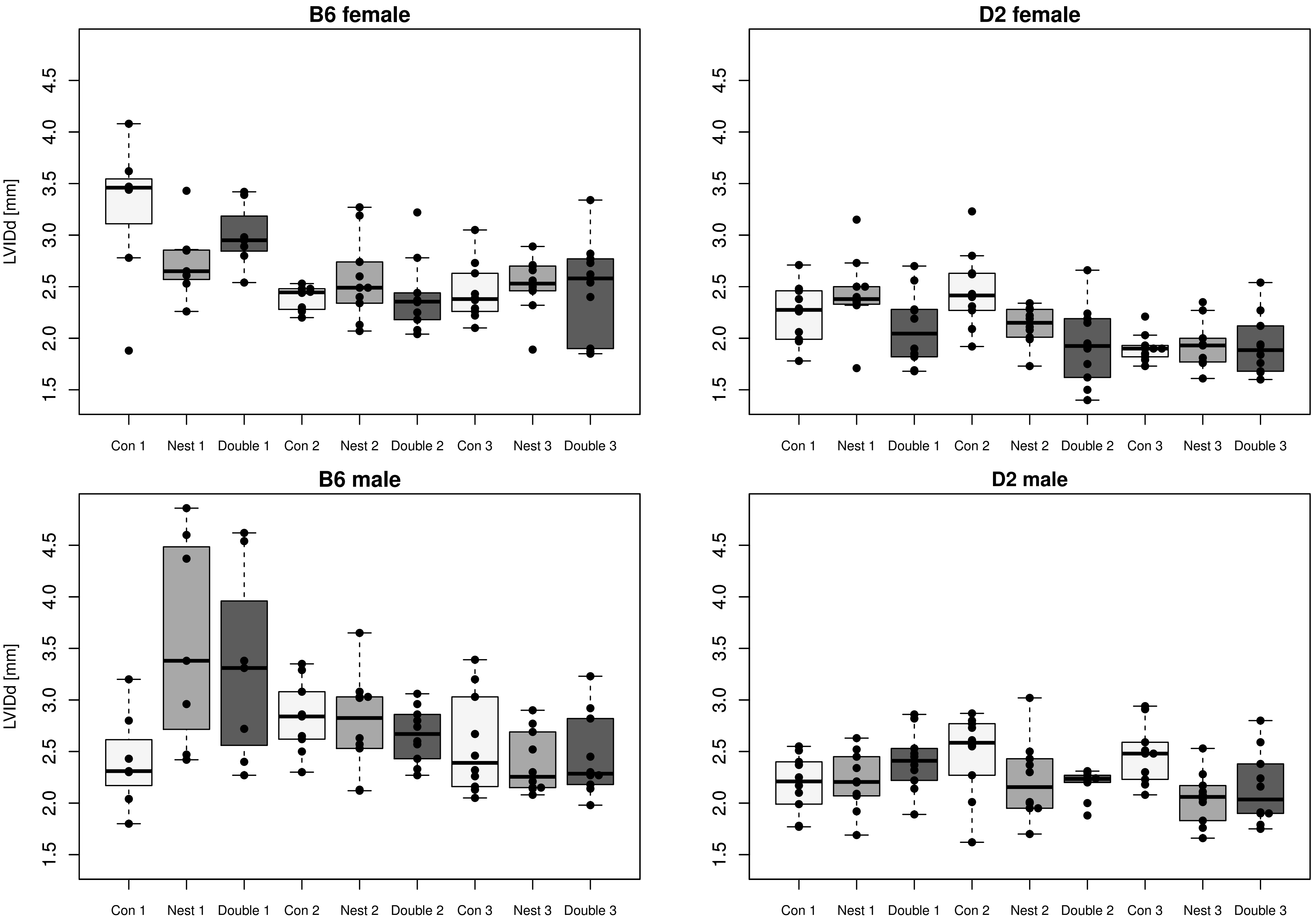

B6 female
D2 female
B6 male
D2 male
LVIDd [mm]
1.5
2.0
2.5
3.0
3.5
4.0
4.5
Con 1
Nest 1
Double 1
Con 2
Nest 2
Double 2
Con 3
Nest 3
Double 3

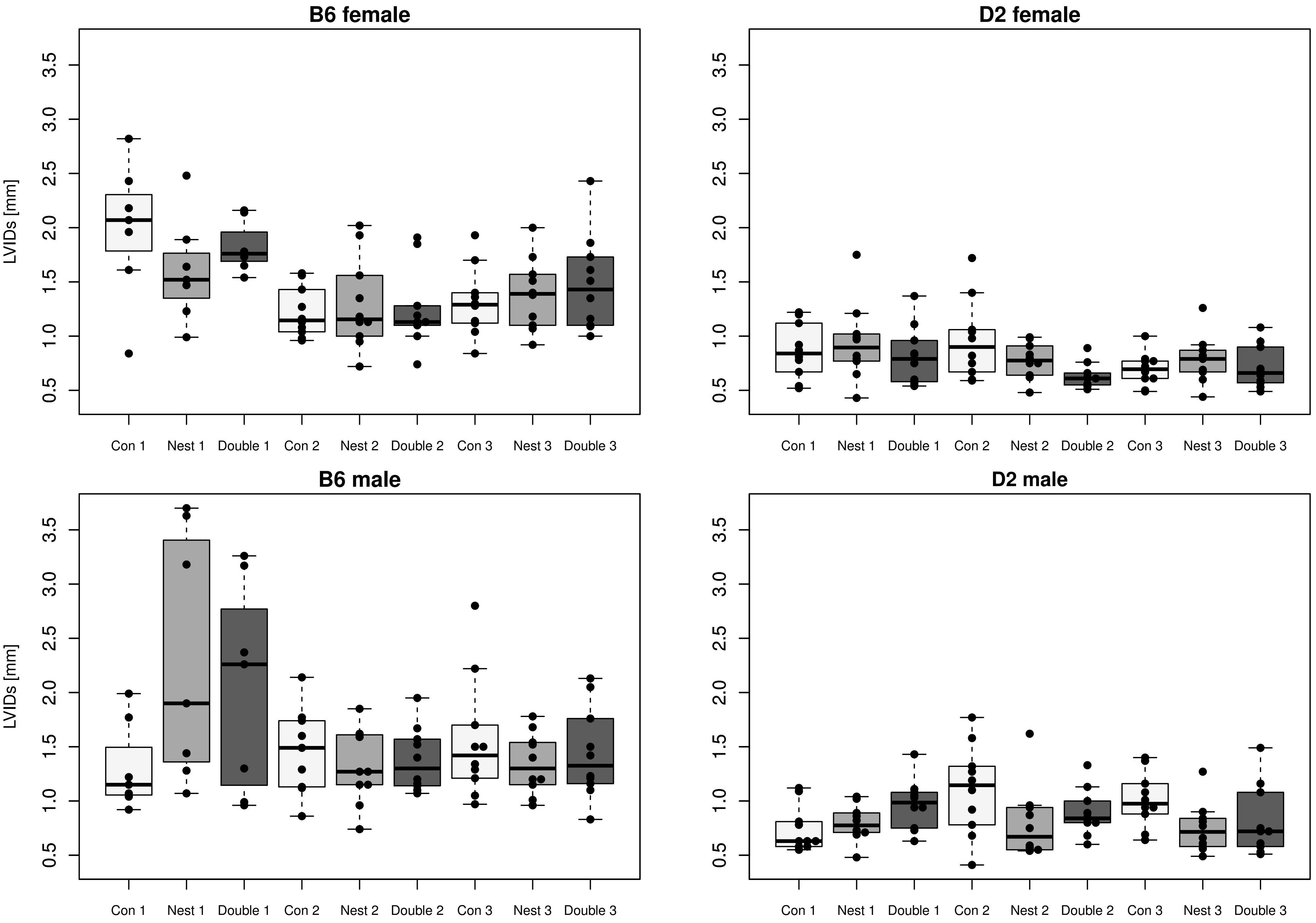

B6 female
D2 female
B6 male
D2 male
LVIDs [mm]
0.5
1.0
1.5
2.0
2.5
3.0
3.5
Con 1
Nest 1
Double 1
Con 2
Nest 2
Double 2
Con 3
Nest 3
Double 3

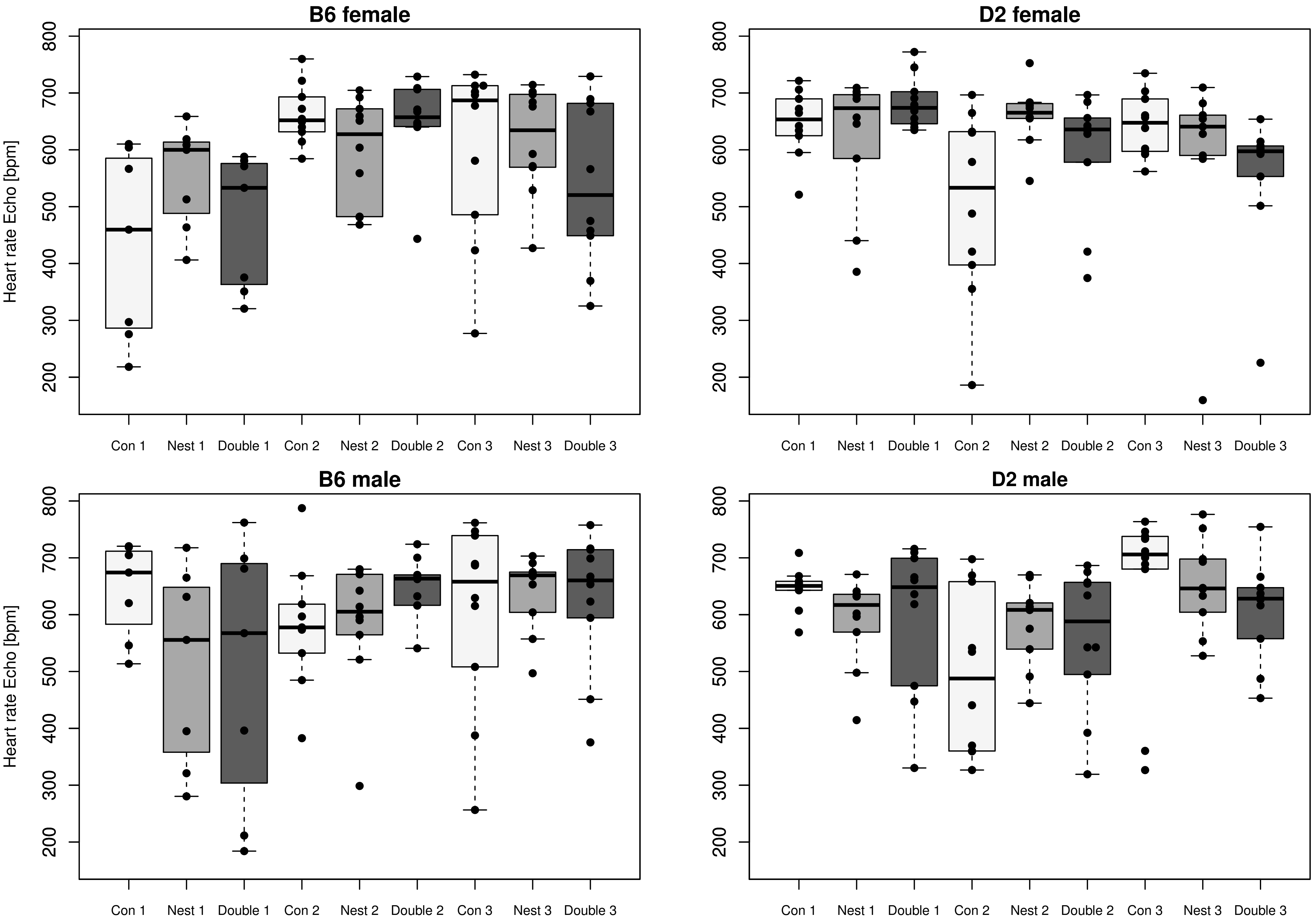
B6 female
D2 female
B6 male
D2 male
Heart rate Echo [bpm]
200
300
400
500
600
700
800
Con 1
Nest 1
Double 1
Con 2
Nest 2
Double 2
Con 3
Nest 3
Double 3

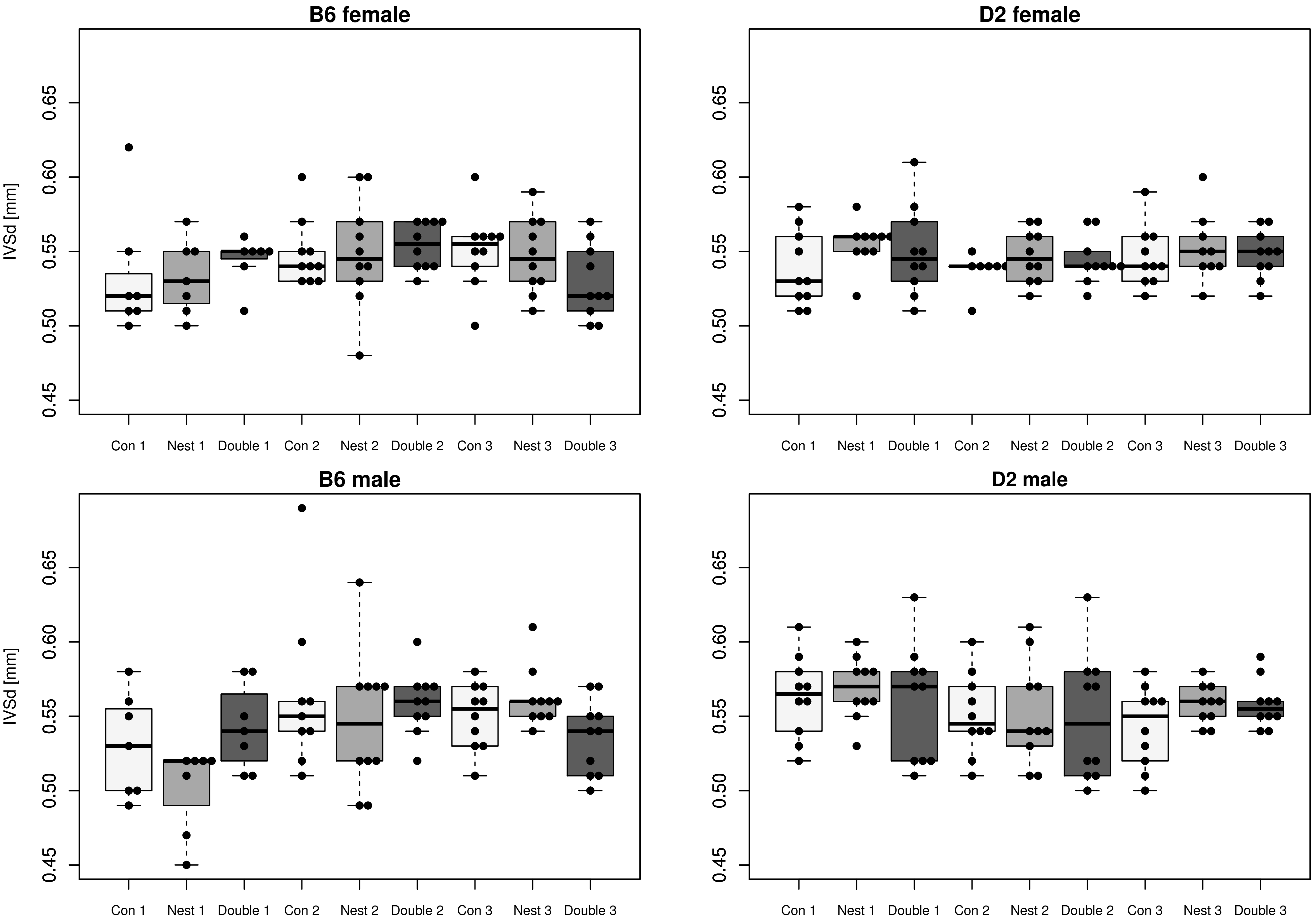
B6 female
D2 female
B6 male
D2 male
IVSd [mm]
0.45
0.50
0.55
0.60
0.65
Con 1
Nest 1
Double 1
Con 2
Nest 2
Double 2
Con 3
Nest 3
Double 3

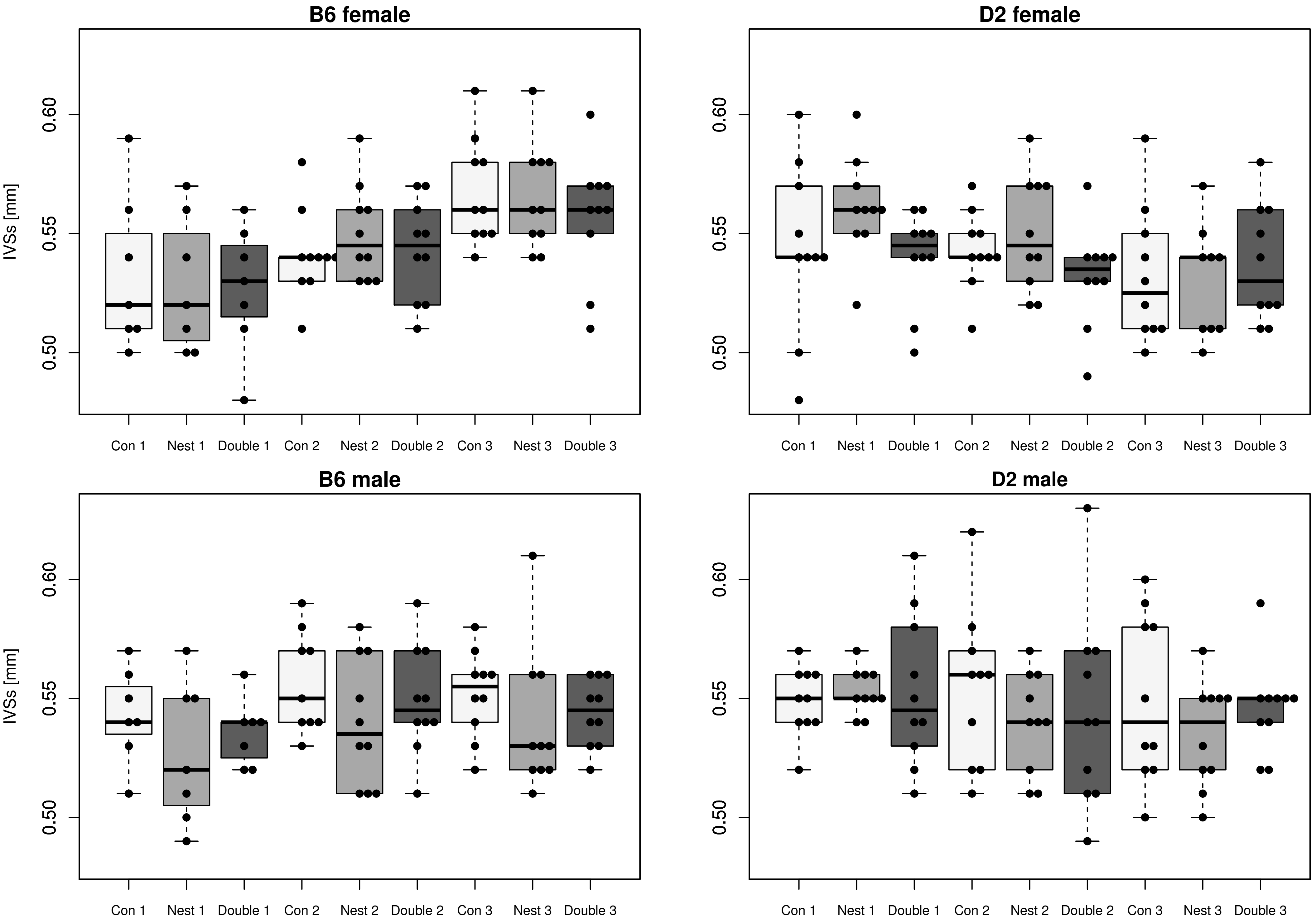

B6 female
D2 female
B6 male
D2 male
IVSs [mm]
0.50
0.55
0.60
Con 1
Nest 1
Double 1
Con 2
Nest 2
Double 2
Con 3
Nest 3
Double 3

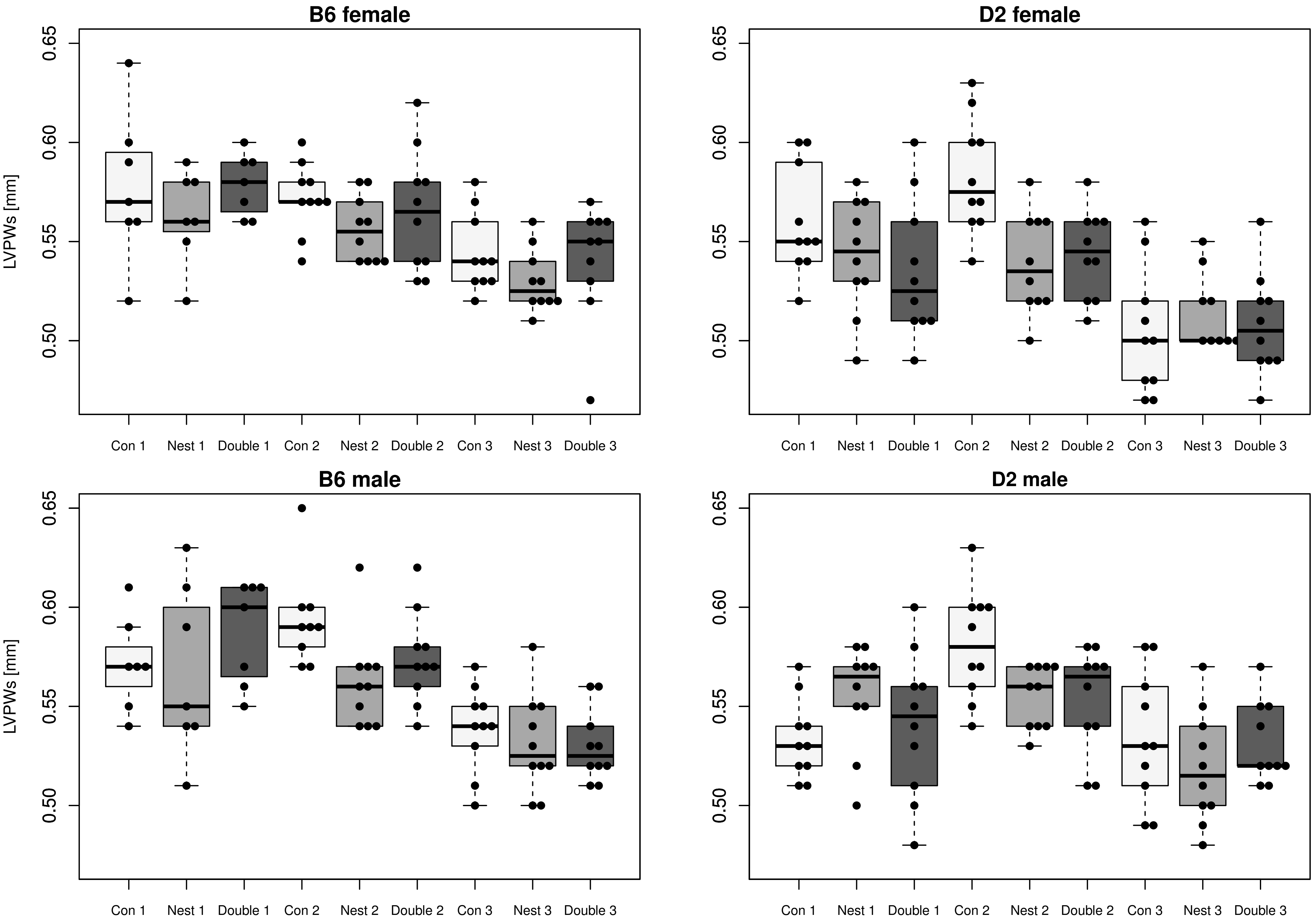

B6 female
D2 female
B6 male
D2 male
LVPWs [mm]
0.50
0.55
0.60
0.65
Con 1
Nest 1
Double 1
Con 2
Nest 2
Double 2
Con 3
Nest 3
Double 3

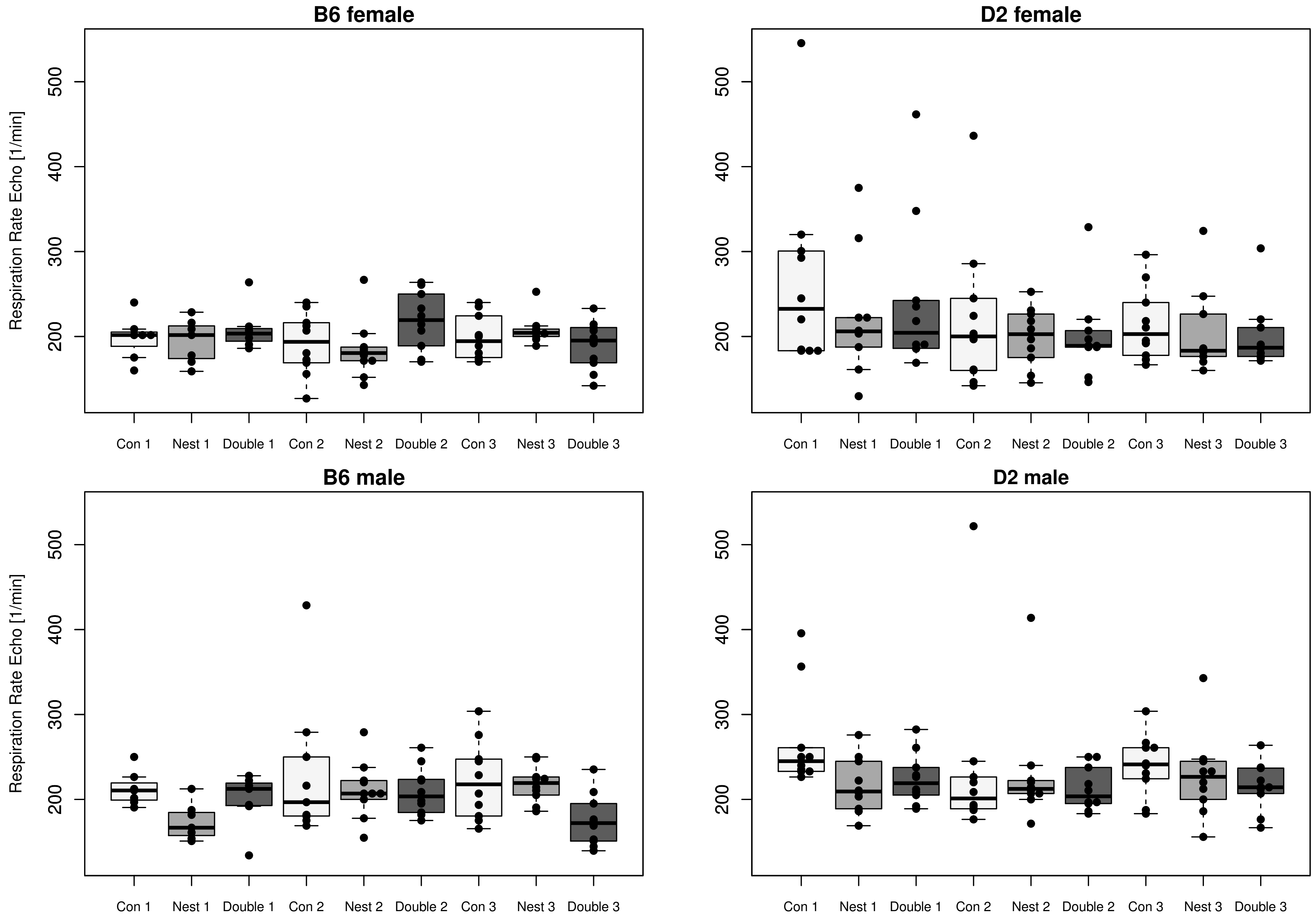
B6 female
D2 female
B6 male
D2 male
Respiration Rate Echo [1/min]
500
400
300
200
Con 1
Nest 1
Double 1
Con 2
Nest 2
Double 2
Con 3
Nest 3
Double 3

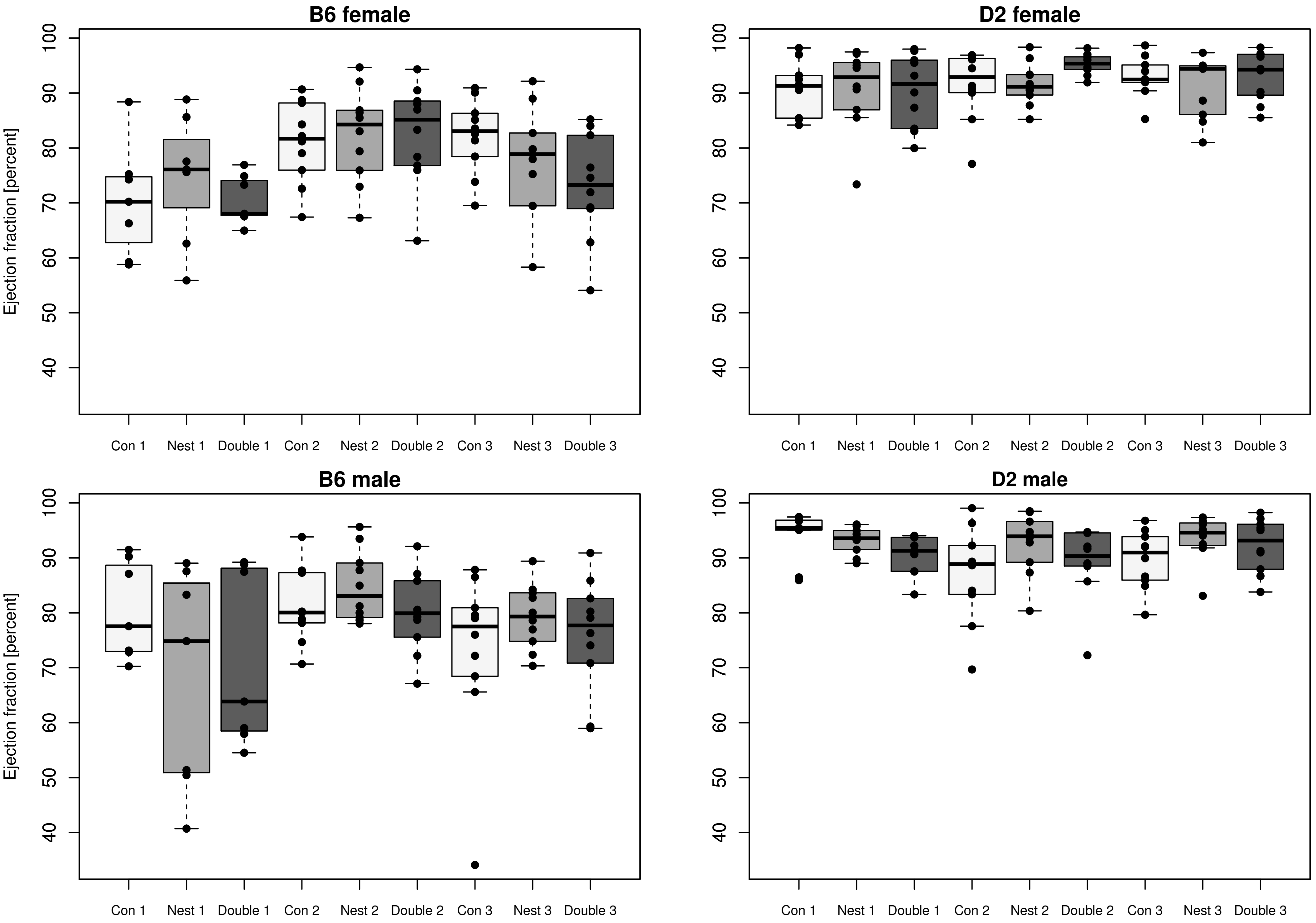
B6 female
D2 female
B6 male
D2 male
Ejection fraction [percent]
100
90
80
70
60
50
40
Con 1
Nest 1
Double 1
Con 2
Nest 2
Double 2
Con 3
Nest 3
Double 3

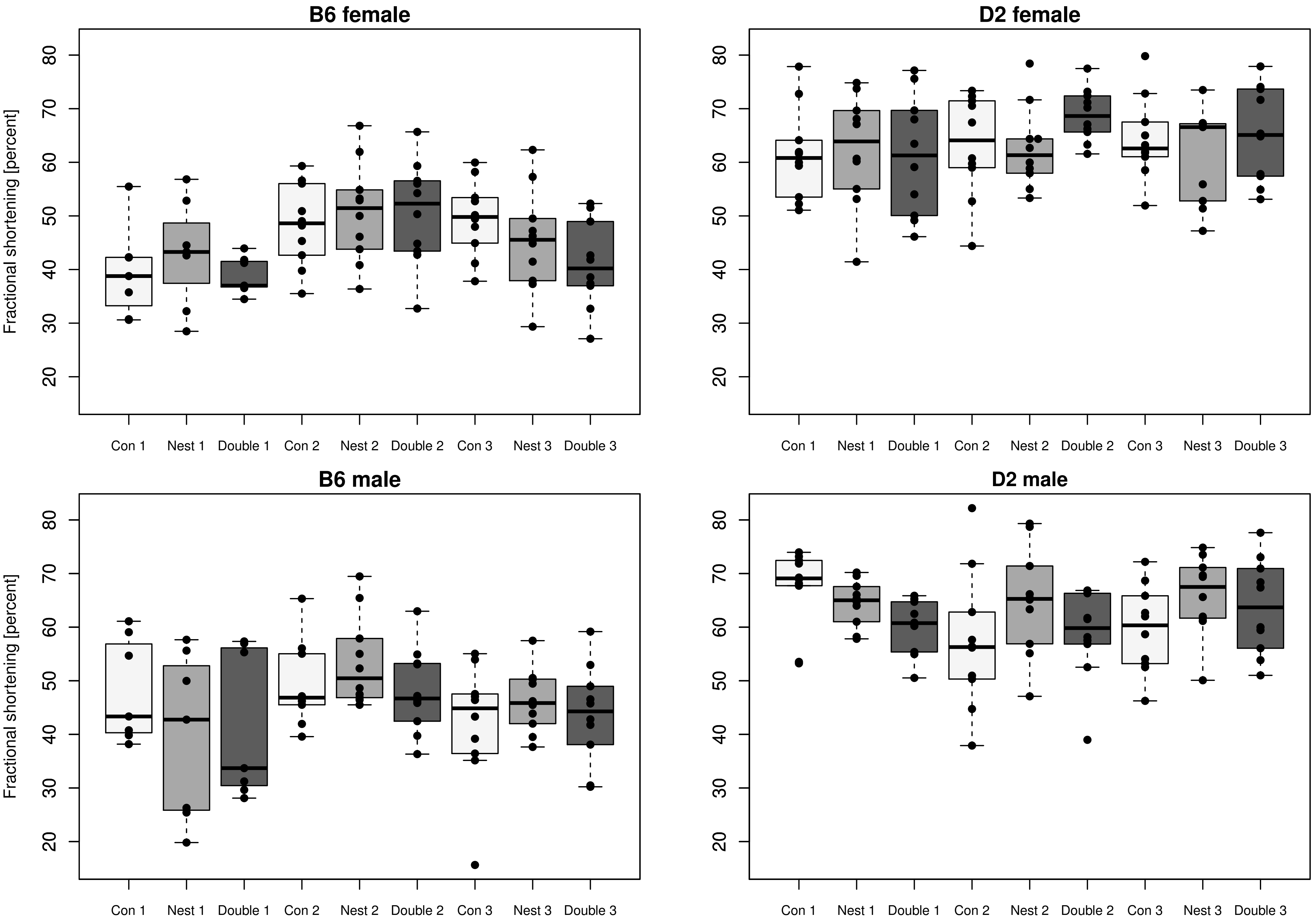

B6 female
D2 female
B6 male
D2 male
Fractional shortening [percent]
20
30
40
50
60
70
80
Con 1
Nest 1
Double 1
Con 2
Nest 2
Double 2
Con 3
Nest 3
Double 3

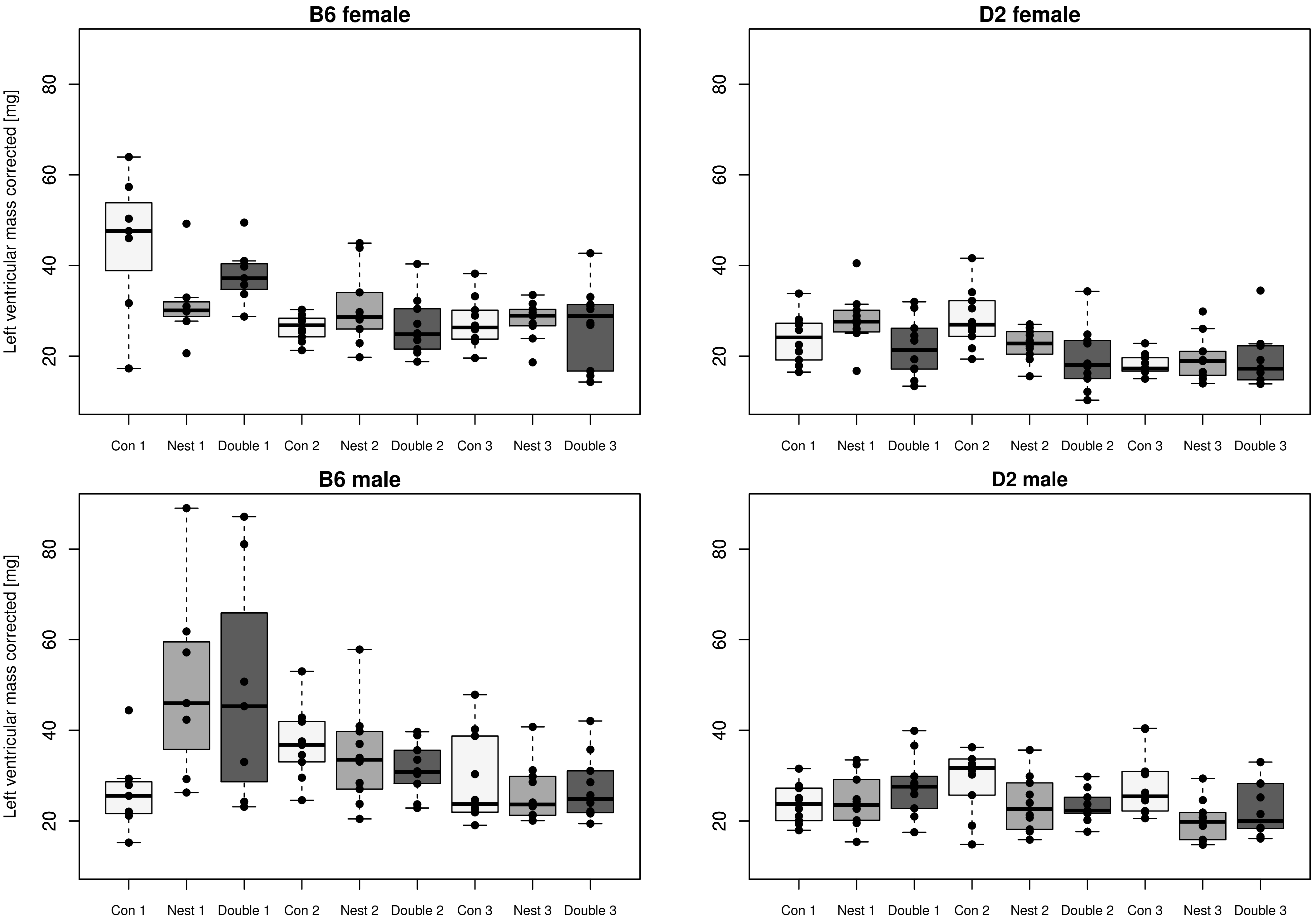

B6 female
D2 female
B6 male
D2 male
Left ventricular mass corrected [mg]
20
40
60
80
Con 1
Nest 1
Double 1
Con 2
Nest 2
Double 2
Con 3
Nest 3
Double 3

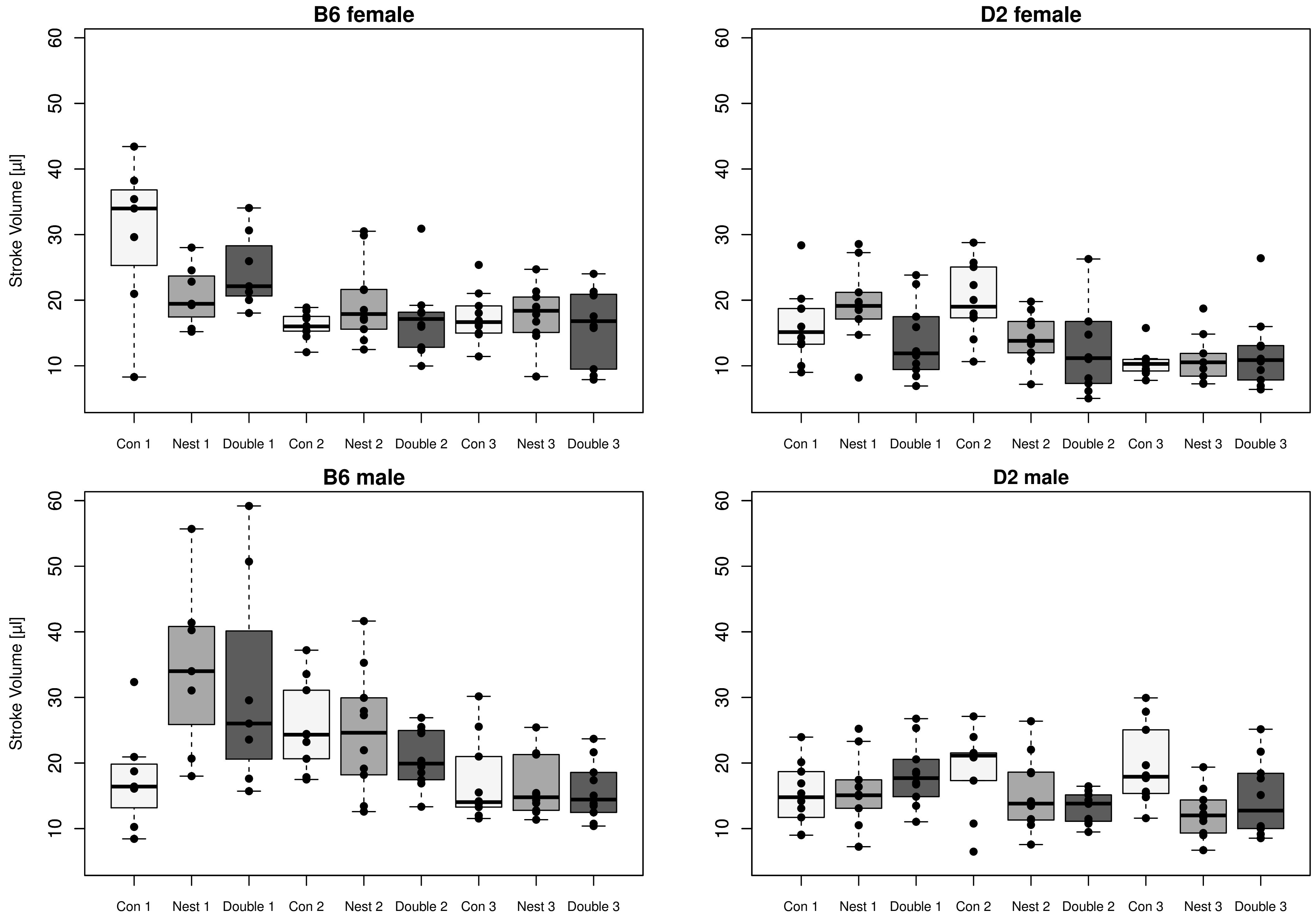
B6 female
D2 female
B6 male
D2 male
Stroke Volume [µl]
10
20
30
40
50
60
Con 1
Nest 1
Double 1
Con 2
Nest 2
Double 2
Con 3
Nest 3
Double 3

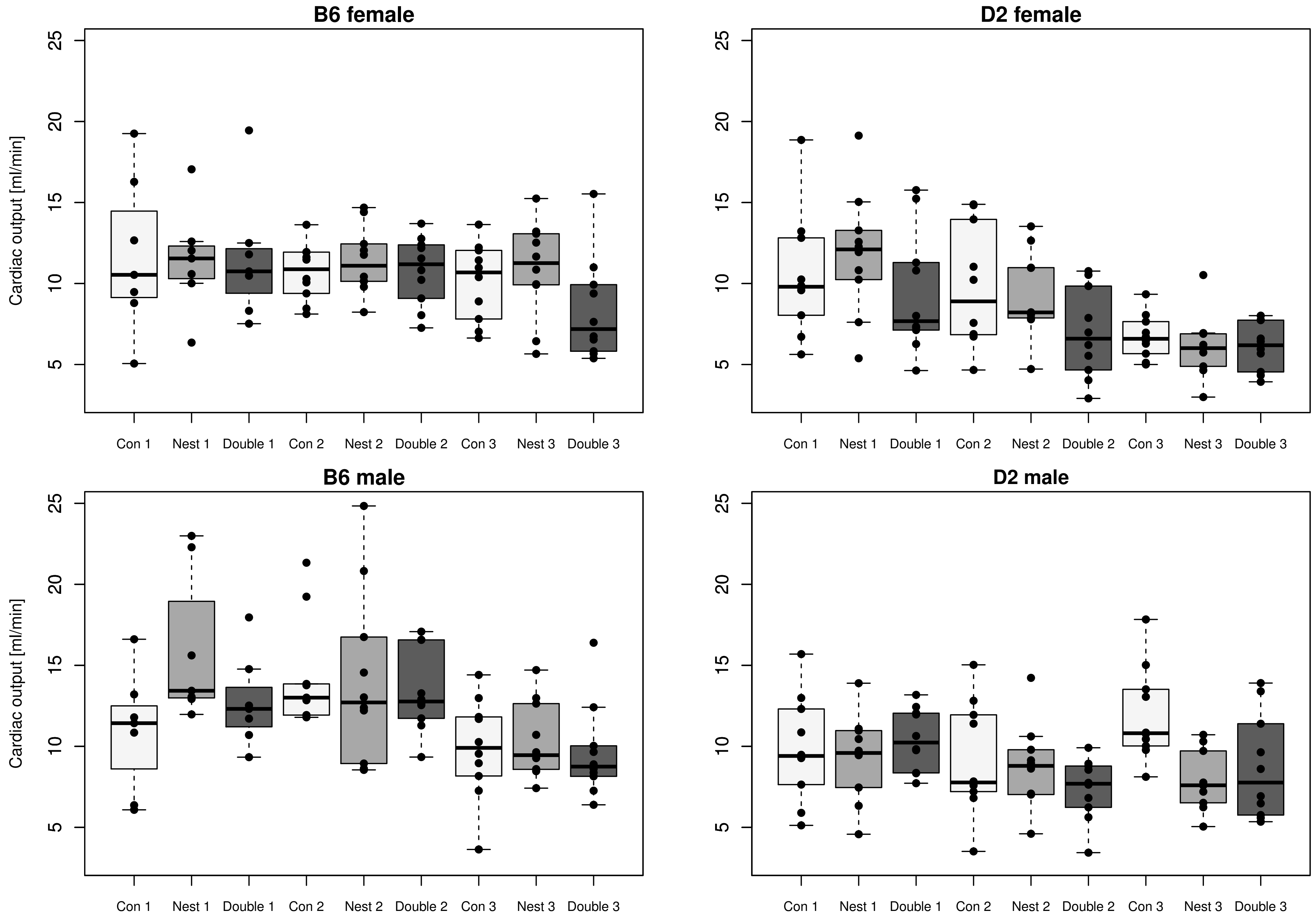

B6 female
D2 female
B6 male
D2 male
Cardiac output [ml/min]
Con 1
Nest 1
Double 1
Con 2
Nest 2
Double 2
Con 3
Nest 3
Double 3
5
10
15
20
25

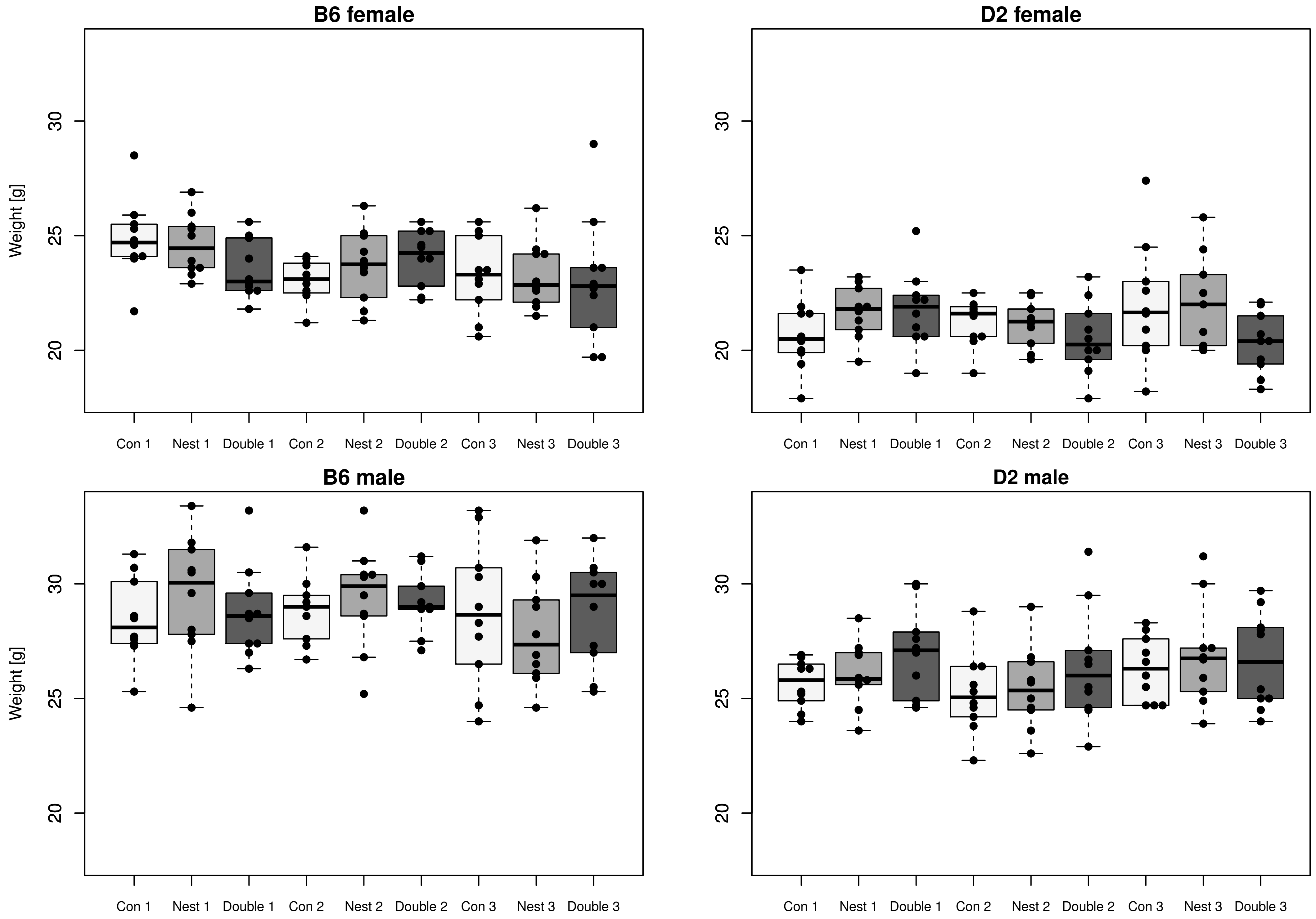

B6 female
D2 female
B6 male
D2 male
Weight [g]
20
25
30
Con 1
Nest 1
Double 1
Con 2
Nest 2
Double 2
Con 3
Nest 3
Double 3

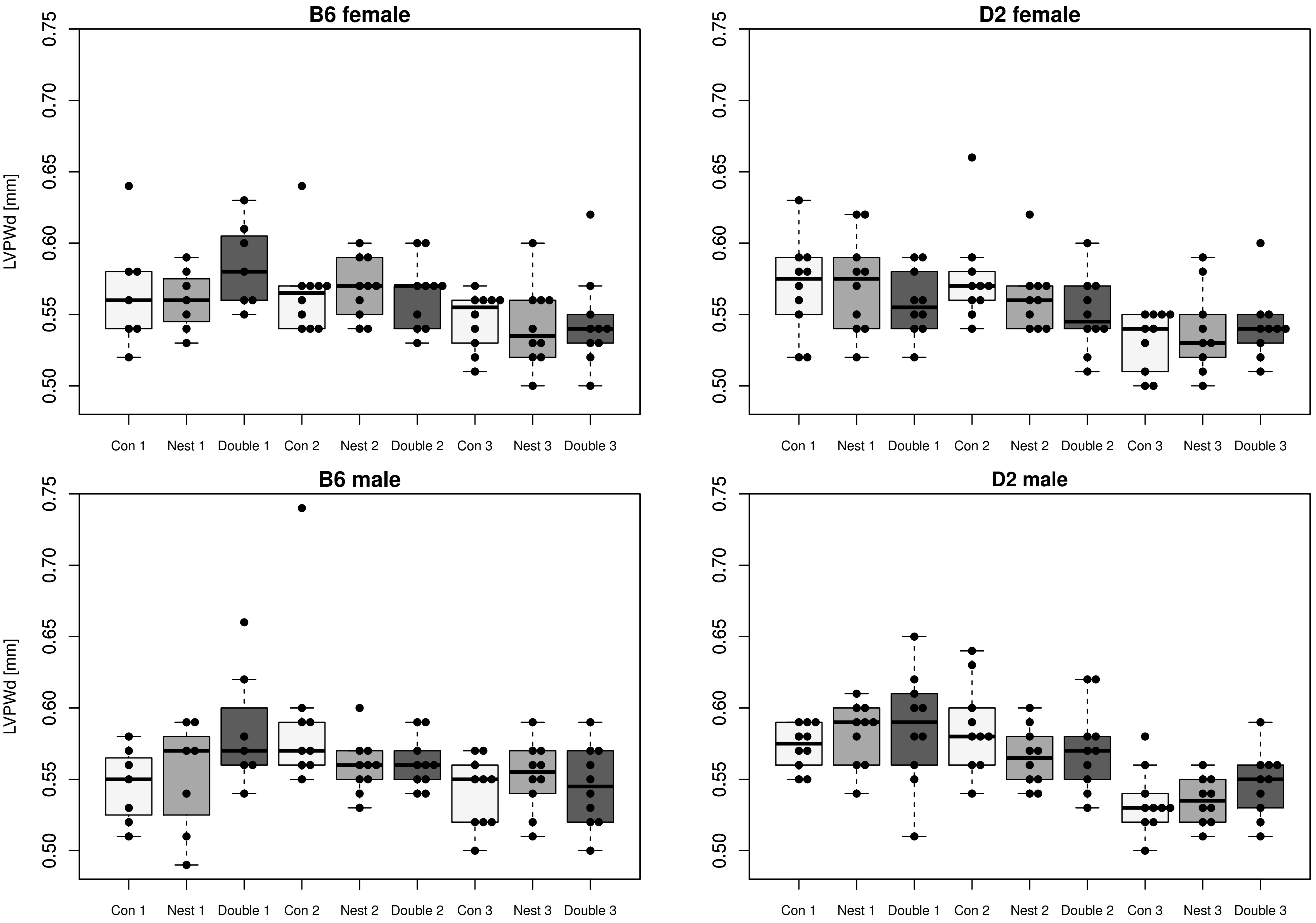

B6 female
D2 female
B6 male
D2 male
LVPWd [mm]
0.50
0.55
0.60
0.65
0.70
0.75
Con 1
Nest 1
Double 1
Con 2
Nest 2
Double 2
Con 3
Nest 3
Double 3

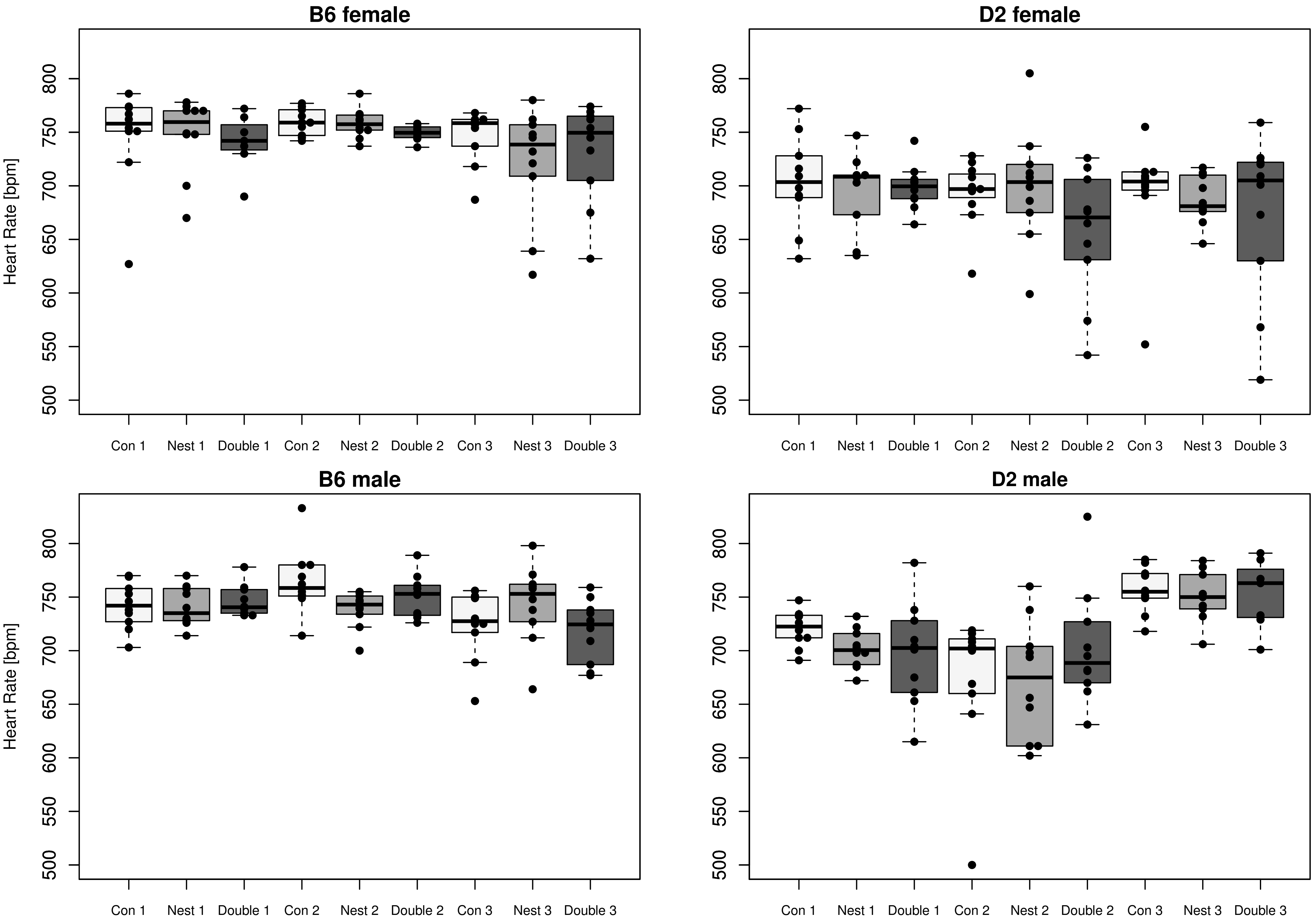

B6 female
D2 female
B6 male
D2 male
Heart Rate [bpm]
500
550
600
650
700
750
800
Con 1
Nest 1
Double 1
Con 2
Nest 2
Double 2
Con 3
Nest 3
Double 3

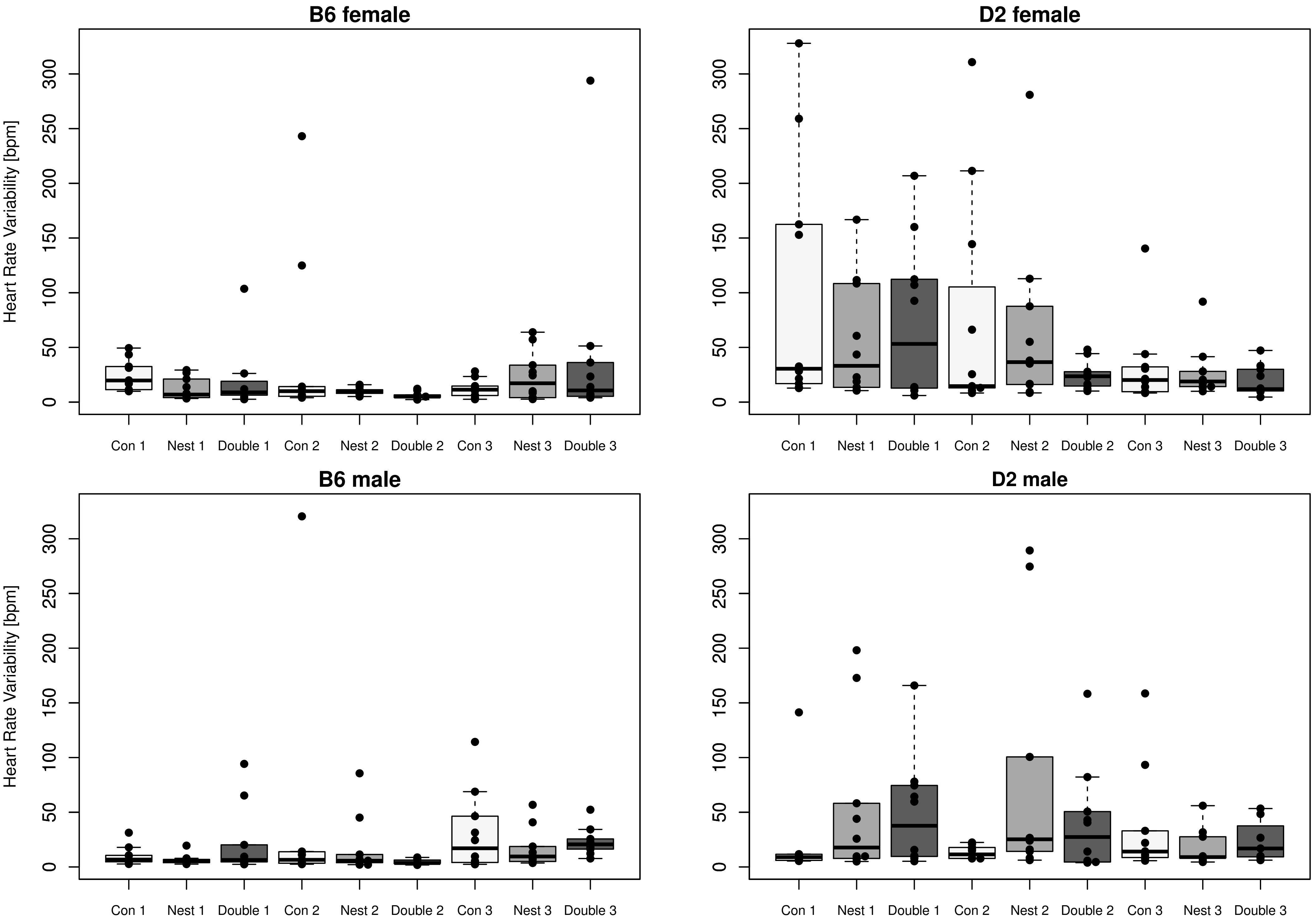
B6 female
D2 female
B6 male
D2 male
Heart Rate Variability [bpm]
0
50
100
150
200
250
300
Con 1
Nest 1
Double 1
Con 2
Nest 2
Double 2
Con 3
Nest 3
Double 3

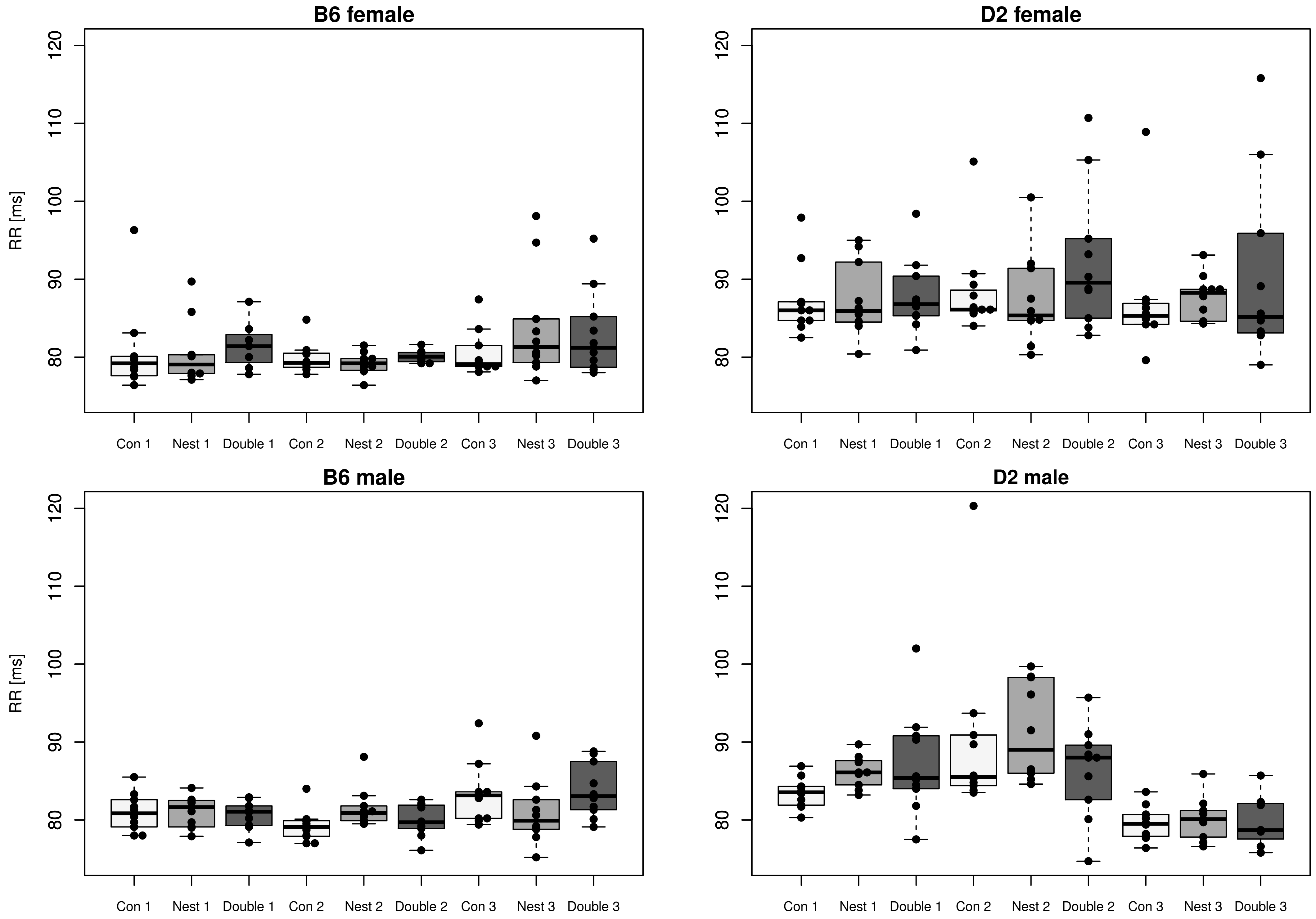

B6 female
D2 female
B6 male
D2 male
RR [ms]
80
90
100
110
120
Con 1
Nest 1
Double 1
Con 2
Nest 2
Double 2
Con 3
Nest 3
Double 3

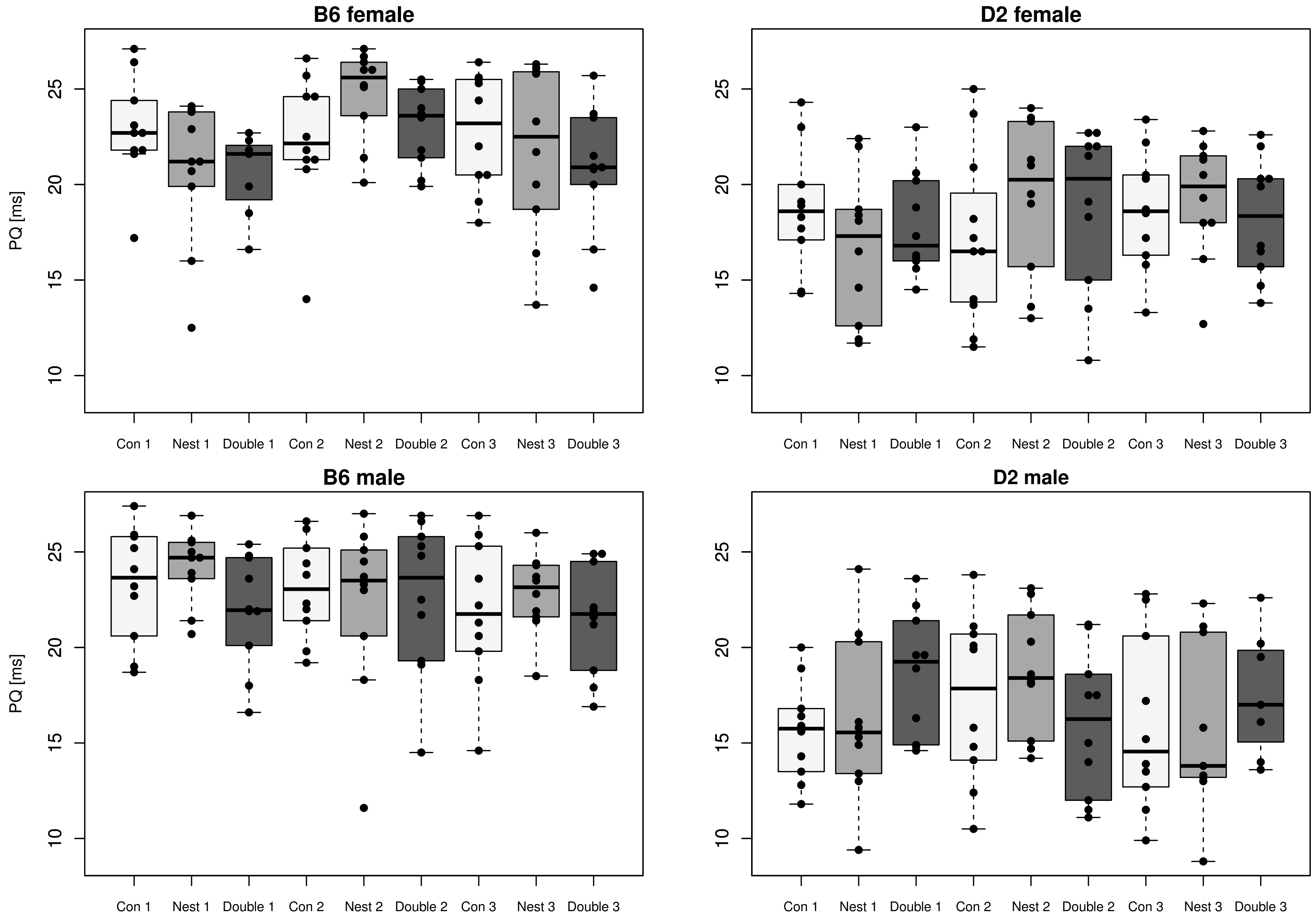

B6 female
D2 female
B6 male
D2 male
PQ [ms]
PQ [ms]
Con 1
Nest 1
Double 1
Con 2
Nest 2
Double 2
Con 3
Nest 3
Double 3

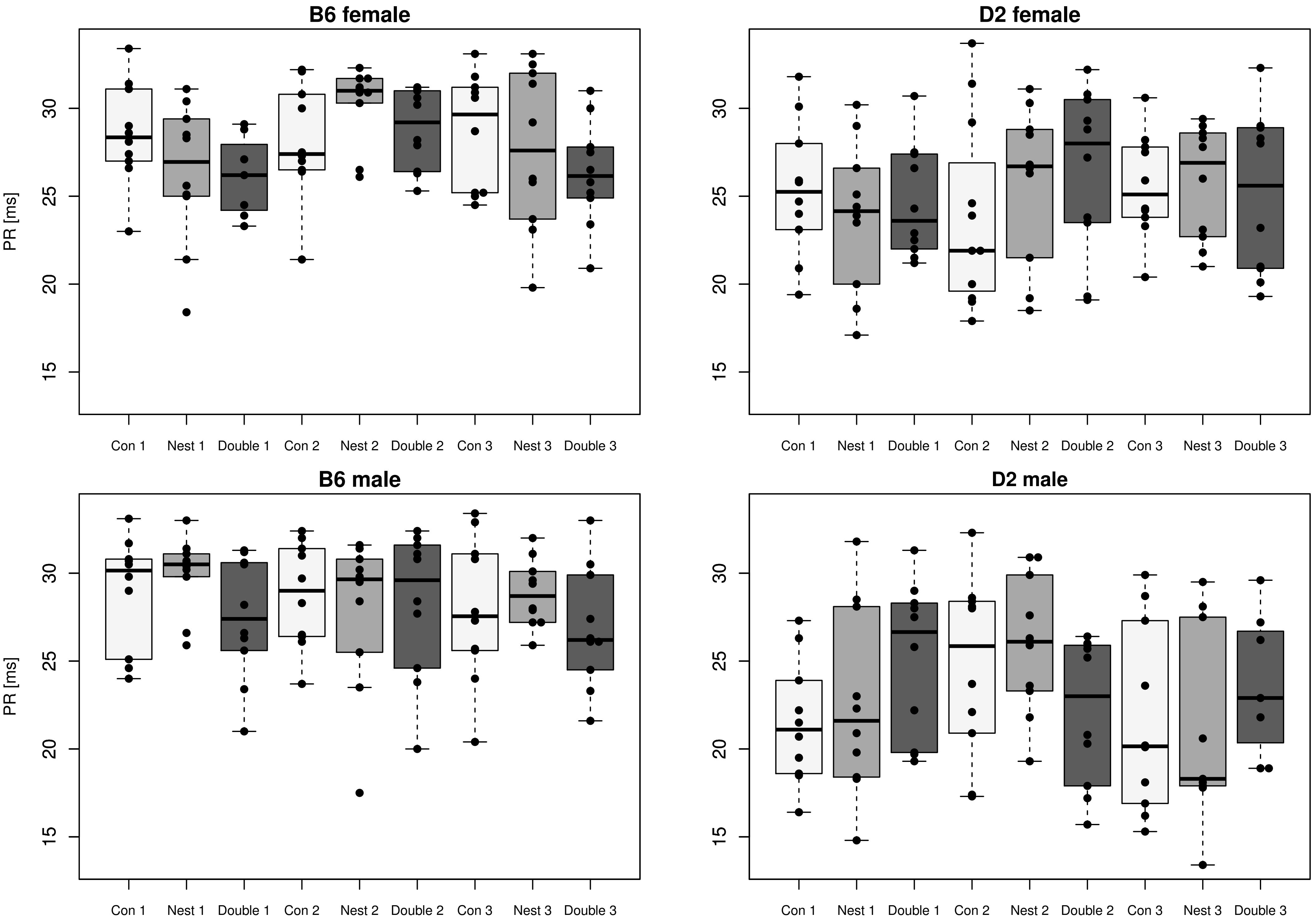
B6 female
D2 female
B6 male
D2 male
PR [ms]
15
20
25
30
Con 1
Nest 1
Double 1
Con 2
Nest 2
Double 2
Con 3
Nest 3
Double 3

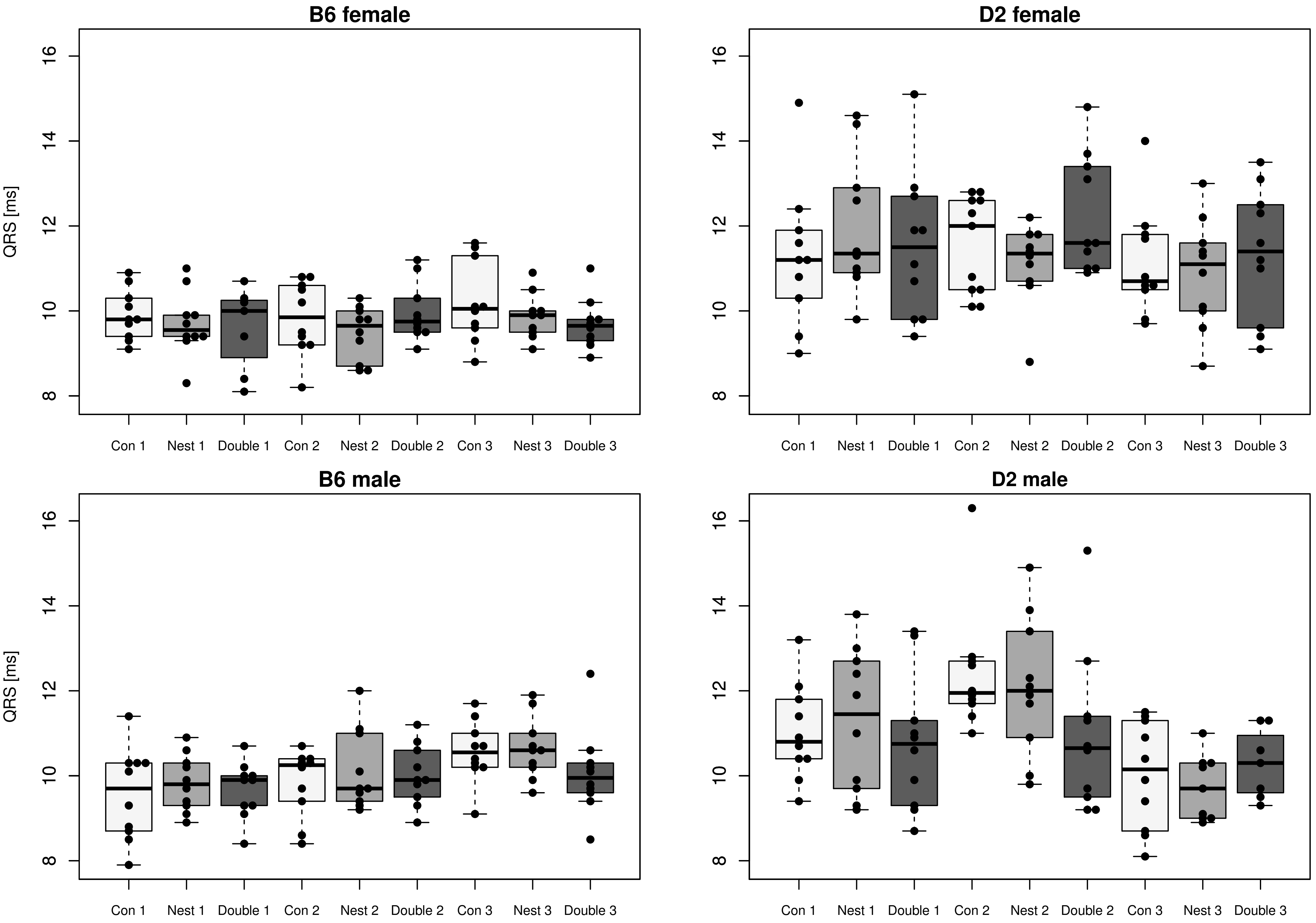

B6 female
D2 female
B6 male
D2 male
QRS [ms]
8
10
12
14
16
Con 1
Nest 1
Double 1
Con 2
Nest 2
Double 2
Con 3
Nest 3
Double 3

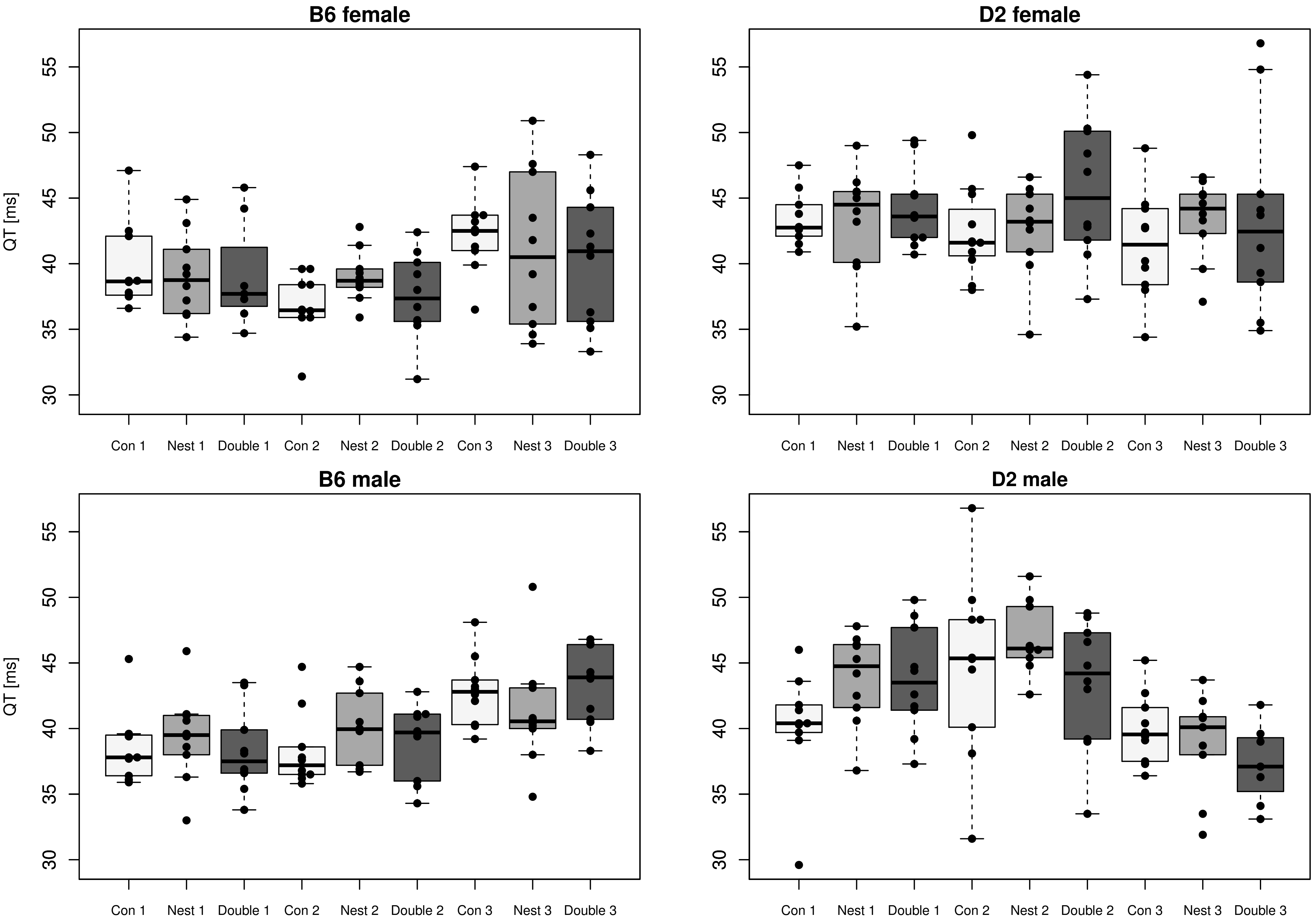

B6 female
D2 female
B6 male
D2 male
QT [ms]
30
35
40
45
50
55
Con 1
Nest 1
Double 1
Con 2
Nest 2
Double 2
Con 3
Nest 3
Double 3

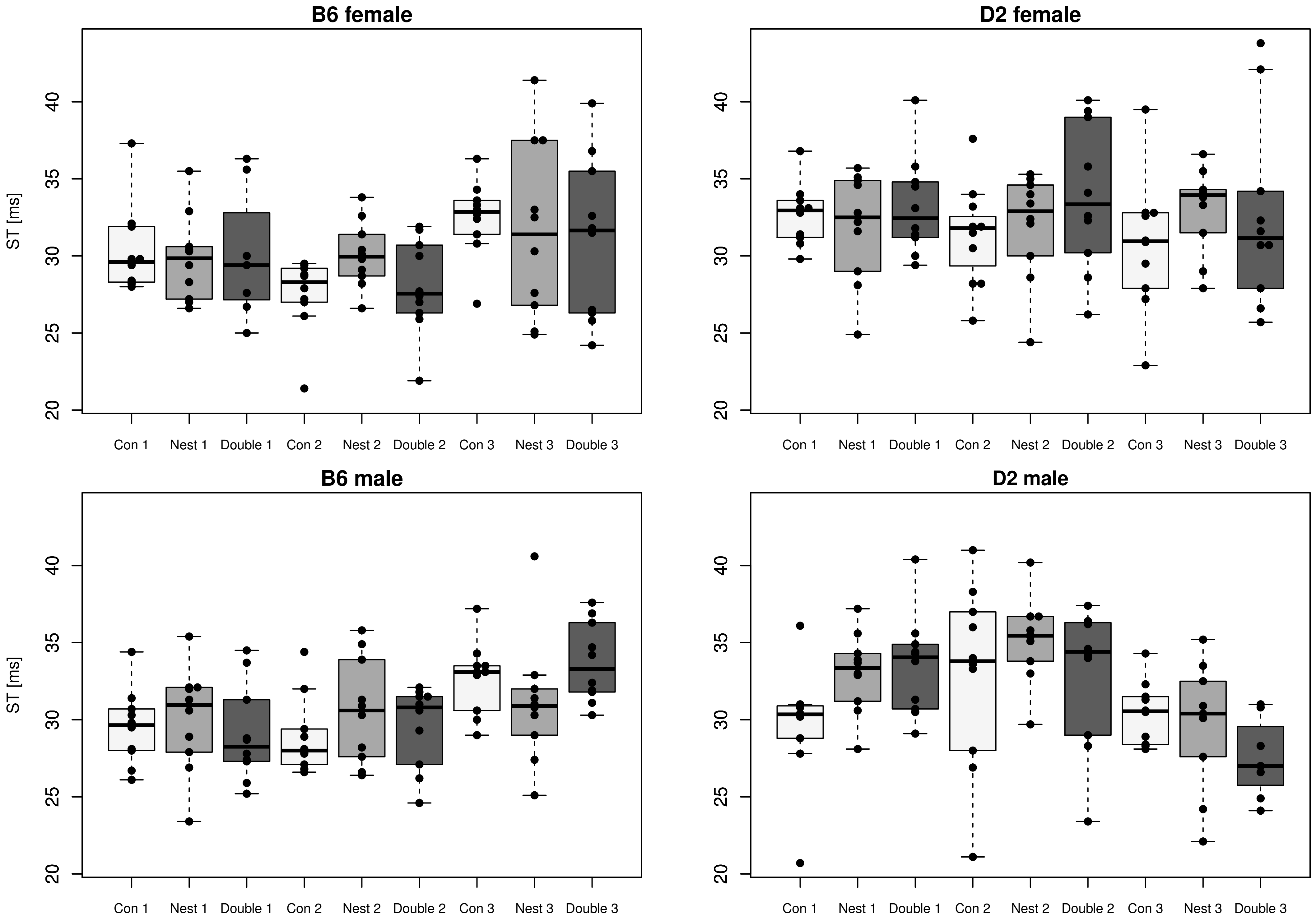
B6 female
D2 female
B6 male
D2 male
ST [ms]
20
25
30
35
40
Con 1
Nest 1
Double 1
Con 2
Nest 2
Double 2
Con 3
Nest 3
Double 3

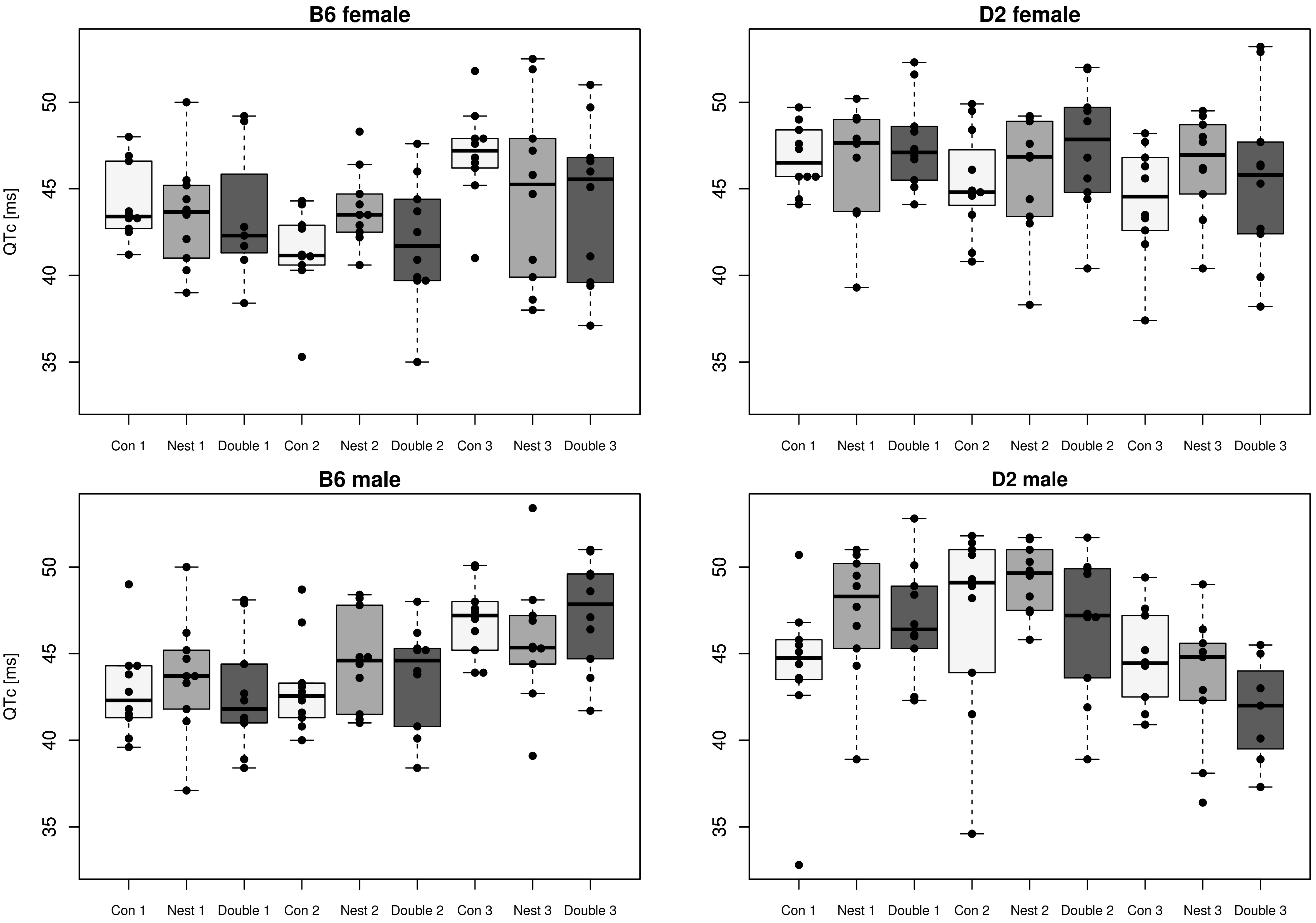

B6 female
D2 female
B6 male
D2 male
QTc [ms]
35
40
45
50
Con 1
Nest 1
Double 1
Con 2
Nest 2
Double 2
Con 3
Nest 3
Double 3

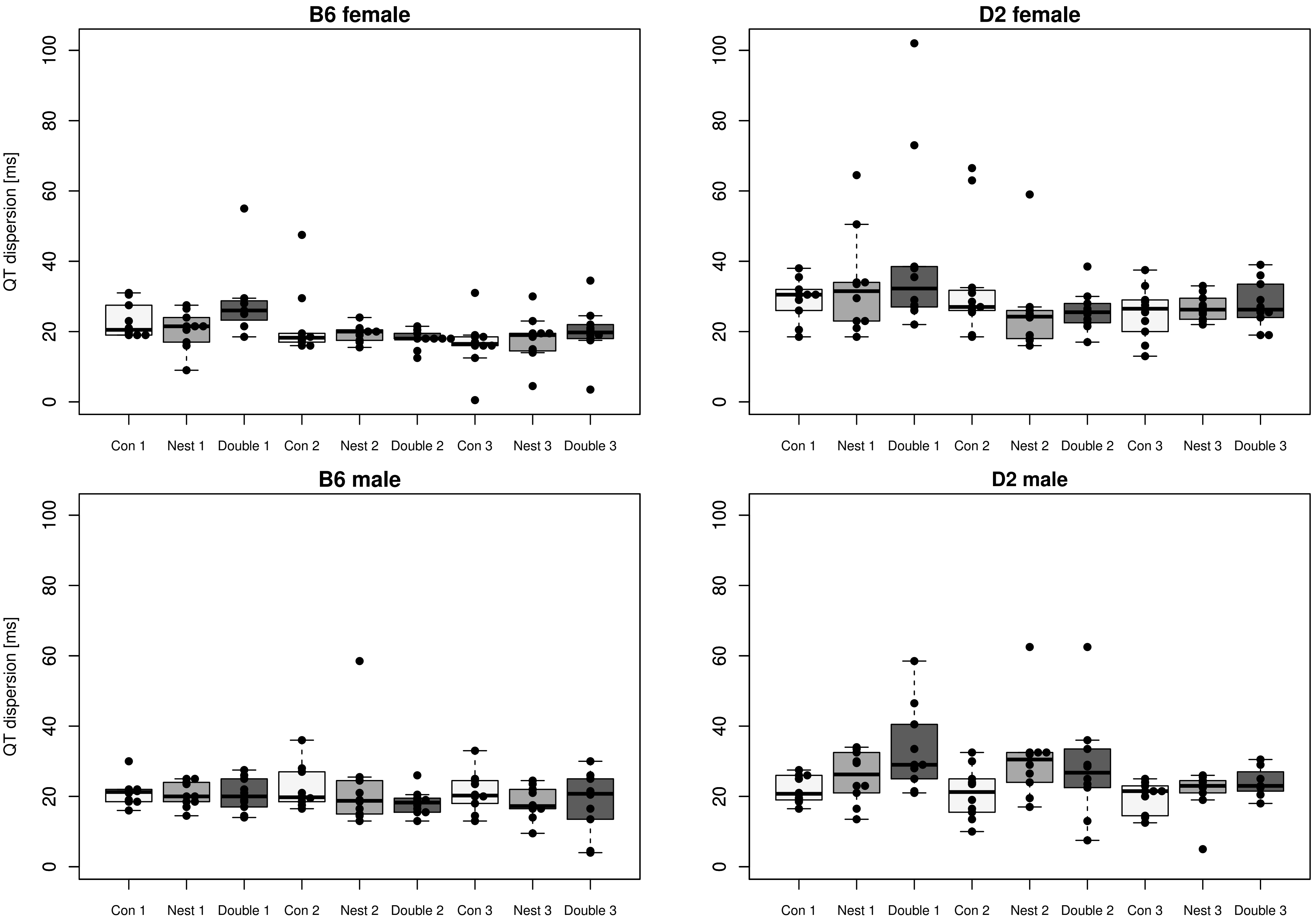
B6 female
D2 female
B6 male
D2 male
QT dispersion [ms]
0
20
40
60
80
100
Con 1
Nest 1
Double 1
Con 2
Nest 2
Double 2
Con 3
Nest 3
Double 3

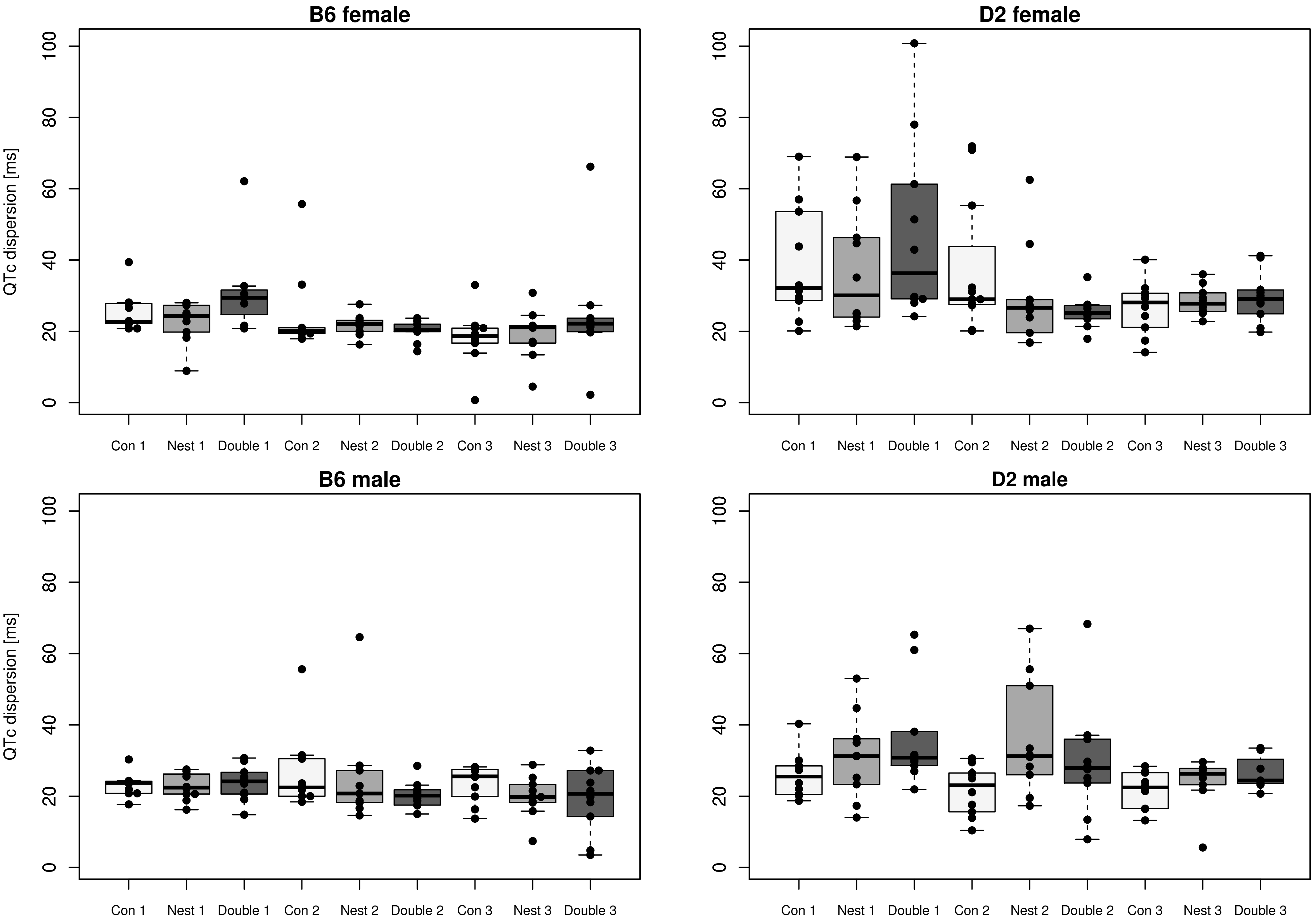
B6 female
D2 female
B6 male
D2 male
QTc dispersion [ms]
0
20
40
60
80
100
Con 1
Nest 1
Double 1
Con 2
Nest 2
Double 2
Con 3
Nest 3
Double 3

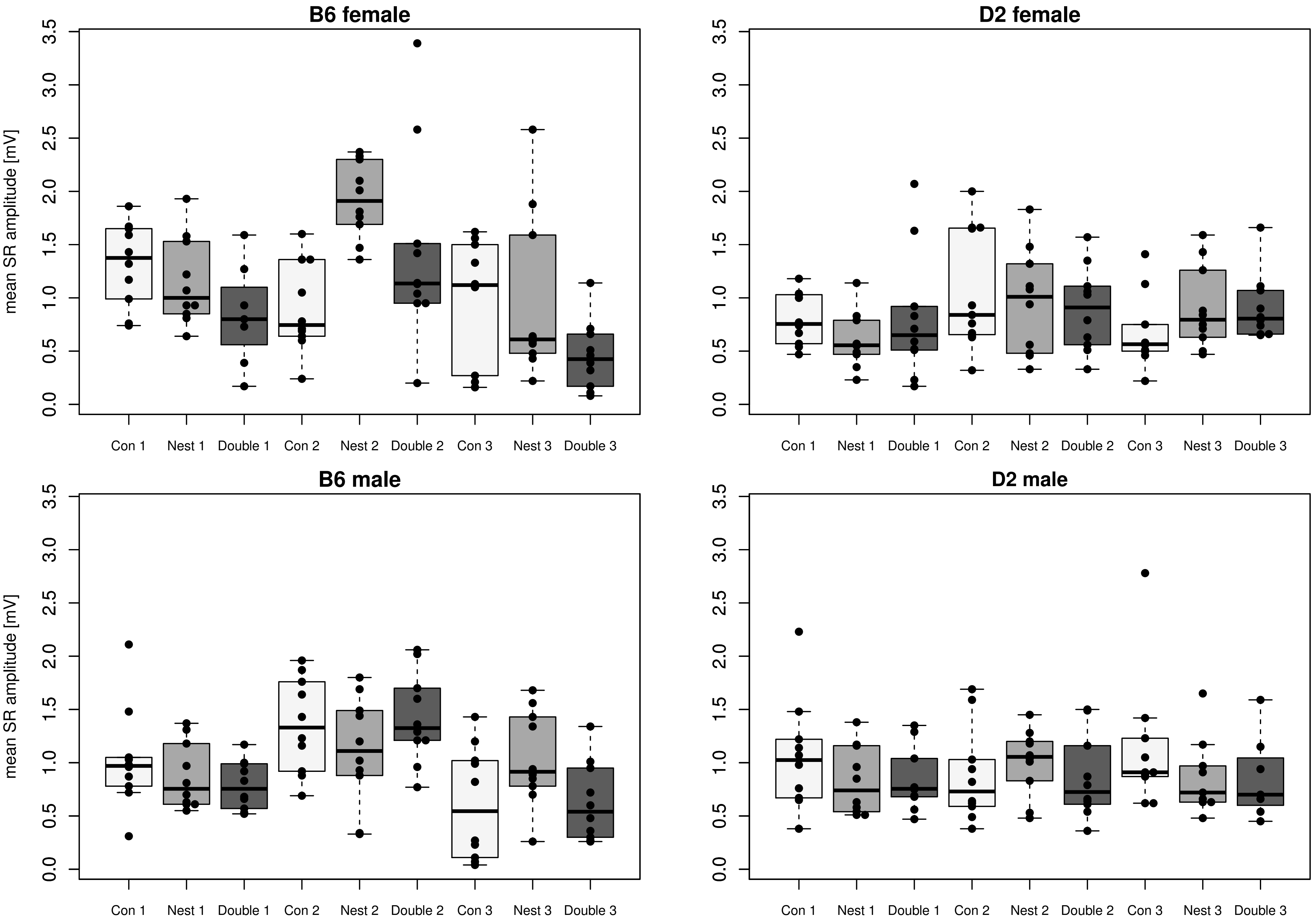
B6 female
D2 female
B6 male
D2 male
mean SR amplitude [mV]
0.0
0.5
1.0
1.5
2.0
2.5
3.0
3.5
Con 1
Nest 1
Double 1
Con 2
Nest 2
Double 2
Con 3
Nest 3
Double 3

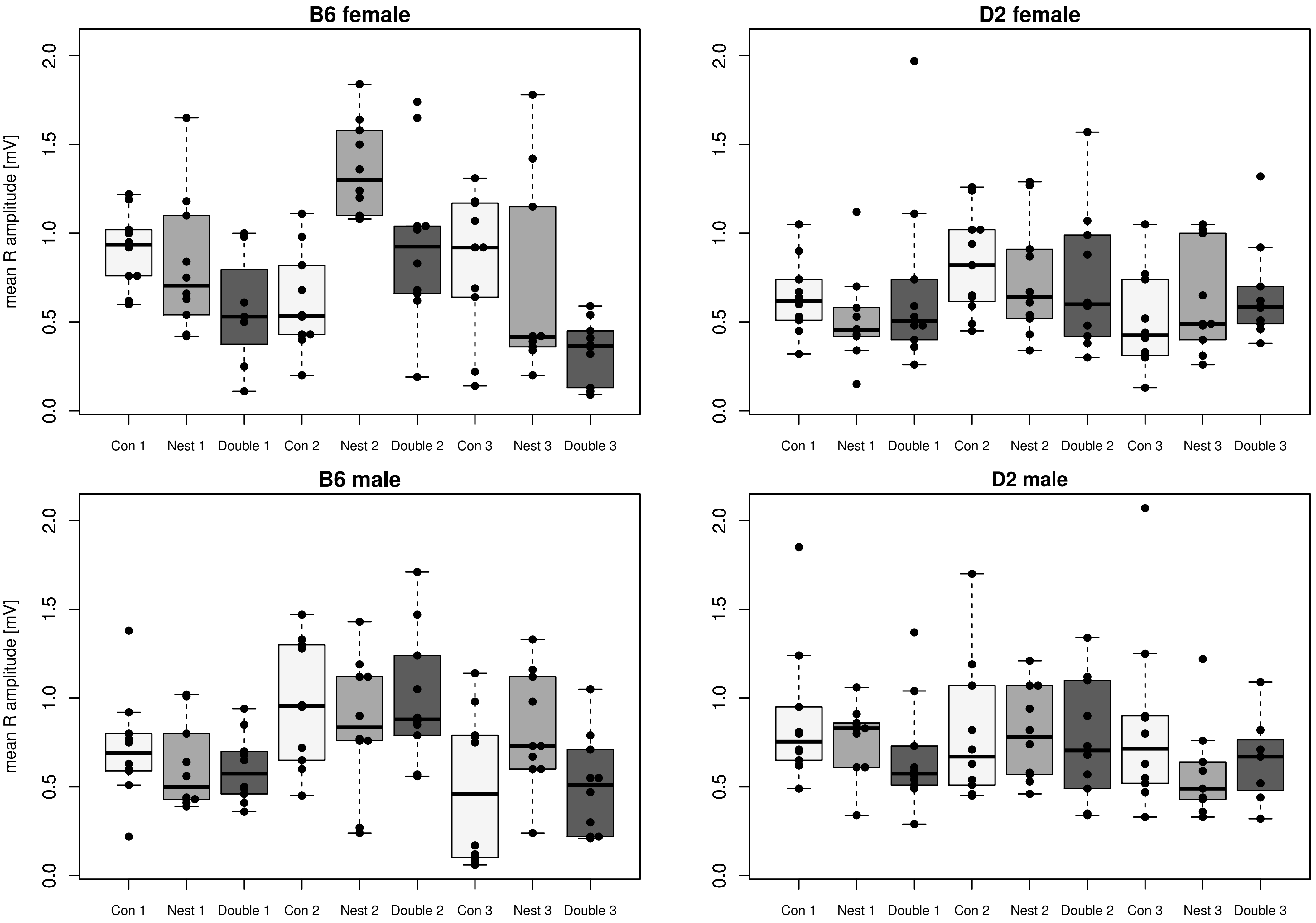
B6 female
D2 female
B6 male
D2 male
mean R amplitude [mV]
0.0
0.5
1.0
1.5
2.0
Con 1
Nest 1
Double 1
Con 2
Nest 2
Double 2
Con 3
Nest 3
Double 3

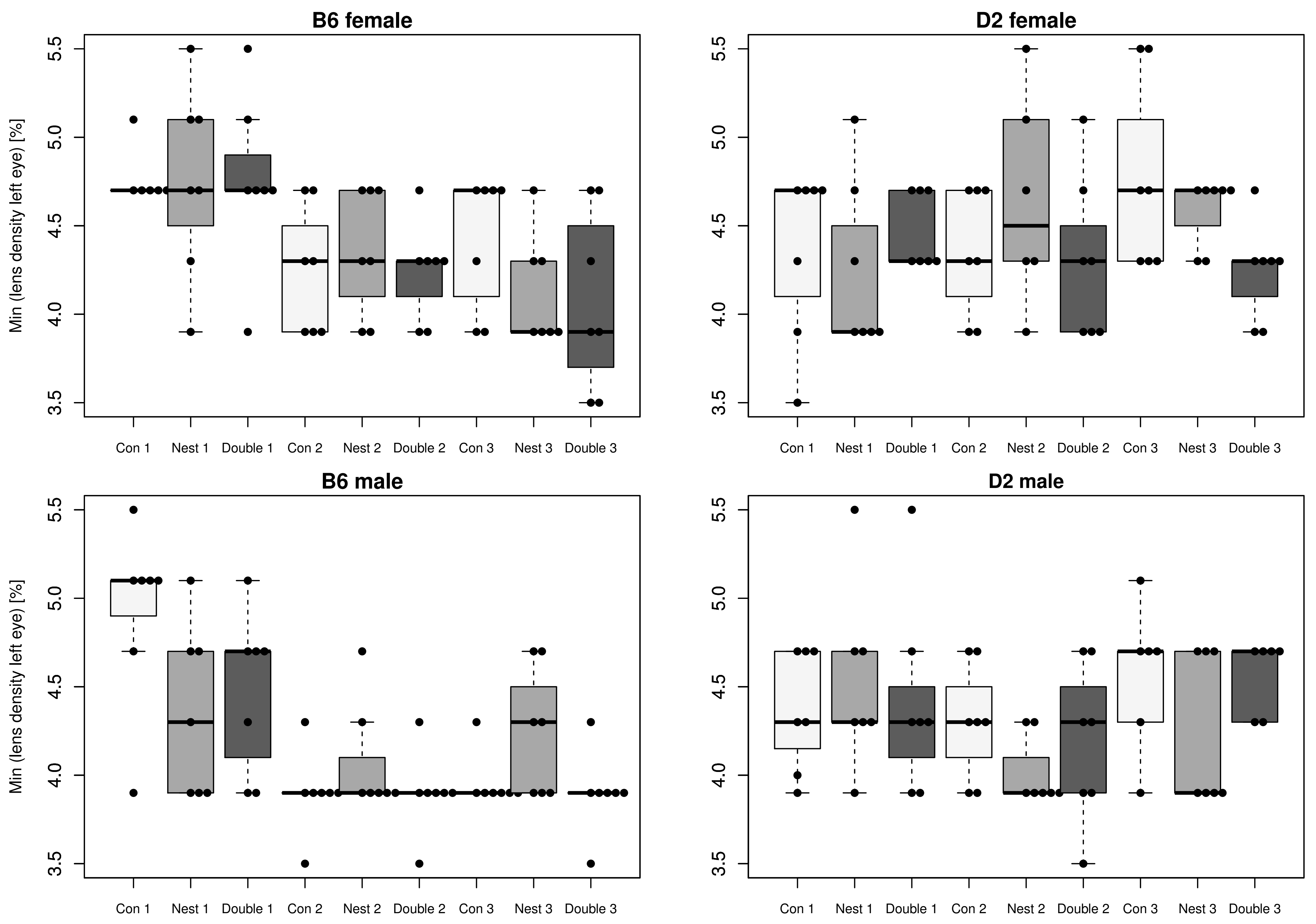
B6 female
D2 female
B6 male
D2 male
Min (lens density left eye) [%]
3.5
4.0
4.5
5.0
5.5
Con 1
Nest 1
Double 1
Con 2
Nest 2
Double 2
Con 3
Nest 3
Double 3

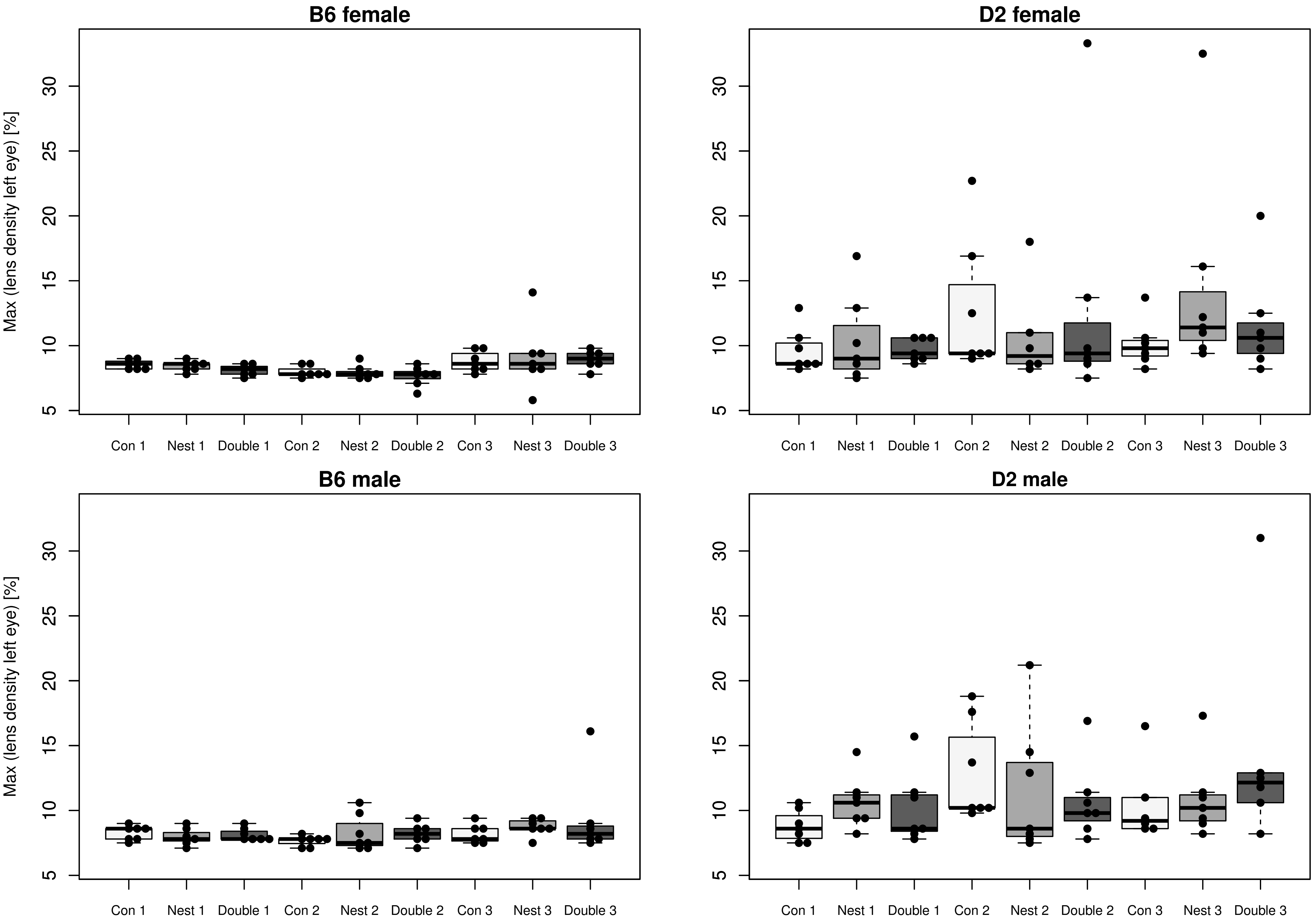
B6 female
D2 female
B6 male
D2 male
Max (lens density left eye) [%]
5
10
15
20
25
30
Con 1
Nest 1
Double 1
Con 2
Nest 2
Double 2
Con 3
Nest 3
Double 3

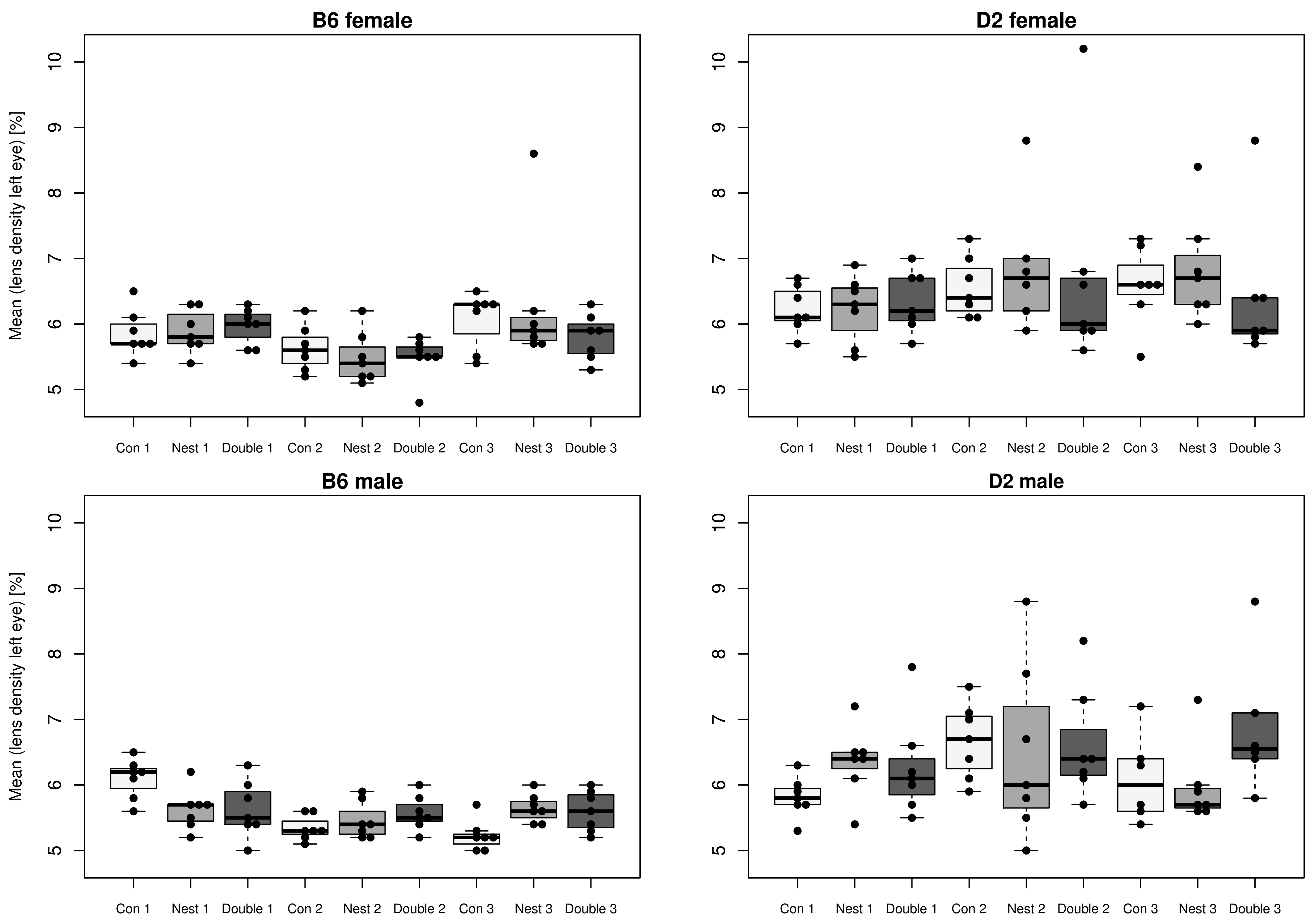
B6 female
D2 female
B6 male
D2 male
Mean (lens density left eye) [%]
5
6
7
8
9
10
Con 1
Nest 1
Double 1
Con 2
Nest 2
Double 2
Con 3
Nest 3
Double 3

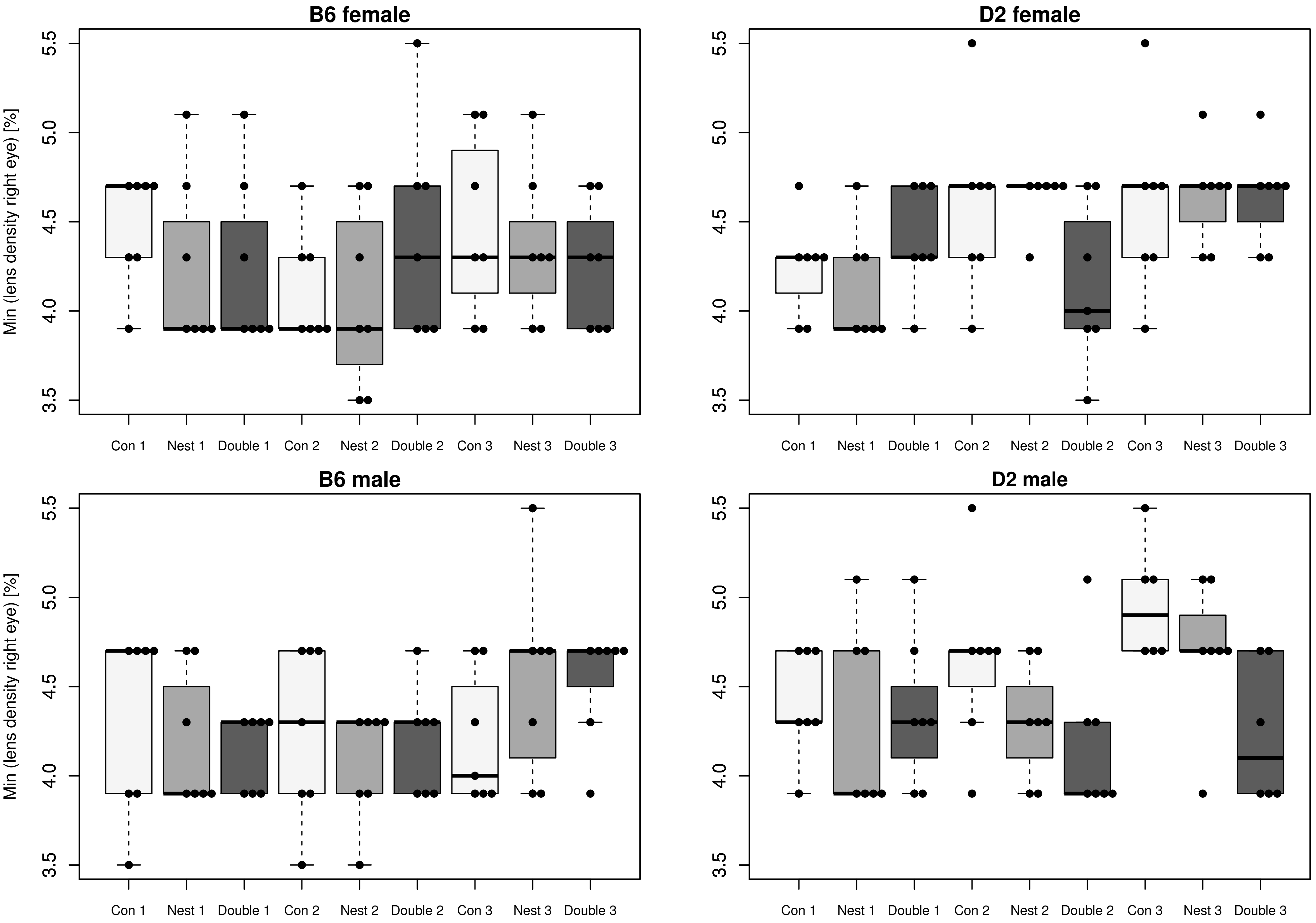
B6 female
D2 female
B6 male
D2 male
Min (lens density right eye) [%]
3.5
4.0
4.5
5.0
5.5
Con 1
Nest 1
Double 1
Con 2
Nest 2
Double 2
Con 3
Nest 3
Double 3

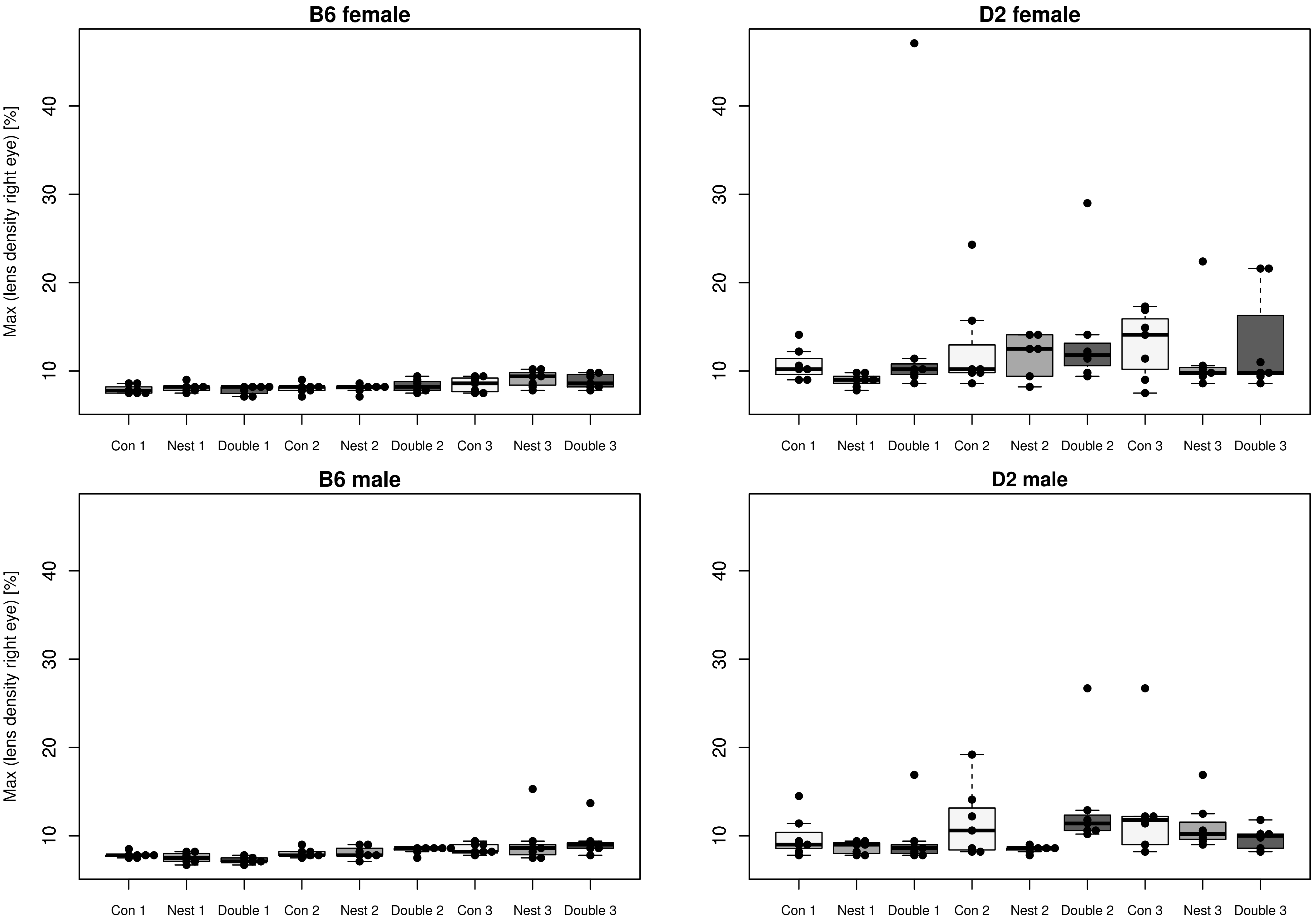

B6 female
D2 female
B6 male
D2 male
Max (lens density right eye) [%]
10
20
30
40
Con 1
Nest 1
Double 1
Con 2
Nest 2
Double 2
Con 3
Nest 3
Double 3

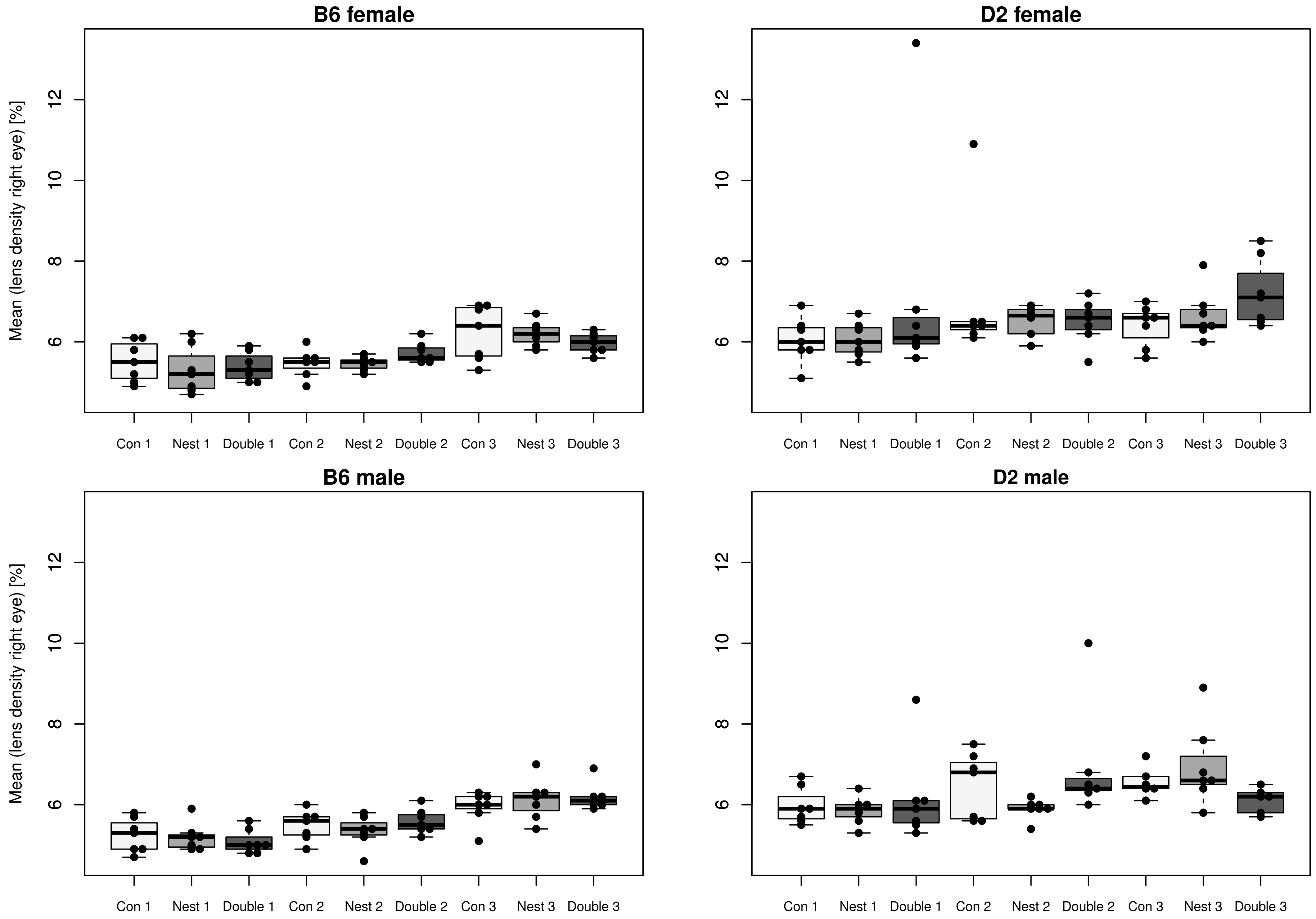
B6 female
D2 female
B6 male
D2 male
Mean (lens density right eye) [%]
12
10
8
6
Con 1
Nest 1
Double 1
Con 2
Nest 2
Double 2
Con 3
Nest 3
Double 3

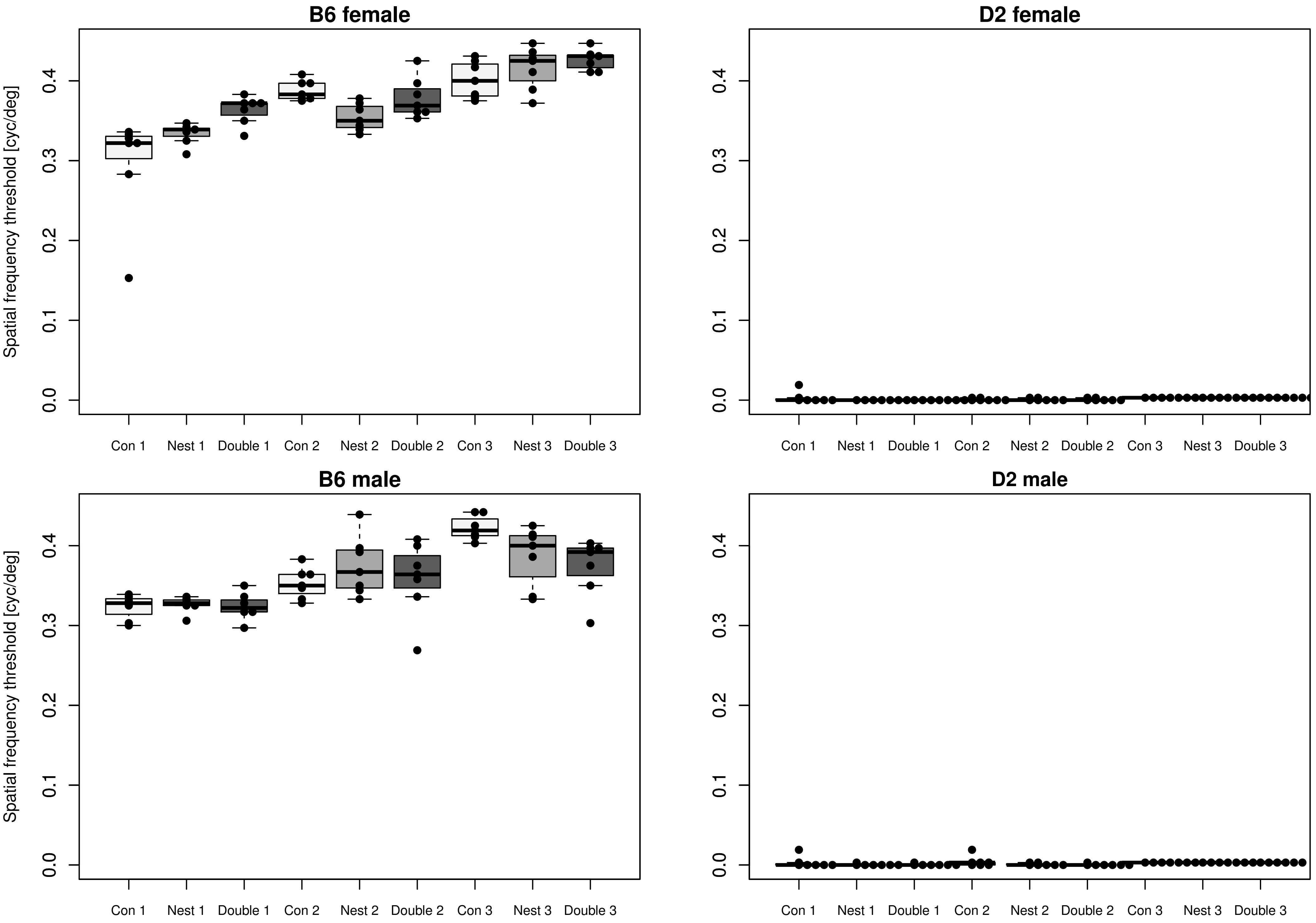
B6 female
D2 female
B6 male
D2 male
Spatial frequency threshold [cyc/deg]
0.0
0.1
0.2
0.3
0.4
Con 1
Nest 1
Double 1
Con 2
Nest 2
Double 2
Con 3
Nest 3
Double 3

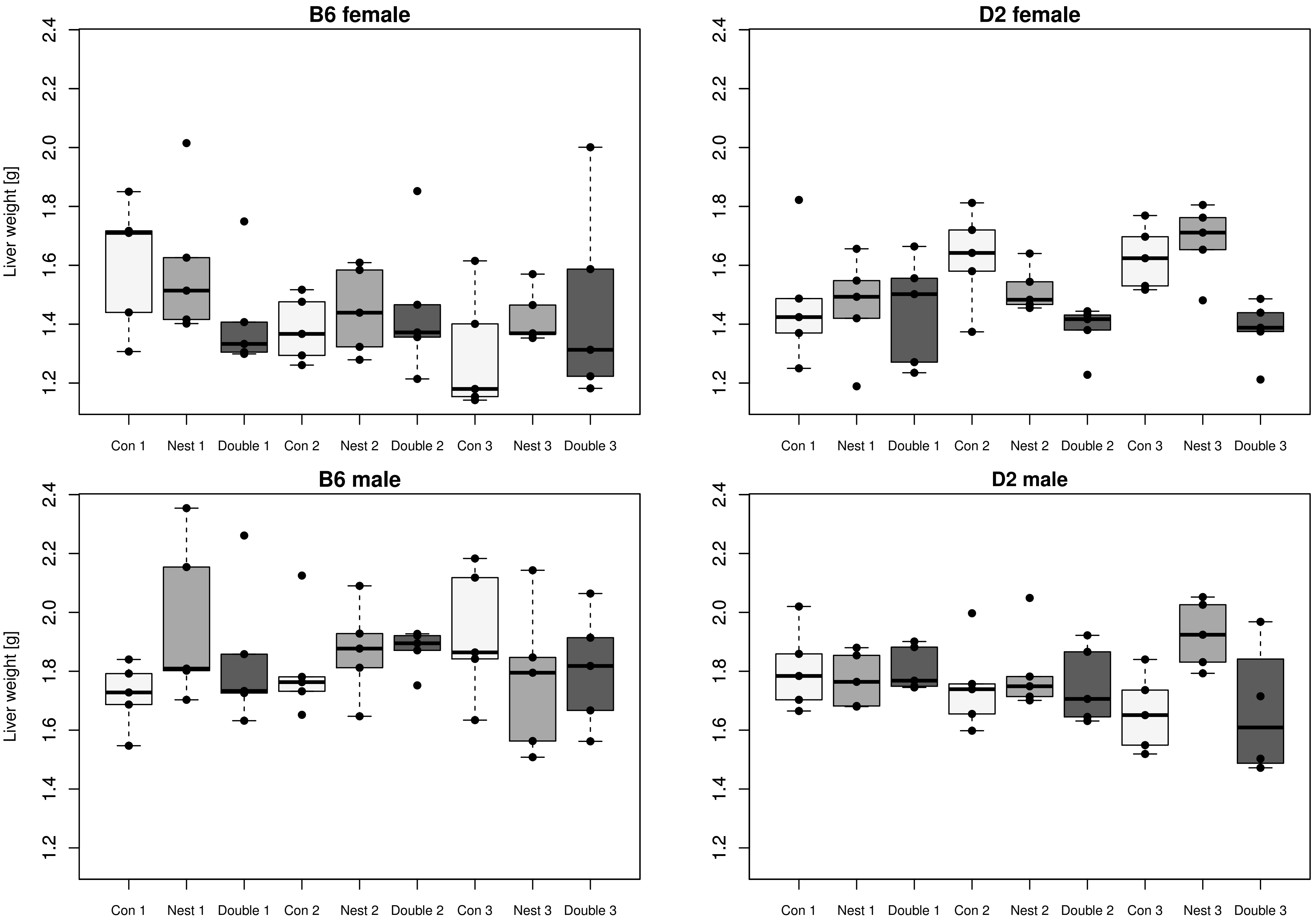

B6 female
D2 female
B6 male
D2 male
Liver weight [g]
1.2
1.4
1.6
1.8
2.0
2.2
2.4
Con 1
Nest 1
Double 1
Con 2
Nest 2
Double 2
Con 3
Nest 3
Double 3

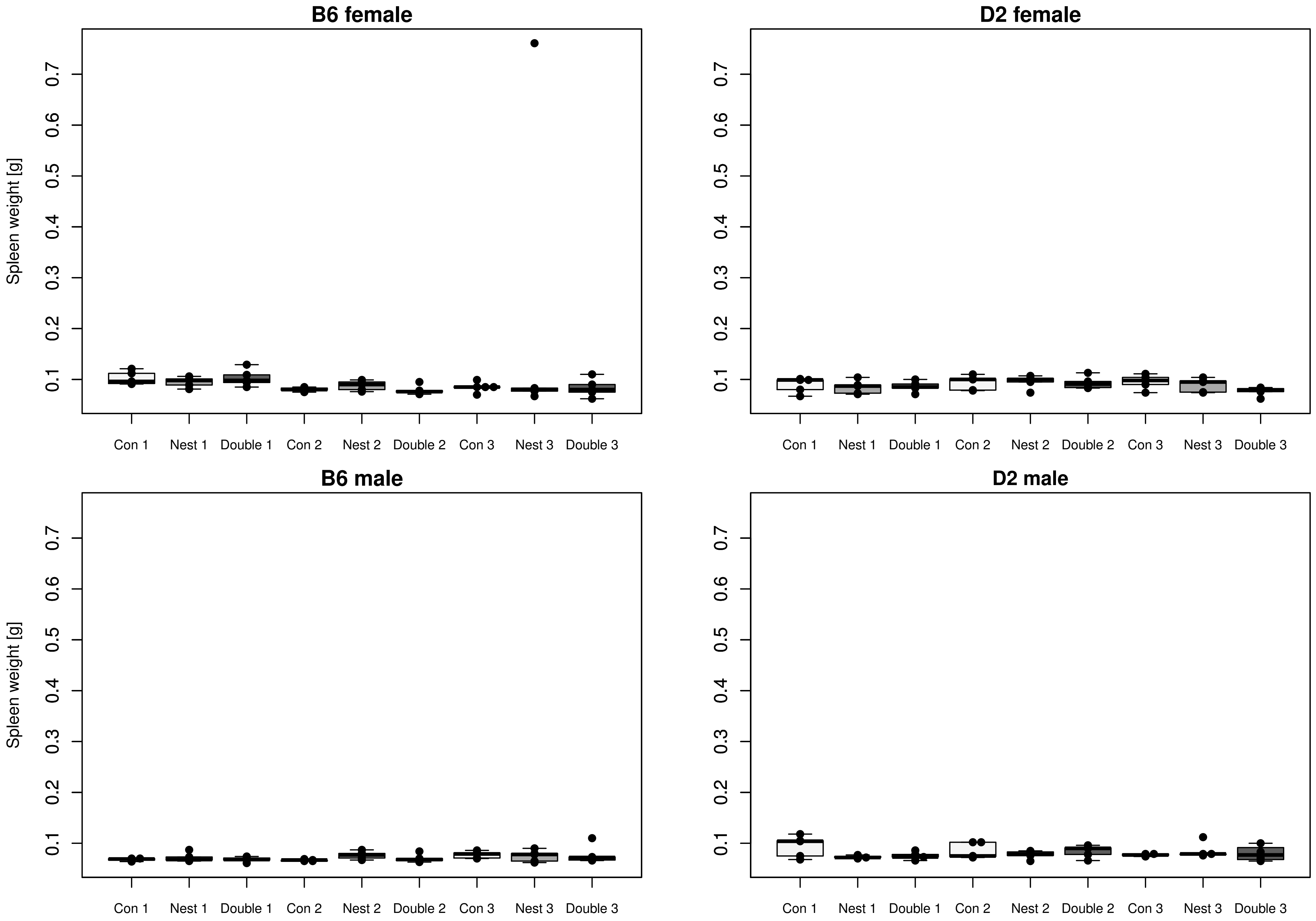
B6 female
D2 female
B6 male
D2 male
Spleen weight [g]
0.1
0.2
0.3
0.4
0.5
0.6
0.7
Con 1
Nest 1
Double 1
Con 2
Nest 2
Double 2
Con 3
Nest 3
Double 3

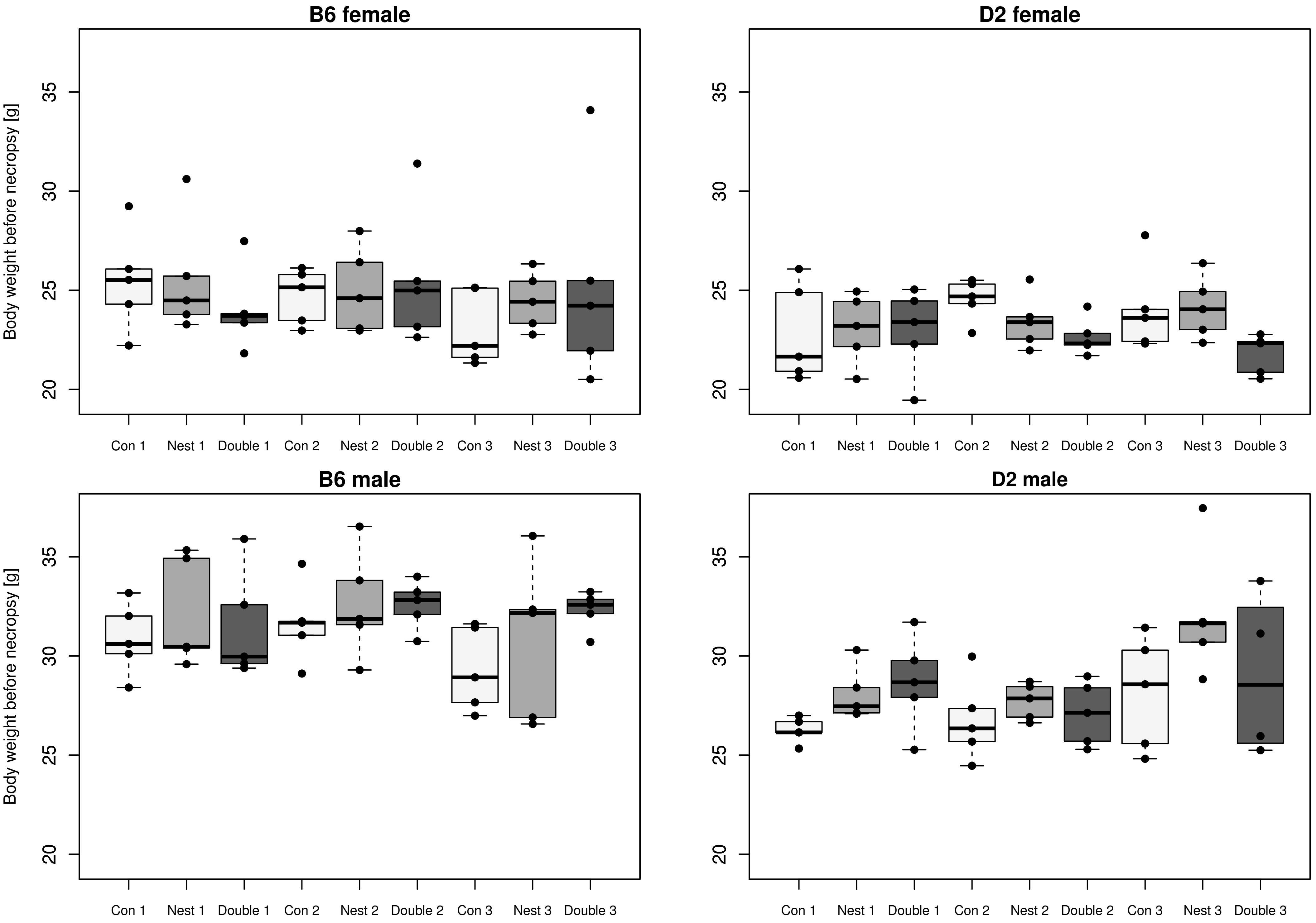
B6 female
D2 female
B6 male
D2 male
Body weight before necropsy [g]
20
25
30
35
Con 1
Nest 1
Double 1
Con 2
Nest 2
Double 2
Con 3
Nest 3
Double 3

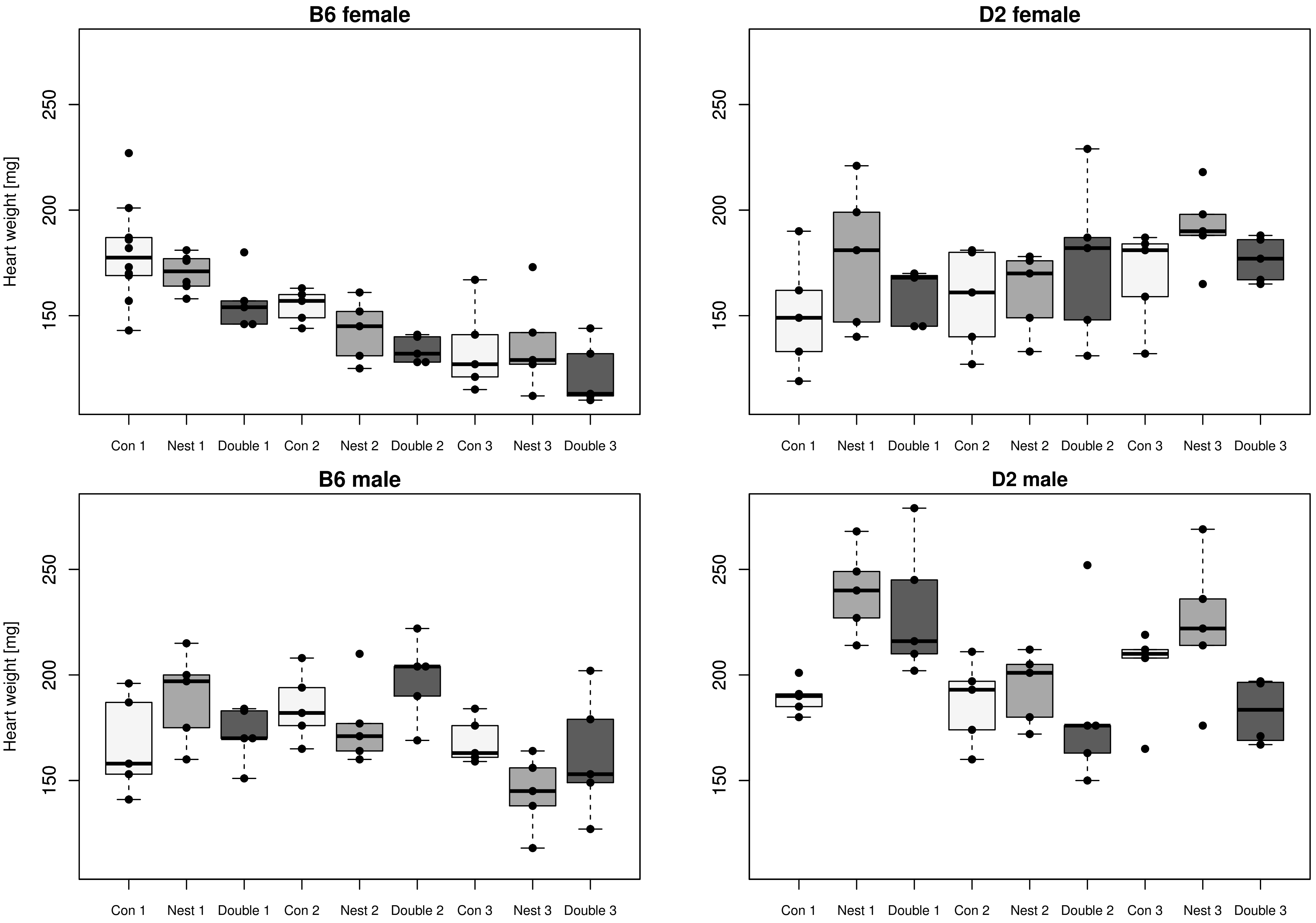
B6 female
D2 female
B6 male
D2 male
Heart weight [mg]
150
200
250
Con 1
Nest 1
Double 1
Con 2
Nest 2
Double 2
Con 3
Nest 3
Double 3

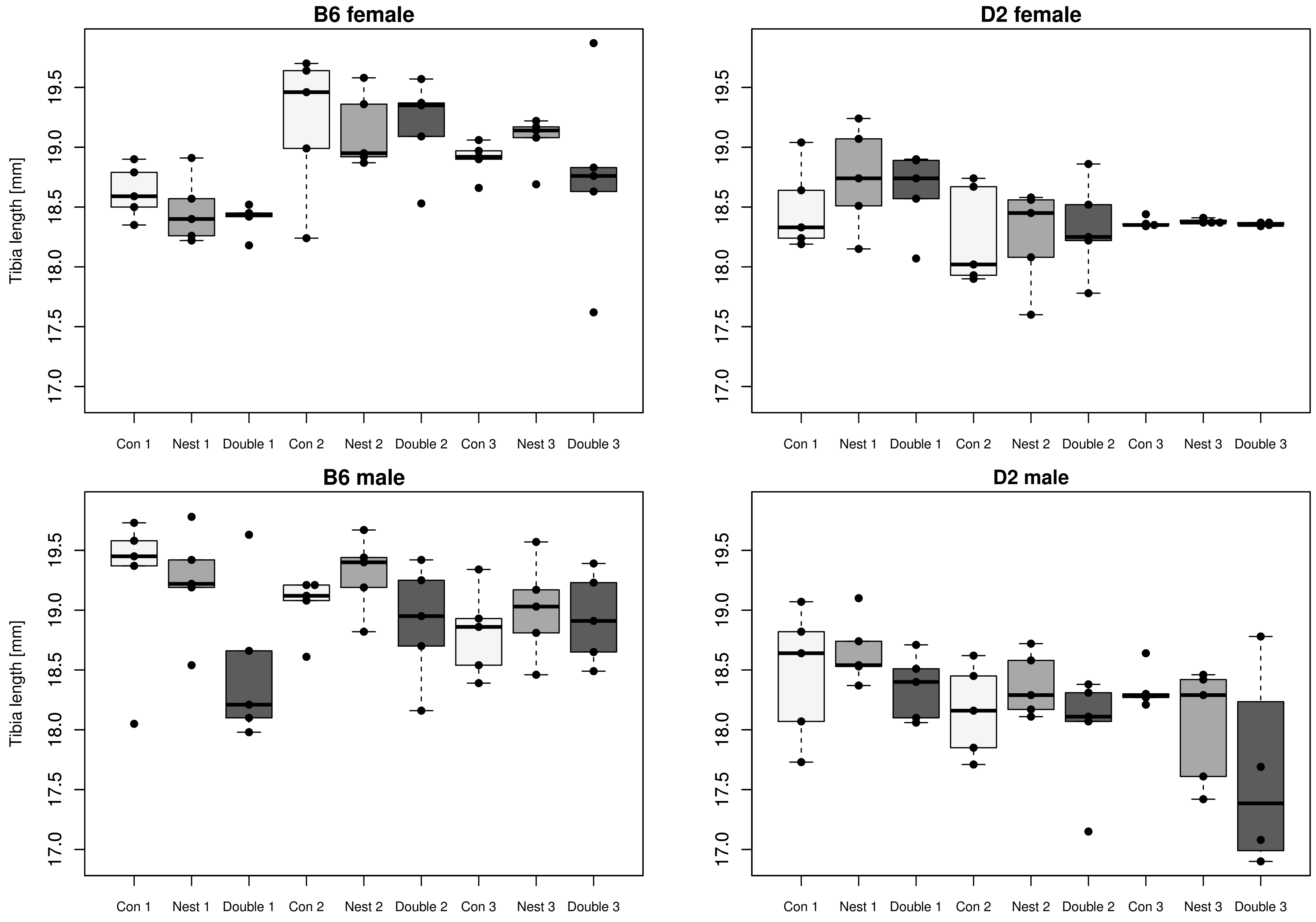

B6 female
D2 female
B6 male
D2 male
Tibia length [mm]
17.0
17.5
18.0
18.5
19.0
19.5
Con 1
Nest 1
Double 1
Con 2
Nest 2
Double 2
Con 3
Nest 3
Double 3
