# Supplementary figures and images for: Laboratory mouse housing conditions can be improved using common environmental enrichment without compromising data
Source: PLoS Biol. 2018 Apr 16;16(4):e2005019. doi: 10.1371/journal.pbio.2005019 (PMC5922977; doi:10.1371/journal.pbio.2005019)

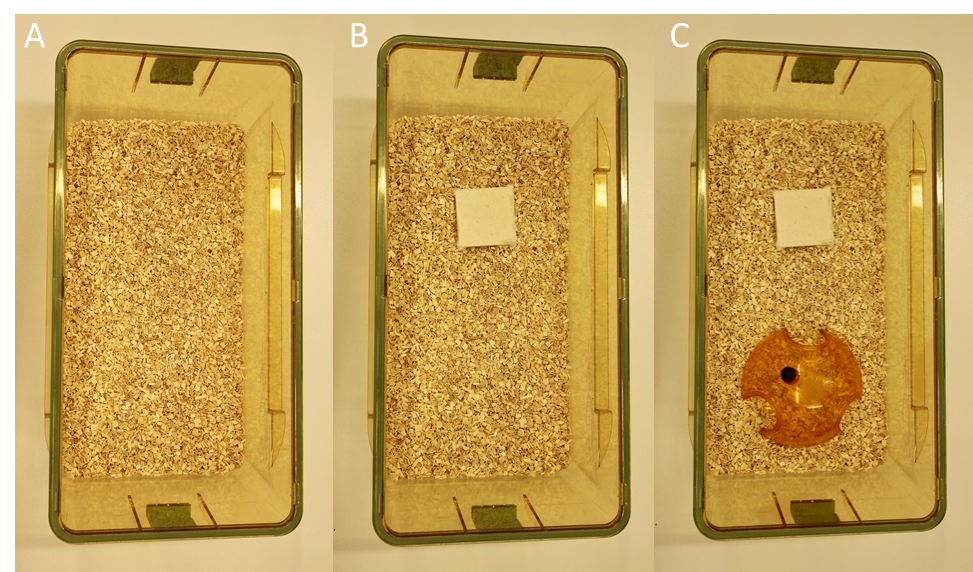

Supplement: S2 Fig — (A) “Control” group without any items; (B) group “nest” with 1 cotton nestlet (PLEXX, Article ref. 14010); (C) group “double” with 1 cotton nestlet plus 1 plastic mouse igloo (PLEXX, Article ref. 13100). (PNG) [file pbio.2005019.s003.png]
