# Supplementary material for: Laboratory mouse housing conditions can be improved using common environmental enrichment without compromising data
Source: PLoS Biol. 2018 Apr 16;16(4):e2005019. doi: 10.1371/journal.pbio.2005019 (PMC5922977; doi:10.1371/journal.pbio.2005019)
Supplement: S1 Table — Results are presented as number of animals receiving the parameter-specific score in combined cohorts and are described as follows: (x/y/z) with x = number of animals with score “0”; y = number of animals with score “1”; z = number of animals with score “2.” Specifications for scores: body position: 0 = inactive; 1 = active; 2 = excessively active; tremor: 0 = absent; 1 = present; defecation: 0 = present; 1 = absent; transfer arousal: 0 = prolonged freeze; 1 = brief freeze; 2 = immediate movement; gait: 0 = fluid; 1 = abnormal; tail elevation: 0 = dragging; 1 = horizontal; 2 = elevated; startle response: 0 = no reaction; 1 = Preyer reflex; 2 = jumping; touch escape: 0 = no response; 1 = response to touch; 2 = flees prior to touch; trunk curl: 0 = absent; 1 = present; limb grasping: 0 = absent; 1 = present; pinna reflex: 0 = present; 1 = absent; urination: 0 = present; 1 = absent; contact righting reflex: 0 = present; 1 = absent; evidence of biting: 0 = no aggression; 1 = aggressive; vocalization: 0 = no; 1 = yes. SHIRPA, Smithkline Beecham, MRC Harwell, Imperial College, the Royal London hospital Phenotype Assessment. (PDF) [file pbio.2005019.s004.pdf]

S1 Table. **Results of SHIRPA (neurology screen).**

strain	B6						D2					
experimental group	control		nest		double		control		nest		double	
sex	m	f	m	f	m	f	m	f	m	f	m	f
body position	0/30/0	0/30/0	0/30/0	0/30/0	0/30/0	0/30/0	0/30/0	0/30/0	0/30/0	0/30/0	0/30/0	0/30/0
tremor	30/0	30/0	29/1	30/0	30/0	30/0	30/0	30/0	30/0	30/0	30/0	30/0
defecation	20/10	16/14	14/16	19/11	20/10	17/13	11/19	18/12	14/16	21/9	17/13	26/4
transfer arousal	0/23/7	0/13/17	0/19/11	0/16/14	0/22/8	0/17/13	0/27/3	0/29/1	0/28/2	0/29/1	0/29/1	0/29/1
gait	30/0	30/0	30/0	30/0	30/0	30/0	30/0	30/0	30/0	30/0	30/0	30/0
tail elevation	0/21/9	0/23/7	0/20/10	0/23/7	0/21/9	0/29/1	0/30/0	0/30/0	0/30/0	0/30/0	0/30/0	0/30/0
startle response	0/30/0	0/30/0	0/30/0	0/30/0	0/29/1	0/30/0	0/30/0	1/29/0	2/28/0	0/30/0	0/30/0	0/30/0
touch escape	0/19/11	0/21/9	0/25/5	0/25/5	0/22/8	0/23/7	0/23/7	0/24/6	0/25/5	0/22/8	0/22/8	0/25/5
trunk curl	30/0	30/0	30/0	30/0	30/0	30/0	30/0	30/0	30/0	30/0	30/0	30/0
limb grasping	30/0	30/0	30/0	30/0	30/0	30/0	30/0	30/0	30/0	30/0	30/0	30/0
pinna reflex	30/0	30/0	30/0	30/0	30/0	30/0	30/0	30/0	30/0	30/0	30/0	30/0
urination	8/22	4/26	9/21	11/19	8/22	6/24	6/24	1/29	5/25	6/24	4/26	4/26
contact righting reflex	30/0	30/0	30/0	30/0	30/0	30/0	30/0	30/0	30/0	30/0	30/0	30/0
evidence of biting	30/0	30/0	30/0	30/0	30/0	29/1	30/0	30/0	30/0	30/0	30/0	30/0
vocalisation	25/5	25/5	26/4	23/7	23/7	28/2	27/3	28/2	26/4	27/3	28/2	28/2

Results are presented as number of animals receiving the parameter-specific score in combined cohorts and are described as following: (x/y/z) with x= number of animals with score “0”, y= number of animals with score “1”, z= number of animals with score “2”. Specifications for scores: body position: 0= inactive, 1= active, 2= excessively active; tremor: 0= absent, 1= present; defecation: 0= present, 1= absent; transfer arousal: 0= prolonged freeze, 1= brief freeze, 2= immediate movement; gait: 0= fluid, 1= abnormal; tail elevation: 0= dragging, 1= horizontal, 2= elevated; startle response: 0= no reaction, 1= Preyer reflex, 2= jumping; touch escape: 0= no response, 1= response to touch, 2= flees prior to touch; trunk curl: 0= absent, 1= present; limb grasping: 0= absent, 1= present; pinna reflex: 0= present, 1= absent; urination: 0= present, 1= absent; contact righting reflex: 0= present, 1= absent; evidence of biting: 0= no aggression, 1= aggressive; vocalization: 0= no, 1= yes.
