# Supplementary material for: Laboratory mouse housing conditions can be improved using common environmental enrichment without compromising data
Source: PLoS Biol. 2018 Apr 16;16(4):e2005019. doi: 10.1371/journal.pbio.2005019 (PMC5922977; doi:10.1371/journal.pbio.2005019)
Supplement: S2 Table — Affected animals are shown as portion of total examined animals of combined cohorts. (PDF) [file pbio.2005019.s005.pdf]

S2 Table. **Summary of background lesions in histopathological examination (pathology screen).**

strain	B6						D2					
**experimental group**	**control**		**nest**		**double**		**control**		**nest**		**double**	
**sex**	**m**	**f**	**m**	**f**	**m**	**f**	**m**	**f**	**m**	**f**	**m**	**f**
**Testes**												
focal vacuolation of germinal epithelium	0/6		0/6		2/6		0/6		1/5		0/5	
**seminal vesicle**												
focal hyperplasia	0/6		0/6		0/6		1/6		0/5		1/5	
**ovary**												
follicular cyst		1/6		0/6		0/6		0/6		0/5		0/5
**heart**												
calcification of epi- and mycardium	0/6	0/6	0/6	0/6	0/6	0/6	11/12	10/12	11/12	11/12	12/12	11/12
**lung**												
focal inflammatory infiltrates	0/6	1/6	0/6	0/6	0/6	0/6	0/6	0/6	0/5	0/5	0/5	1/5
focal vascular mineralization	0/6	0/6	0/6	0/6	0/6	0/6	0/6	0/6	0/5	0/5	0/5	1/5
**salivary gland (parotid)**												
basophilic hypertrophic foci	0/6	1/6	0/6	0/6	0/6	0/6	0/6	0/6	0/5	0/5	0/5	1/5
**salivary gland (submandibular)**												
focal lymphohistiocytic infiltration	0/6	0/6	0/6	1/6	0/6	0/6	0/6	0/6	0/5	0/5	0/5	1/5
**Thyroid gland**												
vacuolation of glandular epithelium	6/6	6/6	6/6	6/6	6/6	6/6	0/6	0/6	0/5	0/5	0/5	0/5
focal follicular cell hyperplasia	0/6	0/6	0/6	0/6	0/6	0/6	1/6	1/6	0/5	0/5	0/5	1/5
**parathyroid gland**												
small cyst	0/6	1/6	0/6	0/6	0/6	0/6	0/6	0/6	0/5	0/5	0/5	0/5
basophilic hypertrophic foci	0/6	0/6	0/6	0/6	0/6	0/6	0/6	0/6	0/5	0/5	0/5	1/5
**thymus**												
focal cyst	0/6	0/6	0/6	0/6	0/6	0/6	0/6	0/6	0/5	0/5	1/5	0/5
**liver**												
focal microgranulomas	2/6	5/6	4/6	2/6	3/6	3/6	1/6	4/6	0/5	3/5	2/5	3/5
eosinophilic foci	0/6	0/6	0/6	0/6	0/6	0/6	0/6	0/6	1/5	0/5	1/5	1/5
**adrenal gland**												
subcapsular cell hyperlasia (type A)	0/6	5/6	1/6	4/6	0/0	4/6	2/6	4/6	2/5	4/5	4/5	3/5
acessory cortical nodule	0/6	0/6	0/6	0/6	0/6	1/6	0/6	0/6	0/5	0/5	0/5	0/5
**pancreas**												
focal inflammatory infiltrates	1/6	1/6	0/6	2/6	0/6	1/6	1/6	1/6	0/5	2/5	0/5	2/5
foci of cellular alteration	0/6	0/6	0/6	0/6	0/6	0/6	1/6	3/6	0/5	1/5	0/5	0/5
**stomach**												
keratinic cyst	0/6	1/6	0/6	1/6	0/6	0/6	0/6	0/6	0/5	0/5	0/5	0/5
**small intestine**												
amyloidosis of lamina propria	0/6	0/6	0/6	0/6	0/6	0/6	0/6	0/6	0/5	2/5	0/5	2/5
**kidney**												
focal tubular regneration	0/6	0/6	0/6	0/6	0/6	1/6	0/6	0/6	0/5	0/5	0/5	0/5
transitional cell papilloma	0/6	0/6	0/6	0/6	0/6	1/6	0/6	0/6	0/5	0/5	0/5	0/5
**urinary bladder**												
focal inflammatory infiltration	0/6	0/6	0/6	0/6	0/6	0/6	0/6	1/6	0/5	0/5	0/5	0/5

Affected animals are shown as portion of total examined animals of combined cohorts.
