# Supplementary material for: Laboratory mouse housing conditions can be improved using common environmental enrichment without compromising data
Source: PLoS Biol. 2018 Apr 16;16(4):e2005019. doi: 10.1371/journal.pbio.2005019 (PMC5922977; doi:10.1371/journal.pbio.2005019)
Supplement: S1 Text — (PDF) [file pbio.2005019.s006.pdf]

S1 Text. **Results of morphological examination (dysmorphology screen).**

No morphological abnormalities were found in any of the following measured parameters, neither in B6, nor in D2 mice: Tail, coat, limbs, vibrissae, head, ears, snout, paws, digits, nails, skin, teeth, genitalia, mouth, lips.
