# Supplementary material for: Laboratory mouse housing conditions can be improved using common environmental enrichment without compromising data
Source: PLoS Biol. 2018 Apr 16;16(4):e2005019. doi: 10.1371/journal.pbio.2005019 (PMC5922977; doi:10.1371/journal.pbio.2005019)
Supplement: S2 Text — (PDF) [file pbio.2005019.s007.pdf]

S2 Text. **Reduction of animal numbers in single groups or screens.**

- All three cohorts, all groups: n=7 for eye screen; n=4-5 for pathology screen/macroscopical analysis; n=1-2 for pathology screen/histological analysis
- 1st cohort, all groups of B6: n=7 for cardiovascular screen/ECHO
- 1st cohort, group of B6/female/nest: n=9 for DEXA
- 1st cohort, group of D2/male/con: n=9 for IgE
- 1st cohort, group of D2/male/nest: n=9 for blood sampling, ABR and DEXA
- 1st cohort, group of D2/female/control: n=9 for blood sampling, ABR and DEXA
- 2nd cohort, group of B6/male/con: n=9 for ECHO
- 2nd cohort, group of B6/female/nest: n=9 for blood sampling, ABR and DEXA
- 2nd cohort, group of D2/female/nest: n=9 for blood sampling, ABR and DEXA
- 3rd cohort, all groups of B6: n=8 for calorimetry
- 3rd cohort, group of B6/male/con: n=7 for DEXA
- 3rd cohort, group of B6/male/double: n=7 for DEXA
- 3rd cohort, group of B6/female/con: n=9 for DEXA
- 3rd cohort, group of D2/female/nest: n=9 for ECHO, n=8 for IgE
- 3rd cohort, group of D2/male/con: n=9 for hematology, n=6 for Scheimpflug
- 3rd cohort, group of D2/male/nest: n=9 for blood sampling and ECG
- 3rd cohort, group of D2/male/double: n=8 for blood sampling, ABR and DEXA, n=7 for ECG, n=6 for Scheimpflug and virtual drum
- 3rd cohort, group of D2/female/nest: n=9 for blood sampling
- 3rd cohort, group of D2/female/double: n=9 for IgE
- Due to technical reasons one cohort of D2 mice could not be measured with DEXA analysis and is therefore not included in linear model analysis
- Due to technical reasons, parameters Glucose level fasted, AUC 0-30 and AUC 30-120 had to be excluded for B6 mice and were not included in linear model analysis.
